# Enantioselective Nickel‐Catalyzed Intramolecular Allylic Alkenylations Enabled by Reversible Alkenylnickel *E*/*Z* Isomerization

**DOI:** 10.1002/anie.201703380

**Published:** 2017-06-12

**Authors:** Connor Yap, Gabriel M. J. Lenagh‐Snow, Somnath Narayan Karad, William Lewis, Louis J. Diorazio, Hon Wai Lam

**Affiliations:** ^1^ The GSK Carbon Neutral Laboratories for Sustainable ChemistryUniversity of NottinghamJubilee CampusTriumph RoadNottinghamNG7 2TUUK; ^2^ School of ChemistryUniversity of NottinghamUniversity ParkNottinghamNG7 2RDUK; ^3^ AstraZenecaPharmaceutical Technology and DevelopmentEtherow F53/20, Charter WayMacclesfieldCheshireSK10 2NAUK

**Keywords:** allylic substitution, asymmetric catalysis, cyclization, isomerization, nickel

## Abstract

Enantioselective nickel‐catalyzed arylative cyclizations of substrates containing a *Z*‐allylic phosphate tethered to an alkyne are described. These reactions give multisubstituted chiral aza‐ and carbocycles, and are initiated by the addition of an arylboronic acid to the alkyne, followed by cyclization of the resulting alkenylnickel species onto the allylic phosphate. The reversible *E*/*Z* isomerization of the alkenylnickel species is essential for the success of the reactions.

Enantioselective metal‐catalyzed allylic substitutions of achiral or racemic substrates using carbon‐centered nucleophiles are a major class of reactions for preparing enantioenriched chiral compounds.^1^ Although numerous developments have been described,^1^ there are only a few reports of the enantioselective allylation of alkenyl nucleophiles.^2^ Chiral copper–N‐heterocyclic carbene catalysts are highly effective in the enantioselective additions of alkenylaluminum,2a–2c alkenylboron,2d,2f,2h and allenylboron2e reagents to achiral allylic phosphates. Chiral iridium2g and rhodium2i catalysts are also effective in enantioselective additions of alkenylboron reagents to racemic allylic alcohols2g and allylic halides,2i respectively.

While the aforementioned examples provide valuable enantioenriched chiral 1,4‐diene building blocks,^2^ several aspects remain underdeveloped. For example, reactions involving fully substituted alkenyl nucleophiles are rare.^3^ The integration of these reactions into domino processes that form more than one new carbon–carbon bond is also not well‐established.^3^ Murakami and co‐workers have partially addressed these issues by developing rhodium‐catalyzed cyclizations of 1,6‐enynes, in which the reaction is triggered by addition of an arylboronic acid to the alkyne (Scheme 1 A).^3^, ^4^ These reactions give cyclopentanes containing a tetrasubstituted exocyclic alkene.3a,3b However, only two enantioselective reactions were reported, and low selectivity was observed in the initial addition to the alkyne, which led to other products being formed.3b Therefore, the availability of other methods to meet these challenges would significantly enhance the utility of domino alkyne carbometalation–allylic alkenylations.

**Scheme 1:**
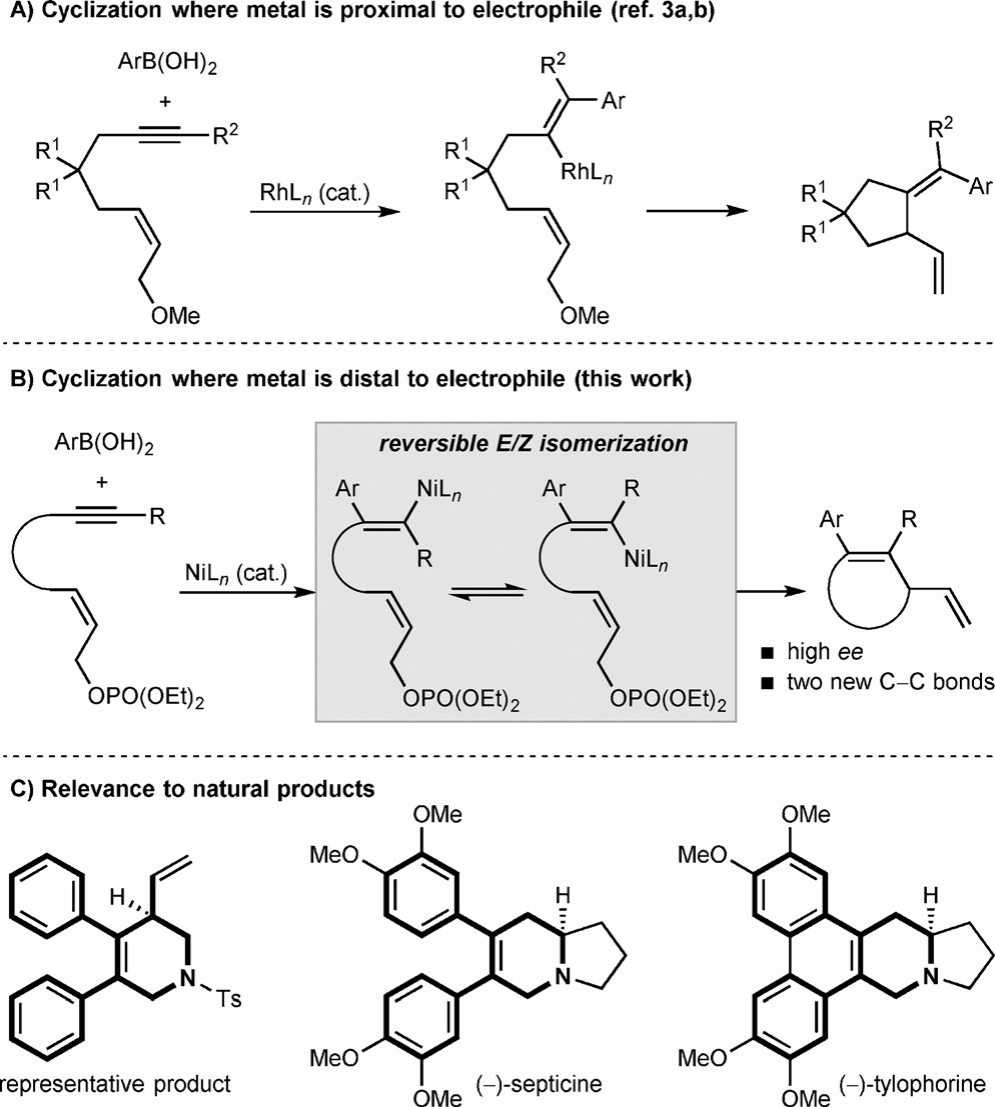
Arylative cyclizations of enynes.

Here, we describe highly enantioselective intramolecular alkenylations of allylic phosphates with fully substituted alkenylnickel species, which are themselves generated by the nickel‐catalyzed addition of arylboronic acids to internal alkynes (Scheme 1 B). Notably, this process gives chiral 1,4‐diene‐containing hetero‐ and carbocycles that are inaccessible from these substrates with established conditions using rhodium catalysis.^3^ These include 4,5‐diaryl‐1,2,3,6‐tetrahydropyridines, which are seen in naturally occurring alkaloids such as (−)‐septicine and (−)‐tylophorine (Scheme 1 C).^5^

Our studies began with the arylative cyclization of substrate **1 a**, which contains a *Z*‐allylic phosphate tethered to an alkyne (Table 1). Guided by our recent work,^6^ we anticipated that arylnickelation of the alkyne to place nickel distal to the electrophile, followed by reversible *E*/*Z* isomerization of the alkenylnickel species, would provide a species capable of cyclizing onto the allylic phosphate to give products of formal *anti*‐carbometalation (Scheme 1 B).^7^ However, of the existing reports of enantioselective nickel‐catalyzed allylic substitutions with carbon‐centered nucleophiles,^8^, ^9^ none describe the use of alkenyl nucleophiles, and success in this ring‐closing step was therefore uncertain.

**Table 1:** Evaluation of reaction conditions.^[a]^ 
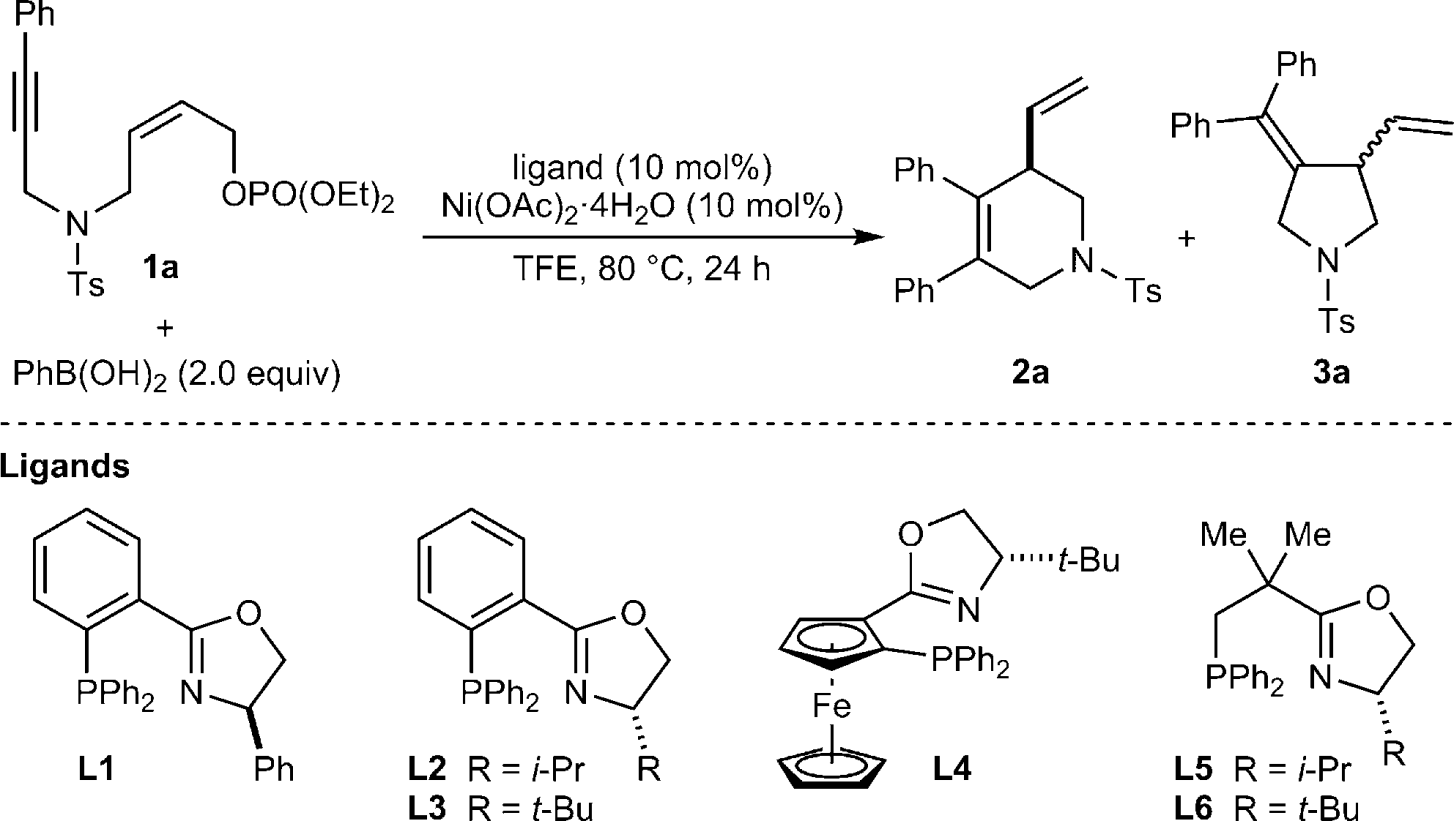

Entry	Ligand	**2 a**:**3 a** ^[b]^	Combined yield [%]^[c]^	*ee* of **2 a** [%]^[d]^
1	**L1**	10:1	46	−36^[*e*]^
2	**L2**	10:1	44	80
3	**L3**	10:1	60	98
4	**L4**	–	<5	–
5	**L5**	>19:1	80	90
6	**L6**	>19:1	33	>99
7^[f]^	**L6**	>19:1	80	96

[a] Reactions were conducted using 0.05 mmol of **1 a**. [b] Determined by ^1^H NMR analysis of the crude reactions. [c] Determined by ^1^H NMR analysis using 1,4‐dimethoxybenzene as an internal standard. [d] Determined by HPLC analysis on a chiral stationary phase. [e] Major product was the enantiomer of **2 a**. [f] Conducted at 100 °C.

We were pleased to observe that reaction of **1 a** with PhB(OH)_2_ (2.0 equiv) in the presence of Ni(OAc)_2_⋅4 H_2_O (10 mol %) and various chiral P,N‐ligands (10 mol %) in mixtures of MeCN with THF or 2‐MeTHF did indeed provide the *anti*‐carbometallative cyclization product **2 a**. However, these reactions proceeded in low conversions (<10 %) and **2 a** was accompanied by comparable amounts of pyrrolidine **3 a**, resulting from arylnickelation of the alkyne with the opposite regioselectivity. No reaction was observed in other solvents such as DMF, dioxane, and toluene. Fortunately, in 2,2,2‐trifluoroethanol (TFE), reactions conducted with phosphinooxazoline (PHOX) ligands **L1**–**L3** 10 gave **2 a** in moderate NMR yields and in 36–98 % *ee*, accompanied by only a small quantity of pyrrolidine **3 a** (entries 1–3).^11^ (*S*,*S*)‐*t*‐Bu‐FOXAP (**L4**) gave no reaction (entry 4). Improved selectivities (>19:1) in favor of **2 a** were obtained using NeoPHOX ligands^12^ **L5** and **L6** (entries 5–7).^13^ Although the enantioselectivity was higher using (*S*)‐*t*‐Bu‐NeoPHOX (**L6**) (compare entries 5 and 6), the conversion was modest (entry 6). However, increasing the temperature to 100 °C gave a significantly higher yield of **2 a** with only a small decrease in enantioselectivity (entry 7). The conditions of entry 7 were subsequently employed to test the generality of this process.^14^

The scope of this reaction with respect to the alkyne‐tethered allylic phosphate was then explored in reactions with PhB(OH)_2_, which gave products **2 a**–**2 i** in 45–92 % yield and 49–99 % *ee* (Scheme 2). High selectivities (≥14:1) in favor of the six‐membered ring products were observed. Regarding the substituent on the alkyne, the reaction is compatible with a phenyl group (**2 a**), various *para*‐ (**2 b** and **2 c**), *meta*‐ (**2 d**), and *ortho*‐substituted benzenes (**2 e**), and a 2‐thienyl group (**2 f**). An alkenyl group on the alkyne is also tolerated, though the product **2 g** was formed in 49 % *ee*. The reaction of a substrate containing a methyl group on the alkyne gave only a complex mixture of products. Replacement of the *p*‐toluenesulfonyl group with a 4‐nitrobenzenesulfonyl group is possible (**2 h**). Finally, changing the linking group between the alkyne and the allylic phosphate to an all‐carbon tether enabled the formation of carbocycle **2 i** in 51 % yield and 96 % *ee*.

**Scheme 2:**
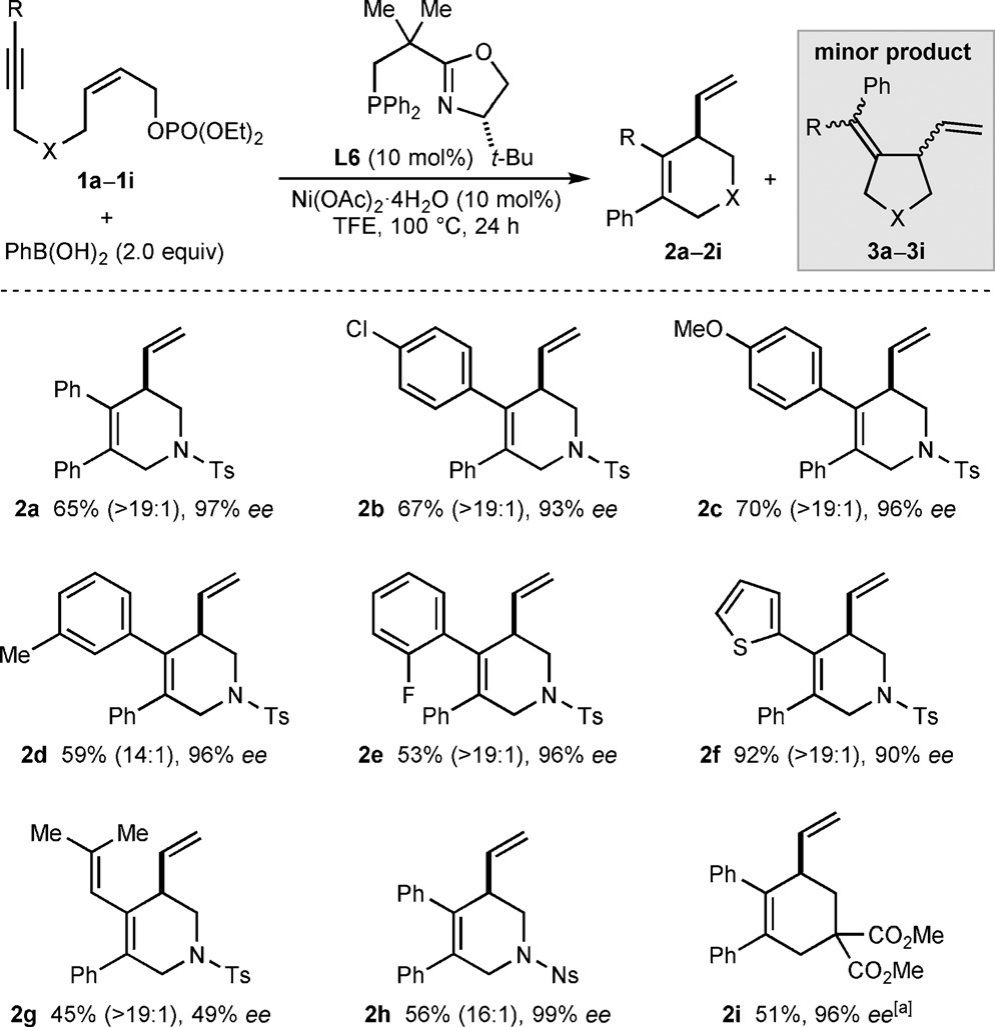
Scope of 1,6‐enynes. Reactions were conducted using 0.30 mmol of **1**. Yields are of isolated products. Values in parentheses refer to the ratio of **2**:**3** as determined by ^1^H NMR analysis of the crude reactions. Unless stated otherwise, the minor isomers **3** were not evident in the isolated products. Enantiomeric excesses were determined by HPLC analysis on a chiral stationary phase. [a] Product **2 i** contained trace quantities of inseparable, unidentified impurities, and the ratio of **2 i**:**3 i** could not be determined.

Pleasingly, this process is compatible with a range of other (hetero)arylboronic acids, which gave products **2 j**–**2 r** in 47–66 % yield and 96–99 % *ee* from three different 1,6‐enynes (Scheme 3). The scope includes *para*‐ (**2 p** and **2 q**), *meta*‐ (**2 j** and **2 r**), *ortho*‐ (**2 m**), and disubstituted phenylboronic acids (**2 k** and **2 n**), containing methyl (**2 k** and **2 p**), halide (**2 j**, **2 m**, and **2 n**), alkoxy (**2 n**), nitrile (**2 q**), or ester groups (**2 r**). In addition, 3‐thienylboronic acid (**2 l**) and 2‐naphthylboronic acid (**2 o**) are also tolerated.

**Scheme 3:**
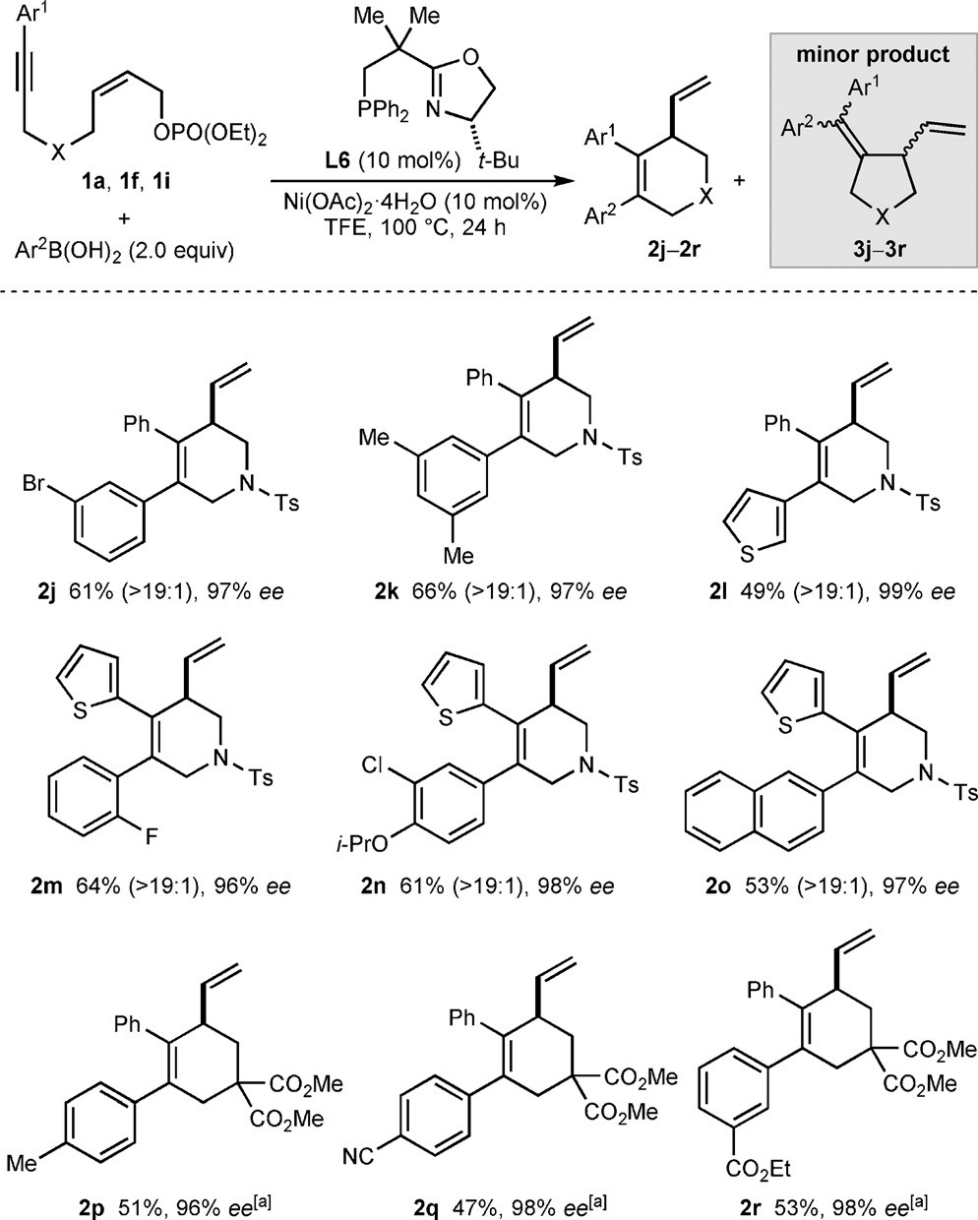
Scope of boronic acids. Reactions were conducted using 0.30 mmol of **1**. Yields are of isolated products. Values in parentheses refer to the ratio of **2**:**3** as determined by ^1^H NMR analysis of the crude reactions. Unless stated otherwise, the minor isomers **3** were not evident in the isolated products. Enantiomeric excesses were determined by HPLC analysis on a chiral stationary phase. [a] Products **2 p**–**2 r** contained trace quantities of inseparable, unidentified impurities, and the ratio of **2**:**3** could not be determined.

Further studies of the scope of the alkyne‐tethered allylic phosphate are shown in Equations (1)–(3). A trimethylsilyl‐substituted alkyne **1 j** gave low conversions under the standard conditions, but replacing ligand **L6** with **L5** gave **2 s** in 70 % yield and 69 % *ee* [Eq. 1]. The *Z*‐stereochemistry of the allylic phosphate appears to be essential, as *E*‐allylic phosphate **4** only underwent hydroarylation to give **5** in 63 % yield, as a 2:1 ratio of geometric isomers [Eq. 2, stereochemistry of **5** not assigned]. Although the reasons for this phenomenon are not understood, one possibility is that the steric demands of this reaction are better accommodated by *Z*‐allylic phosphates. The standard conditions were ineffective in the formation of a cyclopentene from 1,5‐enyne **6**, but use of (*R*)‐Ph‐PHOX (**L1**) in place of **L6** gave **7** in 64 % yield and 42 % *ee* [Eq. 3].
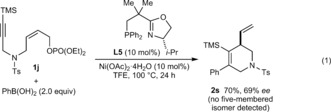


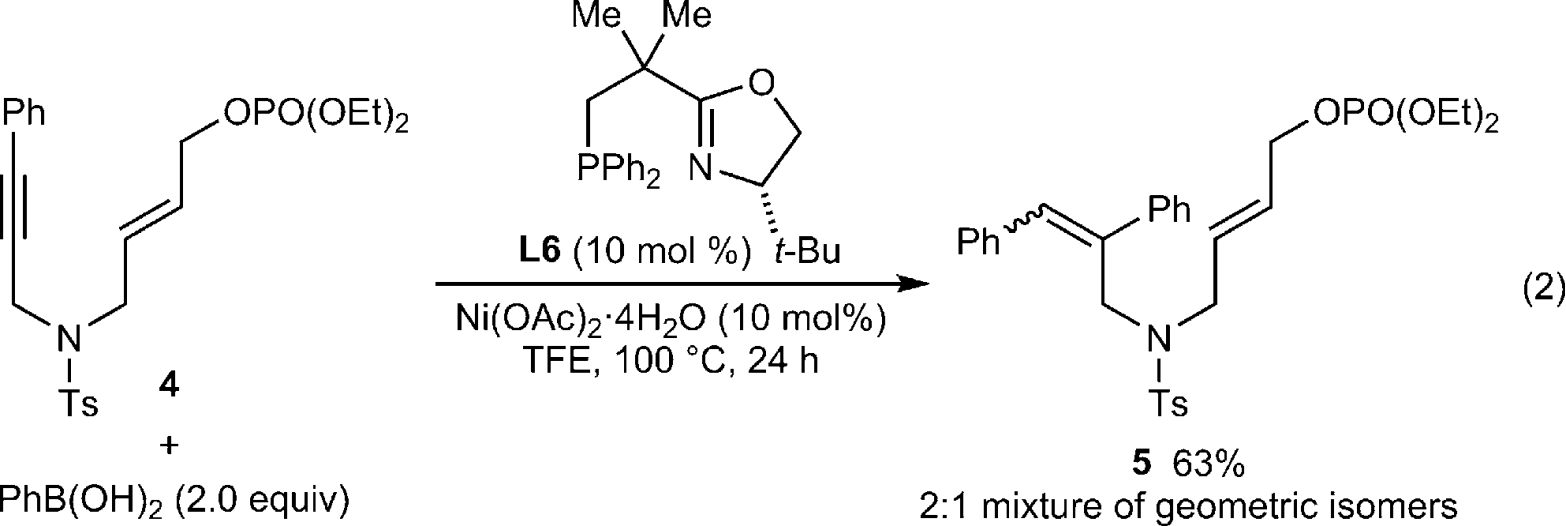


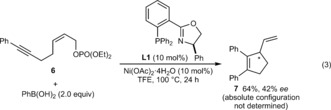



Finally, replacing the arylboronic acid with other pronucleophilic species was examined. Although no reaction occurred with methylboronic acid, (*E*)‐2‐phenylvinylboronic acid reacted with **1 a** to give **8** in 98 % *ee*, albeit in 13 % yield [Eq. 4].
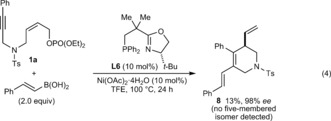



Scheme 4 illustrates a possible catalytic cycle for these reactions, using **1 a** and PhB(OH)_2_ as representative reactants. Transmetalation of PhB(OH)_2_ with the chiral nickel species **9**, which could have hydroxide, acetate, 2,2,2‐trifluoroethoxide, or diethylphosphate ligands resulting from the possible species in the reaction, gives arylnickel species **10**. *Syn*‐phenylnickelation of **1 a** gives alkenylnickel species (*E*)‐**11**, which undergoes reversible *E*/*Z* isomerization to give (*Z*)‐**11**.^6^, 7a, 15 The mechanism of *E*/*Z* isomerization is not currently known, but may involve the intermediacy of zwitterionic carbene‐type species.15a, 16 Migratory insertion of the alkene of the allylic phosphate into the carbon–nickel bond of (*Z*)‐**11** gives alkylnickel species **12**, from which β‐phosphate elimination would liberate the product **2 a**, regenerating the nickel species **9**. This mechanism for allylic substitution^17^ is similar to that proposed by Murakami and co‐workers for arylative cyclizations of 1,6‐enynes.3a,3b Furthermore, it stands in contrast to other related Ni‐catalyzed allylic substitutions, which are thought to proceed through allylnickel intermediates.1j, 8j–8l, 18 However, although we have proposed that nickel remains in the +2 oxidation state throughout, we do not rule out alternative mechanisms involving Ni^I^ species.7a

**Scheme 4:**
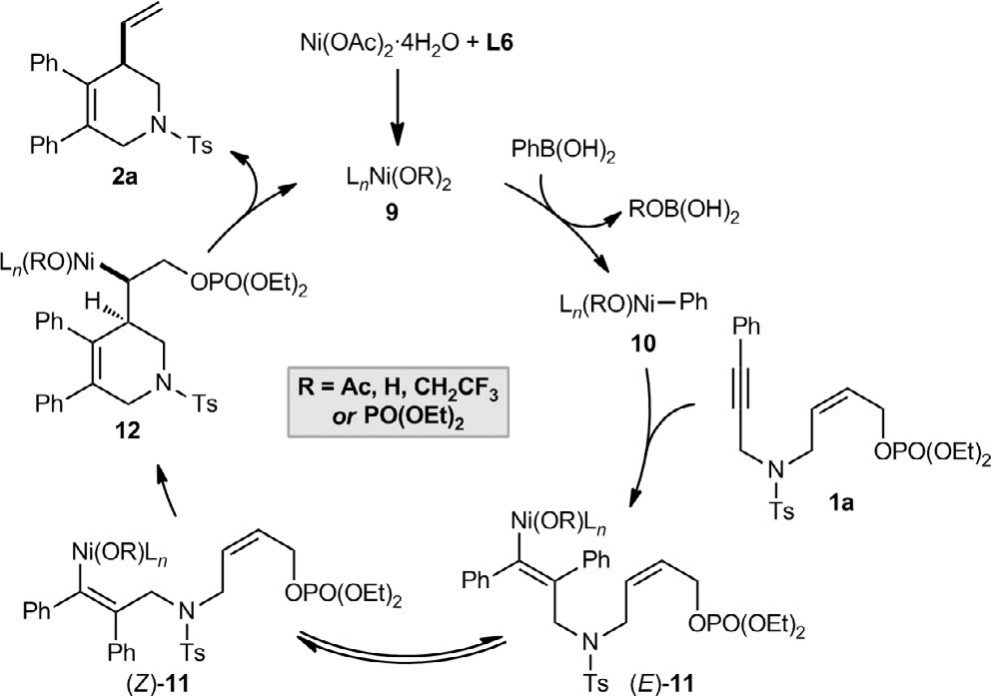
Postulated catalytic cycle.

In conclusion, we have reported enantioselective nickel‐catalyzed allylic alkenylations of allylic phosphates which provide a range of chiral 1,4‐diene‐containing hetero‐ and carbocycles in high enantiomeric excesses. This reaction further demonstrates the power of the reversible *E*/*Z* isomerization of alkenylnickel species in providing products that would otherwise be inaccessible using *syn*‐selective alkyne carbometalation processes.

## Conflict of interest

The authors declare no conflict of interest.

## Supporting information

As a service to our authors and readers, this journal provides supporting information supplied by the authors. Such materials are peer reviewed and may be re‐organized for online delivery, but are not copy‐edited or typeset. Technical support issues arising from supporting information (other than missing files) should be addressed to the authors.

Supplementary
