# Supplementary material for: Enantioselective Nickel‐Catalyzed Intramolecular Allylic Alkenylations Enabled by Reversible Alkenylnickel E/Z Isomerization
Source: Angew Chem Int Ed Engl. 2017 Jun 12;56(28):8216–20. doi: 10.1002/anie.201703380 (PMC5499723; doi:10.1002/anie.201703380)
Supplement: Supplementary file 1 — Supplementary [file ANIE-56-8216-s001.pdf]

Angewandte Chemie
Eine Zeitschrift der Gesellschaft Deutscher Chemiker

Supporting Information

Enantioselective Nickel-Catalyzed Intramolecular Allylic Alkenylations Enabled by Reversible Alkenylnickel *E*/*Z* Isomerization

*Connor Yap, Gabriel M. J. Lenagh-Snow, Somnath Narayan Karad, William Lewis, Louis J. Diorazio, and Hon Wai Lam**

anie_201703380_sm_miscellaneous_information.pdf

Author Contributions

C.Y. Conceptualization: Equal; Data curation: Lead; Formal analysis: Lead; Investigation: Lead; Methodology: Lead; Validation: Equal; Writing—review & editing: Supporting
G.L. Data curation: Supporting; Formal analysis: Supporting; Investigation: Supporting; Methodology: Supporting; Validation: Supporting; Writing—review & editing: Supporting
S.K. Data curation: Supporting; Formal analysis: Supporting; Investigation: Supporting; Methodology: Supporting; Validation: Supporting; Writing—review & editing: Supporting
W.L. Data curation: Supporting; Formal analysis: Supporting; Validation: Supporting
L.D. Supervision: Supporting; Writing—review & editing: Supporting
H.L. Conceptualization: Equal; Formal analysis: Supporting; Funding acquisition: Lead; Supervision: Lead; Validation: Supporting; Writing—original draft: Lead; Writing—review & editing: Lead.

Supporting Information

Supporting Information

General Information....................2
Preparation of Enynes....................3
Enantioselective Nickel-Catalyzed Intramolecular Allylic Substitution....................17
Further Exploration of Substrate Scope....................29
NMR Spectra....................33
HPLC Traces....................75
References....................96

General Information

All air-sensitive reactions were carried out under an inert atmosphere using oven-dried apparatus. 2,2,2-Trifluoroethanol was purchased from Acros Organics and degassed before use using the freeze-pump-thaw technique. All commercially available reagents were used as received unless otherwise stated. Petroleum ether refers to Sigma-Aldrich product 24587 (petroleum ether boiling point 40-60 °C). Thin layer chromatography (TLC) was performed on Merck DF-Alufoilien 60F254 0.2 mm precoated plates. Compounds were visualized by exposure to UV light or by dipping the plates into solutions of potassium permanganate or vanillin followed by gentle heating. Flash column chromatography was carried out using silica gel (Fisher Scientific 60 Å particle size 35-70 micron or Fluorochem 60 Å particle size 40-63 micron). Melting points were recorded on a Gallenkamp melting point apparatus and are uncorrected. The solvent of recrystallization is reported in parentheses. Infra-red (IR) spectra were recorded on either a Shimadzu IRAffinity-1 or a Nicolet Avatar 360 FT instrument on the neat compound using the attenuated total refraction technique. NMR spectra were acquired on Bruker AV500, Bruker AV500(III)HD, Bruker AV400, Bruker AV(III)400HD, or Bruker DPX400 spectrometers. ^{1}H and ^{13}C NMR spectra were referenced to external tetramethylsilane *via* the residual protonated solvent (^{1}H) or the solvent itself (^{13}C). All chemical shifts are reported in parts per million (ppm). For $CDCl_3$, the shifts are referenced to 7.27 ppm for ^{1}H NMR spectroscopy and 77.0 ppm for ^{13}C NMR spectroscopy. Coupling constants (*J*) are quoted to the nearest 0.1 Hz. Assignments were made using the DEPT sequence with secondary pulses at 90° and 135°. High-resolution mass spectra were recorded using electrospray ionization (ESI) or Gas Chromatography Mass Spectrometry (GC/MS) techniques. X-ray diffraction data were collected at 120 K on an Agilent SuperNova diffractometer using CuKα radiation. Chiral HPLC analysis was performed on an Agilent 1290 series or Agilent 1260 series instrument using 4.6 × 250 mm columns. 2-[2-(Diphenylphosphino)ethyl]pyridine (Sigma-Aldrich product 695599) was used as an achiral ligand to obtain authentic racemic compounds. Ligand (**L6)** was prepared according to the literature procedure.[1]

Preparation of Enynes

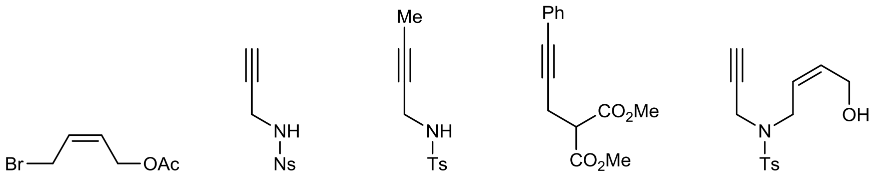

S1 **S2** **S3** **S4** **S5**

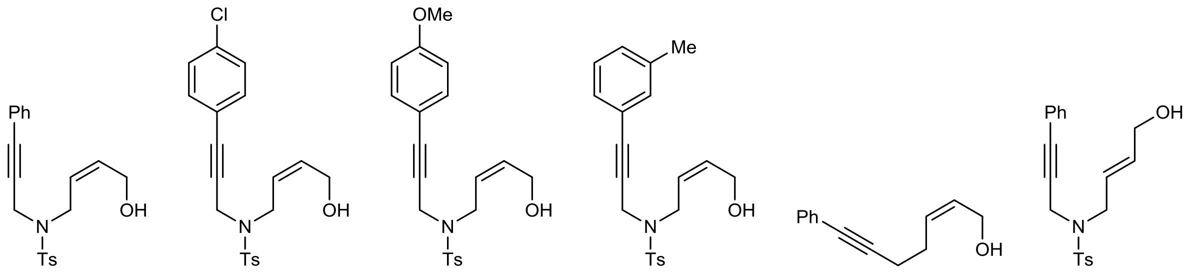

S6 **S7** **S8** **S9** **S10** **S11**

Alkene **S1** was prepared according to a previously reported procedure.[2] **S2**,[3] **S3**,[4] **S4**,[5] **S5**,[6] **S6**,[6] **S7**,[7] **S8**,[8] **S9**,[9] **S10**,[10] and **S11**[7] were prepared according to previously reported procedures.

Preparation of Substrates 1a-1h

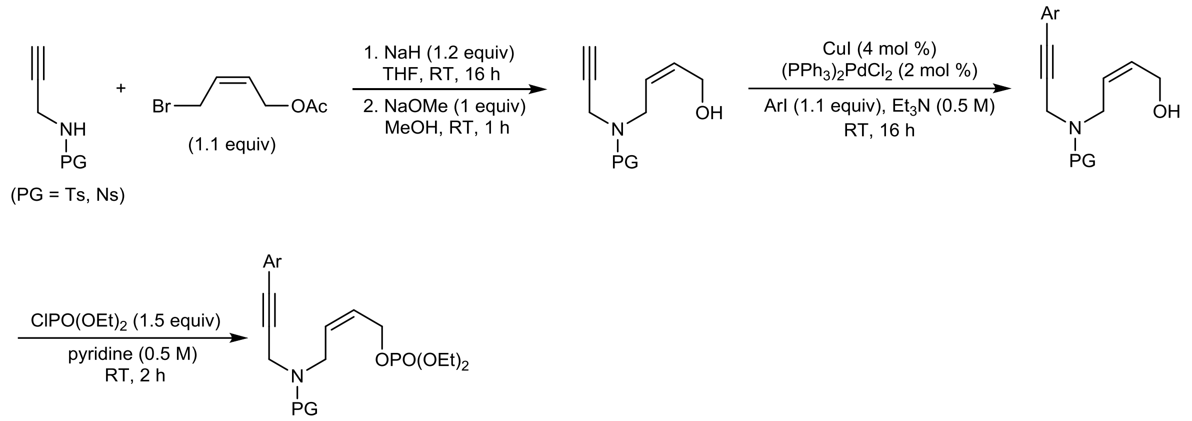

(*Z*)-*N*-(4-Hydroxybut-2-en-1-yl)-4-nitro-*N*-(prop-2-yn-1-yl)benzenesulfonamide (S12)

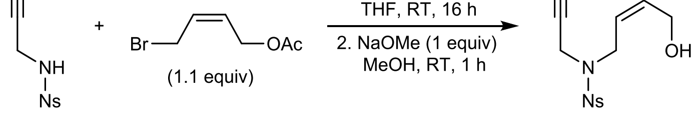

S2 **S1** **S12**

Alkynyl sulfonamide **S2**[3] (3.23 g, 13.4 mmol) was added in one portion to a stirred suspension of NaH (588 mg, 14.7 mmol, 60% dispersion in mineral oil) in anhydrous THF (100 mL) under argon at 0 °C. The ice bath was then removed and the mixture stirred at room temperature for 45 min. A solution of (*Z*)-4-bromobut-2-en-1-yl acetate **S1**[2] (2.85 g, 14.7 mmol) in anhydrous THF (14 mL) was

then added *via* syringe and the resulting mixture stirred at room temperature for 18 h. The reaction was then quenched with H_2O, diluted with EtOAc (400 mL), washed with H_2O (100 mL), brine (100 mL), dried ($MgSO_4$) and concentrated *in vacuo* to afford the crude allylic acetate. This material was dissolved in MeOH (30 mL) under argon, NaOMe (726 mg, 13.4 mmol) added and the mixture stirred at room temperature for 1 h. The mixture was concentrated *in vacuo* and the residue dissolved in EtOAc (100 mL), washed with H_2O (50 mL), brine (50 mL), dried ($MgSO_4$), filtered and concentrated *in vacuo* to give the crude alcohol. Purification by flash column chromatography (40 to 50% EtOAc:petroleum ether) gave *enyne* **S12** (2.89 g, 69% over 2 steps) as a pale orange solid. R_f = 0.25 (50% EtOAc:petroleum ether); m.p. 63-65 °C (Et_2O); IR 3721 (OH), 3277 (≡CH), 2030, 1695, 1526, 1345, 1308, 1232, 1088, 855, 776, 656 cm^{-1}; ^1H NMR (400 MHz, $CDCl_3$) δ 8.37 (2H, d, *J* = 8.9 Hz, Ar**H**), 8.06 (2H, d, *J* = 8.9 Hz, Ar**H**), 5.93 (1H, dtt, *J* = 11.0, 6.6, 1.4 Hz, =C**H**CH_2O), 5.52 (1H, dtt, *J* = 11.0, 7.3, 1.4 Hz, NCH_2C**H**=), 4.31-4.21 (2H, m, =CHC**H**$_2$O), 4.17 (2H, d, *J* = 2.4 Hz, ≡CC**H**$_2$N), 4.04-3.89 (2H, m, NC**H**$_2$CH=), 2.07 (1H, t, *J* = 2.4 Hz, ≡C**H**); ^{13}C NMR (100.6 MHz, $CDCl_3$) δ 150.2 (C), 144.6 (C), 135.0 (CH), 129.0 (2 × CH), 124.7 (CH), 124.2 (2 × CH), 75.9 (C), 74.5 (CH), 58.1 (CH_2), 43.2 (CH_2), 36.1 (CH_2); HRMS (ESI) Exact mass calculated for $[C_{13}H_{14}N_2NaO_5S]^+$ $[M+Na]^+$: 333.0516, found 333.0510.

N.B. The ^1H NMR peak corresponding to (O**H**) overlaps with the residual water peak in the spectrum.

General Procedure A: Sonogashira Coupling

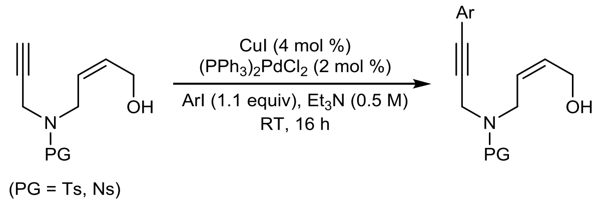

$(PPh_3)_2PdCl_2$ (2 mol %), CuI (4 mol %), the appropriate terminal alkyne and a stirrer bar were placed in a 20 mL microwave vial fitted with a septum cap and the contents evacuated and charged with argon (3 cycles). Et_3N (0.5 M) was then added and the contents stirred until dissolved. The appropriate aryl halide (1.1 equiv) was then added and the resulting mixture was stirred at room temperature for 16 h. The contents were diluted with EtOAc (10 mL per mmol of alkyne), washed with 10% aqueous HCl solution (2 × organic volume), 50% brine (3 × organic volume), dried ($MgSO_4$), filtered and concentrated *in vacuo* to afford the crude material. Dry loading and purification by flash column chromatography (EtOAc:petroleum ether) afforded the title compounds.

N.B. Dry loading of the crude material is essential for obtaining good yields of material. In our hands, wet loading of the compounds resulted in significantly lower yields.

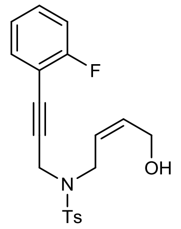

(*Z*)-*N*-[3-(2-Fluorophenyl)prop-2-yn-1-yl]-*N*-(4-hydroxybut-2-en-1-yl)-4-methylbenzenesulfonamide (S13). Prepared according to General Procedure A, using enyne **S5**[6] (500 mg, 1.78 mmol), 2-fluoroiodobenzene (226 μL, 1.95 mmol), $(PPh_3)_2PdCl_2$ (24.9 mg, 0.03 mmol), CuI (13.5 mg, 0.07 mmol) and Et_3N (4 mL). Purification by flash column chromatography (40 to 60% EtOAc:petroleum ether) gave *enyne* **S13** (610 mg, 91%) as a dark orange oil. R_f = 0.28 (50% EtOAc:petroleum ether); IR 3690 (OH), 3053, 2851, 1610, 1597, 1942, 1425, 1337, 1320, 1288, 1215, 1183, 1154, 963, 818, 677, 582 cm^{-1}; ^{1}H NMR (400 MHz, $CDCl_3$) δ 7.77 (2H, d, *J* = 8.3 Hz, Ar**H**), 7.33-7.22 (3H, m, Ar**H**), 7.11-6.99 (3H, m, Ar**H**), 5.92 (1H, dtt, *J* = 11.0, 6.7, 1.4 Hz, =C**H**CH_2OH), 5.58 (1H, dtt, *J* = 11.0, 7.4, 1.4 Hz, NCH_2C**H**=), 4.35 (2H, s, ≡CC**H**$_2$N), 4.26 (2H, d, *J* = 6.7, 1.4 Hz, =CHC**H**$_2$OH), 3.98 (2H, dd, *J* = 7.4, 1.4 Hz, NC**H**$_2$CH=), 2.29 (3H, s, ArC**H**$_3$); ^{13}C NMR (100.6 MHz, $CDCl_3$) δ 162.7 (d, $^1J_{C\text{-}F}$ = 251.6 Hz, C), 143.7 (C), 135.54 (C), 134.45 (CH), 133.3 (CH), 130.3 (d, $^3J_{C\text{-}F}$ = 8.0 Hz, CH), 129.5 (2 × CH), 127.8 (2 × CH), 125.6 (CH), 123.8 (d, $^4J_{C\text{-}F}$ = 3.7 Hz, CH), 115.4 (d, $^2J_{C\text{-}F}$ = 20.6 Hz, CH), 110.5 (d, $^2J_{C\text{-}F}$ = 15.6 Hz, C), 86.9 (d, $^3J_{C\text{-}F}$ = 3.6 Hz, C), 79.2 (C), 58.1 (CH_2), 43.1 (CH_2), 36.9 (CH_2), 21.4 (CH_3); HRMS (ESI) Exact mass calculated for $[C_{20}H_{20}FNNaO_3S]^+$ [M + Na]$^+$: 396.1040, found 396.1042.

N.B. The ^{1}H NMR peak corresponding to (O**H**) overlaps with the residual water peak in the spectrum.

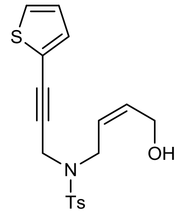

(*Z*)-*N*-(4-Hydroxybut-2-en-1-yl)-4-methyl-*N*-(3-(thiophen-2-yl)prop-2-yn-1-yl)benzenesulfonamide (S14). Prepared according to General Procedure A, using enyne **S5**[6] (1.00 g, 3.57 mmol), 2-iodothiophene (432 μL, 3.93 mmol), $(PPh_3)_2PdCl_2$ (50.2 mg, 0.07 mmol), CuI (27.2 mg, 0.14 mmol) and Et_3N (7 mL). Purification by flash column chromatography (30 to 50% EtOAc:petroleum ether) gave *enyne* **S14** (779 mg, 60%) as a dark yellow oil. R_f = 0.31 (50% EtOAc:petroleum ether); IR 3543 (OH), 2920, 2223 (C≡C), 1596, 1425, 1190, 1156, 948, 847, 705 cm^{-1}; ^{1}H NMR (400 MHz, $CDCl_3$) δ 7.76 (2H, d, *J* = 8.3 Hz, Ar**H**), 7.33-7.28 (2H, m, Ar**H**), 7.22 (1H, dd, *J* = 5.1, 1.2 Hz, Ar**H**), 6.96 (1H, dd, *J* = 3.7, 1.2 Hz, Ar**H**), 6.92 (1H, dd, *J* = 5.1, 3.6 Hz, Ar**H**), 5.91 (1H, dtt, *J* = 11.0, 6.7, 1.4 Hz, =C**H**CH_2OH), 5.56 (1H, dtt, *J* = 11.0, 7.4 , 1.4 Hz, NCH_2C**H**=), 4.32 (2H, s, ≡CC**H**$_2$N), 4.27-4.22 (2H, br m, =CHC**H**$_2$OH), 3.94 (2H, dd, *J* = 7.4 Hz, 1.4 Hz, NC**H**$_2$CH=), 2.39 (3H, s, ArC**H**$_3$), 1.68-1.66 (1H, br s, O**H**); ^{13}C NMR (100.6 MHz, $CDCl_3$) δ 143.8 (C), 135.3 (C), 134.4 (CH), 132.3 (CH), 129.7 (2 × CH), 127.7 (2 × CH), 127.4 (CH), 126.8 (CH), 125.5 (CH), 121.7 (C), 85.5 (C), 79.1 (C), 58.1 (CH_2), 43.2 (CH_2), 37.0 (CH_2), 21.5 (CH_3); HRMS (ESI) Exact mass calculated for $[C_{18}H_{20}NO_3S_2]^+$ [M+H]$^+$: 362.0879, found 362.0880.

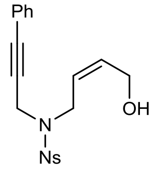

(*Z*)-*N*-(4-Hydroxybut-2-en-1-yl)-4-nitro-*N*-(3-phenylprop-2-yn-1-yl)benzenesulfonamide (S15). Prepared according to General Procedure A, using enyne **S12** (1.00 g, 3.22 mmol), iodobenzene (396 μL, 3.54 mmol), $(PPh_3)_2PdCl_2$ (44.9 mg, 0.06 mmol), CuI (24.3 mg, 0.12 mmol) and Et_3N (10 mL). Purification by flash column chromatography (30 to 50% EtOAc:petroleum ether) gave *enyne* **S15** (900 mg, 72%) as an orange solid. R_f = 0.41 (50% EtOAc:petroleum ether); m.p. 104-106 °C (Et_2O); IR 3527 (OH), 2931, 1608, 1427, 1306, 1157, 1056, 962, 852, 765, 710, 686, 565 cm^{-1}; ^1H NMR (500 MHz, $CDCl_3$) δ 8.29 (2H, d, *J* = 8.8 Hz, Ar**H**), 8.09 (2H, d, *J* = 8.8 Hz, Ar**H**), 7.33-7.29 (1H, m, Ar**H**), 7.26-7.22 (2H, m, Ar**H**), 7.06-7.03 (2H, m, Ar**H**), 5.96 (1H, dtt, *J* = 11.0, 6.6, 1.3 Hz, =C**H**CH_2OH), 5.59 (1H, dtt, *J* = 11.0, 7.4, 1.5 Hz, NCH_2C**H**=), 4.37 (2H, s, ≡C$\mathbf{H}_2$N), 4.28 (2H, d, *J* = 6.6 Hz, =CHC$\mathbf{H}_2$OH), 4.10-3.97 (2H, m, NC$\mathbf{H}_2$CH=), 1.53-1.47 (1H, br, O**H**); ^{13}C NMR (100.6 MHz, $CDCl_3$) δ 150.1 (C), 144.6 (C), 135.0 (CH), 131.2 (2 × CH), 129.1 (CH), 129.0 (2 × CH), 128.5 (2 × CH), 124.9 (CH), 124.1 (2 × CH), 121.2 (C), 86.4 (C), 80.8 (C), 58.1 (CH_2), 43.4 (CH_2), 37.0 (CH_2); HRMS (ESI) Exact mass calculated for $[C_{19}H_{18}N_2NaO_5S]^+$ $[M+Na]^+$: 409.0829, found 409.0828.

(*Z*)-*N*-(4-Hydroxybut-2-en-1-yl)-4-methyl-*N*-(5-methylhex-4-en-2-yn-1-yl)benzenesulfonamide (S16)

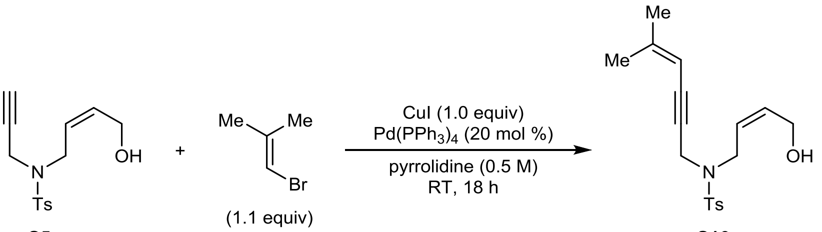

$Pd(PPh_3)_4$ (624 mg, 0.54 mmol) and CuI (510 mg, 2.68 mmol) were added to a flask which was then sealed and purged with N_2. Degassed pyrrolidine (18 mL) was then added and the resulting solution sparged with N_2 for 10 mins. Enyne **S5**[6] (748 mg, 2.68 mmol) and 1-bromo-2-methyl-1-propene (300 μL, 2.95 mmol) were then added, and the mixture was stirred at room temperature for 18 h. The mixture was diluted with H_2O (100 mL), and extracted with EtOAc (2 × 100 mL). The combined organic layers were washed with brine (20 mL), dried ($MgSO_4$), filtered and concentrated *in vacuo* to afford the crude 1,3-enyne. Purification by flash column chromatography (50% EtOAc:petroleum ether) gave *dienyne* **S16** (700 mg, 56%) as a dark brown oil. R_f = 0.46 (50% EtOAc:petroleum ether); IR 3479 (OH), 2923, 1719, 1597, 1493, 1438, 1334, 1155, 1089, 1035, 899, 813, 733, 660, 586, 543 cm^{-1}; ^1H NMR (400 MHz, $CDCl_3$) δ 7.73 (2H, d, *J* = 8.3 Hz, Ar**H**), 7.28 (2H, d, *J* = 8.3 Hz, Ar**H**), 5.90-5.83 (1H, m, =C**H**CH_2OH), 5.56-5.49 (1H, m, NCH_2C**H**=), 5.00–4.99 (1H, m, C**H**=C$(CH_3)_2$), 4.24 (2H, d, *J* = 1.9 Hz, ≡C$\mathbf{H}_2$N) 4.20 (2H, dd, *J* = 6.8, 1.3 Hz, =CHC$\mathbf{H}_2$OH), 3.90 (2H, dd, *J* = 7.4,

1.3 Hz, NC**H**$_2$CH=), 2.41 (3H, s, ArC**H**$_3$), 1.79 (1H, br s, O**H**), 1.74 (3H, s, CH=C(C**H**$_3$)$_2$), 1.64 (3H, s, CH=C(C**H**$_3$)$_2$); ^{13}C NMR (100.6 MHz, CDCl$_3$) δ 149.3 (C), 143.5 (C), 135.7 (C), 134.0 (CH), 129.4 (2 × CH), 127.7 (2 × CH), 125.7 (CH), 104.0 (CH), 83.8 (C), 83.5 (C), 57.9 (CH$_2$), 42.9 (CH$_2$), 37.0 (CH$_2$), 24.6 (CH$_3$), 21.4 (CH$_3$), 20.8 (CH$_3$); HRMS (ESI) Exact mass calculated for $[C_{18}H_{23}NNaO_3S]^+$ $[M+Na]^+$: 356.1291, found: 356.1288.

General Procedure B: Phosphorylation of Allylic Alcohols

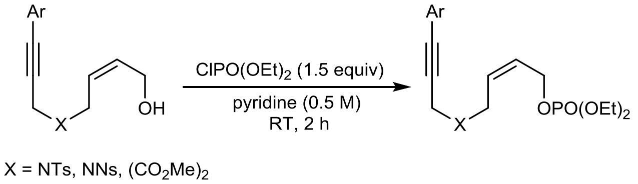

Diethyl chlorophosphate (1.0 to 1.5 equiv) was added dropwise to a solution of the appropriate alcohol (1.0 equiv) in anhydrous pyridine (approximately 0.5 M) at 0 °C. After 10 min, the ice bath was removed and the mixture stirred for the indicated time (2 to 22 h) at room temperature. The reaction was then poured into 10% aqueous $CuSO_4$ solution (14 × pyridine volume) and extracted with EtOAc (2 × $CuSO_4$ volume). The combined organic layers were washed with saturated aqueous $NaHCO_3$ solution (0.25 × organic volume), brine (0.25 × organic volume), dried ($MgSO_4$), filtered and concentrated *in vacuo*. Purification by flash column chromatography (EtOAc:petroleum ether) afforded the allylic phosphate.

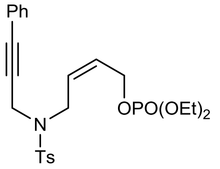

(*Z*)-(4-{[4-Methyl-*N*-(3-phenylprop-2-yn-1-yl)phenyl]sulfonamido}but-2-en-1-yl) diethyl phosphate (1a). Prepared according to General Procedure B using enyne **S6**[6] (451 mg, 1.27 mmol), diethyl chlorophosphate (274 μL, 1.90 mmol) and anhydrous pyridine (3 mL), with a reaction time of 19 h. Purification by flash column chromatography (50 to 65% EtOAc:petroleum ether) gave *allylic phosphate* **1a** (419 mg, 67%) as a pale yellow oil. R$_f$ = 0.53 (EtOAc); IR 2981, 2927, 1598, 1511, 1491, 1443, 1396, 1344, 1305, 1249, 1160, 1091, 966, 922, 896, 833, 814, 802 cm^1; ^{1}H NMR (400 MHz, CDCl$_3$) δ 7.80–7.77 (2H, m, Ar**H**), 7.32–7.23 (5H, m, Ar**H**), 7.12–7.09 (2H, m, Ar**H**), 5.90 (1H, dtt, *J* = 11.0, 6.7, 1.4 Hz, =C**H**CH$_2$O), 5.70 (1H, dtt, *J* = 11.0, 7.3, 1.4 Hz, NCH$_2$C**H**=), 4.67 (2H, ddd, 8.4, 6.7, 1.4 Hz, =C**H**CH$_2$O), 4.31 (2H, s, ≡CC**H**$_2$N), 4.10–4.01 (4H, m, 2 × OC**H**$_2$CH$_3$), 3.98 (2H, br d, *J* = 7.3 Hz, NC**H**$_2$CH=), 2.35 (3H, s, ArC**H**$_3$), 1.28 (6H, td, *J* = 7.1, 1.0 Hz, 2 × OCH$_2$C**H**$_3$); ^{13}C NMR (100.6 MHz, CDCl$_3$) δ 143.6 (C), 135.5 (C), 131.4 (2 × CH), 129.8 (d, $^3J_{C\text{-}P}$ = 6.8 Hz, CH), 129.5 (2 × CH), 128.4 (CH), 128.1 (2 × CH), 127.9 (CH), 127.7 (2 × CH), 121.8 (C), 85.8 (C), 81.3 (C), 63.8 (d, $^2J_{C\text{-}P}$ = 5.9 Hz, 2 × CH$_3$), 62.4 (d, $^2J_{C\text{-}P}$ = 5.3 Hz, CH$_2$), 43.2 (CH$_2$), 36.9 (CH$_2$), 21.3 (CH$_3$), 16.0 (d,

$^{3}J_{C\text{-}P}$ = 6.7 Hz, 2 × CH_3); HRMS (ESI) Exact mass calculated for $[C_{24}H_{31}NO_6PS]^+$ $[M+H]^+$: 492.1604, found: 492.1606.

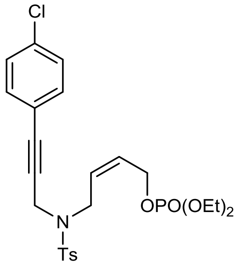

(*Z*)-4-({*N*-[3-(4-chlorophenyl)prop-2-yn-1-yl]-4-methylphenyl}sulfonamido)but-2-en-1-yl diethyl phosphate (1b). Prepared according to General Procedure B using enyne **S7**[7] (1.45 g, 3.71 mmol), diethyl chlorophosphate (609 μL, 4.22 mmol) and anhydrous pyridine (7 mL), with a reaction time of 22 h. Purification by flash column chromatography (50 to 60% EtOAc:petroleum ether) gave *allylic phosphate* **1b** (1.46 g, 75%) as an off-white solid. R_f = 0.42 (80% EtOAc:petroleum ether); m.p. 37–38 °C (Et_2O); IR 2986, 2909, 1597, 1489, 1340, 1321, 1266, 1248, 1158, 1122, 1089, 1061, 1015, 970, 956, 942, 898, 812 cm^{-1}; ^{1}H NMR (400 MHz, $CDCl_3$) δ 7.83–7.69 (2H, m, Ar**H**), 7.29–7.24 (2H, m, Ar**H**), 7.24–7.19 (2H, m, Ar**H**), 7.07–7.00 (2H, m, Ar**H**), 5.88 (1H, dtt, *J* = 11.0, 6.7, 1.4 Hz, =C**H**CH_2O), 5.67 (1H, dtt, *J* = 11.0, 7.3, 1.6 Hz, NCH_2C**H**=), 4.64 (2H, ddd, *J* = 8.7, 6.7, 1.4 Hz, =CHC$\mathbf{H}_2$O), 4.28 (2H, s, ≡CC$\mathbf{H}_2$N), 4.09–4.00 (4H, m, 2 × OC$\mathbf{H}_2$$CH_3$), 3.95 (2H, br d, *J* = 7.3 Hz, NC$\mathbf{H}_2$CH=), 2.35 (3H, s, ArC$\mathbf{H}_3$), 1.27 (6H, td, *J* = 7.1, 1.0 Hz, 2 × OCH_2C$\mathbf{H}_3$); ^{13}C NMR (100.6 MHz, $CDCl_3$) δ 143.7 (C), 135.6 (C), 134.6 (C), 132.7 (2 × CH), 129.9 (d, $^{3}J_{C\text{-}P}$ = 6.8 Hz, CH), 129.6 (2 × CH), 128.5 (2 × CH), 127.9 (CH), 127.8 (2 × CH), 120.4 (C), 84.7 (C), 82.5 (C), 63.8 (d, $^{2}J_{C\text{-}P}$ = 5.9 Hz, 2 × CH_2), 62.3 (d, $^{2}J_{C\text{-}P}$ = 5.4 Hz, CH_2), 43.3 (CH_2), 36.9 (CH_2), 21.4 (CH_3), 16.0 (d, $^{3}J_{C\text{-}P}$ = 6.6 Hz, 2 × CH_3); HRMS (ESI) Exact mass calculated for $[C_{24}H_{30}ClNO_6PS]^+$ $[M+H]^+$: 526.1214, found: 526.1211.

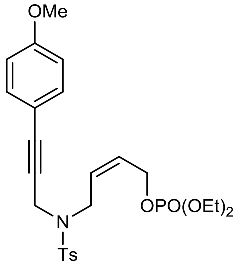

(*Z*)-[4-({*N*-[3-(4-methoxyphenyl)prop-2-yn-1-yl]-4-methylphenyl}sulfonamido)but-2-en-1-yl] diethyl phosphate (1c). Prepared according to General Procedure B using enyne **S8**[8] (1.43 g, 3.71 mmol), diethyl chlorophosphate (609 μL, 4.22 mmol) and anhydrous pyridine (7 mL), with a reaction time of 22 h. Purification by flash column chromatography (50 to 70% EtOAc:petroleum ether) gave *allylic phosphate* **1c** (1.55 g, 80%) as a pale yellow oil. R_f = 0.35 (80% EtOAc:petroleum ether); IR 2981, 2910, 1605, 1568, 1509, 1442, 1345, 1290, 1247, 1159, 1107, 1090, 1019, 968, 896, 834, 815, 800 cm^{-1}; ^{1}H NMR (400 MHz, $CDCl_3$) δ 7.82–7.73 (2H, m, Ar**H**), 7.35–7.24 (2H, m, Ar**H**), 7.09–7.00 (2H, m, Ar**H**), 6.83–6.72 (2H, m, Ar**H**), 5.87 (1H, dtt, *J* = 11.0, 6.7, 1.5 Hz, =C**H**CH_2O), 5.67 (1H, dtt, *J* = 11.0, 7.2, 1.4 Hz, NCH_2C**H**=), 4.66 (2H, ddd, *J* = 8.7, 6.7, 1.5 Hz, =CHC$\mathbf{H}_2$O), 4.28 (2H, s, ≡CC$\mathbf{H}_2$N), 4.09-4.00 (4H, m, 2 × OC$\mathbf{H}_2$$CH_3$), 3.95 (2H, br d, *J* = 7.3 Hz, NC$\mathbf{H}_2$CH=), 3.79 (3H, s, OC$\mathbf{H}_3$), 2.36 (3H, s, ArC$\mathbf{H}_3$), 1.27 (6H, td, *J* = 7.1, 1.0 Hz, 2 × OCH_2C$\mathbf{H}_3$); ^{13}C NMR (100.6 MHz, $CDCl_3$) δ 159.7 (C), 143.6 (C), 135.6 (C), 133.0 (2 × CH), 129.8

(d, $^3J_{C\text{-}P}$ = 7.0 Hz, CH), 129.5 (2 × CH), 128.0 (CH), 127.8 (2 × CH), 114.0 (C), 113.7 (2 × CH), 85.8 (C), 79.9 (C), 63.8 (d, $^2J_{C\text{-}P}$ = 5.8 Hz, 2 × CH_2), 62.4 (d, $^2J_{C\text{-}P}$ = 5.7 Hz, CH_2), 55.3 (CH_3), 43.2 (CH_2), 37.0 (CH_2), 21.4 (CH_3), 16.0 (d, $^3J_{C\text{-}P}$ = 6.6 Hz, 2 × CH_3); HRMS (ESI) Exact mass calculated for $[C_{25}H_{33}NO_7PS]^+$ $[M+H]^+$: 522.1710, found: 522.1704.

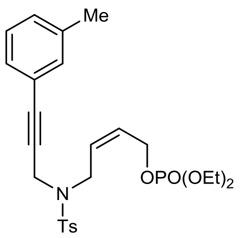

(*Z*)-[4-({4-Methyl-*N*-[3-(*m*-tolyl)prop-2-yn-1-yl]phenyl}sulfonamido)but-2-en-1-yl] diethyl phosphate (1d). Prepared according to General Procedure B using enyne **S9**[9] (279 mg, 0.75 mmol), diethyl chlorophosphate (163 μL, 1.13 mmol) and anhydrous pyridine (2 mL), with a reaction time of 2 h. Purification by flash column chromatography (50 to 70% EtOAc:petroleum ether gave *allylic phosphate* **1d** (252 mg, 66%) as a pale yellow oil. R_f = 0.53 (70% EtOAc:petroleum ether); IR 3030, 1655, 1411, 1345, 1117, 959, 847, 814, 735, 658, 543 cm^{-1}; ^{1}H NMR (500 MHz, $CDCl_3$) δ 7.76-7.69 (2H, m, Ar**H**), 7.26-7.19 (2H, m, Ar**H**), 7.10-7.03 (2H, m, Ar**H**), 6.86-6.82 (2H, m, Ar**H**), 5.88-5.80 (1H, m, =C**H**CH_2O), 5.66-5.59 (1H, m, NCH_2C**H**=), 4.61 (2H, ddd, *J* = 8.5, 6.8, 1.5 Hz, CHC**H**$_2$O), 4.24 (2H, s, ≡CC**H**$_2$N), 4.06-3.94 (4H, m, 2 × OC**H**$_2$$CH_3$), 3.91 (2H, d, *J* = 7.3 Hz, NC**H**$_2$CH=), 2.29 (3H, s, Ar**CH**$_3$), 2.24 (3H, s, ArC**H**$_3$), 1.23-1.18 (6H, m, 2 × OCH_2C**H**$_3$); ^{13}C NMR (126 MHz, $CDCl_3$) δ 143.4 (C), 137.5 (C), 135.3 (C), 131.8 (CH), 129.7 (d, $^2J_{C\text{-}P}$ = 6.5 Hz, CH), 129.4 (2 × CH), 129.2 (CH), 128.4 (CH), 127.8 (CH), 127.63 (CH), 127.58 (2 × CH), 121.5 (C), 85.8 (C), 80.6 (C), 63.6 (d, $^2J_{C\text{-}P}$ = 5.9 Hz, 2 × CH_2), 62.2 (d, $^2J_{C\text{-}P}$ = 5.6 Hz, CH_2), 43.0 (CH_2), 36.7 (CH_2), 21.2 (CH_3), 20.9 (CH_3), 15.8 (d, $^3J_{C\text{-}P}$ = 6.5 Hz, 2 × CH_3); HRMS (ESI) Exact mass calculated for $[C_{25}H_{33}NO_6PS]^+$ $[M+H]^+$: 506.1761, found 506.1746.

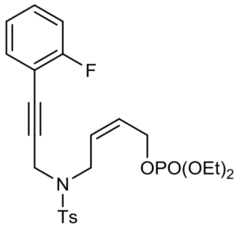

(*Z*)-[4-({*N*-[3-(2-Fluorophenyl)prop-2-yn-1-yl]-4-methylphenyl}sulfonamido)but-2-en-1-yl] diethyl phosphate (1e). Prepared according to General Procedure B using enyne **S13** (529 mg, 1.41 mmol), diethyl chlorophosphate (306 μL, 2.12 mmol) and anhydrous pyridine (3 mL), with a reaction time of 2 h. Purification by flash column chromatography (70% EtOAc:petroleum ether) gave *allylic phosphate* **1e** (606 mg, 84%) as a dark orange oil. R_f = 0.37 (70% EtOAc:petroleum ether); IR 3110, 1596, 1573, 1342, 1307, 1103, 1023, 803, 697, 593 cm^{-1}; ^{1}H NMR (400 MHz, $CDCl_3$) δ 7.76 (2H, d, *J* = 8.3 Hz, Ar**H**), 7.32-7.21 (3H, m, Ar**H**), 7.10 (1H, td, *J* = 7.3, 1.9 Hz, Ar**H**), 7.06-6.98 (2H, m, Ar**H**), 5.89 (1H, dtt, *J* = 11.0, 6.7, 1.4 Hz, =C**H**CH_2O), 5.68 (1H, dtt, *J* = 11.0, 7.2, 1.3 Hz, NCH_2C**H**=), 4.66 (2H, ddd, *J* = 8.9, 6.6, 1.5 Hz, =CHC**H**$_2$O), 4.34 (2H, s, ≡CC**H**$_2$N), 4.10-4.01 (4H, m, 2 × OC**H**$_2$$CH_3$), 3.97 (2H, dd, *J* = 7.2, 1.6 Hz, NC**H**$_2$CH=), 2.28 (3H, s, ArC**H**$_3$), 1.27 (6H, td, *J* = 7.1, 1.0 Hz, 2 × OCH_2C**H**$_3$); ^{13}C NMR (100.6 MHz, $CDCl_3$) δ 162.6

(d, $^{1}J_{C-F}$ = 252.3 Hz, C), 143.7 (C), 135.5 (C), 133.3 (CH), 130.3 (d, $^{3}J_{C-F}$ = 8.1 Hz, CH), 130.1 (d, $^{3}J_{C-P}$ = 6.5 Hz, CH), 129.6 (2 × CH), 127.81 (CH), 127.75 (2 × CH) 123.8 (d, $^{4}J_{C-F}$ = 3.8 Hz, CH), 115.3 (d, $^{2}J_{C-F}$ = 21.1 Hz, CH), 110.5 (d, $^{2}J_{C-F}$ = 15.9 Hz, C), 86.8 (d, $^{3}J_{C-F}$ = 3.6 Hz, C), 79.3 (C), 63.8 (d, $^{2}J_{C-P}$ = 5.9 Hz, 2 × CH_2), 62.4 (d, $^{2}J_{C-P}$ = 5.5 Hz, CH_2), 43.3 (CH_2), 37.0 (CH_2), 21.4 (CH_3), 16.0 (d, $^{3}J_{C-P}$ = 6.7 Hz, 2 × CH_3); HRMS (ESI) Exact mass calculated for $[C_{24}H_{29}FNNaO_6PS]^+$ $[M+Na]^+$: 532.1329, found 532.1337.

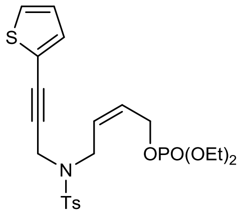

(*Z*)-[4-({4-Methyl-*N*-[3-(thiophen-2-yl)prop-2-yn-1-yl]phenyl}sulfonamido)but-2-en-1-yl] diethyl phosphate (1f). Prepared according to General Procedure B using enyne **S14** (700 mg, 1.52 mmol), diethyl chlorophosphate (348 μL, 2.28 mmol) and anhydous pyridine (3 mL), with a reaction time of 2 h. Purification by flash column chromatography (60 to 70% EtOAc:petroleum ether) gave *allylic phosphate* **1f** (531 mg, 70%) as a dark orange oil. R_f = 0.50 (70% EtOAc:petroleum ether); IR 2982, 1635, 1418, 1342, 1323, 1192, 1159, 1023, 957, 848, 759 cm^{-1} ; ^{1}H NMR (400 MHz, $CDCl_3$) δ 7.81-7.69 (2H, m, Ar**H**), 7.35-7.24 (2H, m, Ar**H**), 7.20 (1H, dd, *J* = 5.2, 1.2 Hz, Ar**H**), 6.98 (1H, d, *J* = 3.6, 1.2 Hz, Ar**H**), 6.90 (1H, dd, *J* = 5.2, 3.6 Hz, Ar**H**), 5.87 (1H, dtt, *J* = 11.0, 6.7, 1.4 Hz, =C**H**CH$_2$O), 5.66 (1H, dtt, *J* = 11.0, 7.3, 1.3 Hz, NC**H**$_2$CH=), 4.72-4.61 (2H, m, =CHC**H**$_2$O), 4.30 (2H, s, ≡CC**H**$_2$N), 4.11-4.01 (4H, m, 2 × OC**H**$_2$CH$_3$), 3.93 (2H, d, *J* = 7.3 Hz, NC**H**$_2$CH=), 2.82 (3H, s, ArC**H**$_3$), 1.28 (6H, td, *J* = 7.1, 1.0 Hz, 2 × OCH$_2$C**H**$_3$); ^{13}C NMR (100.6 MHz, $CDCl_3$) δ 143.8 (C), 135.3 (C), 132.4 (CH), 130.0 (d, $^{2}J_{C-P}$ = 7.0 Hz, CH), 129.7 (2 × CH), 127.8 (CH), 127.7 (2 × CH), 127.3 (CH), 126.8 (CH), 121.7 (C), 85.3 (C), 79.1 (C), 63.8 (d, $^{2}J_{C-P}$ = 5.9 Hz, 2 × CH_2), 62.4 (d, $^{2}J_{C-P}$ = 5.3 Hz, CH_2), 43.3 (CH_2), 37.1 (CH_2), 21.5 (CH_3), 16.0 (d, $^{3}J_{C-P}$ = 6.7 Hz, 2 × CH_3); HRMS (ESI) Exact mass calculated for $[C_{22}H_{28}NNaO_6PS_2]^+$ $[M+Na]^+$: 520.0988, found 520.0988.

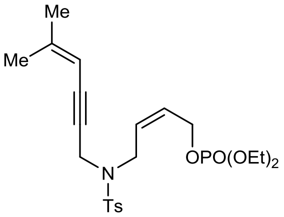

(*Z*)-(4-{[4-Methyl-*N*-(5-methylhex-4-en-2-yn-1-yl)phenyl]sulfonamido}but-2-en-1-yl) diethyl phosphate (1g). Prepared according to General Procedure B using enyne **S16** (670 mg, 2.01 mmol), diethyl chlorophosphate (435 μL, 3.01 mmol) and pyridine (4 mL), with a reaction time of 4 h. Purification by flash column chromatography (10 to 70% EtOAc:petroleum ether) gave *allylic phosphate* **1g** (500 mg, 53%) as a brown oil. R_f = 0.68 (80% EtOAc:petroleum ether); IR 2980, 2910, 1443, 1345, 1262, 1203, 1159, 1090, 1017, 959, 898, 814, 659, 584, 544 cm^{-1}; ^{1}H NMR (400 MHz, $CDCl_3$) δ 7.73 (2H, d, *J* = 8.3 Hz, Ar**H**), 7.30-7.28 (2H, m, Ar**H**), 5.87-5.80 (1H, m, =C**H**CH$_2$O), 5.67-5.61 (1H, m, NCH$_2$C**H**=), 5.01 (1H, s, C**H**=C(CH$_3$)$_2$), 4.65-4.61 (2H, m, =CHC**H**$_2$O), 4.23 (2H, d, *J* = 2.0 Hz, ≡CC**H**$_2$N), 4.14-4.06 (4H, m, 2 × OC**H**$_2$CH$_3$), 3.91-3.88 (2H,

m, NC**H**$_2$CH=), 2.42 (3H, s, ArC**H**$_3$), 1.74 (3H, d, *J* = 0.6 Hz, CH=C(C**H**$_3$)$_2$), 1.65 (3H, s, CH=C(C**H**$_3$)$_2$), 1.35-1.31 (6H, m, 2 × OCH$_2$C**H**$_3$); ^{13}C NMR (100.6 MHz, CDCl$_3$) δ 149.2 (C), 143.5 (C), 135.7 (C), 129.54 (d, $^3J_{C-P}$ = 7.0 Hz, CH), 129.45 (2 × CH), 128.0 (CH), 127.7 (2 × CH), 104.2 (CH), 83.9 (C), 83.2 (C), 63.8 (d, $^2J_{C-P}$ = 5.8 Hz, 2 × CH$_2$), 62.4 (d, $^2J_{C-P}$ = 5.6 Hz, CH$_2$), 43.1 (CH$_2$), 37.2 (CH$_2$), 24.6 (CH$_3$), 21.4 (CH$_3$), 20.8 (CH$_3$), 16.1 (d, $^3J_{C-P}$ = 6.9 Hz, 2 × CH$_3$); HRMS (ESI) Exact mass calculated for [$C_{22}H_{32}NNaO_6PS$]$^+$ [M+Na]$^+$: 492.1580, found: 492.1576.

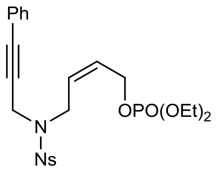

(*Z*)-(4-{[4-Nitro-*N*-(3-phenylprop-2-yn-1-yl)phenyl]sulfonamido}but-2-en-1-yl) diethyl phosphate (1h). Prepared according to General Procedure B using enyne **S15** (500 mg, 1.29 mmol), diethyl chlorophosphate (280 μL, 1.94 mmol) and anhydrous pyridine (3 mL), with a reaction time of 2 h. Purification by flash column chromatography (40 to 60% EtOAc:petroleum ether) gave *allylic phosphate* **1h** (605 mg, 90%) as a pale yellow solid. R$_f$ = 0.48 (70% EtOAc:petroleum ether); m.p. 66-68 °C (Et$_2$O); IR 2978, 1608, 1489, 1343, 1248, 1161, 995, 957, 890, 814, 755, 681, 659, 595 cm^{-1}; ^{1}H NMR (400 MHz, CDCl$_3$) δ 8.35-8.21 (2H, m, Ar**H**), 8.16-8.01 (2H, m, Ar**H**), 7.34-7.26 (1H, m, Ar**H**), 7.25-7.19 (2H, m, Ar**H**), 7.11-6.98 (2H, m, Ar**H**), 5.93 (1H, dtt, *J* = 11.1, 6.7, 1.5 Hz, =C**H**CH$_2$O), 5.69 (1H, dtt, *J* = 11.0, 7.3, 1.2 Hz, NCH$_2$C**H**=), 4.66 (2H, ddd, *J* = 9.0, 6.7, 1.5 Hz, =CHC**H**$_2$O), 4.35 (2H, s, ≡CC**H**-$_2$N), 4.13-3.96 (6H, m, NC**H**$_2$CH= and 2 × OC**H**$_2$CH$_3$), 1.27 (6H, td, *J* = 7.1, 1.0 Hz, 2 × OCH$_2$C**H**$_3$); ^{13}C NMR (100.6 MHz, CDCl$_3$) δ 150.0 (C), 144.5 (C), 131.2 (2 × CH), 130.5 (d, $^3J_{C-P}$= 6.5 Hz, CH), 129.0 (3 × CH), 128.4 (2 × CH), 127.2 (CH), 124.1 (2 × CH), 121.2 (C), 86.5 (C), 80.7 (C), 63.8 (d, $^2J_{C-P}$ = 5.9 Hz, 2 × CH$_2$), 62.1 (d, $^2J_{C-P}$ = 5.7 Hz, CH$_2$), 43.5 (CH$_2$), 37.1 (CH$_2$), 16.0 (d, $^3J_{C-P}$ = 6.6 Hz, 2 × CH$_3$); HRMS (ESI) Exact mass calculated for [$C_{23}H_{27}NaN_2O_8PS$]$^+$ [M+Na]$^+$: 545.1118, found 545.1101.

Preparation of Substrate 1i

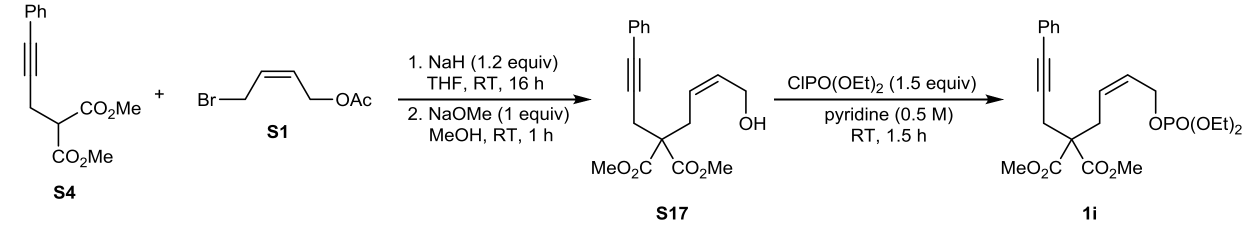

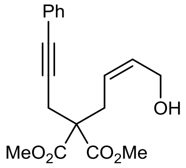

(*Z*)-2-(4-Hydroxybut-2-en-1-yl)-2-(3-phenylprop-2-yn-1-yl) dimethyl malonate (S17). Malonate **S4**[5] (4.61 g, 18.7 mmol) was added in one portion to a stirred suspension of NaH (900 mg, 22.5 mmol, 60% dispersion in mineral oil) in anhydrous THF (100 mL) under argon at 0 °C. The ice bath was then removed and the mixture was stirred at room temperature for 45 min. A solution of (*Z*)-4-bromobut-2-en-1-yl acetate **S1**[2] (3.97 g, 20.6 mmol) in anhydrous THF (14 mL) was then added *via* syringe and the resulting mixture was stirred at room temperature for 18 h. The reaction was then quenched with H_2O, diluted with EtOAc (400 mL), washed with H_2O (100 mL), brine (100 mL), dried ($MgSO_4$), filtered and concentrated *in vacuo* to afford the crude allylic acetate. This material was dissolved in MeOH (20 mL) under argon, NaOMe (1.10 g, 20.4 mmol) added and the mixture stirred at room temperature for 1 h. The mixture was concentrated *in vacuo* and the residue was dissolved in EtOAc (100 mL), washed with H_2O (50 mL), brine (50 mL), dried ($MgSO_4$), filtered and concentrated *in vacuo* to give the crude alcohol. Purification by flash column chromatography (40 to 60% EtOAc:petroleum ether) gave *enyne* **S17** (2.89 g, 69% over 2 steps) as a colorless oil. Spectroscopic data was in accordance with those previously reported.[6]

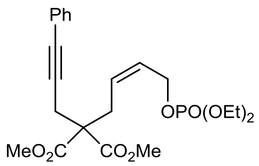

(*Z*)-2-{4-[(Diethoxyphosphoryl)oxy]but-2-en-1-yl}-2-(3-phenylprop-2-yn-1-yl) dimethyl malonate (1i). Prepared according to General Procedure B using enyne **S17** (3.20 g, 10.1 mmol), diethyl chlorophosphate (2.61 g, 15.2 mmol) and anhydrous pyridine (20 mL), with a reaction time of 1.5 h. Purification by flash column chromatography (50% to 65% EtOAc:petroleum ether) gave *allylic phosphate* **1i** (3.70 g, 81%) as a yellow oil. R_f = 0.56 (80% EtOAc:petroleum ether); IR 2984, 1734, 1490, 1437, 1368, 1262, 1202, 1102, 1016, 977, 853, 817, 757, 692, 692, 527 cm^{-1}; ^{1}H NMR (400 MHz, $CDCl_3$) δ 7.39-7.36 (2H, m, Ar**H**), 7.30-7.26 (3H, m, Ar**H**), 5.80 (1H, dtd, *J* = 10.6, 6.8, 1.9 Hz, =C**H**CH_2O), 5.55-5.47 (1H, m, CCH_2C**H**=), 4.69-4.64 (2H, m, =CHC**H**$_2$O), 4.11-4.03 (4H, m, 2 × OC**H**$_2$$CH_3$), 3.77 (3H, s, CO_2C**H**$_3$), 3.77 (3H, s, CO_2C**H**$_3$), 3.02 (2H, d, *J* = 2.1 Hz, ≡CC**H**$_2$C), 2.93 (2H, dt, *J* = 8.1, 1.8 Hz, CC**H**$_2$CH=), 1.31-1.14 (6H, m, 2 × OCH_2C**H**$_3$); ^{13}C NMR (100.6 MHz, $CDCl_3$) δ 170.0 (2 × C), 131.6 (2 × CH), 129.1 (d, $^2J_{C-P}$ = 6.8 Hz, CH), 128.2 (2 × CH), 128.1 (CH), 127.1 (CH), 122.8 (C), 83.8 (2 × C), 63.7 (d, $^2J_{C-P}$ = 5.9 Hz, 2 × CH_2), 62.7 (d, $^2J_{C-P}$ = 5.5 Hz, CH_2), 56.9 (C), 52.9 (2 × CH_3), 30.4 (CH_2), 23.7 (CH_2), 16.0 (d, $^3J_{C-P}$ = 6.6 Hz, 2 × CH_3); HRMS (ESI) Exact mass calculated for $[C_{22}H_{29}NaO_8P]^+$ $[M+Na]^+$: 475.1492, found: 475.1476.

Preparation of Substrate S19

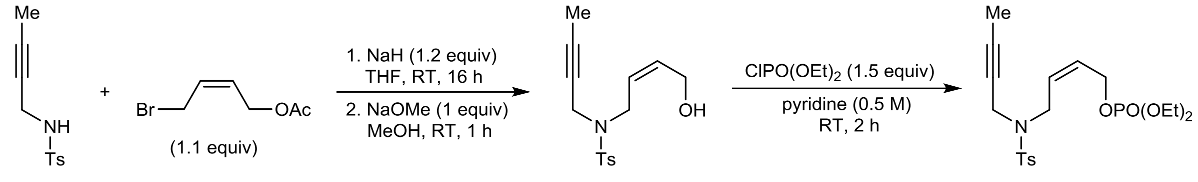

(*Z*)-*N*-(But-2-yn-1-yl)-*N*-(4-hydroxybut-2-en-1-yl)-4-methylbenzenesulfonamide (S18)

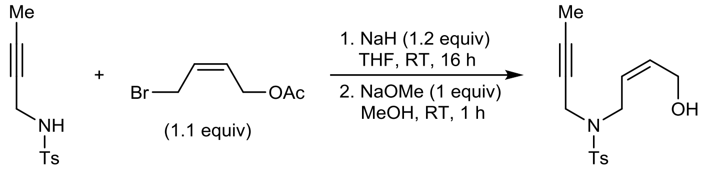

Alkynyl sulfonamide **S3**[4] (1.76 g, 6.92 mmol) was added in one portion to a stirred suspension of NaH (332 mg, 8.30 mmol, 60% dispersion in mineral oil) in anhydrous THF (35 mL) under argon at 0 °C. The ice bath was then removed and the mixture stirred at room temperature for 45 min. A solution of (*Z*)-4-bromobut-2-en-1-yl acetate **S1**[2] (1.46 g, 7.61 mmol) in anhydrous THF (14 mL) was then added *via* syringe and the resulting mixture stirred at room temperature for 18 h. The reaction was then quenched with H_2O, diluted with EtOAc (100 mL), washed with H_2O (50 mL), brine (50 mL), dried ($MgSO_4$), filtered and concentrated *in vacuo* to afford the crude allylic acetate. This material was dissolved in MeOH (10 mL) under argon, NaOMe (372 mg, 6.92 mmol) added and the mixture stirred at room temperature for 1 h. The mixture was concentrated *in vacuo* and the residue dissolved in EtOAc (50 mL), washed with H_2O (25 mL), brine (25 mL), dried ($MgSO_4$), filtered and concentrated *in vacuo* to give the crude alcohol. Purification by flash column chromatography (30 to 50% EtOAc:petroleum ether) gave *enyne* **S18** (1.52 g, 69% over 2 steps) as a pale yellow oil. R_f = 0.34 (50% EtOAc:petroleum ether); IR 3404 (OH), 2920, 1597, 1494, 1436, 1341, 1261, 1017, 947, 896, 814 cm^{-1}; ^1H NMR (400 MHz, $CDCl_3$) δ 7.82-7.66 (2H, m, Ar**H**), 7.39-7.27 (2H, m, Ar**H**), 5.91-5.83 (1H, m, =C**H**CH_2O), 5.54-5.46 (1H, m, NCH_2C**H**=), 4.20 (2H, d, *J* = 6.8 Hz, CHC**H**$_2$O), 4.02 (2H, q, *J* = 2.4 Hz, ≡CC**H**$_2$N), 3.87 (2H, d, *J* = 7.5 Hz, NC**H**$_2$CH=), 2.43 (3H, s, ArC**H**$_3$), 1.99-1.83 (1H, br s, O**H**), 1.55 (3H, t, *J* = 2.4 Hz, C**H**$_3$C=); ^{13}C NMR (100.6 MHz, $CDCl_3$) δ 143.4 (C), 135.7 (C), 133.9 (CH), 129.3 (2 × CH), 127.8 (2 × CH), 125.8 (CH), 81.8 (C), 71.7 (C), 57.9 (CH_2), 42.8 (CH_2), 36.5 (CH_2), 21.5 (CH_3), 3.2 (CH_3); HRMS (ESI) Exact mass calculated for $[C_{15}H_{19}NNaO_3S]^+$ $[M+Na]^+$: 316.0978, found 316.0968.

(*Z*)-4-{[*N*-(But-2-yn-1-yl)-4-methylphenyl]sulfonamide}but-2-en-1-yl diethyl phosphate (S19)

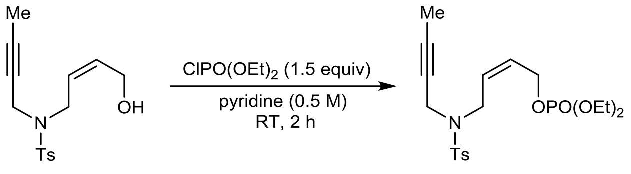

Prepared according to General Procedure B using enyne **S18** (500 mg, 1.70 mmol), diethyl chlorophosphate (369 μL, 2.56 mmol) and anhydrous pyridine (4 mL), with a reaction time of 2 h. Purification by flash column chromatography (60 to 70% EtOAc:petroleum ether) gave *allylic phosphate* **S19** (442 mg, 60%) as a pale yellow oil. R_f = 0.25 (50% EtOAc:petroleum ether; IR 2983, 1598, 1443, 1394, 1261, 1091, 960, 898, 802, 758, 656 cm^{-1}; ^1H NMR (400 MHz, $CDCl_3$) δ 7.76-7.64 (2H, m, Ar**H**), 7.37-7.26 (2H, m, Ar**H**), 5.80 (1H, dtt, *J* = 11.0, 6.6, 1.4 Hz, =C**H**CH_2O), 5.58 (1H, dtt, *J* = 11.0, 7.3, 1.4 Hz, NCH_2C**H**=), 4.61 (2H, ddd, *J* = 8.2, 6.6, 1.5 Hz, =CHC**H**$_2$O), 4.15-4.02 (4H, m, 2 × OC**H**$_2$$CH_3$), 3.98 (2H, q, *J* = 2.4 Hz, ≡CC**H**$_2$N), 3.91-3.77 (2H, m, NC**H**$_2$CH=), 2.41 (3H, s, ArC**H**$_3$), 1.54 (3H, t, *J* = 2.4 Hz, C**H**$_3$C≡), 1.31 (6H, td, *J* = 7.1, 1.0 Hz, 2 × OC**H**$_2$$CH_3$); ^{13}C NMR (100.6 MHz, $CDCl_3$) δ 140.4 (C), 135.6 (C), 129.5 (d, $^2J_{C\text{-}P}$ = 7.1 Hz, CH), 129.3 (2 × CH), 128.0 (CH), 127.8 (2 × CH), 82.0 (C), 71.3 (C), 63.8 (d, $^2J_{C\text{-}P}$ = 5.9 Hz, 2 × CH_2), 62.4 (d, $^2J_{C\text{-}P}$ = 5.3 Hz, CH_2), 43.0 (CH_2), 36.6 (CH_2), 21.4 (CH_3), 16.0 (d, $^3J_{C\text{-}P}$ = 6.6 Hz, 2 × CH_3), 3.1 (CH_3); HRMS (ESI) Exact mass calculated for $[C_{19}H_{28}NNaO_6PS]^+$ $[M+Na]^+$: 452.1267, found 452.1264.

Preparation of Substrate 1j

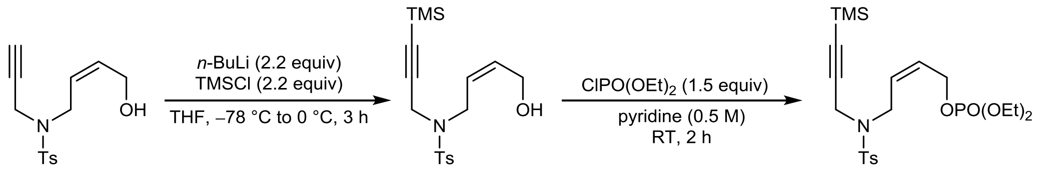

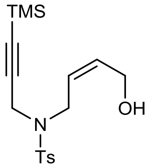

(*Z*)-*N*-(4-Hydroxybut-2-en-1-yl)-4-methyl-*N*-[3-(trimethylsilyl)prop-2-yn-1-yl]benzenesulfonamide (S20). *n*-BuLi (665 μL, 2.38 M in hexanes, 3.94 mmol) was added to a solution of enyne **S5**[6] (500 mg, 1.79 mmol) in THF (6 mL) at –78 °C, and the resulting mixture was stirred at this temperature for 2 h. Chlorotrimethylsilane (499 μL, 3.94 mmol) was then added, and the mixture warmed slowly to 0 °C. After stirring for 30 min at 0 °C, the mixture was diluted with water (10 mL) and extracted with EtOAc (3 × 10 mL). The combined organic layers were washed with saturated aqueous $NaHCO_3$ solution (10 mL), brine (10 mL), dried ($MgSO_4$), filtered and concentrated *in vacuo* to afford the crude material. Purification by

flash column chromatography (30 to 50% EtOAc:petroleum ether) gave *enyne* **S20** (314 mg, 50%) as a colorless oil. R_f = 0.50 (60% EtOAc:petroleum ether); IR 3304 (OH), 3027, 2960, 1598, 1494, 1340, 1320, 1289, 1161, 994, 841, 761, 703, 643, 589, 544 cm^{-1}; ^1H NMR (400 MHz, $CDCl_3$) δ 7.72 (2H, d, *J* = 8.4 Hz, Ar**H**), 7.31-7.29 (2H, m, Ar**H**), 5.88 (1H, dtt, *J* = 11.1, 6.9, 1.4 Hz, =C**H**CH_2O), 5.49 (1H, dtt, *J* = 11.1, 7.5, 1.4 Hz, NCH_2C**H**), 4.21-4.18 (2H, m, =CHC$\mathbf{H}_2$O), 4.09 (2H, s, ≡CC$\mathbf{H}_2$N), 3.90 (2H, dd, *J* = 7.5, 1.4 Hz, NC$\mathbf{H}_2$CH=), 2.42 (3H, s, ArC$\mathbf{H}_3$), 1.82 (1H, s, O**H**), 0.01 (9H, s, Si(C$\mathbf{H}_3$)$_3$); ^{13}C NMR (100.6 MHz, $CDCl_3$) δ 143.5 (C), 135.6 (C), 134.4 (CH), 129.5 (2 × CH), 127.7 (2 × CH), 125.4 (CH), 97.9 (C), 91.2 (C), 57.8 (CH_2), 42.8 (CH_2), 36.8 (CH_2), 21.5 (CH_3), –0.6 (3 × CH_3); HRMS (ESI) Exact mass calculated for $[C_{17}H_{25}NNaO_3SSi]^+$ $[M+Na]^+$: 374.1217, found: 374.1213.

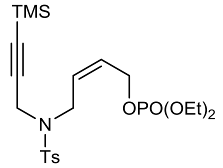

(*Z*)-(4-{[4-Methyl-*N*-(3-(trimethylsilyl)prop-2-yn-1-yl)phenyl]sulfonamido}but-2-en-1-yl) diethyl phosphate (1j). Prepared according to General Procedure B using enyne **S20** (700 mg, 1.99 mmol), diethyl chlorophosphate (430 μL, 2.99 mmol) and pyridine (4 mL) for 2 h. Purification by flash column chromatography (60% EtOAc:petroleum ether) gave *allylic phosphate* **1j** (514 mg, 53%) as a white solid. R_f = 0.40 (60% EtOAc:petroleum ether); m.p. 61–62 °C (Et_2O); IR 2988, 2907, 1475, 1443, 1393, 1324, 1305, 1248, 1105, 1089, 986, 895, 779, 722, 637, 608, 519, 490 cm^{-1}; ^1H NMR (400 MHz, $CDCl_3$) δ 7.74 (2H, d, *J* = 8.4 Hz, Ar**H**), 7.32-7.30 (2H, m, Ar**H**), 5.89-5.82 (1H, m, =C**H**CH_2O), 5.66-5.59 (1H, m, NCH_2C**H**=), 4.67-4.63 (2H, m, =CHC$\mathbf{H}_2$O), 4.15-4.07 (6H, m, 2 × OC$\mathbf{H}_2$$CH_3$ and ≡CC$\mathbf{H}_2$N), 3.94-3.85 (2H, m, NC$\mathbf{H}_2$CH=), 2.44 (3H, s, ArC$\mathbf{H}_3$), 1.35-1.32 (6H, m, 2 × OCH_2C$\mathbf{H}_3$), 0.01 (9H, s, Si(C$\mathbf{H}_3$)$_3$); ^{13}C NMR (100.6 MHz, $CDCl_3$) δ 143.5 (C), 135.6 (C), 129.9 (d, $^3J_{C\text{-}P}$ = 6.1 Hz, CH), 129.5 (2 × CH), 127.8 (CH), 127.7 (2 × CH), 97.5 (C), 91.2 (C), 63.7 (d, $^2J_{C\text{-}P}$ = 6.1 Hz, 2 × CH_2), 62.4 (d, $^2J_{C\text{-}P}$ = 5.3 Hz, CH_2), 43.0 (CH_2), 37.0 (CH_2), 21.5 (CH_3), 16.1 (d, $^3J_{C\text{-}P}$ = 6.7 Hz, 2 × CH_3), –0.6 (3 × CH_3); HRMS (ESI) Exact mass calculated for $[C_{21}H_{34}NNaO_6PSSi]^+$ $[M+Na]^+$: 510.1506, found: 510.1509.

Preparation of Substrate 4

(*E*)-(4-{[4-Methyl-*N*-(3-phenylprop-2-yn-1-yl)phenyl]sulfonamide}but-2-en-1-yl) diethyl phosphate (4)

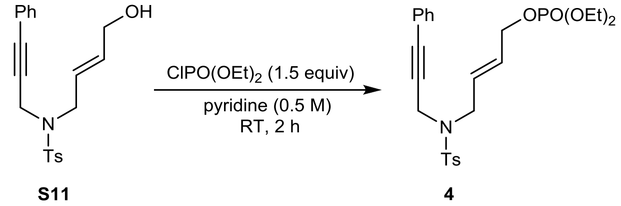

Prepared according to General Procedure B using enyne **S11**[7] (280 mg, 0.79 mmol), diethyl chlorophosphate (130 μL, 15.2 mmol) and anhydrous pyridine (2.5 mL), with a reaction time of 2 h. Purification by flash column chromatography (50% to 85% EtOAc:petroleum ether) gave *allylic phosphate* **4** (330 g, 85%) as a yellow oil. R_f = 0.29 (60% EtOAc:petroleum ether); IR 2980, 2322, 2118, 1990, 1596, 1346, 1259, 1184, 896, 756, 691 cm^{-1}; ^{1}H NMR (400 MHz, $CDCl_3$) δ 7.81-7.68 (2H, m, Ar**H**), 7.32-7.19 (5H, m, Ar**H**), 7.10-6.97 (2H, m, Ar**H**), 5.88 (1H, dt, *J* = 15.6, 5.8 Hz, =C**H**CH_2O), 5.78 (1H, dt, *J* = 15.4, 6.3 Hz, NCH_2C**H**=), 4.52 (2H, ddd, *J* = 8.3, 5.4, 1.2 Hz, =CHC**H**$_2$O), 4.28 (2H, s, ≡CC**H**$_2$N), 4.16-4.02 (4H, m, 2 × OC**H**$_2$$CH_3$), 3.89 (2H, d, *J* = 5.0 Hz, NC**H**$_2$CH=), 2.31 (3H, s, ArC**H**$_3$), 1.37-1.22 (6H, m, 2 × OCH_2C**H**$_3$); ^{13}C NMR (100.6 MHz, $CDCl_3$) δ 143.6 (C), 135.8 (C), 131.4 (2 × CH), 129.7 (d, $^2J_{C-P}$ = 6.7 Hz, CH), 129.5 (2 × CH), 128.4 (CH), 128.1 (2 × CH), 127.9 (CH), 127.8 (2 × CH), 122.0 (C), 85.8 (C), 81.4 (C), 66.6 (d, $^2J_{C-P}$ = 5.5 Hz, CH_2), 63.8 (d, $^2J_{C-P}$ = 5.8 Hz, 2 × CH_2), 47.8 (CH_2), 36.9 (CH_2), 21.4 (CH_3), 16.1 (d, $^3J_{C-P}$ = 6.6 Hz, 2 × CH_3); HRMS (ESI) Exact mass calculated for $[C_{24}H_{30}NNaO_6PS]^+$ $[M+Na]^+$: 514.1424, found: 514.1414.

Preparation of Substrate 6

(*Z*)-(7-Phenylhept-2-en-6-yn-1-yl) diethyl phosphate (6)

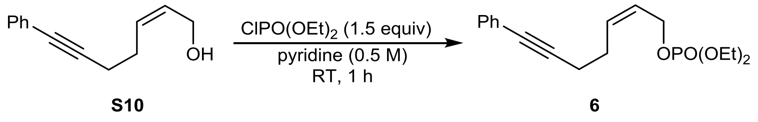

Prepared according to General Procedure B using enyne **S10**[10] (899 mg, 4.83 mmol), diethyl chlorophosphate (1.05 mL, 7.25 mmol) and anhydrous pyridine (5.4 mL) for 1 h. Purification by flash column chromatography (50% to 65% EtOAc:petroleum ether) afforded *allylic phosphate* **6** as a pale brown syrup (1.14 g, 73%). R_f = 0.20 (50% EtOAc:petroleum ether); IR 2981, 2907, 1490, 1442, 1330, 1262, 1100, 1020, 973, 871, 846, 801, 692, 526 cm^{-1}; ^{1}H NMR (400 MHz, $CDCl_3$) δ 7.37-7.34

(2H, m, Ar**H**), 7.28-7.23 (3H, m, Ar**H**), 5.79-5.66 (2H, m, C**H**=C**H**), 4.63 (2H, ddd, J = 8.4, 6.3, 0.9 Hz, =CHC**H**$_2$O), 4.12-4.04 (4H, m, 2 × OC**H**$_2$CH$_3$), 2.49-2.46 (2H, m, ≡CC**H**$_2$CH$_2$), 2.42-2.36 (2H, m, ≡CCH$_2$C**H**$_2$), 1.32-1.27 (6H, m, 2 × OCH$_2$C**H**$_3$); ^{13}C NMR (100.6 MHz, CDCl$_3$) δ 132.8 (CH), 131.4 (2 × CH), 128.1 (2 × CH), 127.6 (CH), 125.6 (d, $^2J_{C\text{-}P}$ = 6.6 Hz, CH), 123.5 (C), 88.8 (C), 81.1 (C), 63.6 (d, $^2J_{C\text{-}P}$ = 5.8 Hz, 2 × CH$_2$), 62.8 (d, $^2J_{C\text{-}P}$ = 5.3 Hz, CH$_2$), 26.7 (CH$_2$), 19.4 (CH$_2$), 16.0 (d, $^3J_{C\text{-}P}$ = 7.0 Hz, CH$_3$); HRMS (ESI) Exact mass calculated for $[C_{17}H_{23}NaO_4P]^+$ $[M+Na]^+$: 345.1226, found: 345.1222.

Enantioselective Nickel-Catalyzed Intramolecular Allylic Substitution: General Procedure C:

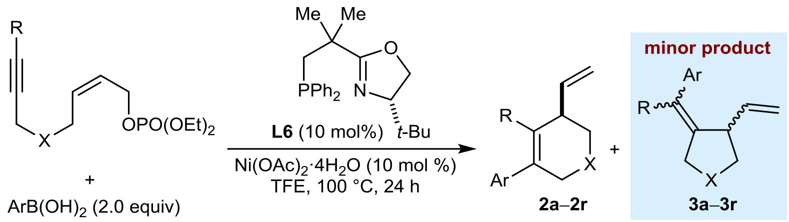

The appropriate substrate (0.30 mmol), arylboronic acid (0.60 mmol), Ni(OAc)$_2$·4H$_2$O (7.5 mg, 0.03 mmol) and (*S*)-*t*-Bu-NeoPHOX (**L6**, 11.0 mg, 0.03 mmol) were added to a microwave vial containing a magnetic stirrer bar. The vial was then sealed with a septum cap and the contents evacuated and charged with argon (3 cycles). 2,2,2-Trifluoroethanol (3 mL) which had been freshly degassed (using 5 freeze-pump-thaw cycles) was then added under argon flow, the septum re-sealed with a layer of vacuum grease, and the contents stirred at 100 °C for 24 h. The reaction was cooled to room temperature, diluted with EtOAc (50 mL) and washed with 50% brine (50 mL). The organic layer was dried (MgSO$_4$) and concentrated *in vacuo* to give the crude mixture. Purification by flash column chromatography (EtOAc:petroleum ether) afforded the title compounds. Unless stated otherwise, the minor isomers **3** were not evident in the isolated products **2**.

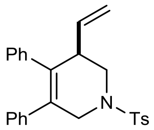

(*R*)-3-Ethenyl-1-(4-methylbenzenesulfonyl)-4,5-diphenyl-1,2,3,6-tetrahydropyridine (2a). Prepared according to General Procedure C using allylic phosphate **1a** (147 mg, 0.30 mmol) and phenylboronic acid (73.2 mg, 0.60 mmol). ^{1}H NMR analysis of the crude material showed the ratio of **2a**:**3a** was >19:1. Purification by flash column chromatography (10% EtOAc:petroleum ether) gave an off-white solid (81 mg, 65%). R$_f$ = 0.33 (30% EtOAc:petroleum ether); m.p. 137–138 °C (Et$_2$O); IR 3078, 3055, 3020, 2977, 2924, 2854, 2804, 1598, 1491, 1454, 1444, 1415, 1354, 1339, 1305, 1227, 1160, 1093, 992, 921 cm^{-1}; $[\alpha]_D^{24}$ –289.0 (*c* 0.66, CHCl$_3$); ^{1}H NMR (400 MHz, CDCl$_3$) δ 7.73-7.70 (2H, m, Ar**H**), 7.36-7.32 (2H, m, Ar**H**), 7.15-7.03 (6H, m, Ar**H**), 7.01-6.96 (2H, m, Ar**H**), 6.90-6.87 (2H, m, Ar**H**), 5.85 (1H, ddd, J = 17.1, 10.3,

7.8 Hz, C**H**=CH_2), 5.04-4.93 (2H, m, CH=C**H_2**), 4.35 (1H, d, *J* = 16.2 Hz, NC**H_a**H_bC=C), 3.75 (1H, ddd, *J* = 11.4, 3.2, 1.0 Hz, NC**H_a**H_bCH), 3.43 (1H, dd, *J* = 16.2, 2.0 Hz, NCH_a**H_b**C=C), 3.43-3.38 (1H, m, C**H**CH=CH_2), 3.05 (1H, dd, *J* = 11.4, 4.0 Hz, NCH_a**H_b**CH), 2.44 (3H, s, ArC**H_3**); ^{13}C NMR (100.6 MHz, $CDCl_3$) δ 143.7 (C), 139.9 (C), 139.0 (C), 136.8 (CH), 135.7 (C), 132.9 (C), 131.8 (C), 129.7 (2 × CH), 129.4 (2 × CH), 129.3 (2 × CH), 128.0 (2 × CH), 127.8 (2 × CH), 127.7 (2 × CH), 127.0 (CH), 126.5 (CH), 117.0 (CH_2), 49.3 (CH_2), 48.0 (CH_2), 44.8 (CH), 21.5 (CH_3); HRMS (ESI) Exact mass calculated for $[C_{26}H_{25}NNaO_2S]^+$ $[M+Na]^+$: 438.1498, found: 438.1502. Enantiomeric excess was determined by HPLC using a Chiralcel OD-H column (98:2 *iso*-hexane:*i*-PrOH, 1.0 mL/min, 230 nm, 25 °C); t_r (major) = 15.6 min, t_r (minor) = 17.6 min, 97% ee.

Slow diffusion of dichloromethane into a solution of **2a** in petroleum ether gave crystals of **2a** that were suitable for X-ray crystallography:

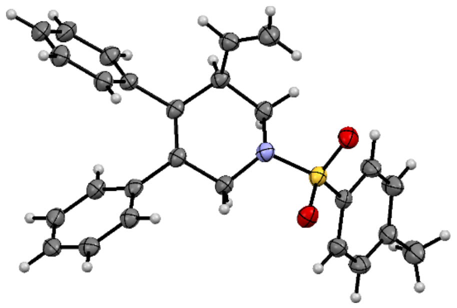

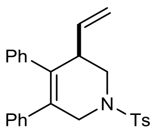

2a
CCDC 1539326
Flack parameter = 0.002(7)

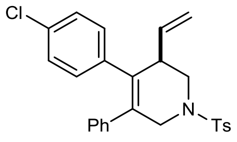

(*R*)-4-(4-Chlorophenyl)-3-ethenyl-1-(4-methylbenzenesulfonyl)-5-phenyl-1,2,3,6-tetrahydropyridine (2b). Prepared according to General Procedure C using allylic phosphate **1b** (157 mg, 0.30 mmol) and phenylboronic acid (73.2 mg, 0.60 mmol). ^{1}H NMR analysis of the crude material showed the ratio of **2b**:**3b** was >19:1. Purification by flash column chromatography (10 to 20% EtOAc:petroleum ether) gave an off-white solid (91 mg, 67%). R_f = 0.33 (30% EtOAc:petroleum ether); m.p. 71–73 °C (Et_2O); IR 3058, 2920, 2851, 1597, 1490, 1455, 1443, 1397, 1376, 1341, 1305, 1232, 1162, 1089, 1060, 1014, 994, 965, 921, 830, 815 cm^{-1}; $[\alpha]_D^{24}$ –271.1 (*c* 0.70, $CHCl_3$); ^{1}H NMR (400 MHz, $CDCl_3$) δ 7.79-7.68 (2H, m, Ar**H**), 7.38-7.34 (2H, m, Ar**H**), 7.19-7.15 (3H, m, Ar**H**), 7.09-7.06 (2H, m, Ar**H**), 7.01-6.97 (2H, m, Ar**H**), 6.85-6.82 (2H, m, Ar**H**), 5.85 (1H, ddd, *J* = 17.1, 10.2, 7.9 Hz, C**H**=CH_2), 5.04 (1H, ddd, *J* = 10.2, 1.4, 0.7 Hz, CH=C**H_a**H_b), 4.98 (1H, ddd, *J* = 17.1, 1.4, 1.0 Hz, CH=CH_a**H_b**); 4.35 (1H, d, *J* = 16.5 Hz, NC**H_a**H_bC=C), 3.74 (1H, ddd, *J* = 11.5, 3.3, 0.9 Hz, NC**H_a**H_bCH), 3.45 (1H, dd, *J* = 16.5, 2.2 Hz, NCH_a**H_b**C=C), 3.38-3.32 (1H, m, C**H**CH=CH_2), 3.06 (1H, dd, *J* = 11.5, 4.1 Hz, NCH_a**H_b**CH), 2.46 (3H, s, ArC**H_3**); ^{13}C NMR (100.6 MHz, $CDCl_3$) δ 143.7 (C), 138.6 (C), 138.3 (C), 136.6 (CH), 134.6 (C), 132.9 (C), 132.6 (C), 132.3 (C), 130.7 (2 × CH), 129.7 (2 × CH), 192.2 (2 × CH), 128.2 (2 ×

CH), 128.0 (2 × CH), 127.8 (2 × CH), 127.3 (CH), 117.3 (CH_2), 49.4 (CH_2), 48.0 (CH_2), 44.8 (CH), 21.5 (CH_3); HRMS (ESI) Exact mass calculated for $[C_{26}H_{24}ClNNaO_2S]^+$ $[M+Na]^+$: 472.1108, found: 472.1106. Enantiomeric excess was determined by HPLC using a Chiralpak AD-H column (98:2 *iso*-hexane:*i*-PrOH, 1.0 mL/min, 230 nm, 25 °C); t_r (minor) = 23.8 min, t_r (major) = 25.9 min, 93% ee.

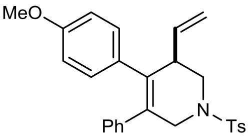

(*R*)-3-Ethenyl-4-(4-methoxyphenyl)-1-(4-methylbenzenesulfonyl)-5-phenyl-1,2,3,6-tetrahydropyridine (2c). Prepared according to General Procedure C using allylic phosphate **1c** (156 mg, 0.30 mmol) and phenylboronic acid (73.2 mg, 0.60 mmol). ^{1}H NMR analysis of the crude material showed the ratio of **2c**:**3c** was >19:1. Purification by flash column chromatography (10 to 20% EtOAc:petroleum ether) gave an off-white solid (94 mg, 70%). R_f = 0.26 (30% EtOAc:petroleum ether); m.p. 62–64 °C (Et_2O); IR 2924, 2835, 1607, 1510, 1493, 1456, 1442, 1341, 1305, 1290, 1245, 1162, 1091, 1061, 1032, 994, 966, 920, 832, 814, 802 cm^{-1}; $[\alpha]_D^{24}$ −290.2 (*c* 0.66, $CHCl_3$); ^{1}H NMR (400 MHz, $CDCl_3$) δ 7.75-7.71 (2H, m, Ar**H**), 7.37-7.34 (2H, m, Ar**H**), 7.19-7.12 (3H, m, Ar**H**), 7.02-6.99 (2H, m, Ar**H**), 6.85-6.81 (2H, m, Ar**H**), 6.66-6.62 (2H, m, Ar**H**), 5.87 (1H, ddd, *J* = 17.1, 10.3, 7.8 Hz, C**H**=CH_2), 5.04-4.94 (2H, m, CH=C$\mathbf{H_2}$), 4.36 (1H, d, *J* = 15.9 Hz, NC$\mathbf{H_a}H_b$C=C), 3.77 (1H, ddd, *J* = 11.4, 3.0, 0.9 Hz, NC$\mathbf{H_a}H_b$CH), 3.72 (3H, s, OC$\mathbf{H_3}$), 3.44-3.40 (1H, m, NC$H_a\mathbf{H_b}$C=C), 3.42-3.37 (1H, m, C**H**CH=CH_2), 3.03 (1H, dd, *J* = 11.4, 3.9 Hz, NC$H_a\mathbf{H_b}$CH), 2.46 (3H, s, ArC$\mathbf{H_3}$); ^{13}C NMR (100.6 MHz, $CDCl_3$) δ 158.0 (C), 143.6 (C), 139.3 (C), 137.1 (CH), 135.1 (C), 132.9 (C), 132.1 (C), 131.2 (C), 130.5 (2 × CH), 129.7 (2 × CH), 129.4 (2 × CH), 128.0 (2 × CH), 127.8 (2 × CH), 126.9 (CH), 116.9 (CH_2), 113.1 (2 × CH), 55.0 (CH_3), 49.4 (CH_2), 48.1 (CH_2), 44.7 (CH), 21.5 (CH_3); HRMS (ESI) Exact mass calculated for $[C_{27}H_{27}NNaO_3S]^+$ $[M+Na]^+$: 468.1604, found: 468.1601. Enantiomeric excess was determined by HPLC using a Chiralcel OD-H column (98:2 *iso*-hexane:*i*-PrOH, 1.0 mL/min, 280 nm, 25 °C); t_r (major) = 21.0 min, t_r (minor) = 24.3 min, 96% ee.

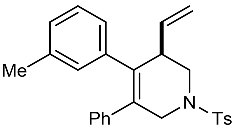

(*R*)-3-Ethenyl-1-(4-methylbenzenesulfonyl)-4-(3-methylphenyl)-5-phenyl-1,2,3,6-tetrahydropyridine (2d). Prepared according to General Procedure C using allylic phosphate **1d** (150 mg, 0.30 mmol) and phenylboronic acid (72.3 mg, 0.60 mmol). ^{1}H NMR analysis of the crude material showed a mixture of **2d** and **3d** (14:1). Purification by flash column chromatography (10% EtOAc:petroleum ether) gave an off-white solid (75 mg, 59%). R_f = 0.35 (30% EtOAc:petroleum ether); m.p. 161–162 °C (Et_2O); IR 3051, 2919, 1599, 1492, 1455, 1374, 1305, 1226, 1161, 1092, 1061, 988, 952, 912, 813, 790, 702, 651, 620, 566, 549 cm^{-1}; $[\alpha]_D^{20}$ −276.0 (*c* 1.00, CH_2Cl_2); ^{1}H NMR (400 MHz, $CDCl_3$) δ 7.79-7.66 (2H, m, Ar**H**),

7.41-7.29 (2H, m, Ar**H**), 7.14-7.11 (3H, m, Ar**H**), 7.00-6.94 (3H, m, Ar**H**), 6.88-6.86 (1H, m, Ar**H**), 6.71 (1H, d, *J* = 1.8 Hz, Ar**H**), 6.67 (1H, dd, *J* = 7.6, 1.5 Hz, Ar**H**), 5.85 (1H, ddd, *J* = 17.1, 10.4, 7.7 Hz, C**H**=CH_2), 5.03-4.97 (2H, m, CH=C$\mathbf{H}_2$), 4.35 (1H, *J* = 15.4 Hz, NC$\mathbf{H_a}$$H_b$C=C), 3.77-3.74 (1H, m, NC$\mathbf{H_a}$$H_b$CH), 3.45-3.39 (2H, m, NC$H_a$$\mathbf{H_b}$C=C and C**H**CH=$CH_2$), 3.04 (1H, dd, *J* = 11.4, 4.0 Hz, NC$H_a$$\mathbf{H_b}$CH), 2.44 (3H, s, ArC$\mathbf{H}_3$), 2.16 (3H, s, ArC$\mathbf{H}_3$); ^{13}C NMR (100.6 MHz, $CDCl_3$) δ 143.6 (C), 139.7 (C), 139.1 (C), 137.1 (C), 136.9 (CH), 135.7 (C), 132.9 (C), 131.5 (C), 130.0 (CH), 129.7 (2 × CH), 129.3 (2 × CH), 127.9 (2 × CH), 127.8 (2 × CH), 127.4 (CH), 127.2 (CH), 126.9 (CH), 126.6 (CH), 116.9 (CH_2), 49.3 (CH_2), 48.0 (CH_2), 44.6 (CH), 21.5 (CH_3), 21.3 (CH_3); HRMS (ESI) Exact mass calculated for $[C_{27}H_{27}NNaO_2S]^+$ $[M+Na]^+$: 452.1655, found: 452.1653. Enantiomeric excess was determined by HPLC using a Chiralcel OD-H column (98:2 *iso*-hexane:*i*-PrOH, 0.5 mL/min, 280 nm, 25 °C); t_r (major) = 25.2 min, t_r (minor) = 27.6 min, 96% ee.

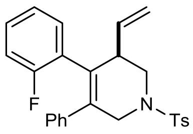

(*R*)-3-Ethenyl-4-(2-fluorophenyl)-1-(4-methylbenzenesulfonyl)-5-phenyl-1,2,3,6-tetrahydropyridine (2e). Prepared according to General Procedure C using allylic phosphate **1e** (152 mg, 0.30 mmol) and phenylboronic acid (73.2 mg, 0.60 mmol). ^{1}H NMR analysis of the crude material showed the ratio of **2e**:**3e** was >19:1. Purification by flash column chromatography (10 to 15% EtOAc:petroleum ether) gave an off-white solid (69 mg, 53%). R_f = 0.32 (10% EtOAc:petroleum ether); m.p. 73-74 °C (Et_2O); IR 3052, 2843, 1357, 1307, 1196, 1104, 997, 749, 775, 653 cm^{-1}; $[\alpha]_D^{20}$ –224.0 (*c* 1.00, CH_2Cl_2); ^{1}H NMR (400 MHz, $CDCl_3$) δ 7.79-7.65 (2H, m, Ar**H**), 7.42-7.30 (2H, m, Ar**H**), 7.18-6.99 (6H, m, Ar**H**), 6.89-6.81 (2H, m, Ar**H**), 6.80-6.71 (1H, m, Ar**H**), 5.96-5.76 (1H, m, C**H**=CH_2), 5.07-4.88 (2H, m, CH=C$\mathbf{H}_2$), 4.30 (1H, d, *J* = 16.6 Hz, NC$\mathbf{H_a}$$H_b$C=C), 3.64 (1H, dd, *J* = 11.4, 3.7 Hz, NC$\mathbf{H_a}$$H_b$CH), 3.51 (1H, dd, *J* = 16.5, 2.2 Hz, NC$H_a$$\mathbf{H_b}$C=C), 3.41-3.29 (1H, m, C**H**CH=CH_2), 3.14 (1H, dd, *J* = 11.4, 4.2 Hz, NC$H_a$$\mathbf{H_b}$CH), 2.45 (3H, s, ArC$\mathbf{H}_3$); ^{13}C NMR (100.6 MHz, $CDCl_3$) δ 159.9 (d, $^1J_{C\text{-}F}$ = 244.6 Hz, C), 143.7 (C), 138.6 (C), 136.4 (CH), 134.1 (C), 132.8 (C), 131.9 (CH), 130.9 (C), 129.7 (2 × CH), 128.7 (CH), 128.6 (2 × CH), 127.9 (2 × CH), 127.8 (2 × CH), 127.5 (C), 127.3 (CH), 123.4 (d, $^4J_{C\text{-}F}$ = 3.2 Hz, CH), 117.1 (CH_2), 115.0 (d, $^2J_{C\text{-}F}$ = 22.1 Hz, CH), 49.3 (CH_2), 47.8 (CH_2), 44.5 (CH), 21.5 (CH_3); HRMS (ESI) Exact mass calculated for $[C_{26}H_{24}FNNaO_2S]^+$ $[M+Na]^+$: 456.1404, found: 456.1411. Enantiomeric excess was determined by HPLC using a Chiralcel AD-H column (98:2 *iso*-hexane:*i*-PrOH, 1.0 mL/min, 230 nm, 25 °C); t_r (major) = 14.4 min, t_r (minor) = 15.8 min, 96% ee.

N.B. In the ^{13}C NMR spectrum, the coupling constant corresponding to $^3J_{C\text{-}F}$ is not reported, as one of the signals overlaps with the (2 x CH_2) peak at 128.6 ppm. The other corresponding peak is reported as (CH) at 128.7 ppm.

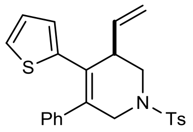

(*R*)-3-Ethenyl-1-(4-methylbenzenesulfonyl)-5-phenyl-4-(thiophen-2-yl)-1,2,3,6-tetrahydropyridine (2f). Prepared according to General Procedure C using allylic phosphate **1f** (149 mg, 0.30 mmol) and phenylboronic acid (73.2 mg, 0.60 mmol). ^{1}H NMR analysis of the crude material showed the ratio of **2f**:**3f** was >19:1. Purification by flash column chromatography (10% EtOAc:petroleum ether) gave an off-white solid (116 mg, 92%). R_f = 0.28 (10% EtOAc:petroleum ether); m.p. 146-147 °C (Et_2O); IR 2921, 2846, 1595, 1456, 1440, 1340, 1232, 1170, 1090, 1017, 992, 978, 820, 735, 655, 560 cm^{-1}; $[\alpha]_D^{21}$ −164.0 (*c* 1.00, CH_2Cl_2); ^{1}H NMR (400 MHz, $CDCl_3$) δ 7.76-7.62 (2H, m, Ar**H**), 7.38-7.23 (5H, m, Ar**H**), 7.15-7.08 (2H, m, Ar**H**), 7.02 (1H, dd, *J* = 5.1, 1.1 Hz, Ar**H**), 6.76-6.67 (1H, m, Ar**H**), 6.64-6.54 (1H, m, Ar**H**), 6.00 (1H, ddd, *J* = 17.5, 10.3, 7.3 Hz, C**H**=CH_2), 5.24-5.09 (2H, m, CH=C**H**$_2$), 4.31 (1H, d, *J* = 16.9 Hz, NC$\mathbf{H_a}$H$_b$C=C), 3.83 (1H, ddd, *J* = 11.4, 2.6 Hz, NC$\mathbf{H_a}$H$_b$CH), 3.45-3.40 (1H, m, C**H**CH=CH_2), 3.37 (1H, dd, *J* = 16.9, 2.0 Hz, NCH$_a\mathbf{H_b}$C=C), 2.94 (1H, dd, *J* = 11.4, 3.7 Hz, NCH$_a\mathbf{H_b}$CH), 3.03 (3H, s, ArC**H**$_3$); ^{13}C NMR (100.6 MHz, $CDCl_3$) δ 143.7 (C), 141.8 (C), 139.1 (C), 137.2 (CH), 132.8 (C), 132.6 (C), 129.7 (2 × CH), 129.2 (2 × CH), 128.6 (2 × CH), 128.1 (C), 127.79 (CH), 127.75 (2 × CH), 126.8 (CH), 126.2 (CH), 125.2 (CH), 117.4 (CH), 50.0 (CH_2), 48.0 (CH_2), 44.7 (CH), 21.5 (CH_3); HRMS (ESI) Exact mass calculated for $[C_{24}H_{23}NNaO_2S_2]^+$ $[M+Na]^+$: 444.1062, found: 444.1064. Enantiomeric excess was determined by HPLC using a Chiralcel IC column (98:2 *iso*-hexane:*i*-PrOH, 1.5 mL/min, 254 nm, 25 °C); t_r (major) = 38.9 min, t_r (minor) = 45.6 min, 90% ee.

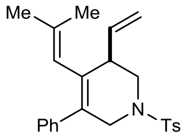

(*R*)-3-Ethenyl-1-(4-methylbenzenesulfonyl)-4-(2-methylprop-1-en-1-yl)-5-phenyl-1,2,3,6-tetrahydropyridine (2g). Prepared according to General Procedure C using allylic phosphate **1g** (141 mg, 0.30 mmol) and phenylboronic acid (73.3 mg, 0.60 mmol). ^{1}H NMR analysis of the crude material showed the ratio of **2g**:**3g** was >19:1. Purification by flash column chromatography (10% EtOAc:petroleum ether) gave a pale brown oil (53 mg, 45%). R_f = 0.30 (30% EtOAc:petroleum ether); IR 2925, 1597, 1493, 1444, 1377, 1161, 1018, 991, 962, 768, 730, 660, 546 cm^{-1}; $[\alpha]_D^{20}$ −44.0 (*c* 1.00, CH_2Cl_2); ^{1}H NMR (400 MHz, $CDCl_3$) δ 7.68 (2H, d, *J* = 8.3 Hz, Ar**H**), 7.33-7.32 (2H, m, Ar**H**), 7.28-7.23 (2H, m, Ar**H**), 7.22-7.17 (1H, m, Ar**H**), 7.16-7.13 (2H, m, Ar**H**), 5.85 (1H, ddd, *J* = 17.2, 10.2, 7.8 Hz, C**H**=CH_2), 5.41-5.40 (1H, m, $(CH_3)_2$C=C**H**), 5.17-5.10 (2H, m, CH=C**H**$_2$), 4.09 (1H, dd, *J* = 16.0, 1.8 Hz, NC$\mathbf{H_a}$H$_b$C=C), 3.53-3.43 (2H, m, NCH$_a\mathbf{H_b}$C=C and NC$\mathbf{H_a}$H$_b$CH), 3.01-2.95 (2H, m, C**H**CH=CH_2 and NCH$_a\mathbf{H_b}$CH), 2.42 (3H, s, ArC**H**$_3$), 1.50 (3H, d, *J* = 1.5 Hz, (C**H**$_3$)$_2$C=CH), 1.12 (3H, d, *J* = 1.3 Hz, (C**H**$_3$)$_2$C=CH); ^{13}C NMR (100.6 MHz, $CDCl_3$) δ 143.5 (C), 140.0 (C), 137.3 (CH), 135.4 (C), 132.9 (C), 132.5 (C), 130.7 (C), 129.6 (2 × CH), 128.7 (2 × CH), 127.84 (2 × CH), 127.77 (2 × CH), 126.9 (CH), 122.8 (CH), 116.4

(CH_2), 48.9 (CH_2), 47.7 (CH_2), 44.2 (CH), 25.3 (CH_3), 21.5 (CH_3), 19.7 (CH_3); HRMS (ESI) Exact mass calculated for $[C_{24}H_{27}NNaO_2S]^+$ $[M+Na]^+$: 416.1655, found: 416.1649. Enantiomeric excess was determined by HPLC using a Chiralcel AD-H column (98:2 *iso*-hexane:*i*-PrOH, 0.25 mL/min, 280 nm, 25 °C); t_r (minor) = 53.0 min, t_r (major) = 58.0 min, 49% ee.

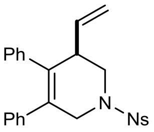

(*R*)-3-Ethenyl-1-(4-nitrobenzenesulfonyl)-4,5-diphenyl-1,2,3,6-tetrahydropyridine (2h). Prepared according to General Procedure C using allylic phosphate **1h** (156 mg, 0.30 mmol) and phenylboronic acid (73.2 mg, 0.60 mmol). ^{1}H NMR analysis of the crude material showed a mixture of **2h** and **3h** (16:1). Purification by flash column chromatography (10% EtOAc:petroleum ether) gave a yellow solid (75 mg, 56%). R_f = 0.28 (10% EtOAc:petroleum ether); m.p. 162-163 °C (Et_2O); IR 2923, 1606, 1530, 1443, 1402, 1312, 1264, 1163, 1087, 995, 853, 773, 695, 634, 619, 561 cm^{-1}; $[\alpha]_D^{20}$ –224.0 (*c* 1.00, CH_2Cl_2); ^{1}H NMR (400 MHz, $CDCl_3$) δ 8.46-8.34 (2H, m, Ar**H**), 8.10-7.95 (2H, m, Ar**H**), 7.17-7.11 (3H, m, Ar**H**), 7.10-7.05 (3H, m, Ar**H**), 7.01-6.96 (2H, m, Ar**H**), 6.92-6.88 (2H, m, Ar**H**), 5.83 (1H, ddd, *J* = 17.5, 10.3, 7.6 Hz, C**H**=CH_2), 5.06-4.97 (2H, m, CH=C**H**$_2$), 4.41 (1H, d, *J* = 16.2 Hz, NC$\mathbf{H_a}$$H_b$C=C), 3.83 (1H, dd, *J* = 11.5, 2.6 Hz, NC$\mathbf{H_a}$$H_b$CH), 3.52-3.41 (2H, m, NC$H_a$$\mathbf{H_b}$C=C and C**H**CH=$CH_2$), 3.13 (1H, dd, *J* = 11.4, 4.0 Hz, NC$H_a$$\mathbf{H_b}$CH); ^{13}C NMR (100.6 MHz, $CDCl_3$) δ 150.2 (C), 142.2 (C), 139.6 (C), 138.5 (C), 136.3 (CH), 135.9 (C), 131.3 (C), 129.3 (2 × CH), 129.2 (2 × CH), 128.9 (2 × CH), 128.1 (2 × CH), 127.8 (2 × CH), 127.3 (CH), 126.7 (CH), 124.4 (2 × CH), 117.5 (CH_2), 49.1 (CH_2), 48.0 (CH_2), 44.6 (CH); HRMS (ESI) Exact mass calculated for $[C_{25}H_{22}N_2NaO_4S]^+$ $[M+Na]^+$: 469.1192, found: 469.1200. Enantiomeric excess was determined by HPLC using a Chiralcel AD-H column (98:2 *iso*-hexane:*i*-PrOH, 0.8 mL/min, 254 nm, 25 °C); t_r (minor) = 48.7 min, t_r (major) = 51.2 min, 99% ee.

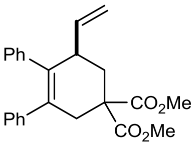

1,1-Dimethyl-(*R*)-5-ethenyl-3,4-diphenylcyclohex-3-ene-1,1-dicarboxylate (2i). Prepared according to General Procedure C using allylic phosphate **1i** (135 mg, 0.30 mmol) and phenylboronic acid (73.2 mg, 0.60 mmol). It was not possible to determine the ratio of **2i**:**3i** by ^{1}H NMR analysis of the crude material. Purification by flash column chromatography (10% EtOAc:petroleum ether) gave an orange oil (58 mg, 51%), which contained trace quantities of inseparable, unidentified impurities. R_f = 0.29 (10% EtOAc:petroleum ether); IR 2952, 1732, 1490, 1433, 1242, 1197, 1054, 1027, 913, 758, 698 cm^{-1}; $[\alpha]_D^{20}$ –64.0 (*c* 1.00, CH_2Cl_2); ^{1}H NMR (400 MHz, $CDCl_3$) δ 7.15-6.96 (8H, m, Ar**H**), 6.90-6.81 (2H, m, Ar**H**), 5.55 (1H, ddd, *J* = 17.1, 10.2, 8.0 Hz, C**H**=CH_2), 4.94-4.83 (2H, m, CH=C**H**$_2$), 3.81 (3H, s, OC**H**$_3$), 3.75 (3H, s, OC**H**$_3$),

3.52-3.42 (1H, m, C**H**CH=CH_2), 3.09 (1H, dt, *J* = 17.1, 2.9 Hz, CC**H$_a$**H$_b$C=C), 2.89 (1H, dd, *J* = 17.1, 1.8 Hz, CCH$_a$**H$_b$**C=C), 2.64 (1H, ddd, *J* = 13.6, 6.6, 2.0 Hz, CC**H$_a$**H$_b$CH), 2.22 (1H, dd, *J* = 13.6, 8.5 Hz, CCH$_a$**H$_b$**CH); ^{13}C NMR (100.6 MHz, $CDCl_3$) δ 172.0 (C), 171.5 (C), 142.7 (C), 140.8 (C), 139.8 (CH), 136.0 (C), 133.6 (C), 129.6 (2 × CH), 128.7 (2 × CH), 127.7 (2 × CH), 127.3 (2 × CH), 126.1 (CH), 125.9 (CH), 116.2 (CH_2), 53.3 (C), 52.7 (CH_3), 52.6 (CH_3), 43.2 (CH), 37.2 (CH_2), 34.7 (CH_2); HRMS (ESI) Exact mass calculated for $[C_{24}H_{24}NaO_4]^+$ $[M+Na]^+$: 399.1567, found: 399.1561. Enantiomeric excess was determined by HPLC using a Chiralcel OD-H column (98:2 *iso*-hexane:*i*-PrOH, 1.0 mL/min, 230 nm, 25 °C); t_r (minor) = 6.4 min, t_r (major) = 7.1 min, 96% ee.

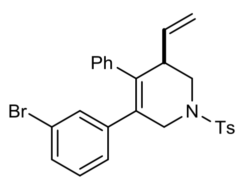

(*R*)-5-(3-Bromophenyl)-3-ethenyl-1-(4-methylbenzenesulfonyl)-4-phenyl-1,2,3,6-tetrahydropyridine (2j). Prepared according to General Procedure C using allylic phosphate **1a** (147 mg, 0.30 mmol) and 3-bromophenylboronic acid (147 mg, 0.60 mmol). ^{1}H NMR analysis of the crude material showed the ratio of **2j**:**3j** was >19:1. Purification by flash column chromatography (15% EtOAc:petroleum ether) gave a yellow solid (91 mg, 61%); R_f = 0.31 (15% EtOAc:petroleum ether); m.p. 64–65 °C (Et_2O); IR 2916, 1557, 1491, 1307, 1160, 1018, 971, 878, 764, 699, 652, 566 cm^{-1}; $[\alpha]_D^{20}$ –172.0 (*c* 1.00, CH_2Cl_2); ^{1}H NMR (400 MHz, $CDCl_3$) δ 7.75-7.70 (2H, m, Ar**H**), 7.38-7.34 (2H, m, Ar**H**), 7.24 (1H, ddd, *J* = 7.9, 2.0, 1.1 Hz, Ar**H**), 7.16 (1H, t, *J* = 1.8 Hz, Ar**H**) 7.15-7.06 (3H, m, Ar**H**), 6.97 (1H, t, *J* = 7.8 Hz, Ar**H**), 6.90-6.84 (3H, m, Ar**H**), 5.82 (1H, ddd, *J* = 17.1, 10.3, 7.8 Hz, C**H**=CH_2), 5.05-4.92 (2H, m, CH=C**H$_2$**), 4.28 (1H, dd, *J* = 16.1, 1.1 Hz, NC**H$_a$**H$_b$C=C), 3.72 (1H, ddd, *J* = 11.5, 3.3, 1.0 Hz, NC**H$_a$**H$_b$CH), 3.45-3.35 (2H, m, NCH$_a$**H$_b$**C=C and C**H**CH=CH_2), 3.05 (1H, dd, *J* = 11.5, 4.0 Hz, NCH$_a$**H$_b$**CH), 2.45 (3H, s, ArC**H$_3$**); ^{13}C NMR (100.6 MHz, $CDCl_3$) δ 143.8 (C), 141.2 (C), 139.4 (C), 137.0 (C), 136.5 (CH), 133.0 (C), 132.1 (CH), 130.4 (C), 130.1 (CH), 129.8 (2 × CH), 129.5 (CH), 129.3 (2 × CH), 128.1 (CH), 127.9 (2 × CH), 127.8 (2 × CH), 126.9 (CH), 122.0 (C), 117.2 (CH_2), 49.1 (CH_2), 48.0 (CH_2), 44.9 (CH), 21.5 (CH_3); HRMS (ESI) Exact mass calculated for $[C_{26}H_{24}BrNNaO_2S]^+$ $[M+Na]^+$: 516.0603, found 516.0600. Enantiomeric excess was determined by HPLC using a Chiralcel OD-H column (99:1 *iso*-hexane:*i*-PrOH, 1.0 mL/min, 254 nm, 25 °C); t_r (major) = 21.0 min, t_r (minor) = 24.0 min, 97% ee.

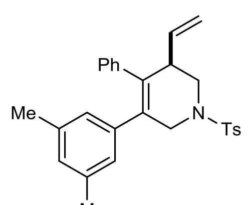

(*R*)-5-(3,5-Dimethylphenyl)-3-ethenyl-1-(4-methylbenzenesulfonyl)-4-phenyl-1,2,3,6-tetrahydropyridine (2k). Prepared according to General Procedure C using allylic phosphate **1a** (147 mg, 0.30 mmol) and 3,5-dimethylphenylboronic acid (90.0 mg, 0.60 mmol). ^{1}H NMR analysis of the

crude material showed the ratio of **2k**:**3k** was >19:1. Purification by flash column chromatography (10% EtOAc:pentane) gave a white solid (88 mg, 66%); R_f = 0.32 (10% EtOAc:pentane); m.p. 149–150 °C (Et_2O); IR 2958, 1639, 1492, 1464, 1398, 1302, 1289, 1241, 1092, 1012, 977, 855, 810, 766 cm^{-1}; $[\alpha]_D^{20}$ –276.0 (*c* 1.00, $CHCl_3$); ^1H NMR (400 MHz, $CDCl_3$) δ 7.73-7.69 (2H, m, Ar**H**), 7.36-7.31 (2H, m, Ar**H**), 7.12-7.03 (3H, m, Ar**H**), 6.92-6.87 (2H, m, Ar**H**) 6.74 (1H, s, Ar**H**), 6.59 (2H, s, Ar**H**), 5.85 (1H, ddd, *J* = 17.2, 10.3, 7.7 Hz, C**H**=CH_2), 5.02-4.92 (2H, m, CH=C$\mathbf{H_2}$), 4.34 (1H, d, *J* = 16.0 Hz, NC$\mathbf{H_a}H_b$C=C), 3.78-3.72 (1H, m, NC$\mathbf{H_a}H_b$CH), 3.42-3.34 (2H, m, NC$H_a\mathbf{H_b}$C=C and C**H**CH=CH_2), 2.99 (1H, dd, *J* = 11.3, 4.0 Hz, NC$H_a\mathbf{H_b}$CH), 2.44 (3H, s, ArC$\mathbf{H_3}$), 2.13 (6H, s, 2 × ArC$\mathbf{H_3}$); ^{13}C NMR (100.6 MHz, $CDCl_3$) δ 143.6 (C), 140.1 (C), 138.8 (C), 137.3 (2 × C), 136.9 (CH), 135.2 (C), 132.9 (C), 131.9 (C), 129.7 (2 × CH), 129.4 (2 × CH), 128.6 (CH), 127.8 (2 × CH), 127.6 (2 × CH), 127.1 (2 × CH), 126.4 (CH), 116.8 (CH_2), 49.4 (CH_2), 48.0 (CH_2), 44.8 (CH), 21.5 (CH_3), 21.1 (2 × CH_3); HRMS (ESI) Exact mass calculated for $[C_{28}H_{29}NNaO_2S]^+$ $[M+Na]^+$: 466.1811, found 466.1815. Enantiomeric excess was determined by HPLC using a Chiralcel AS-H column (98:2 *iso*-hexane:*i*-PrOH, 1.0 mL/min, 230 nm, 25 °C); t_r (minor) = 38.2 min, t_r (major) = 48.9 min, 97% ee.

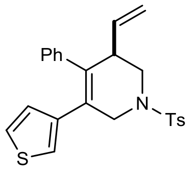

(*R*)-3-Ethenyl-1-(4-methylbenzenesulfonyl)-4-phenyl-5-(thiophen-3-yl)-1,2,3,6-tetrahydropyridine (2l). Prepared according to General Procedure C using allylic phosphate **1a** (143 mg, 0.30 mmol) and 3-thienylboronic acid (76.6 mg, 0.60 mmol). ^1H NMR analysis of the crude material showed the ratio of **2l**:**3l** was >19:1. Purification by flash column chromatography (10% EtOAc:petroleum ether) gave an off-white solid (62 mg, 49%). R_f = 0.31 (15% EtOAc:petroleum ether); m.p. 127–128 °C (Et_2O); IR 2919, 2851, 1636, 1492, 1400, 1339, 1235, 1161, 1090, 996, 968, 887, 868, 850, 782, 747, 699, 567, 547 cm^{-1}; $[\alpha]_D^{20}$ –248.0 (*c* 1.00, CH_2Cl_2); ^1H NMR (400 MHz, $CDCl_3$) δ 7.77-7.70 (2H, m, Ar**H**), 7.35 (2H, d, *J* = 8.0 Hz, Ar**H**), 7.21-7.15 (3H, m, Ar**H**), 7.00 (1H, dd, *J* = 5.0, 2.9 Hz, Ar**H**), 6.96-6.94 (2H, m, Ar**H**), 6.89 (1H, dd, *J* = 3.0, 1.3 Hz, Ar**H**), 6.48 (1H, dd, *J* = 5.0, 1.3 Hz, Ar**H**), 5.84 (1H, ddd, *J* = 17.1, 10.2, 7.8 Hz, C**H**=CH_2), 5.01-4.92 (2H, m, CH=C$\mathbf{H_2}$), 4.39 (1H, d, *J* = 16.1 Hz, NC$\mathbf{H_a}H_b$C=C), 3.77-3.73 (1H, m, NC$\mathbf{H_a}H_b$CH), 3.45 (1H, dd, *J* = 16.1, 2.1 Hz, NC$H_a\mathbf{H_b}$C=C), 3.35 (1H, tt, *J* = 5.3, 2.4 Hz, C**H**CH=CH_2), 3.00 (1H, dd, *J* = 11.4, 4.0 Hz, NC$H_a\mathbf{H_b}$CH), 2.45 (3H, s, ArC$\mathbf{H_3}$); ^{13}C NMR (100.6 MHz, $CDCl_3$) δ 143.7 (C), 140.5 (C), 138.9 (C), 136.6 (CH), 135.8 (C), 132.9 (C), 129.7 (2 × CH), 129.1 (2 × CH), 128.3 (CH), 128.0 (2 × CH), 127.8 (2 × CH), 126.9 (CH), 125.9 (C), 124.4 (CH), 123.3 (CH), 117.0 ($CH_{2,}$) 48.7 (CH_2), 47.9 (CH_2), 45.2 (CH), 21.5 (CH_3); HRMS (ESI) Exact mass calculated for $[C_{24}H_{23}NNaO_2S_2]^+$ $[M+Na]^+$: 444.1062, found: 444.1058. Enantiomeric excess

was determined by HPLC using a Chiralcel OD-H column (98:2 *iso*-hexane:*i*-PrOH, 0.8 mL/min, 254 nm, 25 °C); t_r (major) = 25.3 min, t_r (minor) = 32.2 min, 99% ee.

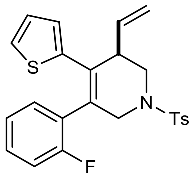

(*R*)-3-Ethenyl-5-(2-fluorophenyl)-1-(4-methylbenzenesulfonyl)-4-(thiophen-2-yl)-1,2,3,6-tetrahydropyridine (2m). Prepared according to General Procedure C using allylic phosphate **1f** (149 mg, 0.30 mmol) and 2-fluorophenylboronic acid (84.0 mg, 0.60 mmol). ^{1}H NMR analysis of the crude material showed the ratio of **2m**:**3m** was >19:1. Purification by flash column chromatography (10% EtOAc:pentane) gave a white solid (85 mg, 64%). R$_f$ = 0.21 (10% EtOAc:pentane); m.p. 56-57 °C (Et_2O); IR 3075, 2850, 1732, 1576, 1447, 1305, 1162, 1044, 992, 910, 816, 724 cm^{-1}; $[\alpha]_D^{20}$ −116.0 (*c* 1.00, $CHCl_3$); ^{1}H NMR (400 MHz, $CDCl_3$) δ 7.71-7.67 (2H, m, Ar**H**), 7.36-7.26 (3H, m, Ar**H**), 7.15-6.98 (4H, m, Ar**H**), 6.76 (1H, dd, *J* = 5.1, 3.7 Hz, Ar**H**), 6.69 (1H, dd, *J* = 3.6, 1.2 Hz, Ar**H**), 5.99 (1H, ddd, *J* = 17.3, 10.2, 7.2 Hz, C**H**=CH_2), 5.27-5.14 (2H, m, CH=C**H$_2$**), 4.23 (1H, d, *J* = 16.8 Hz, NC**H$_a$**H$_b$C=C), 3.81 (1H, ddd, *J* = 11.5, 2.8, 1.0 Hz, NC**H$_a$**H$_b$CH), 3.49-3.41 (1H, m, C**H**CH=CH_2), 3.42 (1H, dd, *J* = 16.8, 2.0 Hz, NCH$_a$**H$_b$**C=C), 3.00 (1H, dd, *J* = 11.5, 3.7 Hz, NCH$_a$**H$_b$**CH), 2.44 (3H, s, ArC**H$_3$**); ^{13}C NMR (100.6 MHz, $CDCl_3$) δ 160.0 (d, $^1J_{C-F}$ = 246.6 Hz, C), 143.7 (C), 141.3 (CH), 137.1 (CH), 133.1 (C), 131.6 (d, $^{3/4}J_{C-F}$ = 3.6 Hz, CH), 130.4 (C), 130.0 (d, $^3J_{C-F}$ = 8.1 Hz, CH), 129.7 (2 × CH), 127.7 (2 × CH), 126.6 (C), 126.5 (CH), 126.4 (C), 126.2 (CH), 125.4 (CH), 124.5 (d, $^{3/4}J_{C-F}$ = 3.6 Hz, CH), 117.8 (CH_2), 116.0 (d, $^2J_{C-F}$ = 22.0 Hz, CH), 49.1 (d, $^4J_{C-F}$ = 1.5 Hz, CH_2), 48.0 (CH_2), 44.5 (CH), 21.5 (CH_3); HRMS (ESI) Exact mass calculated for $[C_{24}H_{22}FNNaO_2S_2]^+$ $[M+Na]^+$: 462.0968, found: 462.0963. Enantiomeric excess was determined by HPLC using a Chiralcel OD-H column (98:2 *iso*-hexane:*i*-PrOH, 1.0 mL/min, 230 nm, 25 °C); t_r (major) = 22.3 min, t_r (minor) = 25.7 min, 96% ee.

N.B. In the ^{13}C NMR spectrum, the coupling constants corresponding to one of the $^2J_{C-F}$ and $^3J_{C-F}$ values are not reported, as they overlap with the (CH) peak at 126.5 ppm. The visible corresponding peaks are reported as (C) at 126.6 ppm and (C) at 126.4 ppm. For the signals at 131.6 and 124.5 ppm, it was not clear whether the *J*-values refer to three-bond or four-bond coupling constants as the magnitude of the *J*-values are identical.

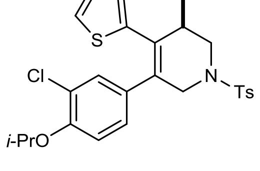

(*R*)-5-[3-Chloro-4-(propan-2-yloxy)phenyl]-3-ethenyl-1-(4-methylbenzenesulfonyl)-4-(thiophen-2-yl)-1,2,3,6-tetrahydropyridine (2n). Prepared according to General Procedure C using allylic phosphate **1f** (149 mg, 0.30 mmol) and 3-chloro-4-isopropoxyphenylboronic acid (128 mg, 0.60 mmol). ^{1}H NMR analysis of the crude material showed the ratio of **2n**:**3n** was >19:1. Purification

by flash column chromatography (10% EtOAc:petroleum ether) gave a yellow solid (91 mg, 61%). R_f = 0.29 (10% EtOAc:petroleum ether); m.p. 61-63 °C (Et_2O); IR 2921, 1596, 1491, 1340, 1284, 1259, 1162, 1056, 995, 879, 699 cm^{-1}; $[\alpha]_D^{20}$ −124.0 (*c* 1.00, CH_2Cl_2); ^{1}H NMR (400 MHz, $CDCl_3$) δ 7.72-7.67 (2H, m, Ar**H**), 7.37-7.31 (2H, m, Ar**H**), 7.12 (1H, d, *J* = 2.2 Hz, Ar**H**), 7.06 (1H, dd, *J* = 5.1, 1.2 Hz, Ar**H**), 6.93 (1H, dd, *J* = 8.5, 2.2 Hz, Ar**H**), 6.83 (1H, d, *J* = 8.5 Hz, Ar**H**), 6.78 (1H, dd, *J* = 5.1, 3.7 Hz, Ar**H**), 6.67 (1H, dd, *J* = 3.7, 1.2 Hz, Ar**H**), 5.96 (1H, ddd, *J* = 17.4, 10.2, 7.4 Hz, C**H**=CH_2), 5.20-5.10 (2H, m, CH=C$\mathbf{H}_2$), 4.54 (1H, hept, *J* = 6.1 Hz, $(CH_3)_2$C**H**), 4.25 (1H, d, *J* = 17.1 Hz, NC$\mathbf{H_a}$$H_b$C=C), 3.81 (1H, dd, *J* = 11.4, 3.1 Hz, NC$\mathbf{H_a}$$H_b$CH), 3.45-3.38 (1H, m, C**H**CH=CH_2), 3.34 (1H, dd, *J* = 17.0, 2.0 Hz, NC$H_a$$\mathbf{H_b}$C=C), 2.92 (1H, dd, *J* = 11.4, 3.7 Hz, NC$H_a$$\mathbf{H_b}$CH), 2.44 (3H, s, ArC$\mathbf{H}_3$), 1.38 (3H, d, *J* = 1.6 Hz, (C$\mathbf{H}_3$)$_2$CH), 1.37 (3H, d, *J* = 1.6 Hz, (C$\mathbf{H}_3$)$_2$CH); ^{13}C NMR (100.6 MHz, $CDCl_3$) δ 153.3 (C), 143.7 (C), 141.5 (C), 137.2 (CH), 132.9 (C), 132.1 (C), 131.1 (CH), 131.0 (C), 129.7 (2 × CH), 128.7 (C), 128.6 (CH), 127.8 (2 × CH), 126.9 (CH), 126.2 (CH), 125.5 (CH), 124.1 (C), 117.5 (CH_2), 115.6 (CH), 72.0 (CH), 49.9 (CH_2), 48.0 (CH_2), 44.7 (CH), 22.0 (2 × CH_3), 21.5 (CH_3); HRMS (ESI) Exact mass calculated for $[C_{27}H_{28}ClNNaO_3S_2]^+$ $[M+Na]^+$: 536.1091, found: 536.1094. Enantiomeric excess was determined by HPLC using a Chiralcel OD-H column (98:2 *iso*-hexane:*i*-PrOH, 0.3 mL/min, 210 nm, 25 °C); t_r (minor) = 70.2 min, t_r (major) = 76.4 min, 98% ee.

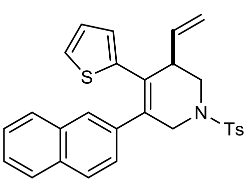

(*R*)-3-Ethenyl-1-(4-methylbenzenesulfonyl)-5-(naphthalen-2-yl)-4-(thiophen-2-yl)-1,2,3,6-tetrahydropyridine (2o). Prepared according to General Procedure C using allylic phosphate **1f** (149 mg, 0.30 mmol) and 2-napthylphenylboronic acid (103 mg, 0.60 mmol). ^{1}H NMR analysis of the crude material showed the ratio of **2o**:**3o** was >19:1. Purification by flash column chromatography (10% EtOAc:petroleum ether) gave a white solid (73 mg, 53%). R_f = 0.18 (10% EtOAc:petroleum ether); m.p. 142-143 °C (Et_2O); IR 2919, 1341, 1163, 1092, 996, 923, 811, 701, 676, 652, 547, 520 cm^{-1}; $[\alpha]_D^{20}$ −224.0 (*c* 1.00, CH_2Cl_2); ^{1}H NMR (400 MHz, $CDCl_3$) δ 7.83-7.76 (2H, m, Ar**H**), 7.73-7.67 (4H, m, Ar**H**), 7.51-7.46 (2H, m, Ar**H**), 7.35-7.30 (2H, m, Ar**H**), 7.15 (1H, dd, *J* = 8.4, 1.8 Hz, Ar**H**), 7.00 (1H, dd, *J* = 5.1, 1.2 Hz, Ar**H**), 6.71 (1H, dd, *J* = 5.1, 3.7 Hz, Ar**H**), 6.63 (1H, dd, *J* = 3.7, 1.2 Hz, Ar**H**), 6.03 (1H, ddd, *J* = 17.4, 10.2, 7.3 Hz, C**H**=CH_2), 5.24-5.14 (2H, m, CH=C$\mathbf{H}_2$), 4.44 (1H, d, *J* = 16.9 Hz, NC$\mathbf{H_a}$$H_b$C=C), 3.88 (1H, dd, *J* = 11.4, 1.5 Hz, NC$\mathbf{H_a}$$H_b$CH), 3.51-3.40 (2H, m, NC$H_a$$\mathbf{H_b}$C=C and C**H**CH=$CH_2$), 2.97 (1H, dd, *J* = 11.4, 3.7 Hz, NC$H_a$$\mathbf{H_b}$CH), 2.43 (3H, s, ArC$\mathbf{H}_3$); ^{13}C NMR (100.6 MHz, $CDCl_3$) δ 143.7 (C), 141.8 (C), 137.2 (CH), 136.6 (C), 133.3 (C), 132.8 (C), 132.7 (C), 132.4 (C), 129.7 (2 × CH), 128.6 (C), 128.15 (CH), 128.13 (CH), 128.0 (CH), 127.8 (2 × CH), 127.7 (CH), 127.4 (CH), 127.2 (CH), 126.31 (CH), 126.27 (CH), 126.2 (CH), 125.3 (CH), 117.5

(CH_2), 50.1 (CH_2), 48.1 (CH_2), 44.9 (CH), 21.5 (CH_3); HRMS (ESI) Exact mass calculated for $[C_{28}H_{25}NNaO_2S_2]^+$ $[M+Na]^+$: 494.1219, found: 494.1216. Enantiomeric excess was determined by HPLC using a Chiralcel AS-H column (90:10 *iso*-hexane:EtOH, 1.0 mL/min, 254 nm, 25 °C); t_r (minor) = 14.8 min, t_r (major) = 23.2 min, 97% ee.

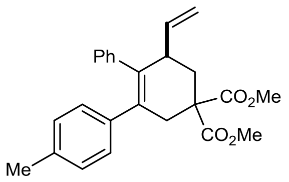

1,1-Dimethyl-(*R*)-5-ethenyl-3-(4-methylphenyl)-4-phenylcyclohex-3-ene-1,1-dicarboxylate (2p). Prepared according to General Procedure C using allylic phosphate **1i** (136 mg, 0.30 mmol) and 4-methylphenylboronic acid (81.7 mg, 0.60 mmol). It was not possible to determine the ratio of **2p**:**3p** by ^{1}H NMR analysis of the crude material. Purification by flash column chromatography (10% EtOAc:petroleum ether) gave a colorless oil (60 mg, 51%), which contained trace quantities of inseparable, unidentified impurities. R_f = 0.29 (30% EtOAc:petroleum ether); IR 3078, 3020, 1731, 1511, 1491, 1241, 1198, 1178, 1054, 915, 817, 761, 700, 518 cm^{-1}; $[\alpha]_D^{20}$ –152.0 (*c* 1.00, CH_2Cl_2); ^{1}H NMR (400 MHz, $CDCl_3$) δ 7.10-7.01 (3H, m, Ar**H**), 6.93- 6.86 (6H, m, Ar**H**), 5.55 (1H, ddd, *J* = 17.1, 10.1, 8.1 Hz, C**H**=CH_2), 4.92-4.86 (2H, m, CH=C**H**$_2$), 3.80 (3H, s, OC**H**$_3$), 3.75 (3H, s, OC**H**$_3$), 3.50-3.44 (1H, m, C**H**CH=CH_2), 3.09 (1H, dd, *J* = 17.1, 2.9 Hz, CC**H**$_{\mathbf{a}}$H$_b$C=C), 2.88 (1H, dt, *J* = 17.1, 1.8 Hz, CCH$_a$**H**$_{\mathbf{b}}$C=C), 2.64 (1H, ddd, *J* = 13.6, 6.6, 2.0 Hz, CC**H**$_{\mathbf{a}}$H$_b$CH), 2.26-2.19 (4H, m, ArC**H**$_3$ and CCH$_a$**H**$_{\mathbf{b}}$CH); ^{13}C NMR (100.6 MHz, $CDCl_3$) δ 172.0 (C), 171.5 (C), 141.0 (C), 139.9 (CH), 139.6 (C), 135.62 (C), 135.56 (C), 133.4 (C), 129.6 (2 × CH), 128.5 (2 × CH), 128.4 (2 × CH), 127.3 (2 × CH), 125.8 (CH), 116.1 (CH_2), 53.3 (C), 52.62 (CH_3), 52.59 (CH_3), 43.2 (CH), 37.3 (CH_2), 34.7 (CH_2), 21.0 (CH_3); HRMS (ESI) Exact mass calculated for $[C_{25}H_{26}NaO_4]^+$ $[M+Na]^+$: 413.1723, found: 413.1723. Enantiomeric excess was determined by HPLC using a Chiralcel AD-H column (99:1 *iso*-hexane:EtOH, 0.5 mL/min, 254 nm, 25 °C); t_r (major) = 13.1 min, t_r (minor) = 15.1 min, 96% ee.

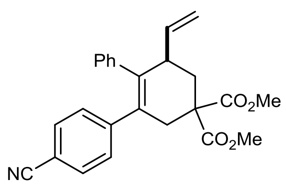

1,1-Dimethyl-(*R*)-3-(4-cyanophenyl)-5-ethenyl-4-phenylcyclohex-3-ene-1,1-dicarboxylate (2q). Prepared according to General Procedure C using allylic phosphate **1i** (136 mg, 0.30 mmol) and 4-cyanophenylboronic acid (88.3 mg, 0.60 mmol). It was not possible to determine the ratio of **2q**:**3q** by ^{1}H NMR analysis of the crude material. Purification by flash column chromatography (10% EtOAc:petroleum ether) gave a colorless oil (57 mg, 47%), which contained trace quantities of inseparable, unidentified impurities. R_f = 0.22 (15% EtOAc:petroleum ether); IR 2953, 2226, 1730, 1635, 1603, 1502, 1492, 1242, 1198, 1053, 912, 837, 763, 730, 701, 648, 567, 493 cm^{-1}; $[\alpha]_D^{20}$ –220.0

(*c* 1.00, $CHCl_3$); ^{1}H NMR (400 MHz, $CDCl_3$) δ 7.39-7.37 (2H, m, Ar**H**), 7.12-7.05 (5H, m, Ar**H**), 6.83-6.80 (2H, m, Ar**H**), 5.56-5.48 (1H, m, C**H**=CH_2), 4.92-4.87 (2H, m, CH=C**H**$_2$), 3.81 (3H, s, OC**H**$_3$), 3.75 (3H, s OC**H**$_3$), 3.48-3.41 (1H, m, C**H**CH=CH_2), 3.05 (1H, dd, *J* = 17.0, 3.0 Hz, CC**H$_a$**H_bC=C), 2.85 (1H, dt, *J* = 17.0, 1.8 Hz, CC**H$_a$**H_bC=C), 2.65 (1H, ddd, *J* = 13.7, 6.5, 2.0 Hz, CC**H$_a$**H_bCH), 2.22 (1H, dd, *J* = 13.7, 8.6 Hz, CCH_a**H$_b$**CH); ^{13}C NMR (100.6 MHz, $CDCl_3$) δ 171.6 (C), 171.1 (C), 147.6 (C), 139.8 (CH), 139.1 (C), 138.2 (C), 132.1 (C), 131.6 (2 × CH), 129.5 (2 × CH), 129.4 (2 × CH), 127.7 (2 × CH), 126.5 (CH), 118.9 (C), 116.6 (CH_2), 109.8 (C), 53.1 (C), 52.79 (CH_3), 52.75 (CH_3), 43.3 (CH), 36.6 (CH_2), 34.4 (CH_2); HRMS (ESI) Exact mass calculated for $[C_{25}H_{23}NNaO_4]^+$ $[M+Na]^+$: 424.1519, found: 424.1517. Enantiomeric excess was determined by HPLC using a Chiralcel OD-H column (99:1 *iso*-hexane:*i*-PrOH, 0.4 mL/min, 254 nm, 25 °C); t_r (minor) = 65.7 min, t_r (major) = 69.1 min, 98% ee.

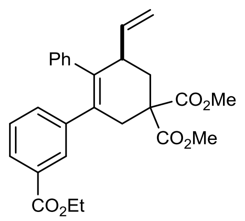

1,1-Dimethyl-(*R*)-5-ethenyl-3-[3-(ethoxycarbonyl)phenyl]-4-phenylcyclohex-3-ene-1,1-dicarboxylate (2r). Prepared according to General Procedure C using allylic phosphate **1i** (136 mg, 0.30 mmol) and 3-ethoxycarbonylphenylboronic acid (117 mg, 0.60 mmol). It was not possible to determine the ratio of **2r**:**3r** by ^{1}H NMR analysis of the crude material. Purification by flash column chromatography (10% EtOAc:petroleum ether) gave a colorless oil (71 mg, 53%), which contained trace quantities of inseparable, unidentified impurities. R_f = 0.25 (30% EtOAc:petroleum ether); IR 2953, 1732, 1716, 1453, 1391, 1285, 1248, 1217, 1105, 1081, 970, 755, 730, 699 cm^{-1}; $[\alpha]_D^{20}$ −132.0 (*c* 1.00, CH_2Cl_2); ^{1}H NMR (400 MHz, $CDCl_3$) δ 7.75-7.72 (2H, m, Ar**H**), 7.15-6.99 (5H, m, Ar**H**), 6.86-6.83 (2H, m, Ar**H**), 5.54 (1H, ddd, *J* = 17.1, 10.2, 8.1 Hz, C**H**=CH_2), 4.93-4.87 (2H, m, CH=C**H**$_2$), 4.35-4.30 (2H, m, C**H**$_2$$CH_3$), 3.82 (3H, s, OC**H**$_3$), 3.76 (3H, s, OC**H**$_3$), 3.46 (1H, dd, *J* = 11.7, 4.9 Hz, C**H**CH=CH_2), 3.11 (1H, dd, *J* = 17.0, 3.1 Hz, CC**H$_a$**H_bC=C), 2.90 (1H, dt, *J* = 17.0, 1.9 Hz, CC**H$_a$**H_bC=C), 2.65 (1H, ddd, *J* = 13.7, 6.6, 2.1 Hz, CC**H$_a$**H_bCH), 2.21 (1H, dd, *J* = 13.7, 8.8 Hz, CCH_a**H$_b$**CH), 1.36 (3H, t, *J* = 7.1 Hz, CH_2C**H$_3$**); ^{13}C NMR (100.6 MHz, $CDCl_3$) δ 171.9 (C), 171.3 (C), 166.5 (C), 142.8 (C), 140.3 (C), 139.6 (CH), 137.1 (C), 133.4 (CH), 132.6 (C), 130.0 (C), 129.61 (CH), 129.58 (2 × CH), 127.7 (CH), 127.5 (2 × CH), 127.4 (CH), 126.1 (CH), 116.3 (CH_2), 60.8 (CH_2), 53.4 (C), 52.7 (2 x CH_3), 43.3 (CH), 36.9 (CH_2), 34.7 (CH_2), 14.3 (CH_3); HRMS (ESI) Exact mass calculated for $[C_{27}H_{28}NaO_6]^+$ $[M+Na]^+$: 471.1778, found: 471.1770. Enantiomeric excess was determined by HPLC using a Chiralcel OD-H column (98:2 *iso*-hexane: *i*-PrOH, 0.3 mL/min, 254 nm, 25 °C); t_r (minor) = 42.8 min, t_r (major) = 51.4 min, 98% ee.

Further Exploration of Substrate Scope

(*S*)-3-Ethenyl-1-(4-methylbenzenesulfonyl)-5-phenyl-4-(trimethylsilyl)-1,2,3,6-tetrahydropyridine (2s)

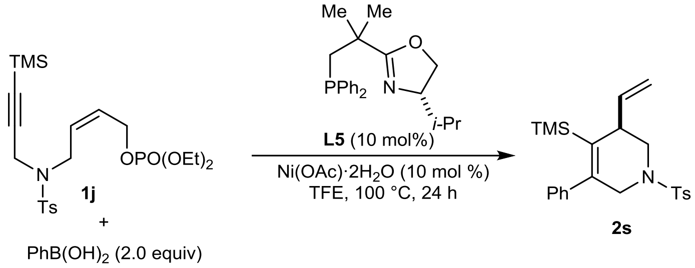

Allylic phosphate **1j** (141 mg, 0.30 mmol), phenylboronic acid (73.4 mg, 0.60 mmol), $Ni(OAc)_2{\cdot}4H_2O$ (7.47 mg, 0.03 mmol) and (*S*)-*i*-Pr-NeoPHOX (**L5**, 10.6 mg, 0.03 mmol) were added to a microwave vial containing a magnetic stirrer bar. The vial was then sealed with a septum cap and the contents evacuated and charged with argon (3 cycles). 2,2,2-Trifluoroethanol (3 mL) which had been freshly degassed (using 5 freeze-pump-thaw cycles) was then added under argon flow, the septum re-sealed with a layer of vacuum grease, and the contents stirred at 100 °C for 24 h. The reaction was cooled to room temperature, diluted with EtOAc (50 mL) and washed with 50% brine (50 mL). The organic layer was dried ($MgSO_4$), filtered and concentrated *in vacuo* to give the crude mixture. ^{1}H NMR analysis of the crude material showed no evidence for formation of the five-membered isomer. Purification by flash column chromatography (10% EtOAc:petroleum ether) gave a colorless oil (90 mg, 70%). R_f = 0.38 (30% EtOAc:petroleum ether); IR 2951, 2809, 1625, 1596, 1492, 1453, 1350, 1248, 1237, 1163, 1093, 1000, 918, 835, 777, 728, 705, 691, 654, 559, 546 cm^{-1}; $[\alpha]_D^{20}$ –16.0 (*c* 1.00, CH_2Cl_2); ^{1}H NMR (400 MHz, $CDCl_3$) δ 7.65 (2H, d, *J* = 8.1 Hz, Ar**H**), 7.32-7.30 (5H, m, Ar**H**), 7.13-7.10 (2H, m, Ar**H**), 5.90 (1H, dddd, *J* = 17.0, 10.2, 7.8, 1.2 Hz, C**H**=CH_2), 5.23-5.17 (2H, m, CH=C**H**$_2$), 3.81 (1H, d, *J* = 16.9 Hz, NC**H**$_a$H$_b$C=C), 3.53-3.49 (1H, m, NC**H**$_a$H$_b$CH), 3.38 (1H, dd, *J* = 11.3, 4.0 Hz, NCH$_a$**H**$_b$C=C), 3.20-3.18 (1H, m, C**H**CH=CH_2), 2.92 (1H, dd, *J* = 11.3, 4.0 Hz, NCH$_a$**H**$_b$CH), 2.43 (3H, s, ArC**H**$_3$), –0.25 (9H, s, Si(C**H**$_3$)$_3$); ^{13}C NMR (100.6 MHz, $CDCl_3$) δ 145.3 (C), 143.5 (C), 141.5 (C), 139.0 (CH), 134.8 (C), 133.1 (C), 129.6 (2 × CH), 128.9 (2 × CH), 128.1 (2 × CH), 127.8 (CH), 127.7 (2 × CH), 117.4 (CH_2), 50.6 (CH_2), 47.9 (CH_2), 42.3 (CH), 21.5 (CH_3), 0.5 (3 × CH_3); HRMS (ESI) Exact mass calculated for $[C_{23}H_{29}NNaO_2SSi]^+$ $[M+Na]^+$: 434.1580, found: 434.1582. Enantiomeric excess was determined by HPLC using a Chiralcel AD-H column (98:2 *iso*-hexane:*i*-PrOH, 1.0 mL/min, 254 nm, 25 °C); t_r (minor) = 10.5 min, t_r (major) = 11.9 min, 69% ee.

(*E*)-4-{[*N*-(2,3-Diphenylallyl)-4-methylphenyl]sulfonamido}but-2-en-1-yl diethyl phosphate (5)

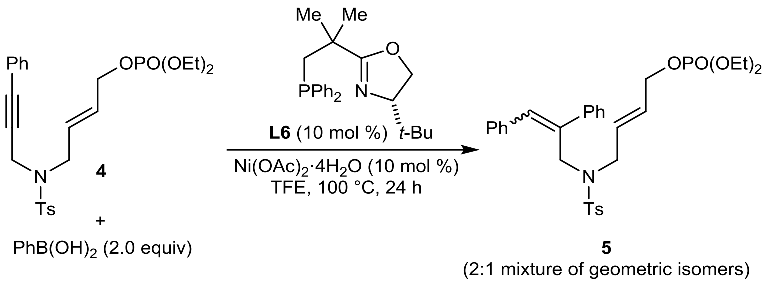

Allylic phosphate **4** (147 mg, 0.30 mmol), phenylboronic acid (73.4 mg, 0.60 mmol), $Ni(OAc)_2{\cdot}4H_2O$ (7.5 mg, 0.03 mmol) and (*S*)-*t*-Bu-NeoPHOX (**L6**, 11.0 mg, 0.03 mmol) were added to a microwave vial containing a magnetic stirrer bar. The vial was then sealed with a septum cap and the contents evacuated and charged with argon (3 cycles). 2,2,2-Trifluoroethanol (3 mL) which had been freshly degassed (using 5 freeze-pump-thaw cycles) was then added under argon flow, the septum re-sealed with a layer of vacuum grease, and the contents stirred at 100 °C for 24 h. The reaction was cooled to room temperature, diluted with EtOAc (50 mL) and washed with 50% brine (50 mL). The organic layer was dried ($MgSO_4$), filtered and concentrated *in vacuo* to give the crude mixture. ^{1}H NMR analysis of the crude material showed a mixture of geometric isomers of **5** (2:1). Purification by flash column chromatography (80% EtOAc:petroleum ether) gave a colorless oil (109 mg, 63%). R_f = 0.32 (60% EtOAc:petroleum ether); IR 3054, 1731, 1576, 1446, 1340, 1264, 1091, 1028, 975, 873, 805, 699, 545 cm^{-1}.

Characteristic signals for major isomer: ^{1}H NMR (400 MHz, $CDCl_3$) δ 6.94 (1H, s, (Ph)C**H**=C), 5.31-5.18 (2H, m, C**H**=C**H**), 4.53-4.51 (2H, m, =CHC**H**$_2$O), 3.47-3.36 (2H, m, NC**H**$_2$CH=), 2.40 (3H, s, ArC**H**$_3$); ^{13}C NMR (100.6 MHz, $CDCl_3$) δ 143.2 (C), 140.2 (C), 137.3 (C), 136.5 (C), 66.4 (d, $^2J_{C\text{-}P}$ = 5.6 Hz, CH_2), 63.7 (d, $^2J_{C\text{-}P}$ = 5.9 Hz, 2 × CH_2), 47.8 (CH_2), 44.5 (CH_2), 21.5 (CH_3), 16.1 (d, $^3J_{C\text{-}P}$ = 6.6 Hz, 2 × CH_3).

Characteristic signals for minor isomer: ^{1}H NMR (400 MHz, $CDCl_3$) δ 6.54 (1H, s, (Ph)C**H**=C), 5.74 (1H, dt, *J* = 15.6, 5.5 Hz), =C**H**CH$_2$O), 5.69-5.60 (1H, m, NCH$_2$C**H**=), 4.47 (2H, ddd, *J* = 7.8, 5.4, 1.1 Hz, =CHC**H**$_2$O), 4.24-4.20 (2H, m, =CC**H**$_2$N), 3.80 (2H, d, *J* = 6.1 Hz, NC**H**$_2$CH=), 2.40 (3H, s, ArC**H**$_3$); ^{13}C NMR (100.6 MHz, $CDCl_3$) δ 138.2 (C), 137.0 (C), 136.0 (C) 66.7 (d, $^2J_{C\text{-}P}$ = 5.4 Hz, CH_2), 63.8 (d, $^2J_{C\text{-}P}$ = 5.8 Hz, 2 × CH_2), 54.1 (CH_2), 48.3 (CH_2).

Aromatic signals in the ^{1}H NMR spectrum for both isomers overlap in the region of 7.57-7.04 ppm. Signals corresponding to (2 × OC**H**$_2$CH$_3$ and 2 × OCH$_2$C**H**$_3$) for both isomers overlap in the region of 4.15-4.01 ppm and 1.37-1.30 ppm respectively. Aromatic signals in the ^{13}C NMR spectrum for both isomers overlap in the region of 143.3 to 127.0 ppm.

HRMS (ESI) Exact mass calculated for $[C_{30}H_{36}NNaO_6PS]^+$ $[M+Na]^+$: 592.1893, found: 592.1883.

(5-Ethenyl-2-phenylcyclopent-1-en-1-yl)benzene (7)

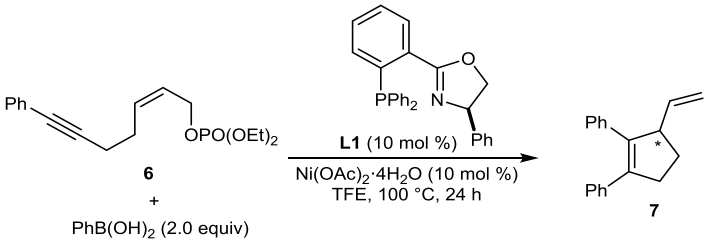

Allylic phosphate **6** (97 mg, 0.30 mmol), phenylboronic acid (73.4 mg, 0.60 mmol), $Ni(OAc)_2{\cdot}4H_2O$ (7.5 mg, 0.03 mmol) and (*R*)-Ph-PHOX (**L1**, 12.2 mg, 0.03 mmol) were added to a microwave vial containing a magnetic stirrer bar. The vial was then sealed with a septum cap and the contents evacuated and charged with argon (3 cycles). 2,2,2-Trifluoroethanol (3 mL) which had been freshly degassed (using 5 freeze-pump-thaw cycles) was then added under argon flow, the septum re-sealed with a layer of vacuum grease, and the contents stirred at 100 °C for 24 h. The reaction was cooled to room temperature, diluted with EtOAc (50 mL) and washed with 50% brine (50 mL). The organic layer was dried ($MgSO_4$), filtered and concentrated *in vacuo* to give the crude mixture. Purification by flash column chromatography (4% EtOAc:petroleum ether) gave a pale brown oil (48 mg, 64%). R_f = 0.60 (15% EtOAc:petroleum ether); IR 2926, 2843, 1634, 1597, 1573, 1489, 1441, 1416, 1340, 1260, 1155, 1066, 1029, 990, 910, 805, 694, 553 cm^{-1}; $[\alpha]_D^{21}$ −36.0 (*c* 1.00, CH_2Cl_2); ^1H NMR (400 MHz, $CDCl_3$) δ 7.25-7.13 (10H, m, Ar**H**), 5.87-5.78 (1H, m, C**H**=CH_2), 5.05-4.93 (2H, m, CH=C$\mathbf{H_2}$), 3.83-3.79 (1H, m, C**H**CH=CH_2), 3.10-2.99 (1H, m, C$\mathbf{H_a}$$H_b$C=C), 2.82-2.71 (1H, m, C$H_a$$\mathbf{H_b}$C=C), 2.36 (1H, dtd, *J* = 12.9, 8.7, 6.4 Hz, =CCH_2C$\mathbf{H_a}$$H_b$), 1.95-1.89 (1H, m, =C$CH_2CH_a$$\mathbf{H_b}$); ^{13}C NMR (100.6 MHz, $CDCl_3$) δ 140.9 (CH), 140.0 (C), 138.5 (C), 137.9 (C), 137.7 (C), 128.9 (2 × CH), 128.2 (2 × CH), 128.0 (2 × CH), 127.9 (2 x CH), 126.6 (CH), 126.5 (CH), 114.3 (CH_2), 55.1 (CH), 36.7 (CH_2), 30.0 (CH_2); (GC/MS) Exact mass calculated for $C_{19}H_{18}$ $[M]^+$: 246.1403, found: 246.1399. Enantiomeric excess was determined by HPLC using a Chiralcel OD-H column (*iso*-hexane, 1.0 mL/min, 230 nm, 25 °C); t_r (minor) = 9.5 min, t_r (major) = 10.3 min, 42% ee.

(*R*,*E*)-4-Phenyl-5-styryl-1-tosyl-3-vinyl-1,2,3,6-tetrahydropyridine (8)

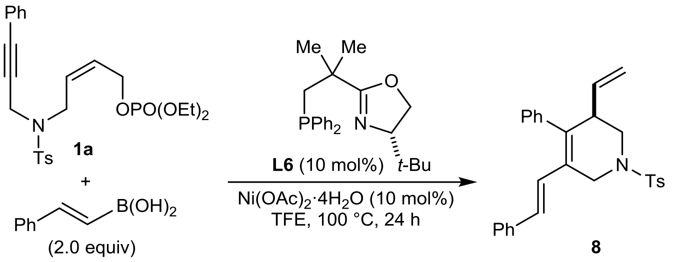

Allylic phosphate **1a** (147 mg, 0.30 mmol), *trans*-2-phenylvinylboronic acid (73.2 mg, 0.60 mmol), $Ni(OAc)_2{\cdot}4H_2O$ (7.5 mg, 0.03 mmol) and (*S*)-*t*-Bu-NeoPHOX (**L6**, 11.0 mg, 0.03 mmol) were added to a microwave vial containing a magnetic stirrer bar. The vial was then sealed with a septum cap and the contents evacuated and charged with argon (3 cycles). 2,2,2-Trifluoroethanol (3 mL) which had been freshly degassed (using 5 freeze-pump-thaw cycles) was then added under argon flow, the septum re-sealed with a layer of vacuum grease, and the contents stirred at 100 °C for 24 h. The reaction was cooled to room temperature, diluted with EtOAc (50 mL) and washed with 50% brine (50 mL). The organic layer was dried ($MgSO_4$), filtered and concentrated *in vacuo* to give the crude mixture. ^{1}H NMR analysis of the crude material showed no evidence for formation of the five-membered isomer. Purification by flash column chromatography (10% EtOAc:*n*-pentane) gave an off-white gum (17 mg, 13%). R_f = 0.20 (10% EtOAc:*n*-pentane); IR 3024, 2922, 2851, 1596, 1492, 1342, 1306, 1261, 1010, 958, 873, 772 cm^{-1}; $[\alpha]_D^{20}$ −100.0 (*c* 0.20, $CHCl_3$); ^{1}H NMR (400 MHz, $CDCl_3$) δ 7.83-7.77 (2H, m, Ar**H**), 7.42-7.28 (5H, m, Ar**H**), 7.26-7.16 (5H, m, Ar**H**), 7.12-7.08 (2H, m, Ar**H**), 6.67 (1H, d, *J* = 16.6 Hz, PhC**H**=CH), 6.47 (1H, d, *J* = 16.6 Hz, PhCH=C**H**), 5.78 (1H, ddd, *J* = 17.1, 10.2, 8.0 Hz, C**H**=CH_2), 5.02-4.87 (2H, m, CH=C**H_2**), 4.37 (1H, d, *J* = 15.5 Hz, NC**H_a**H_bC=C), 3.69-3.59 (2H, m, NC**H_a**H_bCH and NCH_a**H_b**C=C), 3.34-3.29 (1H, m, C**H**CH=CH_2), 3.05 (1H, dd, *J* = 11.4, 4.1 Hz, NCH_a**H_b**CH), 2.46 (3H, s, ArC**H_3**); ^{13}C NMR (100.6 MHz, $CDCl_3$) δ 143.7 (C). 139.5 (C), 139.1 (C), 137.1 (C), 136.6 (CH), 133.1 (C), 129.8 (2 × CH), 129.4 (2 × CH), 128.6 (2 × CH), 128.1 (2 × CH), 127.8 (2 × CH), 127.6 (CH), 127.4 (CH), 127.3 (CH), 127.2 (C), 126.4 (2 × CH), 125.8 (CH), 117.1 (CH_2), 47.9 (CH_2), 45.6 (CH), 45.2 (CH_2), 21.6 (CH_3); HRMS (ESI) Exact mass calculated for $[C_{28}H_{28}NO_2S]^+$ $[M+H]^+$: 442.1835, found: 442.1834. Enantiomeric excess was determined by HPLC using a Chiralcel AD-H column (99:1 *iso*-hexane:*i*-PrOH, 1.0 mL/min, 280 nm, 25 °C); t_r (minor) = 34.8 min, t_r (major) = 43.2 min, 98% ee.

NMR Spectra

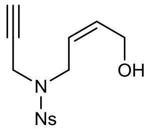

S12

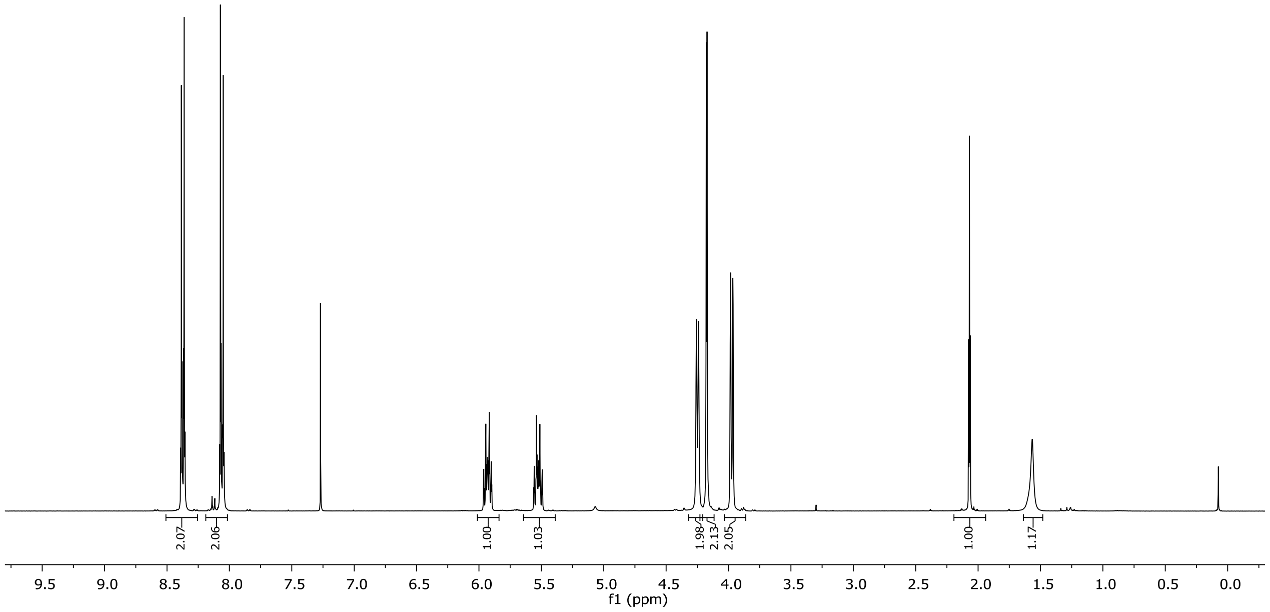

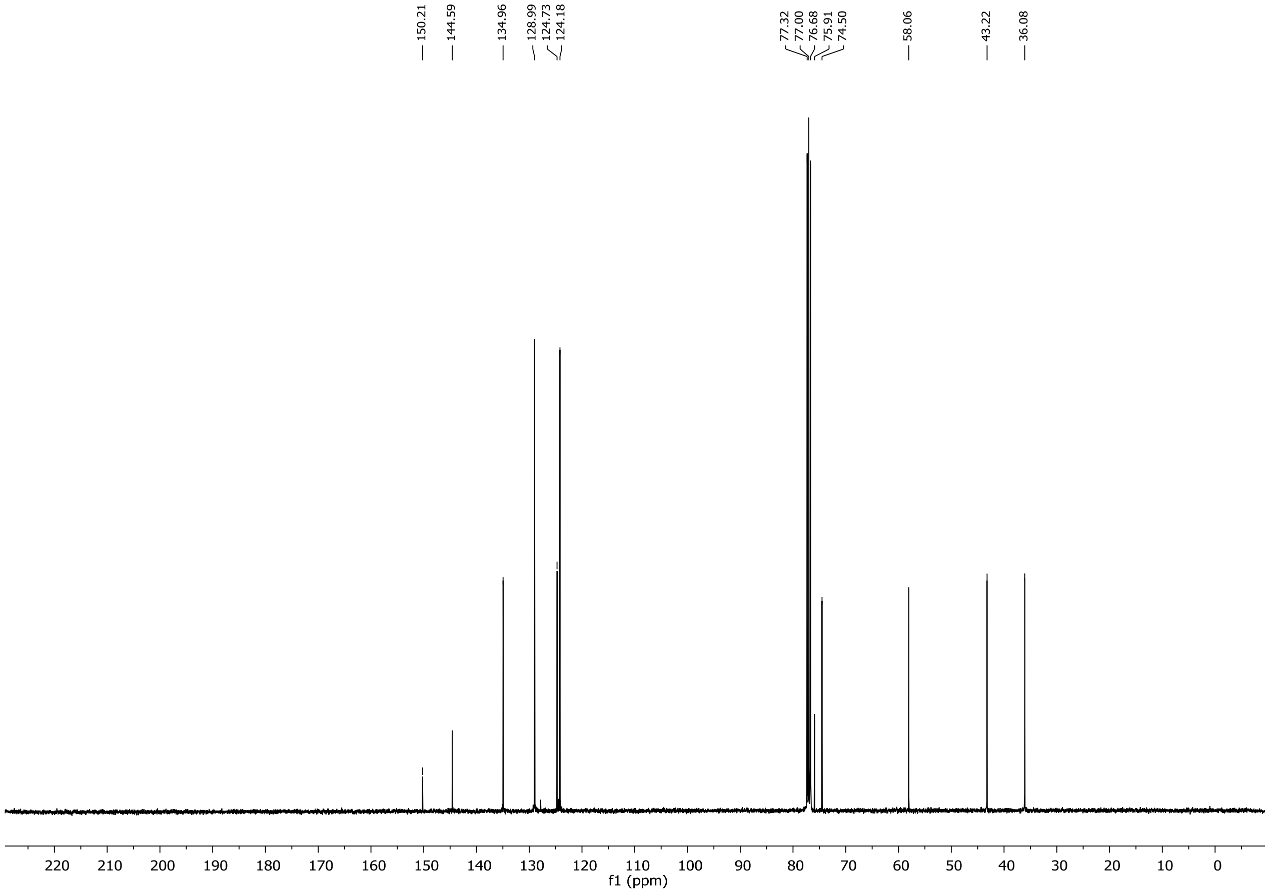

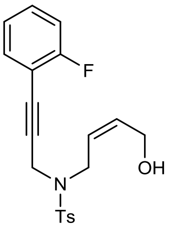
F
N
OH
Ts

S13

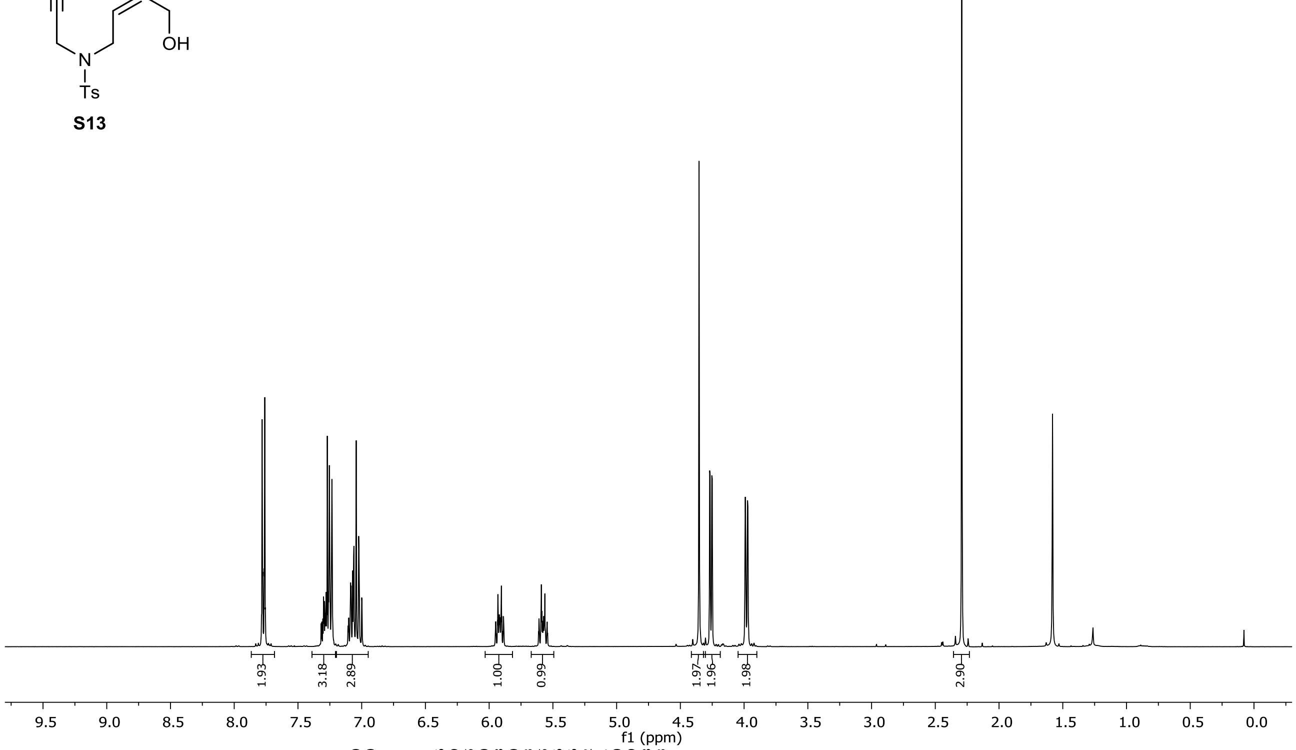
1.93
3.18
2.89
1.00
0.99
1.97
1.96
1.98
2.90
9.5 9.0 8.5 8.0 7.5 7.0 6.5 6.0 5.5 5.0 4.5 4.0 3.5 3.0 2.5 2.0 1.5 1.0 0.5 0.0
f1 (ppm)

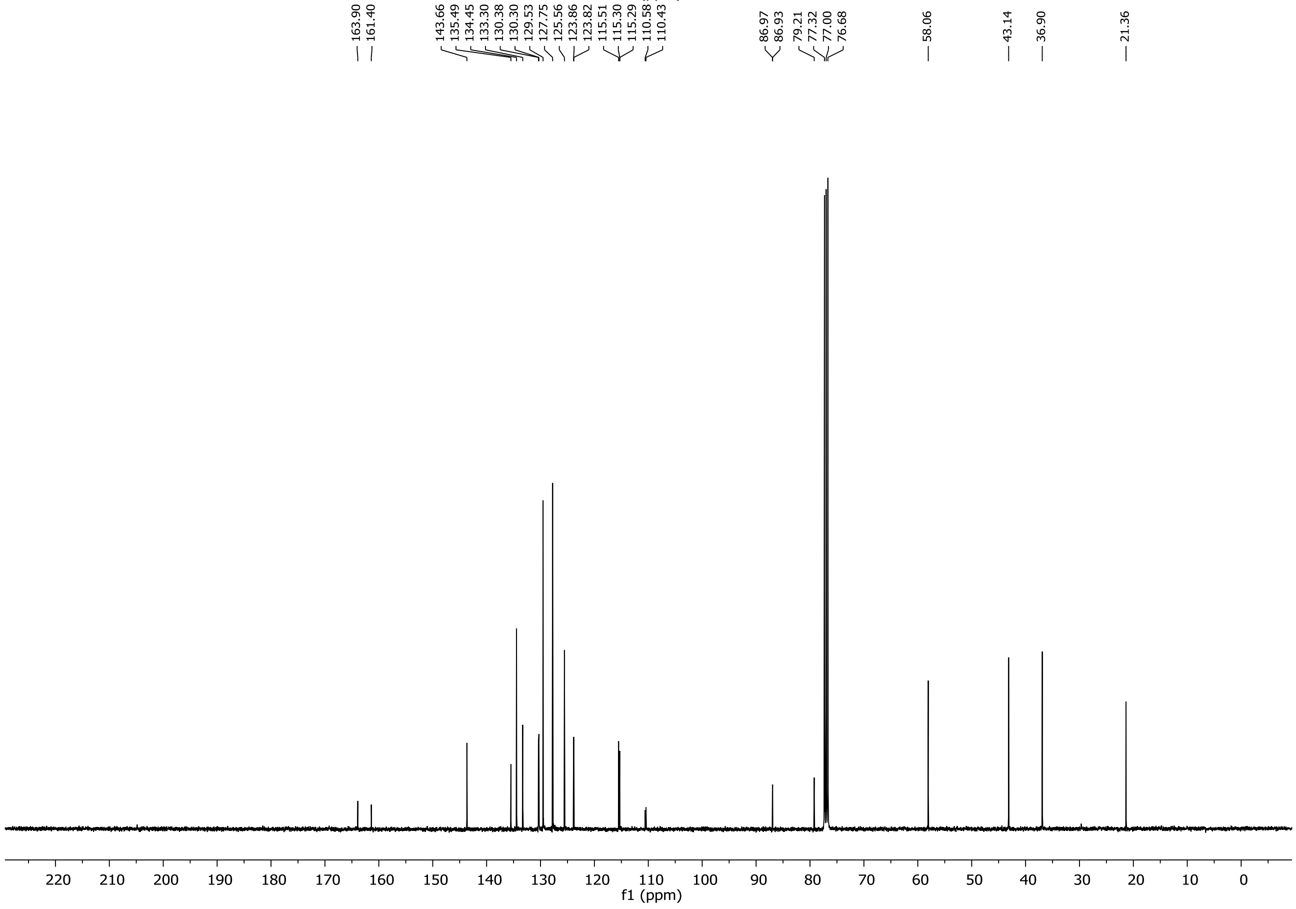
163.90
161.40
143.66
135.49
134.45
133.30
130.38
130.30
129.53
127.75
125.56
123.86
123.82
115.51
115.30
115.29
110.58
110.43
86.97
86.93
79.21
77.32
77.00
76.68
58.06
43.14
36.90
21.36
220 210 200 190 180 170 160 150 140 130 120 110 100 90 80 70 60 50 40 30 20 10 0
f1 (ppm)

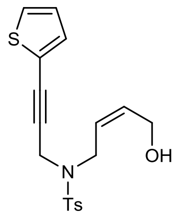
S
N
OH
Ts

S14

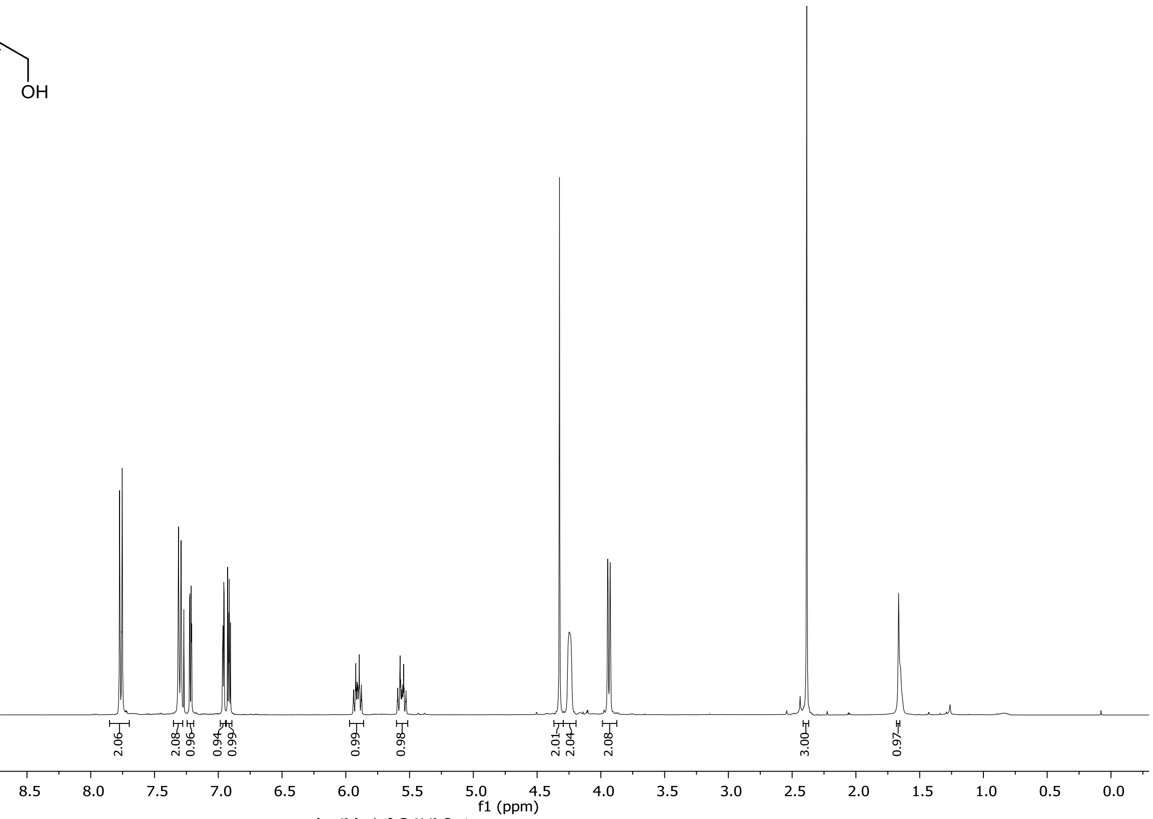
2.06
2.08
0.96
0.94
0.99
0.99
0.98
2.01
2.04
2.08
3.00
0.97
9.5 9.0 8.5 8.0 7.5 7.0 6.5 6.0 5.5 5.0 4.5 4.0 3.5 3.0 2.5 2.0 1.5 1.0 0.5 0.0
f1 (ppm)

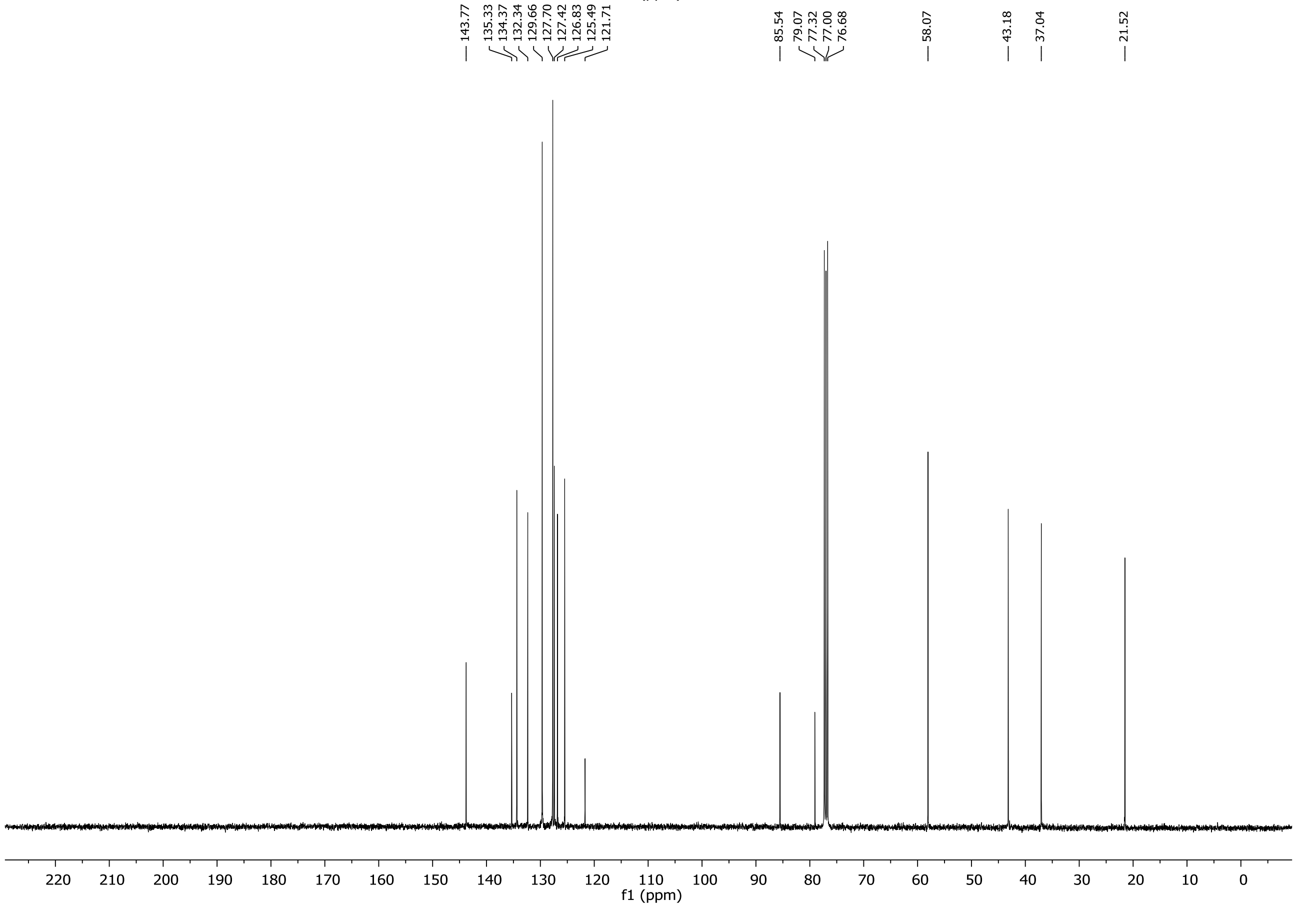
143.77
135.33
134.37
132.34
129.66
127.70
127.42
126.83
125.49
121.71
85.54
79.07
77.32
77.00
76.68
58.07
43.18
37.04
21.52
220 210 200 190 180 170 160 150 140 130 120 110 100 90 80 70 60 50 40 30 20 10 0
f1 (ppm)

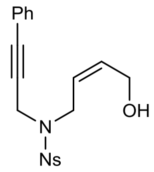
Ph
N
Ns
OH

S15

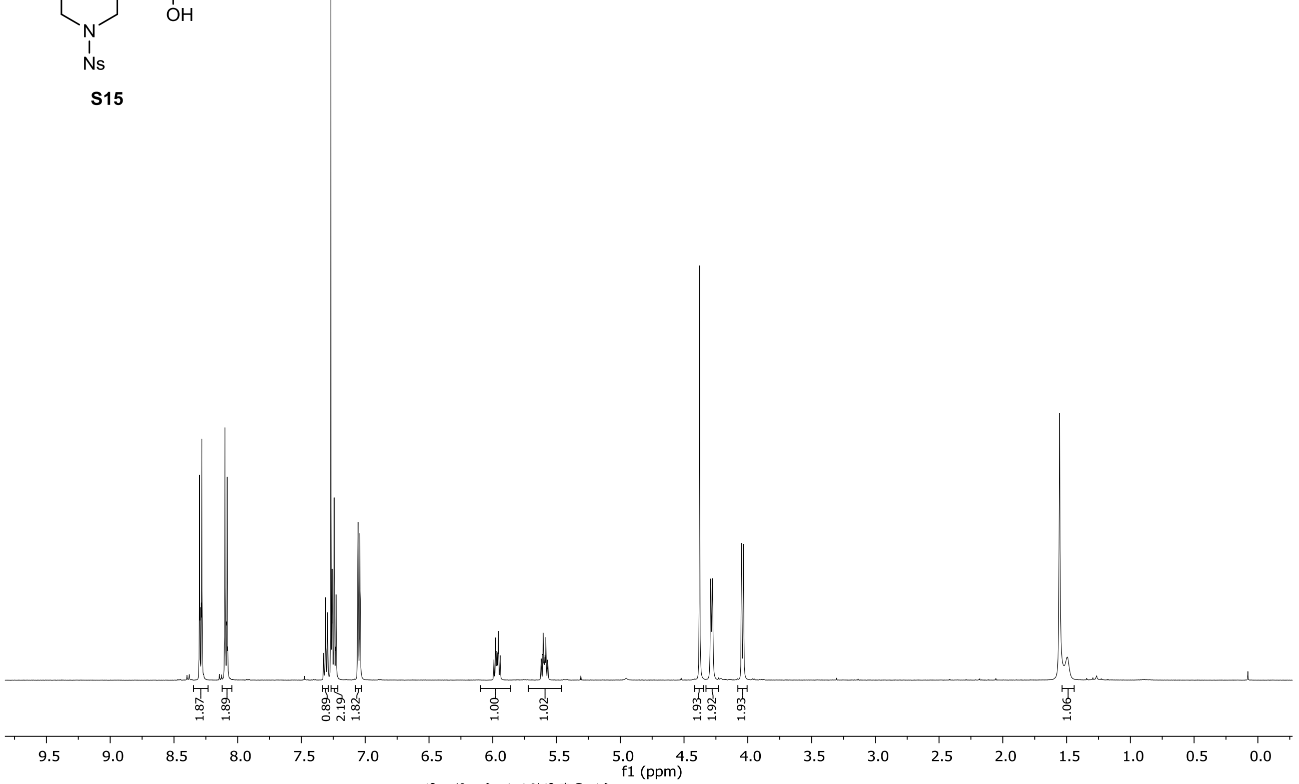
1.87
1.89
0.89
2.19
1.82
1.00
1.02
1.93
1.92
1.93
1.06
9.5 9.0 8.5 8.0 7.5 7.0 6.5 6.0 5.5 5.0 4.5 4.0 3.5 3.0 2.5 2.0 1.5 1.0 0.5 0.0
f1 (ppm)

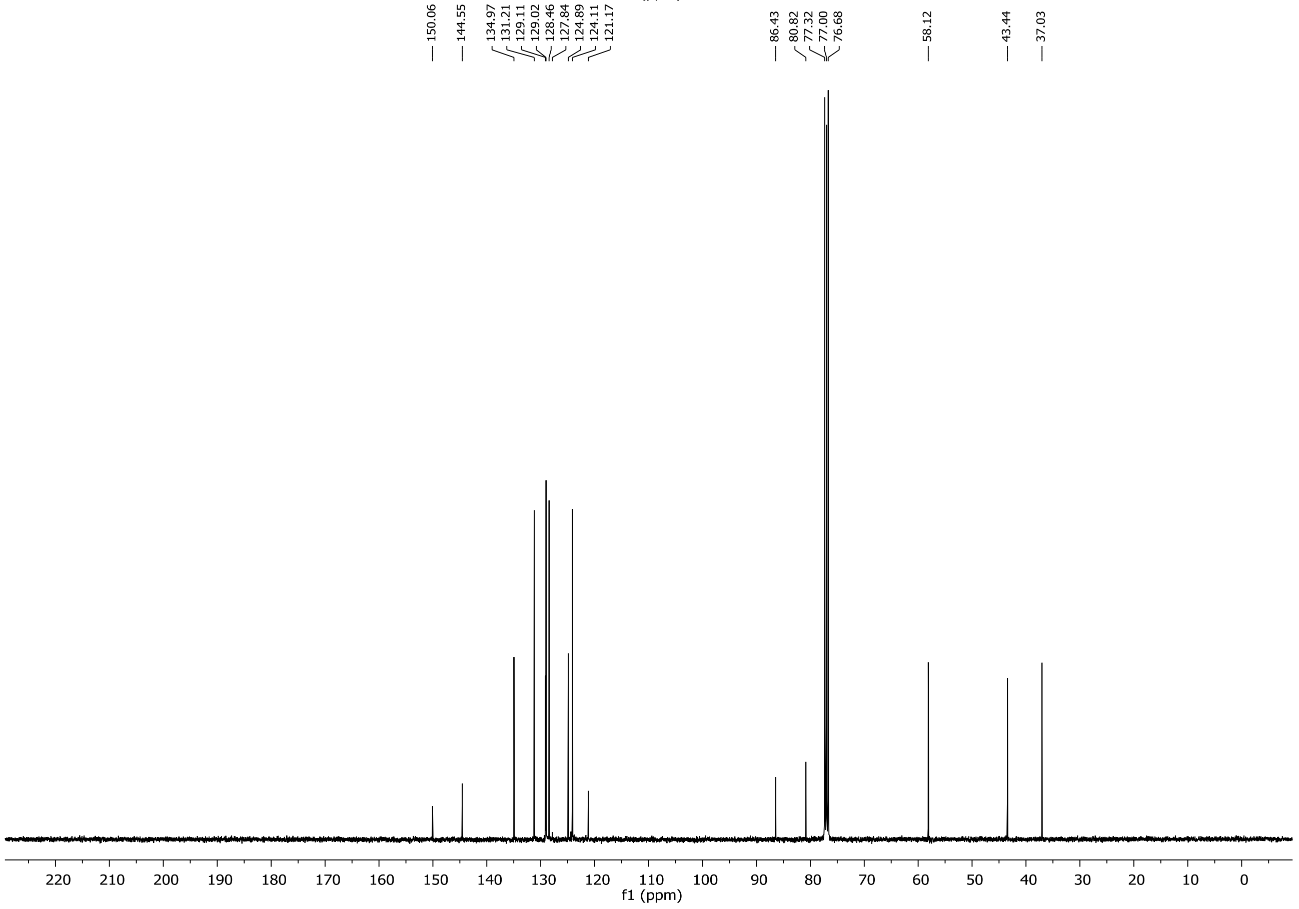
150.06
144.55
134.97
131.21
129.11
129.02
128.46
127.84
124.89
124.11
121.17
86.43
80.82
77.32
77.00
76.68
58.12
43.44
37.03
220 210 200 190 180 170 160 150 140 130 120 110 100 90 80 70 60 50 40 30 20 10 0
f1 (ppm)

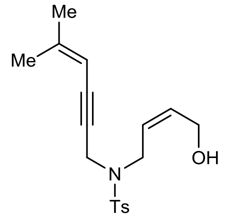
Me
Me
N
Ts
OH

S16

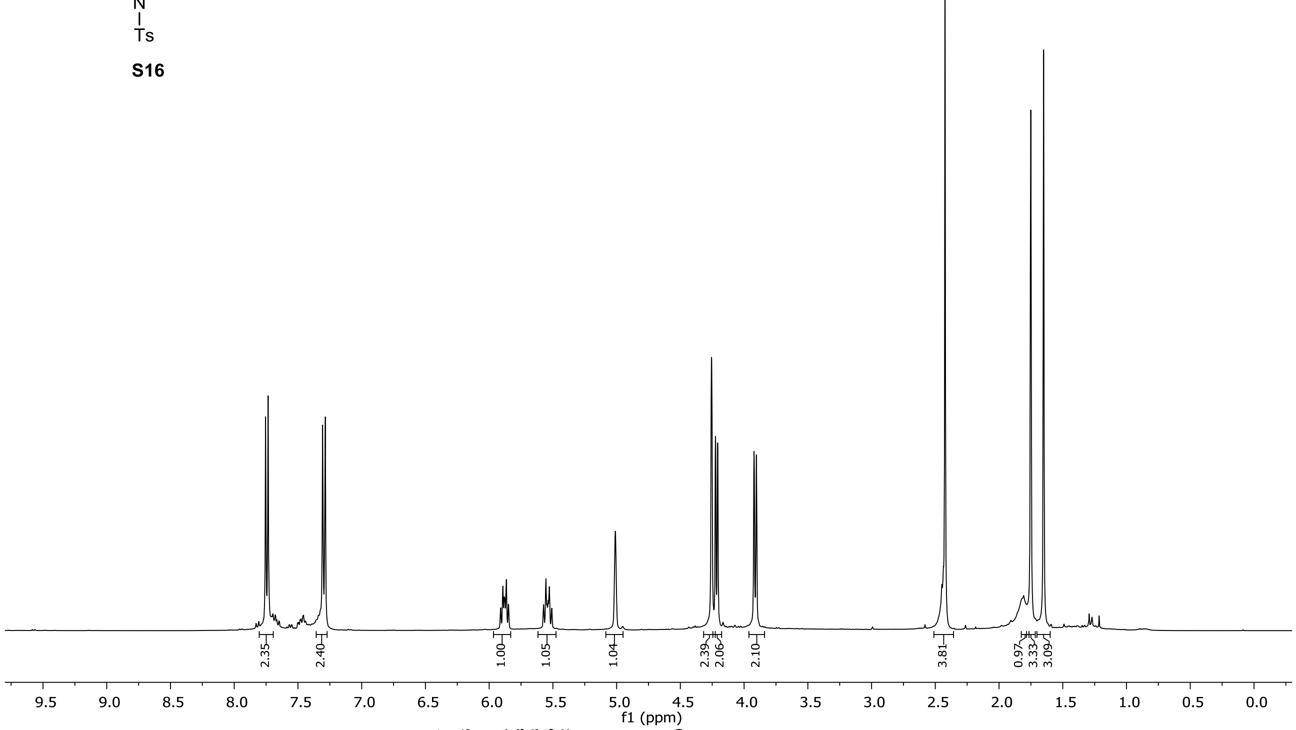
2.35
2.40
1.00
1.05
1.04
2.39
2.06
2.10
3.81
0.97
3.33
3.09
9.5 9.0 8.5 8.0 7.5 7.0 6.5 6.0 5.5 5.0 4.5 4.0 3.5 3.0 2.5 2.0 1.5 1.0 0.5 0.0
f1 (ppm)

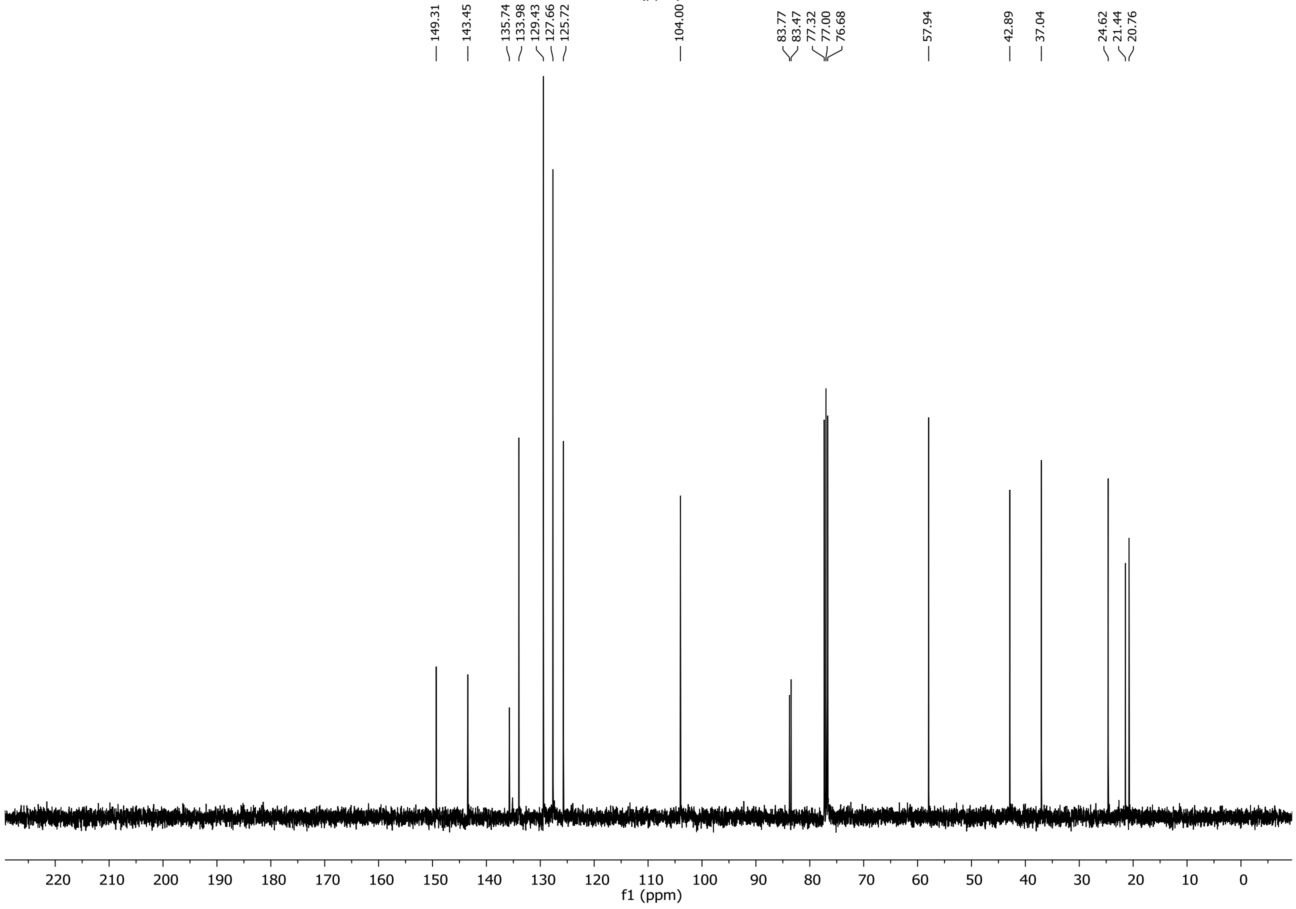
149.31
143.45
135.74
133.98
129.43
127.66
125.72
104.00
83.77
83.47
77.32
77.00
76.68
57.94
42.89
37.04
24.62
21.44
20.76
220 210 200 190 180 170 160 150 140 130 120 110 100 90 80 70 60 50 40 30 20 10 0
f1 (ppm)

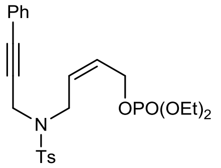
Ph
N
OPO(OEt)$_2$
Ts

1a

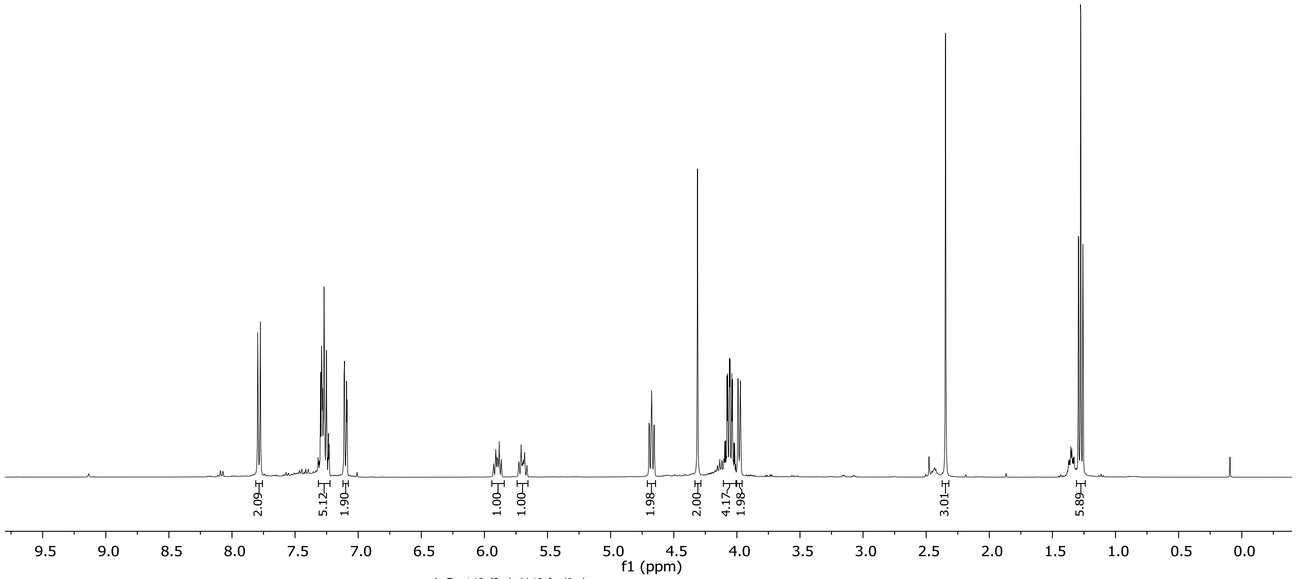
2.09
5.12
1.90
1.00
1.00
1.98
2.00
4.17
1.98
3.01
5.89
9.5 9.0 8.5 8.0 7.5 7.0 6.5 6.0 5.5 5.0 4.5 4.0 3.5 3.0 2.5 2.0 1.5 1.0 0.5 0.0
f1 (ppm)

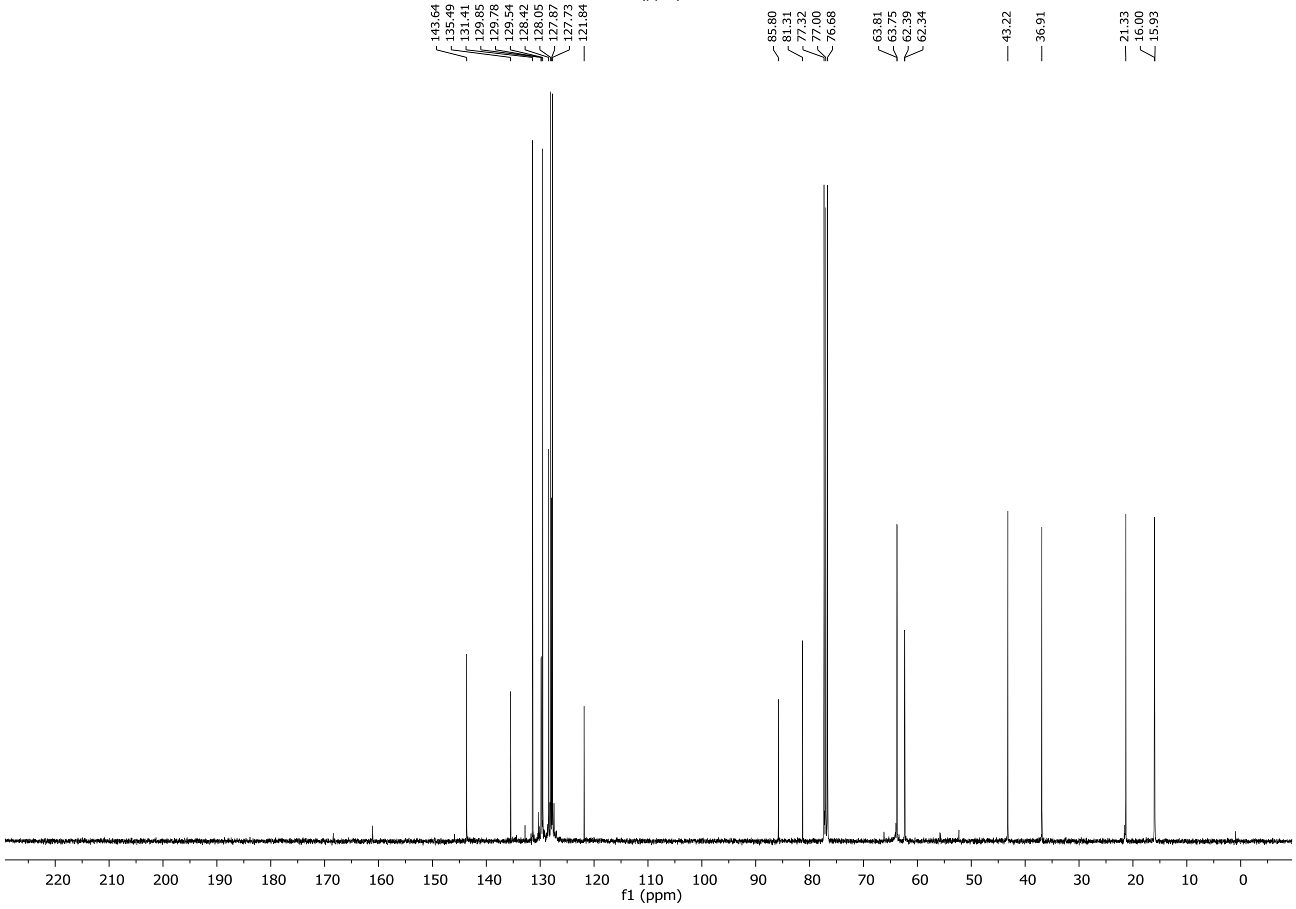
143.64
135.49
131.41
129.85
129.78
129.54
128.42
128.05
127.87
127.73
121.84
85.80
81.31
77.32
77.00
76.68
63.81
63.75
62.39
62.34
43.22
36.91
21.33
16.00
15.93
220 210 200 190 180 170 160 150 140 130 120 110 100 90 80 70 60 50 40 30 20 10 0
f1 (ppm)

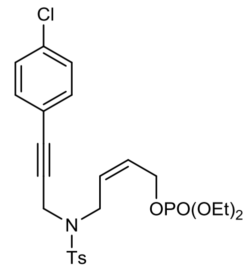
Cl
N
Ts
OPO(OEt)2

1b

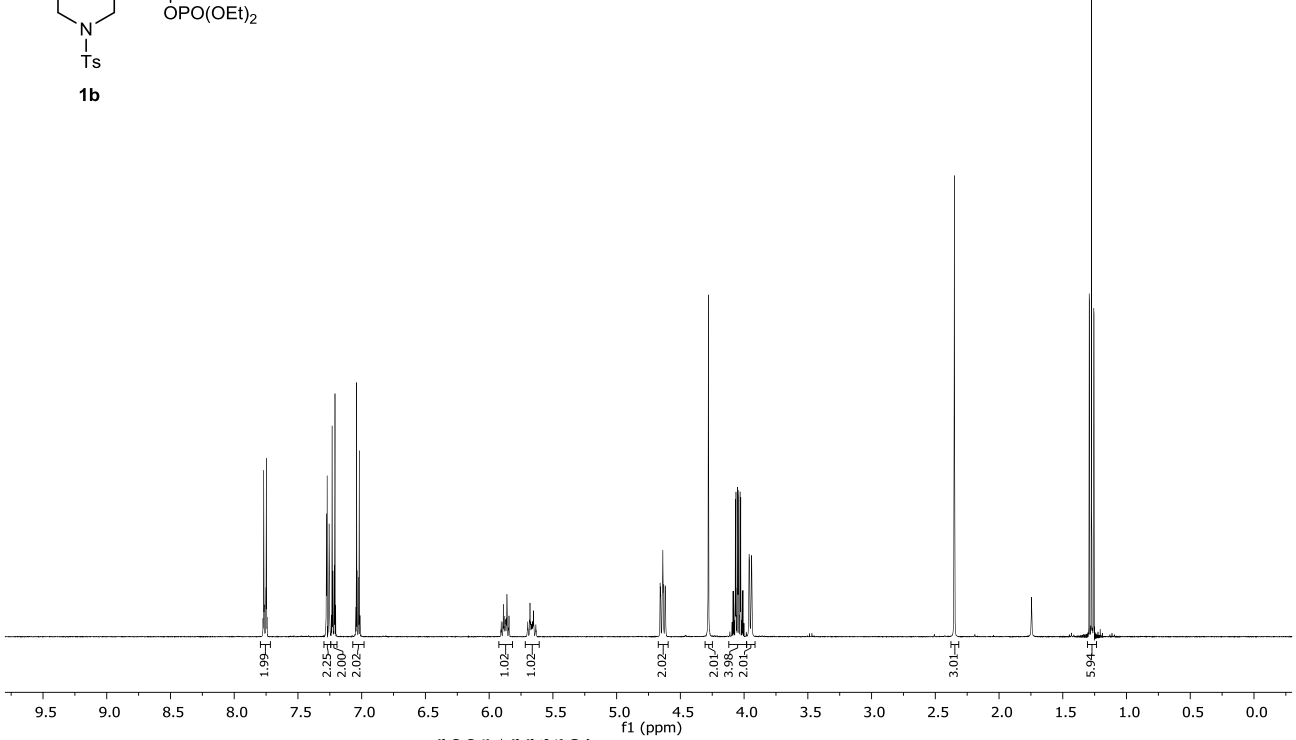
1.99
2.25
2.00
2.02
1.02
1.02
2.02
2.01
3.98
2.01
3.01
5.94
9.5 9.0 8.5 8.0 7.5 7.0 6.5 6.0 5.5 5.0 4.5 4.0 3.5 3.0 2.5 2.0 1.5 1.0 0.5 0.0
f1 (ppm)

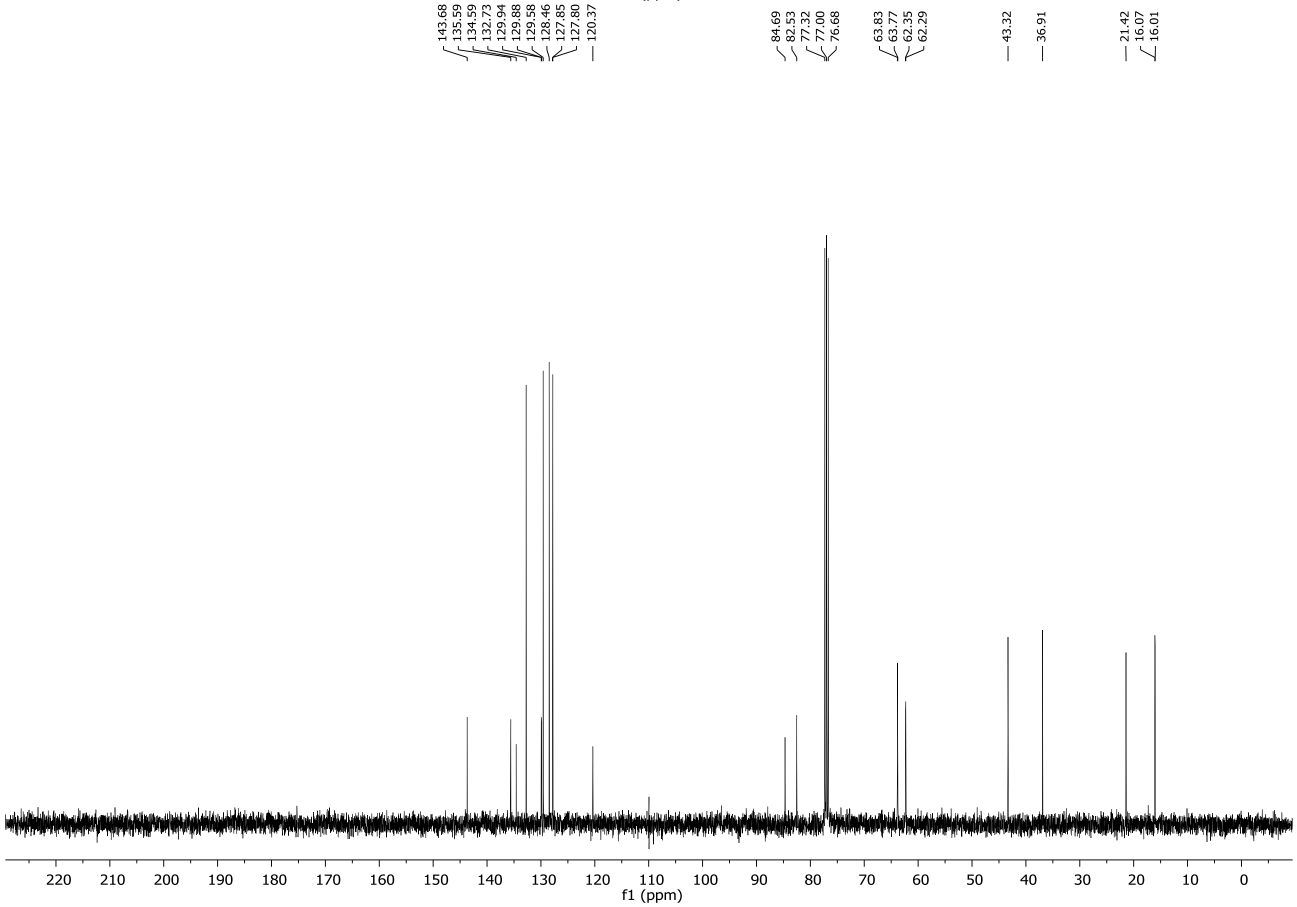
143.68
135.59
134.59
132.73
129.94
129.88
129.58
128.46
127.85
127.80
120.37
84.69
82.53
77.32
77.00
76.68
63.83
63.77
62.35
62.29
43.32
36.91
21.42
16.07
16.01
220 210 200 190 180 170 160 150 140 130 120 110 100 90 80 70 60 50 40 30 20 10 0
f1 (ppm)

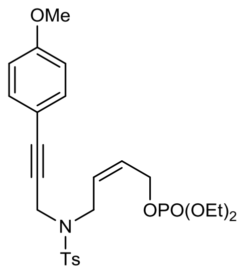
OMe
N
Ts
OPO(OEt)2

1c

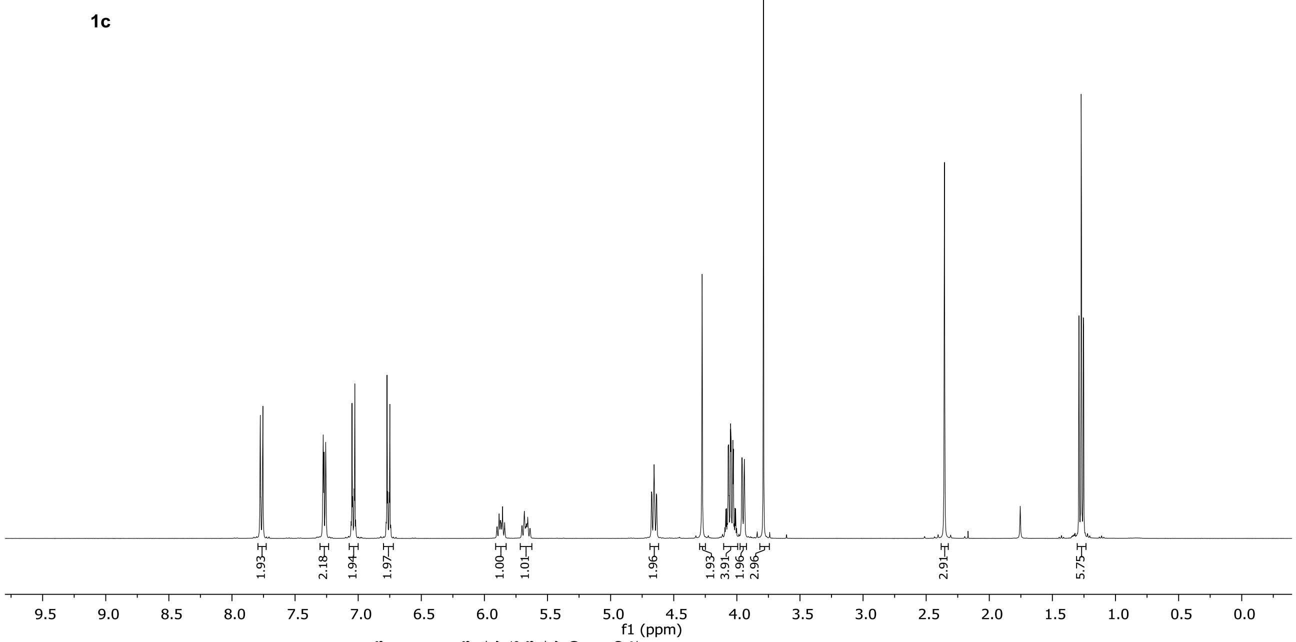
1.93
2.18
1.94
1.97
1.00
1.01
1.96
1.93
3.91
1.96
2.96
2.91
5.75
9.5 9.0 8.5 8.0 7.5 7.0 6.5 6.0 5.5 5.0 4.5 4.0 3.5 3.0 2.5 2.0 1.5 1.0 0.5 0.0
f1 (ppm)

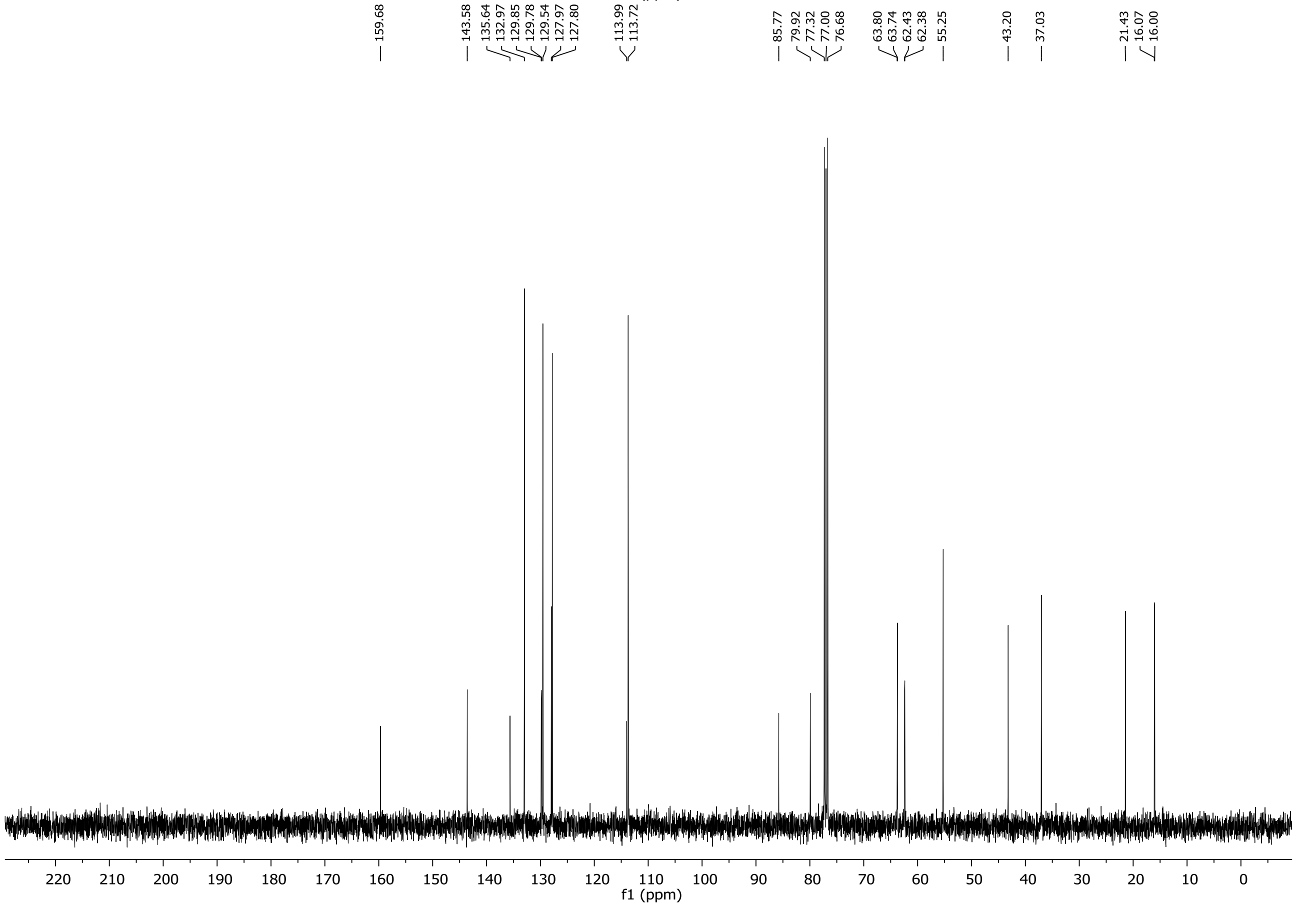
159.68
143.58
135.64
132.97
129.85
129.78
129.54
127.97
127.80
113.99
113.72
85.77
79.92
77.32
77.00
76.68
63.80
63.74
62.43
62.38
55.25
43.20
37.03
21.43
16.07
16.00
220 210 200 190 180 170 160 150 140 130 120 110 100 90 80 70 60 50 40 30 20 10 0
f1 (ppm)

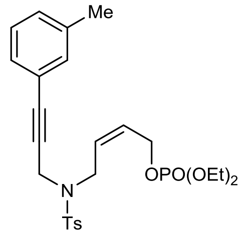
Me
N
Ts
OPO(OEt)2

1d

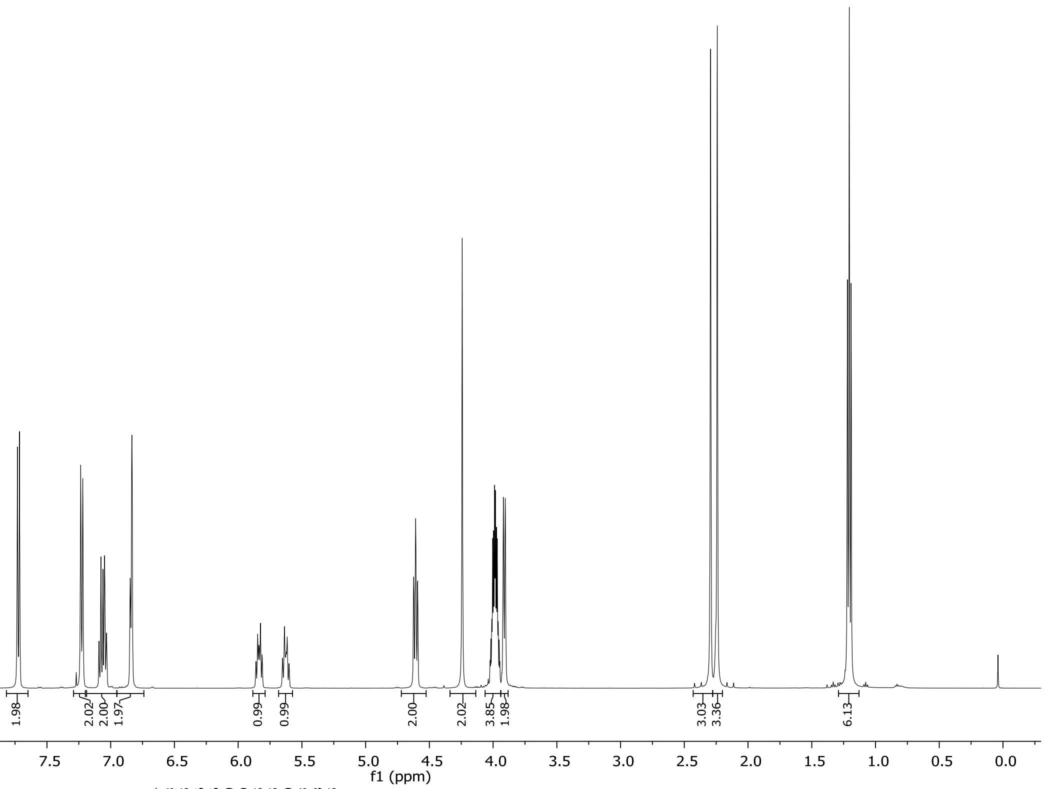
1.98
2.02
2.00
1.97
0.99
0.99
2.00
2.02
3.85
1.98
3.03
3.36
6.13
9.5 9.0 8.5 8.0 7.5 7.0 6.5 6.0 5.5 5.0 4.5 4.0 3.5 3.0 2.5 2.0 1.5 1.0 0.5 0.0
f1 (ppm)

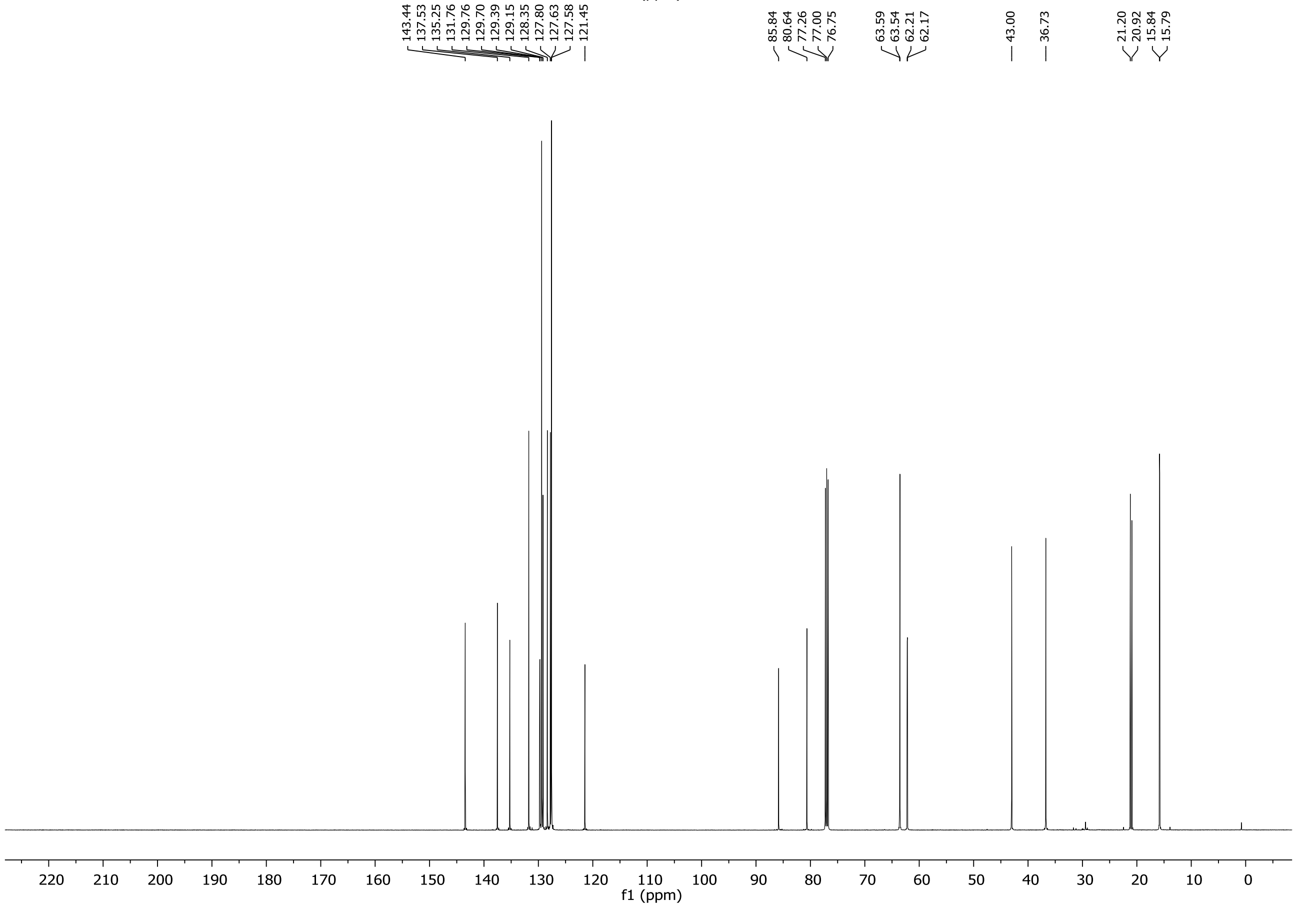
143.44
137.53
135.25
131.76
129.76
129.70
129.39
129.15
128.35
127.80
127.63
127.58
121.45
85.84
80.64
77.26
77.00
76.75
63.59
63.54
62.21
62.17
43.00
36.73
21.20
20.92
15.84
15.79
220 210 200 190 180 170 160 150 140 130 120 110 100 90 80 70 60 50 40 30 20 10 0
f1 (ppm)

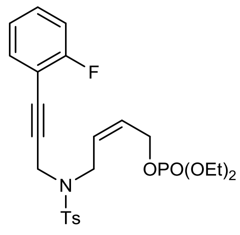
F
N
Ts
OPO(OEt)$_2$

1e

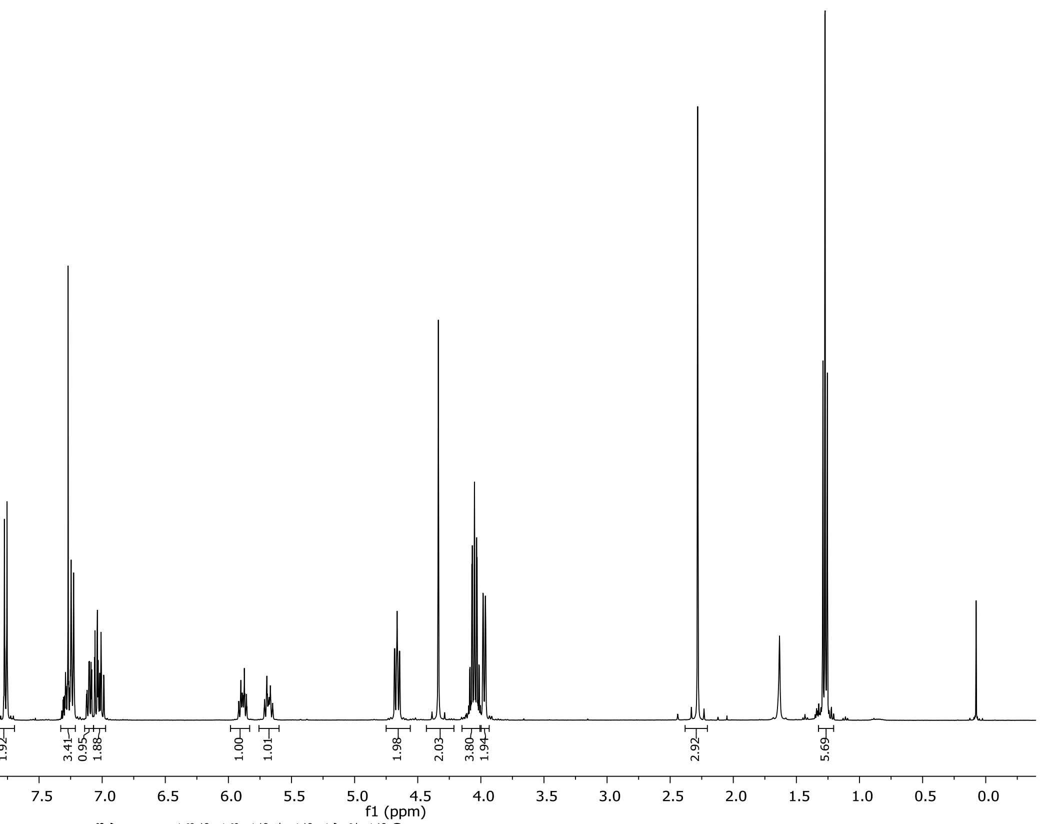
1.92
3.41
0.95
1.88
1.00
1.01
1.98
2.03
3.80
1.94
2.92
5.69
9.5 9.0 8.5 8.0 7.5 7.0 6.5 6.0 5.5 5.0 4.5 4.0 3.5 3.0 2.5 2.0 1.5 1.0 0.5 0.0
f1 (ppm)

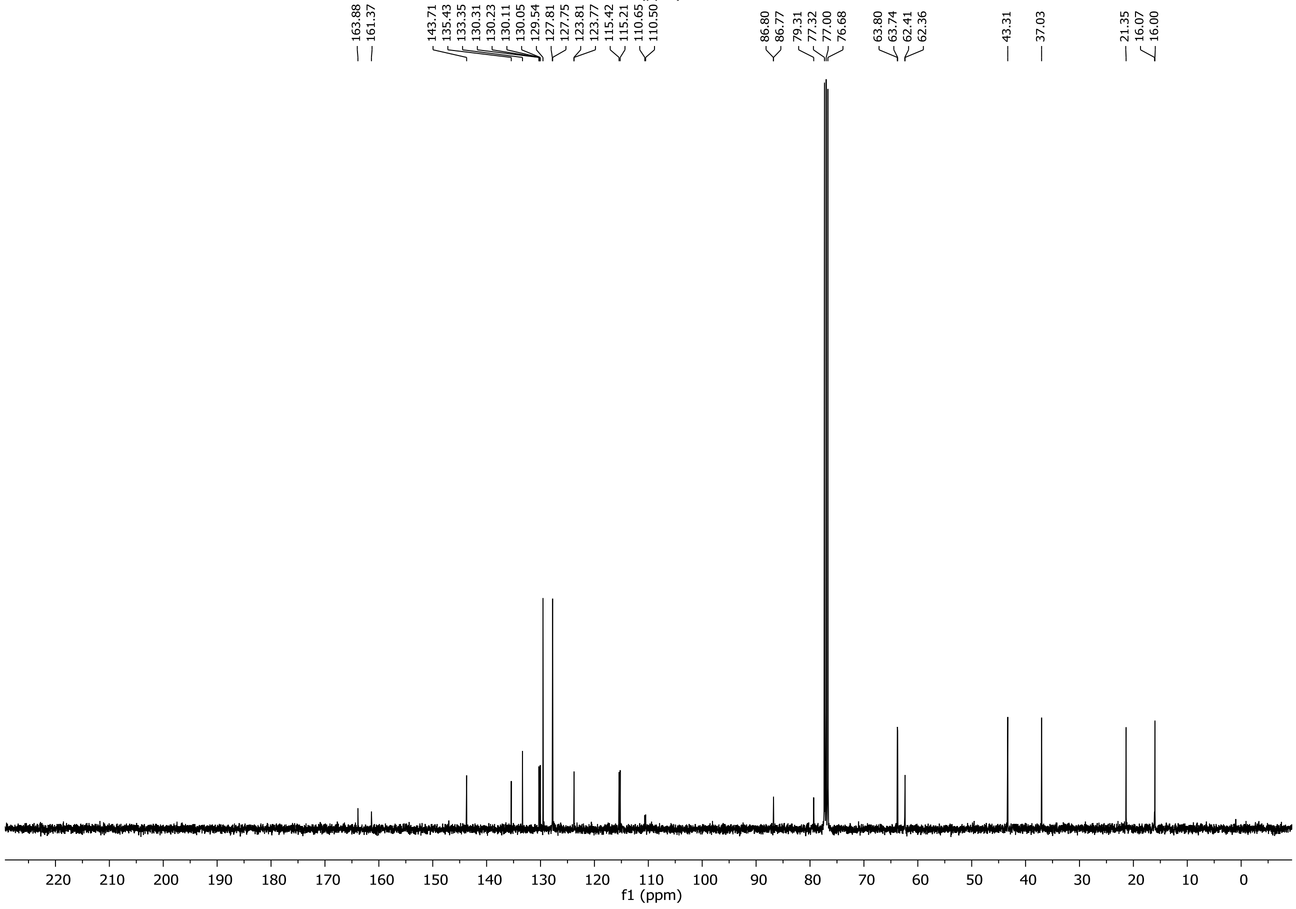
163.88
161.37
143.71
135.43
133.35
130.31
130.23
130.11
130.05
129.54
127.81
127.75
123.81
123.77
115.42
115.21
110.65
110.50
86.80
86.77
79.31
77.32
77.00
76.68
63.80
63.74
62.41
62.36
43.31
37.03
21.35
16.07
16.00
220 210 200 190 180 170 160 150 140 130 120 110 100 90 80 70 60 50 40 30 20 10 0
f1 (ppm)

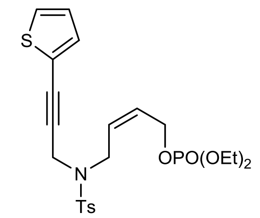
S
N
Ts
OPO(OEt)2

1f

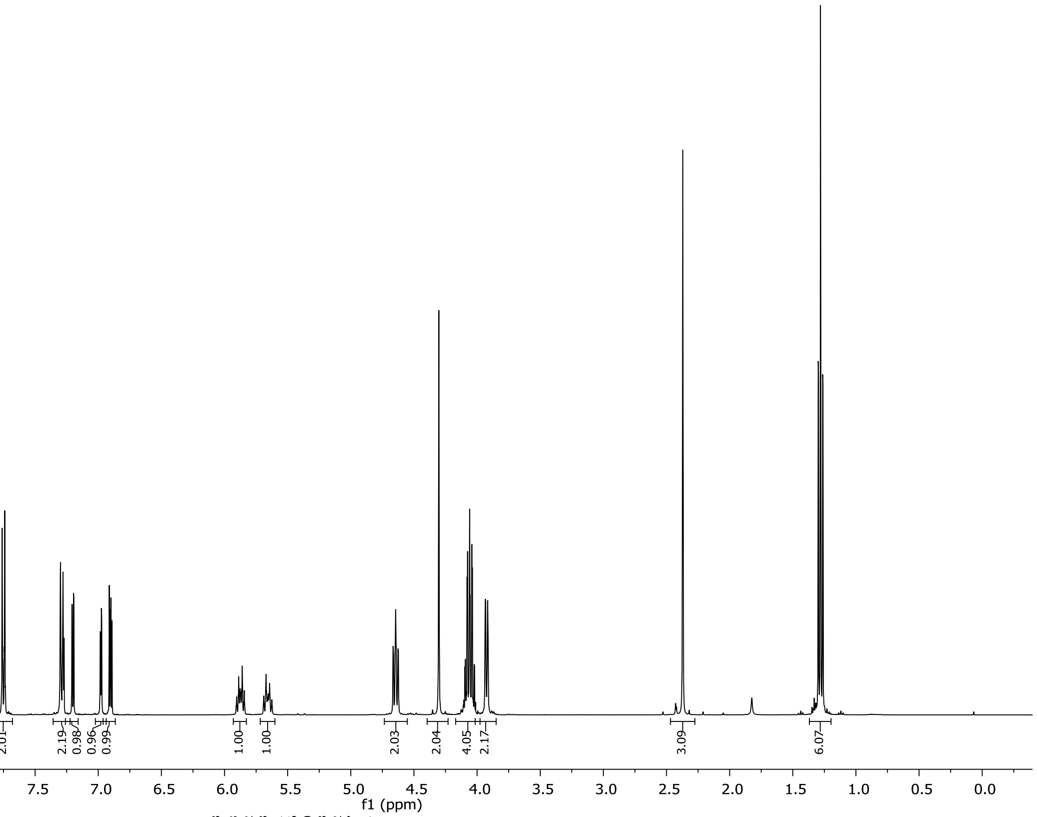
2.01
2.19
0.98
0.96
0.99
1.00
1.00
2.03
2.04
4.05
2.17
3.09
6.07
9.5 9.0 8.5 8.0 7.5 7.0 6.5 6.0 5.5 5.0 4.5 4.0 3.5 3.0 2.5 2.0 1.5 1.0 0.5 0.0
f1 (ppm)

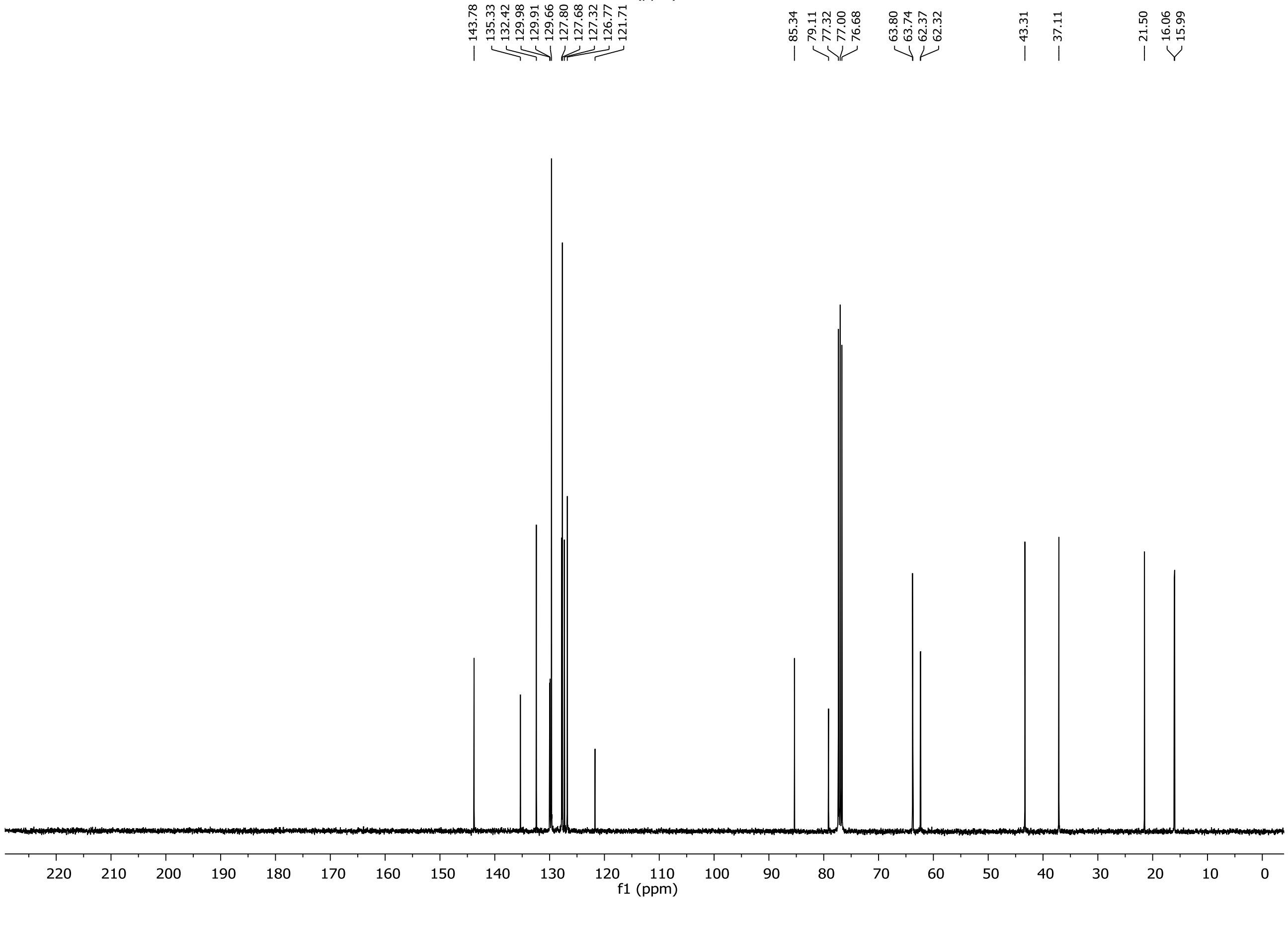
143.78
135.33
132.42
129.98
129.91
129.66
127.80
127.68
127.32
126.77
121.71
85.34
79.11
77.32
77.00
76.68
63.80
63.74
62.37
62.32
43.31
37.11
21.50
16.06
15.99
220 210 200 190 180 170 160 150 140 130 120 110 100 90 80 70 60 50 40 30 20 10 0
f1 (ppm)

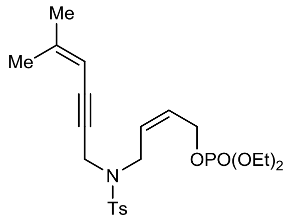
Me
Me
N
Ts
OPO(OEt)2

1g

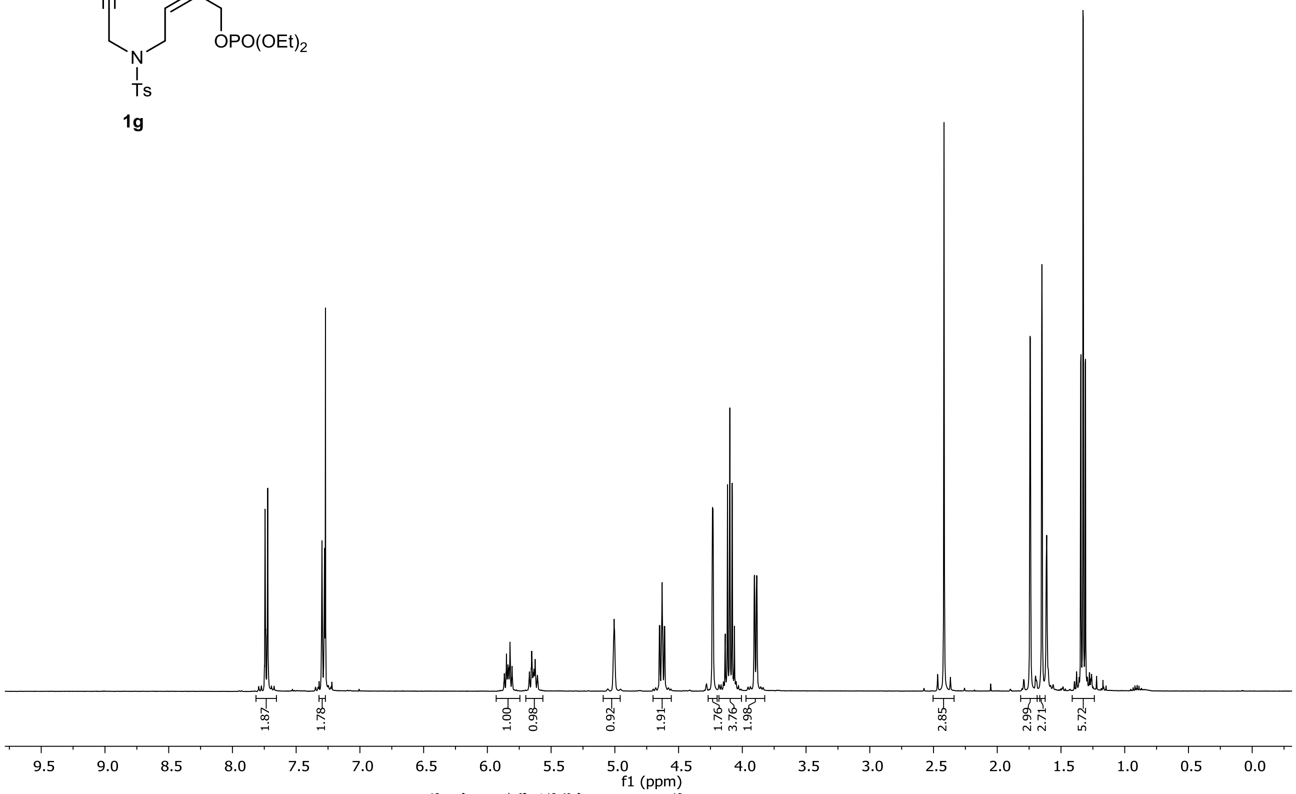
1.87
1.78
1.00
0.98
0.92
1.91
1.76
3.76
1.98
2.85
2.99
2.71
5.72
9.5 9.0 8.5 8.0 7.5 7.0 6.5 6.0 5.5 5.0 4.5 4.0 3.5 3.0 2.5 2.0 1.5 1.0 0.5 0.0
f1 (ppm)

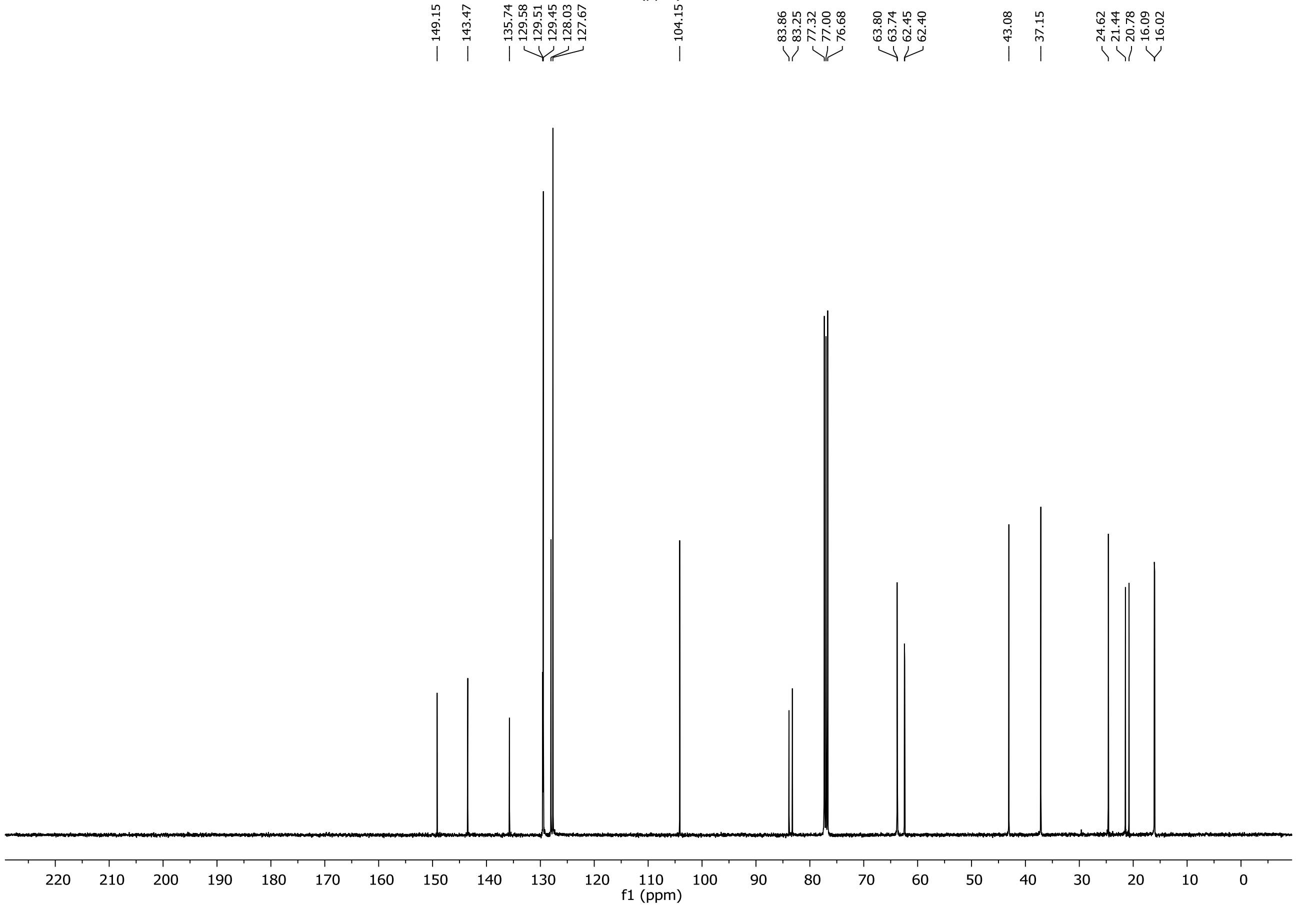
149.15
143.47
135.74
129.58
129.51
129.45
128.03
127.67
104.15
83.86
83.25
77.32
77.00
76.68
63.80
63.74
62.45
62.40
43.08
37.15
24.62
21.44
20.78
16.09
16.02
220 210 200 190 180 170 160 150 140 130 120 110 100 90 80 70 60 50 40 30 20 10 0
f1 (ppm)

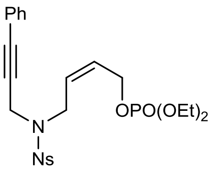
Ph
N
Ns
OPO(OEt)$_2$

1h

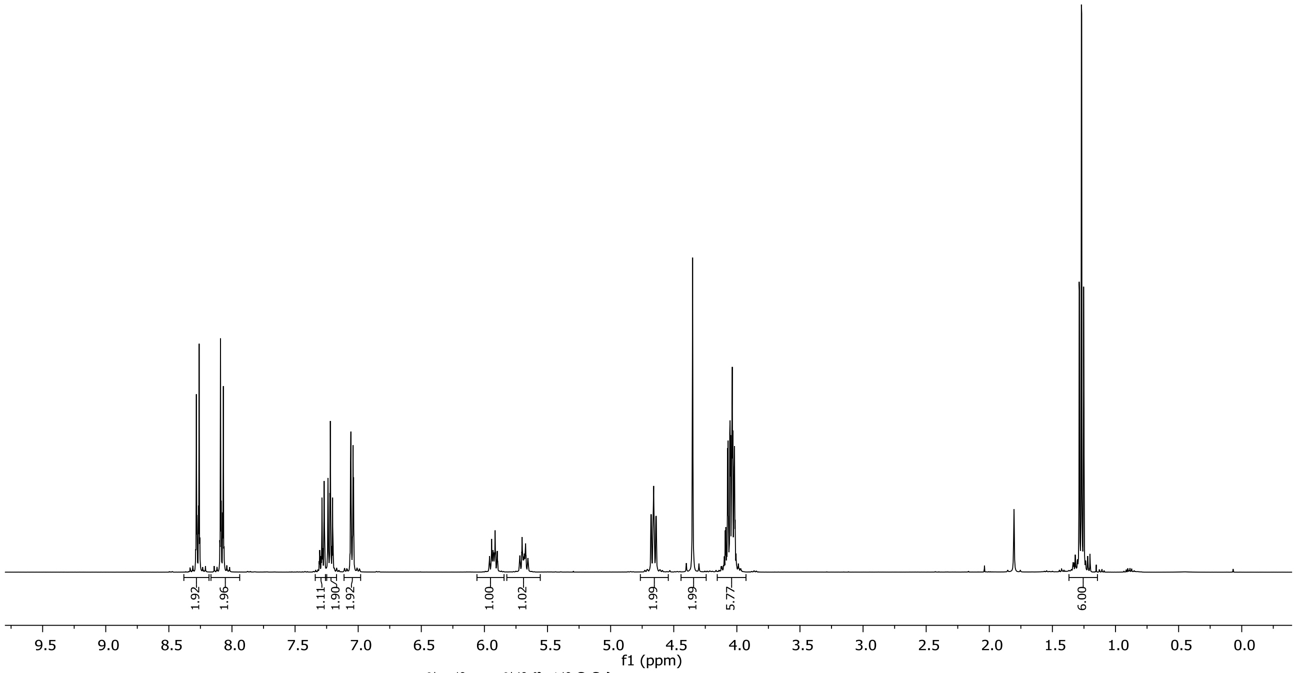
1.92
1.96
1.11
1.90
1.92
1.00
1.02
1.99
1.99
5.77
6.00
f1 (ppm)

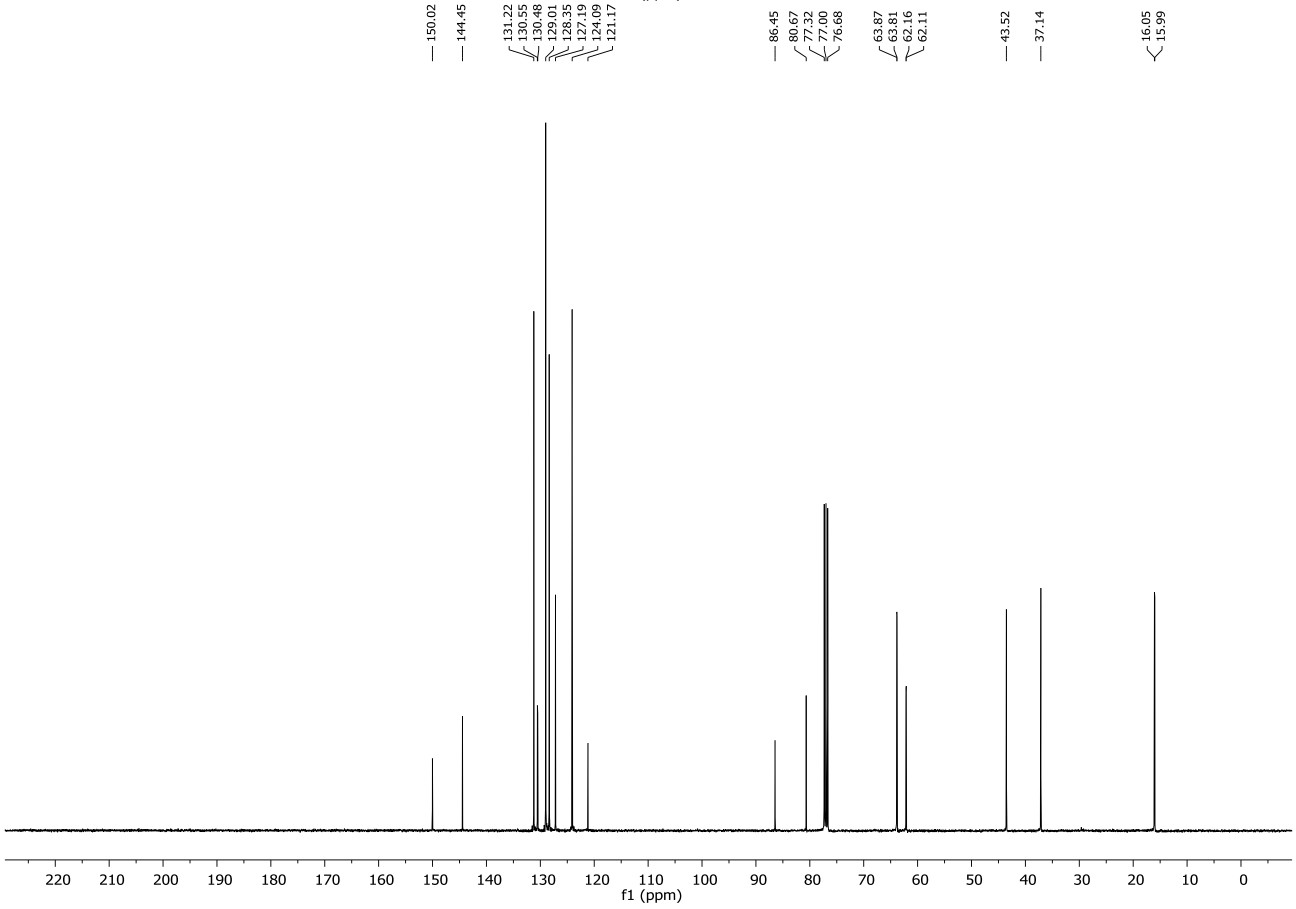
150.02
144.45
131.22
130.55
130.48
129.01
128.35
127.19
124.09
121.17
86.45
80.67
77.32
77.00
76.68
63.87
63.81
62.16
62.11
43.52
37.14
16.05
15.99
f1 (ppm)

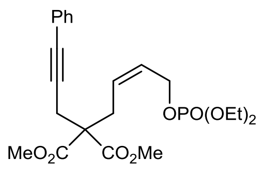
Ph
OPO(OEt)2
MeO2C
CO2Me

1i

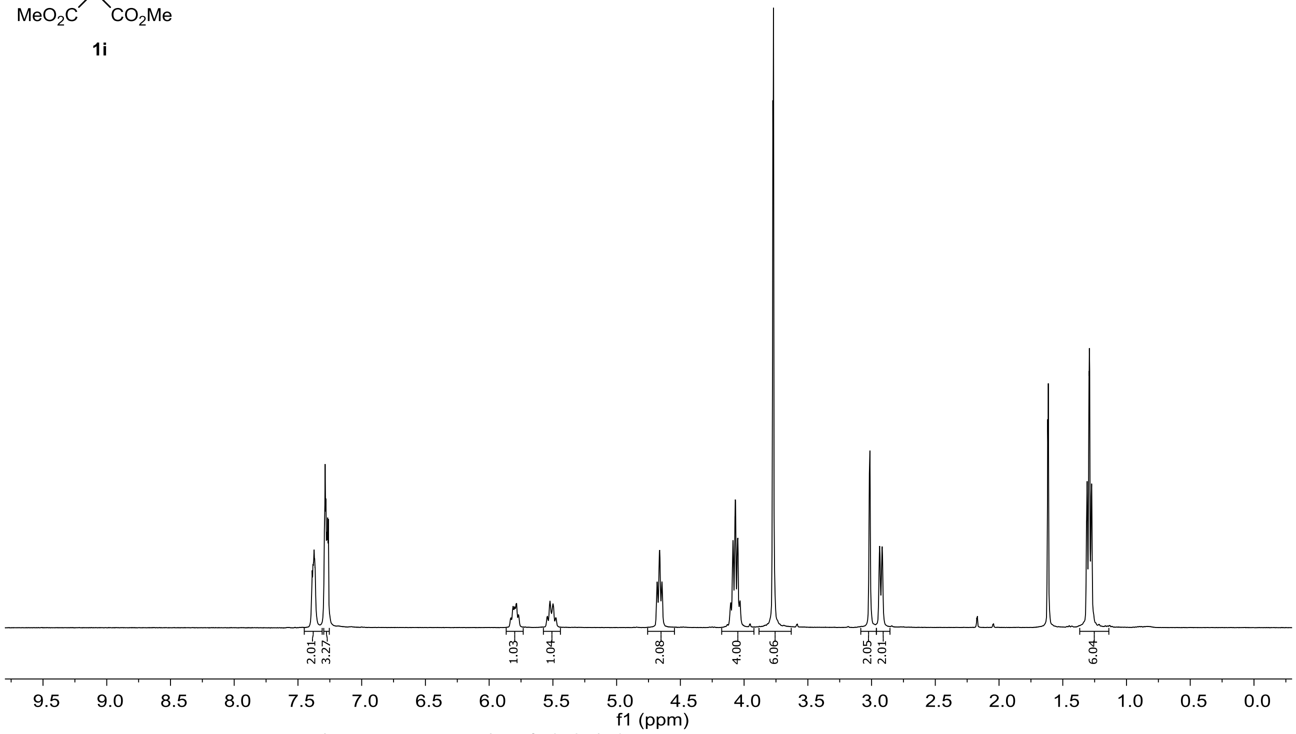
2.01
3.27
1.03
1.04
2.08
4.00
6.06
2.05
2.01
6.04
9.5 9.0 8.5 8.0 7.5 7.0 6.5 6.0 5.5 5.0 4.5 4.0 3.5 3.0 2.5 2.0 1.5 1.0 0.5 0.0
f1 (ppm)

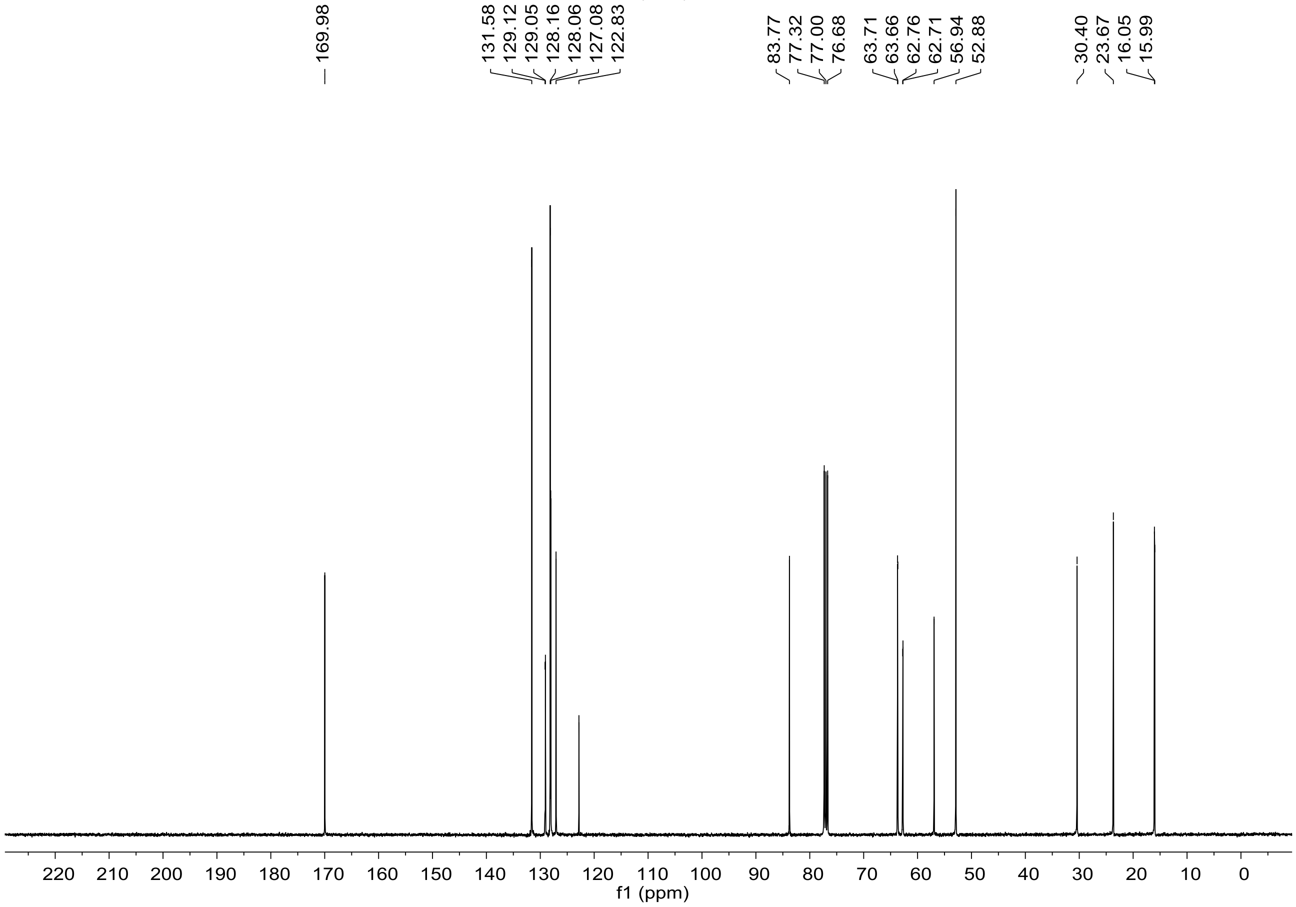
169.98
131.58
129.12
129.05
128.16
128.06
127.08
122.83
83.77
77.32
77.00
76.68
63.71
63.66
62.76
62.71
56.94
52.88
30.40
23.67
16.05
15.99
220 210 200 190 180 170 160 150 140 130 120 110 100 90 80 70 60 50 40 30 20 10 0
f1 (ppm)

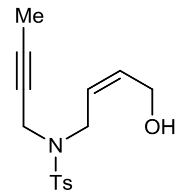
Me
N
Ts
OH

S18

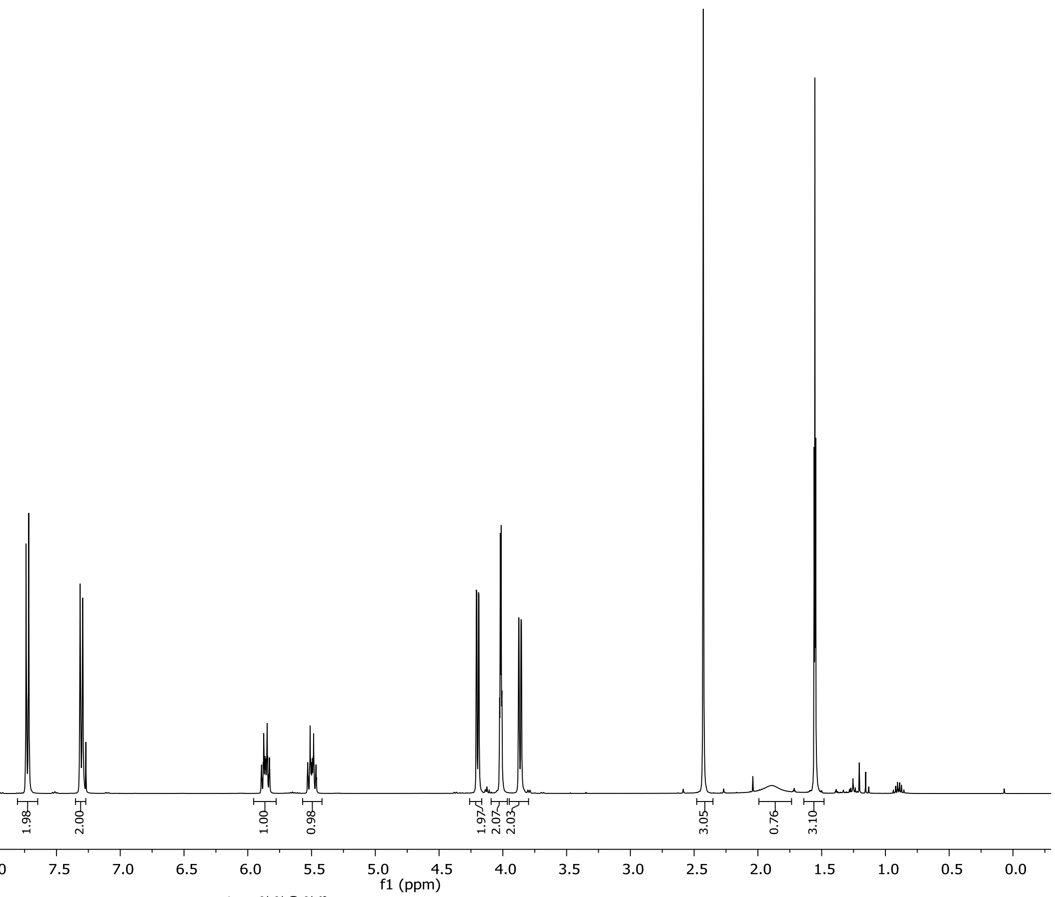
1.98
2.00
1.00
0.98
1.97
2.07
2.03
3.05
0.76
3.10
9.5 9.0 8.5 8.0 7.5 7.0 6.5 6.0 5.5 5.0 4.5 4.0 3.5 3.0 2.5 2.0 1.5 1.0 0.5 0.0
f1 (ppm)

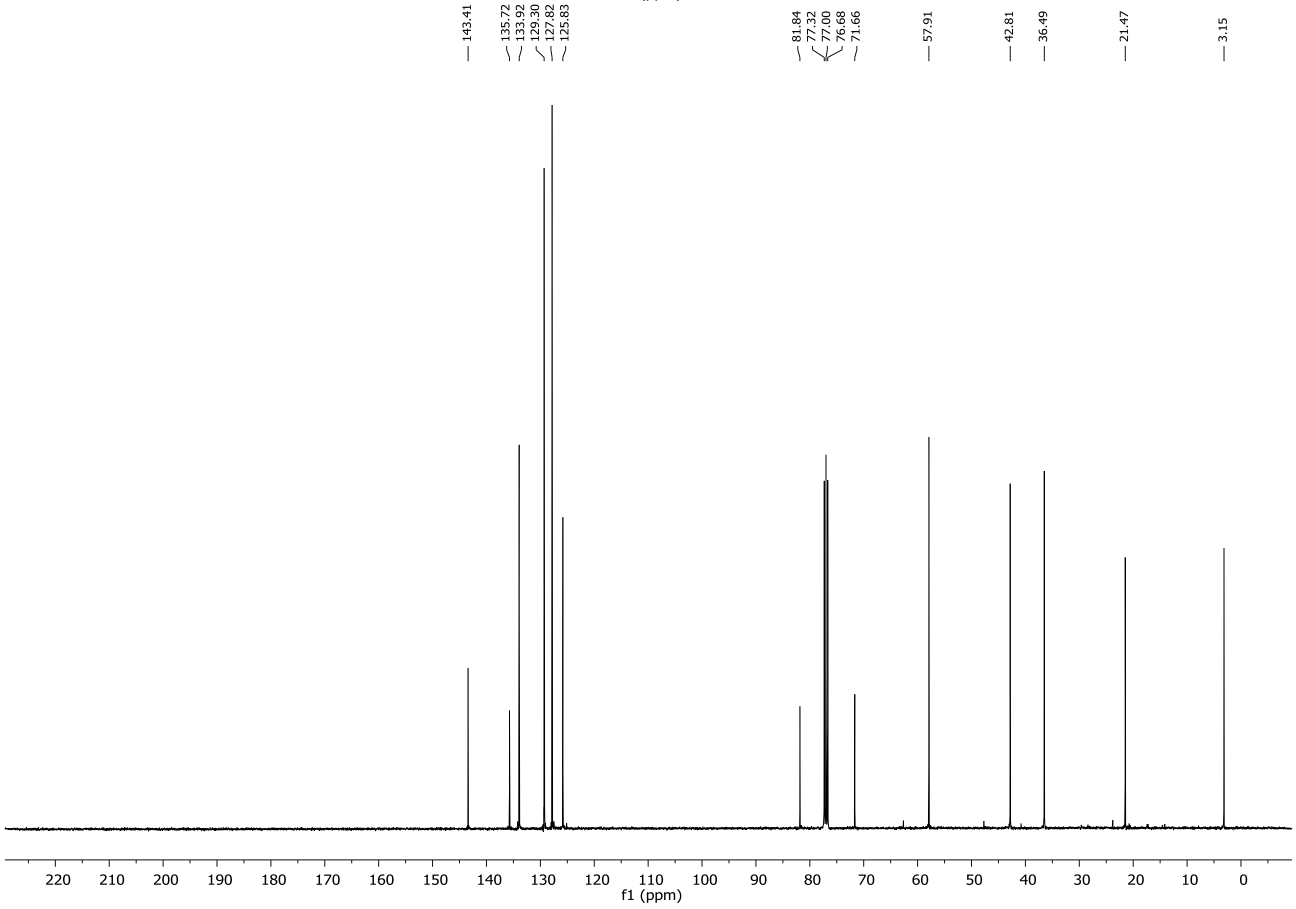
143.41
135.72
133.92
129.30
127.82
125.83
81.84
77.32
77.00
76.68
71.66
57.91
42.81
36.49
21.47
3.15
220 210 200 190 180 170 160 150 140 130 120 110 100 90 80 70 60 50 40 30 20 10 0
f1 (ppm)

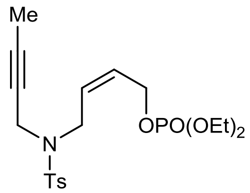
Me
N
Ts
OPO(OEt)2

S19

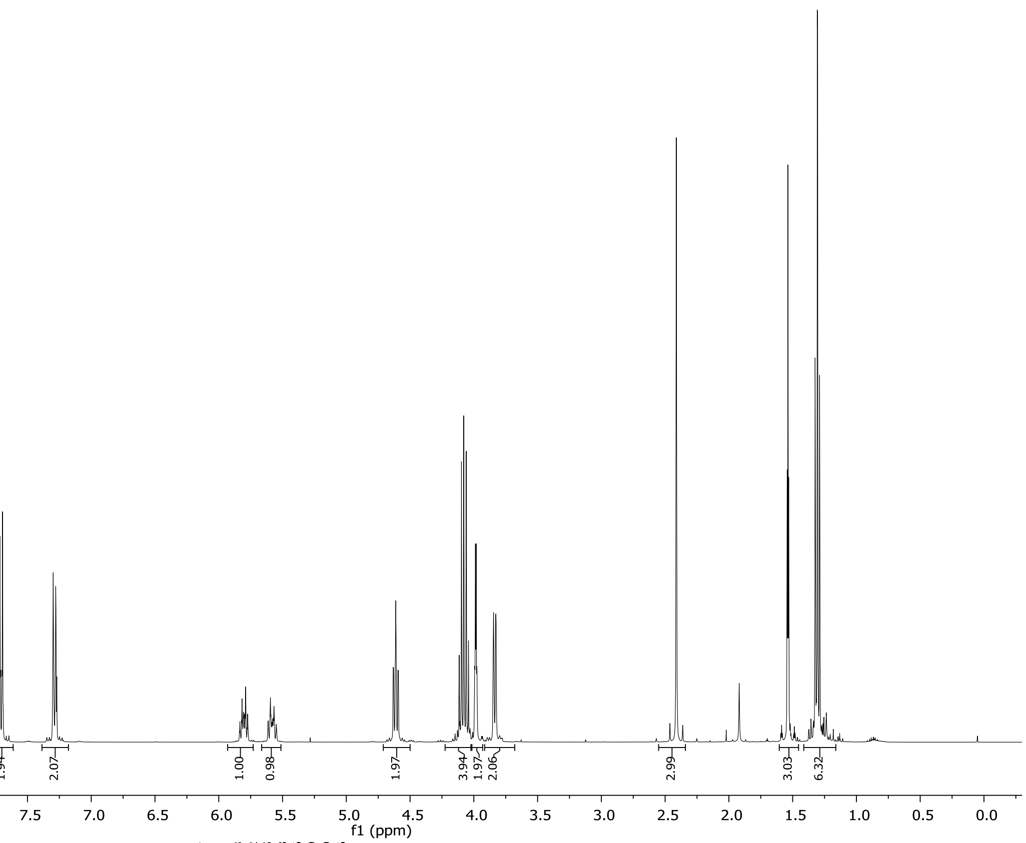
1.94
2.07
1.00
0.98
1.97
3.94
1.97
2.06
2.99
3.03
6.32
9.5
9.0
8.5
8.0
7.5
7.0
6.5
6.0
5.5
5.0
4.5
4.0
3.5
3.0
2.5
2.0
1.5
1.0
0.5
0.0
f1 (ppm)

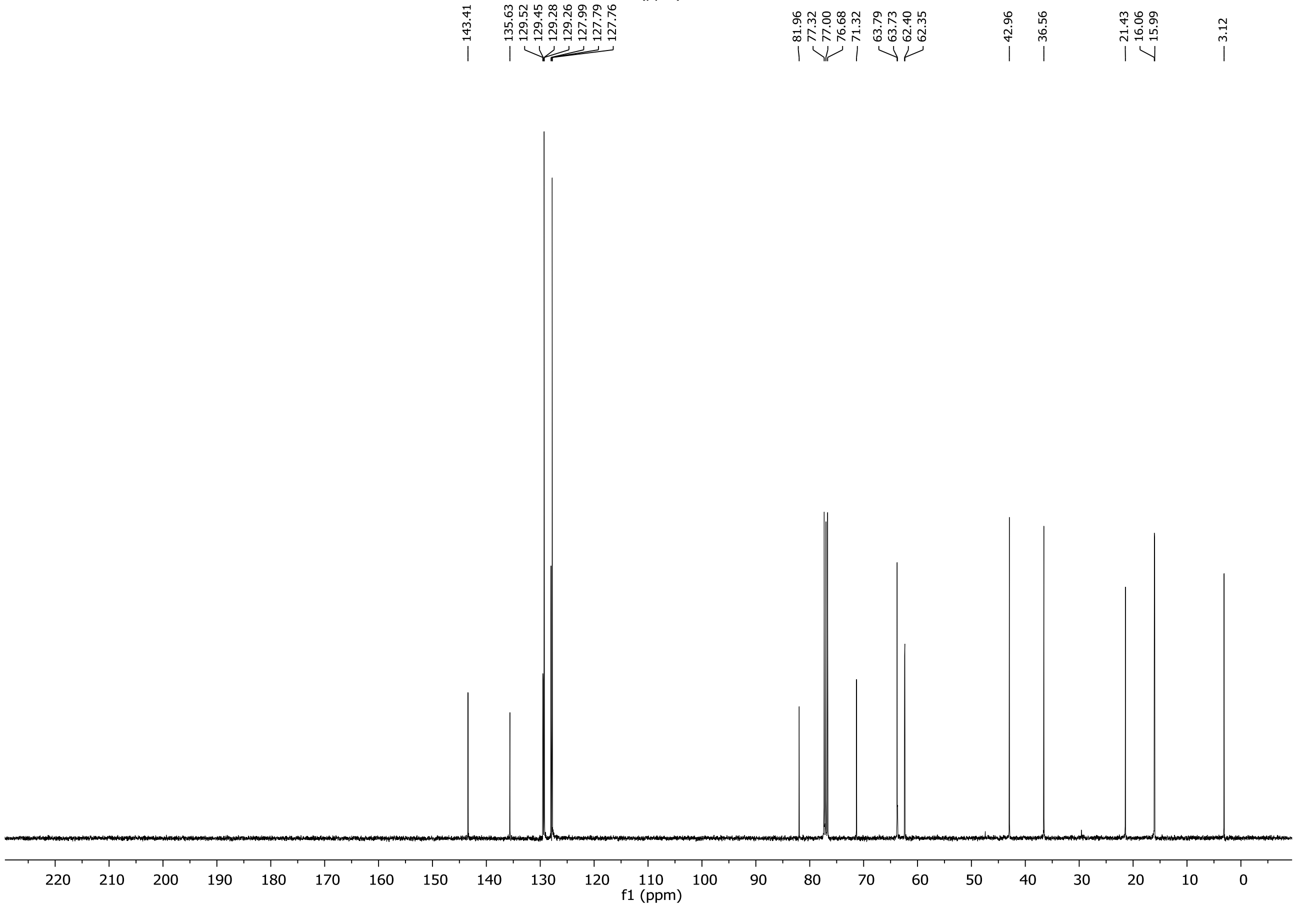
143.41
135.63
129.52
129.45
129.28
129.26
127.99
127.79
127.76
81.96
77.32
77.00
76.68
71.32
63.79
63.73
62.40
62.35
42.96
36.56
21.43
16.06
15.99
3.12
220
210
200
190
180
170
160
150
140
130
120
110
100
90
80
70
60
50
40
30
20
10
0
f1 (ppm)

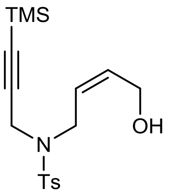
TMS
N
Ts
OH

S20

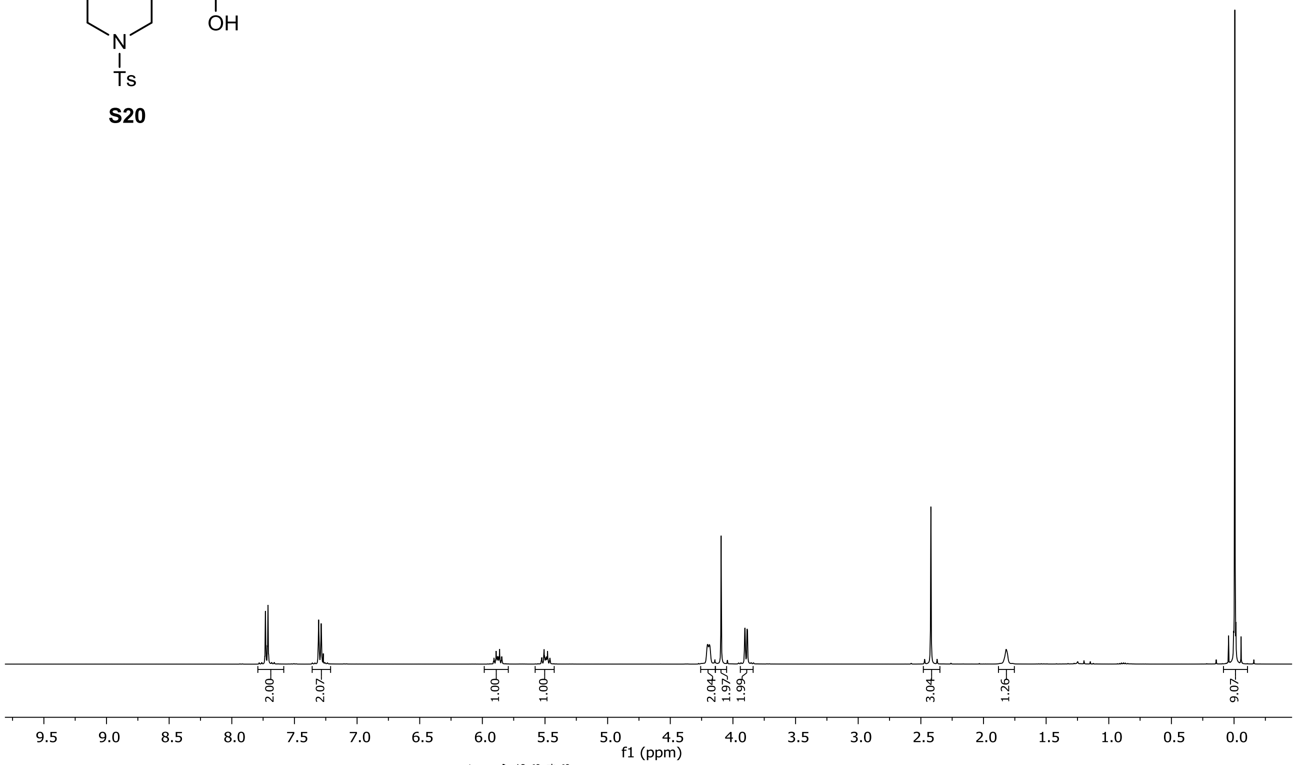
2.00
2.07
1.00
1.00
2.04
1.97
1.99
3.04
1.26
9.07
9.5 9.0 8.5 8.0 7.5 7.0 6.5 6.0 5.5 5.0 4.5 4.0 3.5 3.0 2.5 2.0 1.5 1.0 0.5 0.0
f1 (ppm)

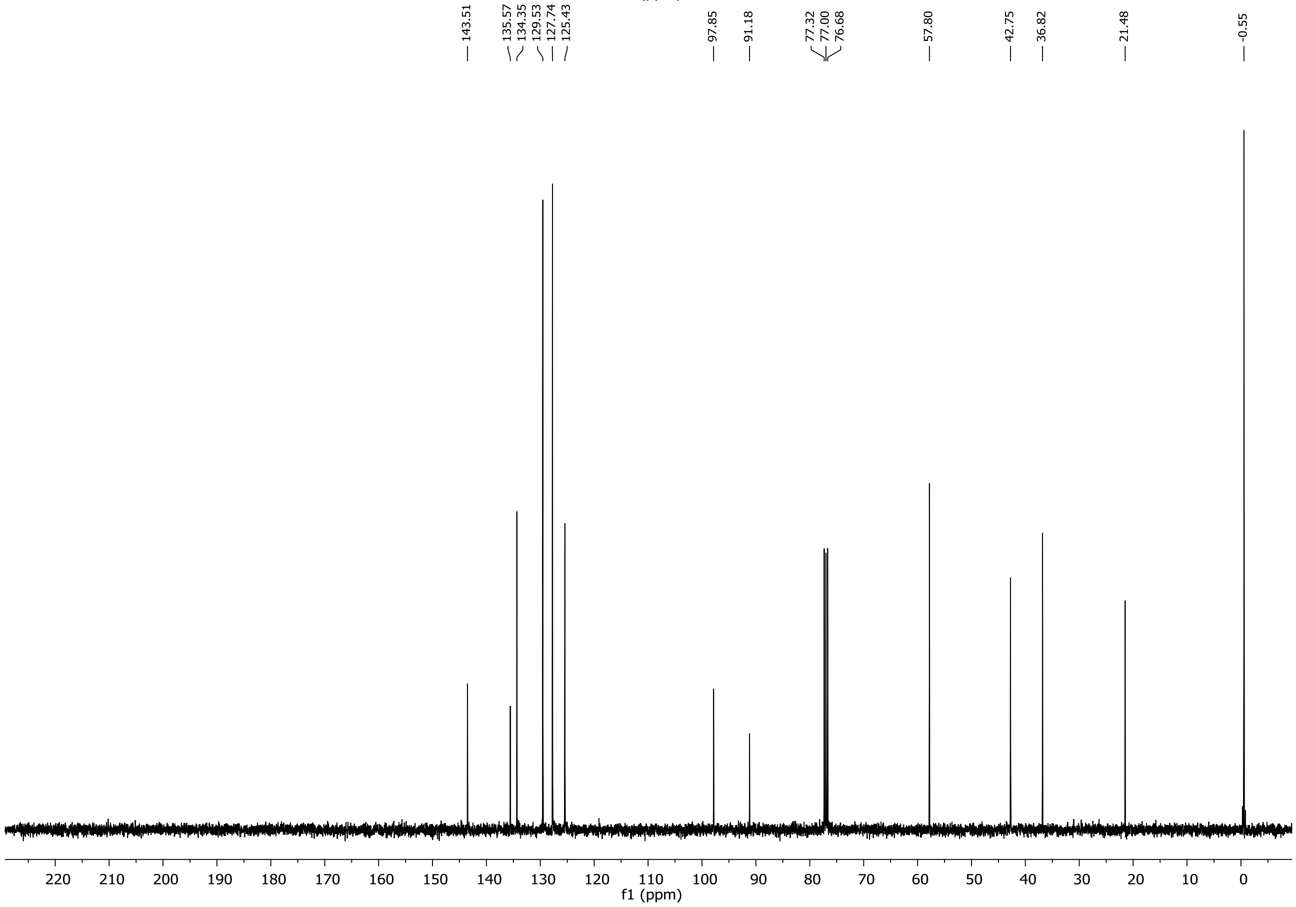
143.51
135.57
134.35
129.53
127.74
125.43
97.85
91.18
77.32
77.00
76.68
57.80
42.75
36.82
21.48
-0.55
220 210 200 190 180 170 160 150 140 130 120 110 100 90 80 70 60 50 40 30 20 10 0
f1 (ppm)

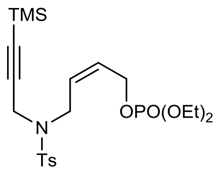
TMS
N
Ts
OPO(OEt)$_2$

1j

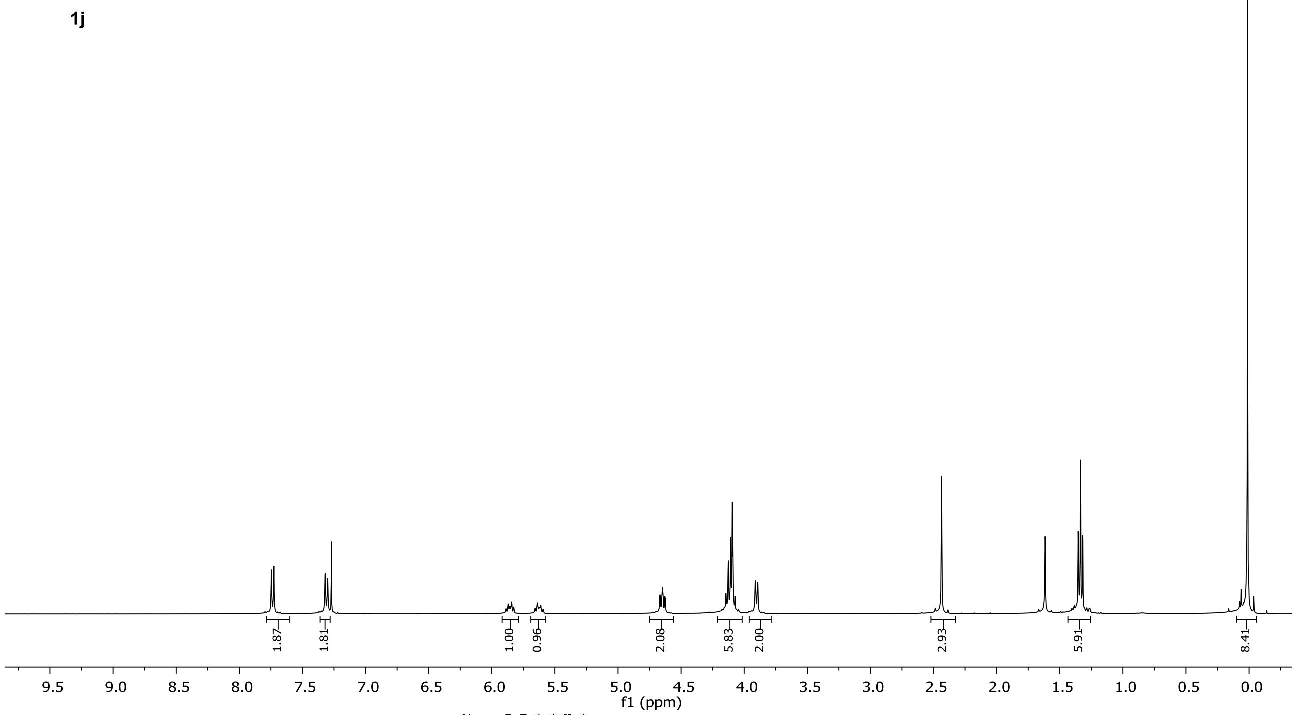
1.87
1.81
1.00
0.96
2.08
5.83
2.00
2.93
5.91
8.41
9.5 9.0 8.5 8.0 7.5 7.0 6.5 6.0 5.5 5.0 4.5 4.0 3.5 3.0 2.5 2.0 1.5 1.0 0.5 0.0
f1 (ppm)

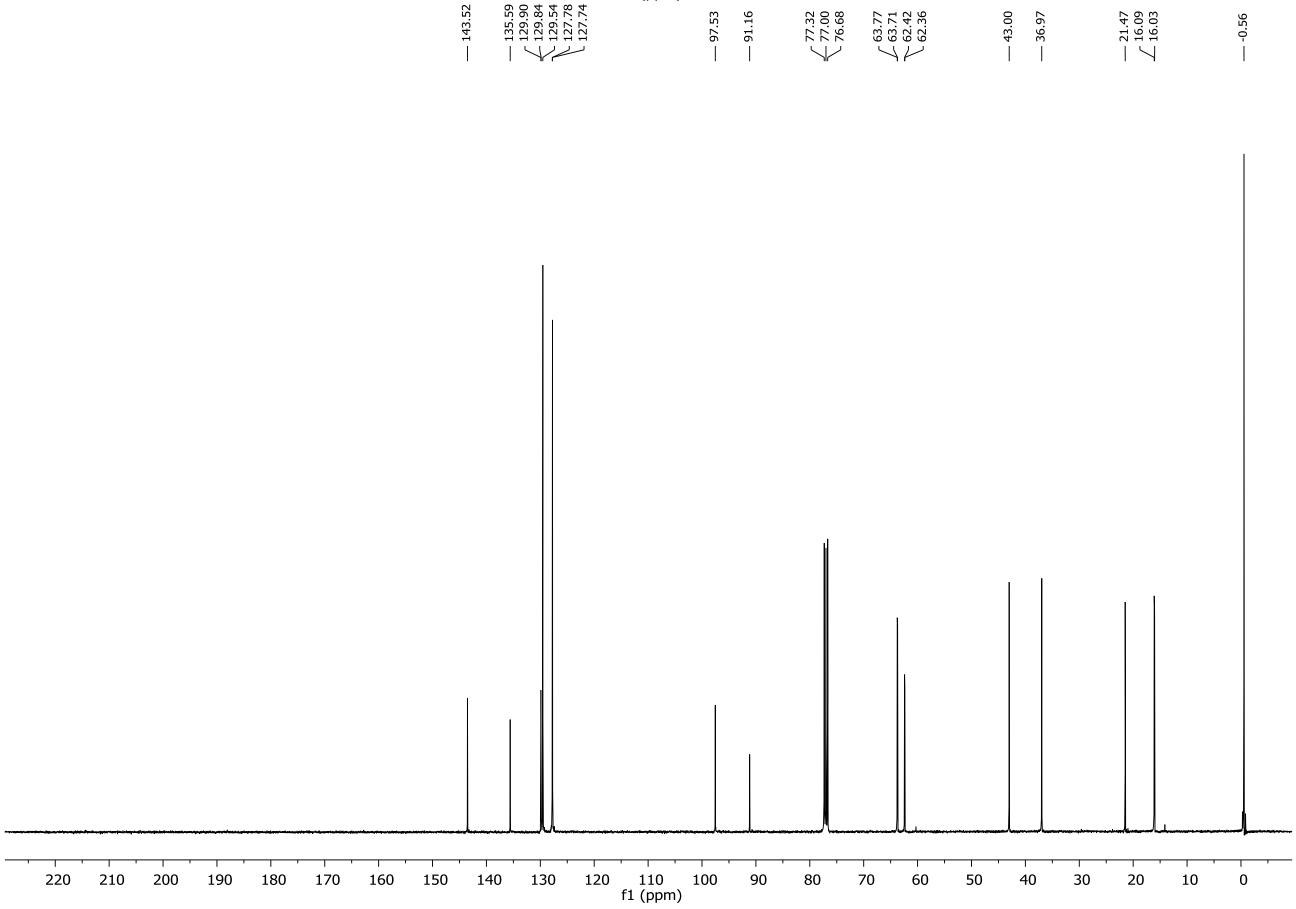
143.52
135.59
129.90
129.84
129.54
127.78
127.74
97.53
91.16
77.32
77.00
76.68
63.77
63.71
62.42
62.36
43.00
36.97
21.47
16.09
16.03
-0.56
220 210 200 190 180 170 160 150 140 130 120 110 100 90 80 70 60 50 40 30 20 10 0
f1 (ppm)

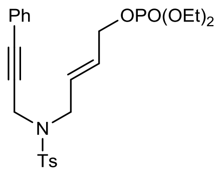
Ph
OPO(OEt)2
N
Ts

4

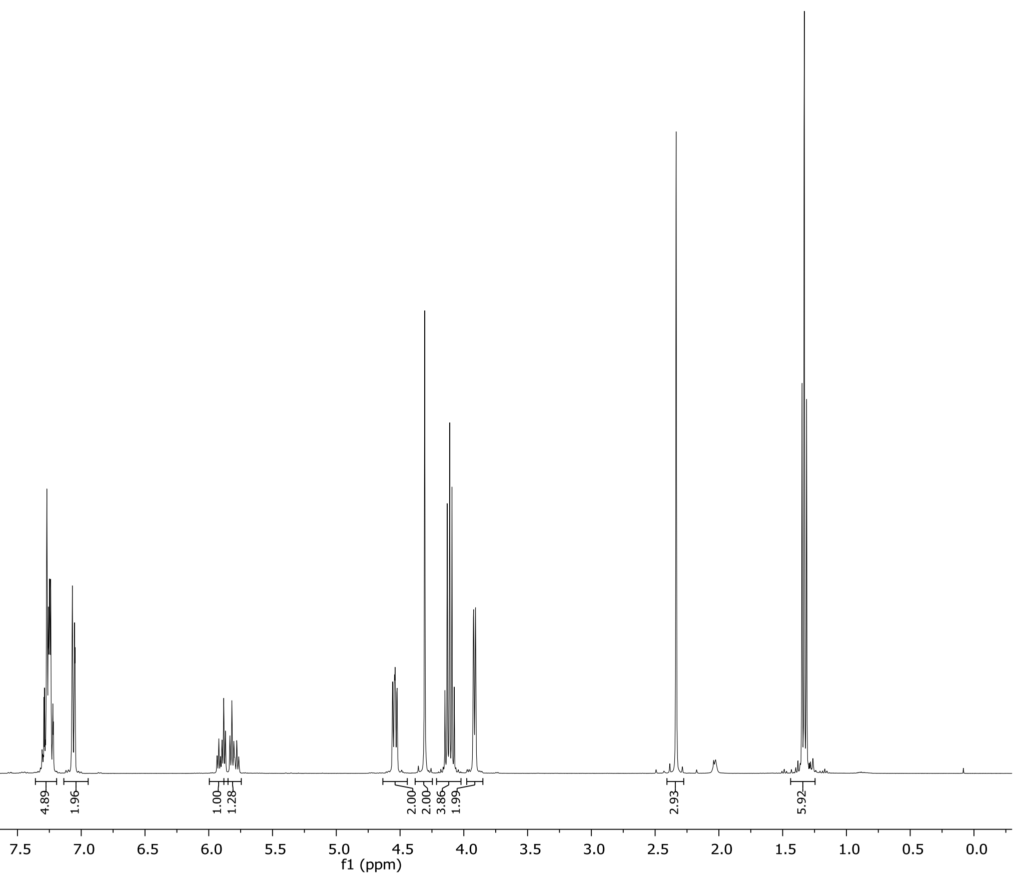
1.95
4.89
1.96
1.00
1.28
2.00
2.00
3.86
1.99
2.93
5.92
9.5 9.0 8.5 8.0 7.5 7.0 6.5 6.0 5.5 5.0 4.5 4.0 3.5 3.0 2.5 2.0 1.5 1.0 0.5 0.0
f1 (ppm)

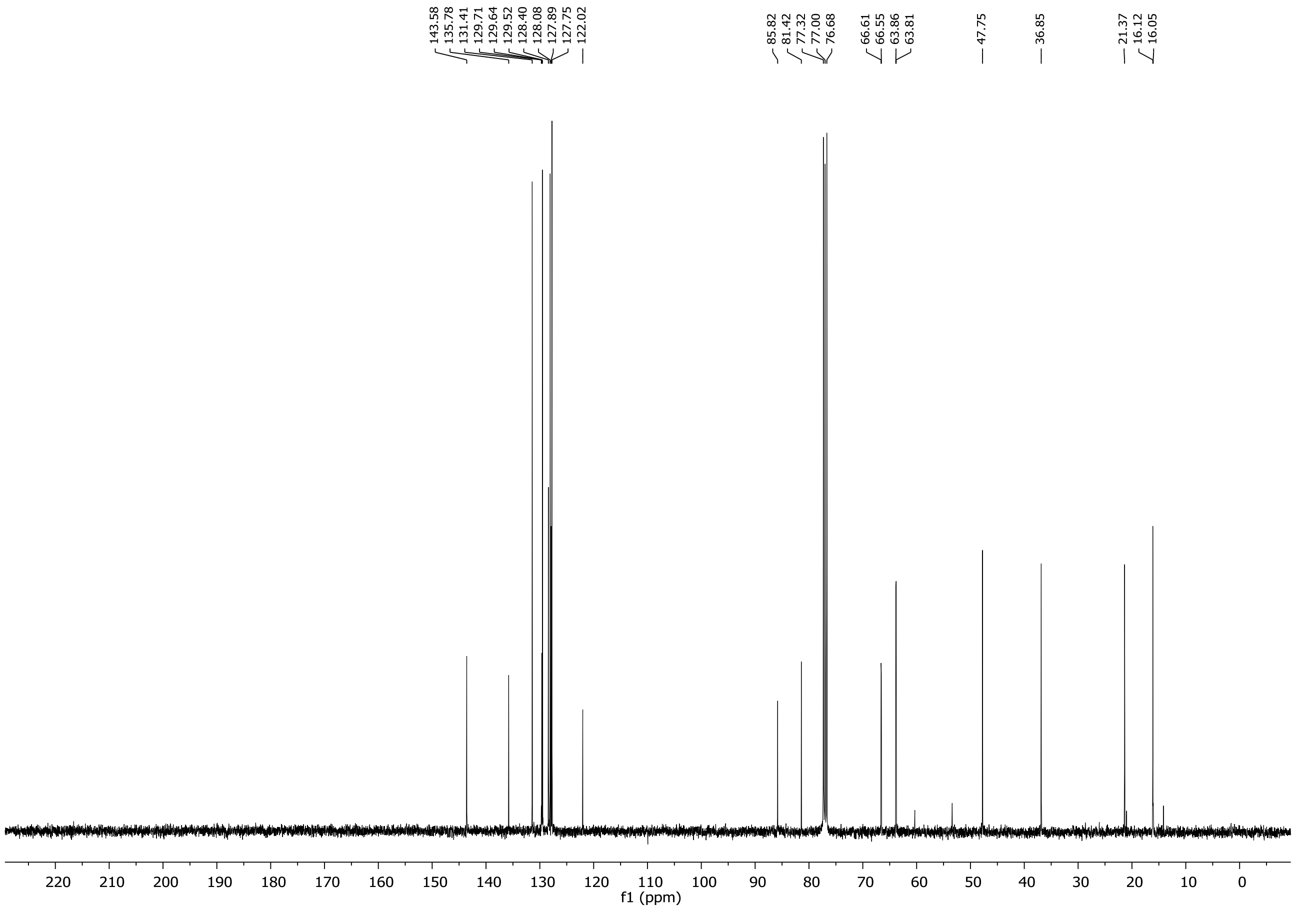
143.58
135.78
131.41
129.71
129.64
129.52
128.40
128.08
127.89
127.75
122.02
85.82
81.42
77.32
77.00
76.68
66.61
66.55
63.86
63.81
47.75
36.85
21.37
16.12
16.05
220 210 200 190 180 170 160 150 140 130 120 110 100 90 80 70 60 50 40 30 20 10 0
f1 (ppm)

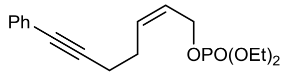
Ph
OPO(OEt)2

6

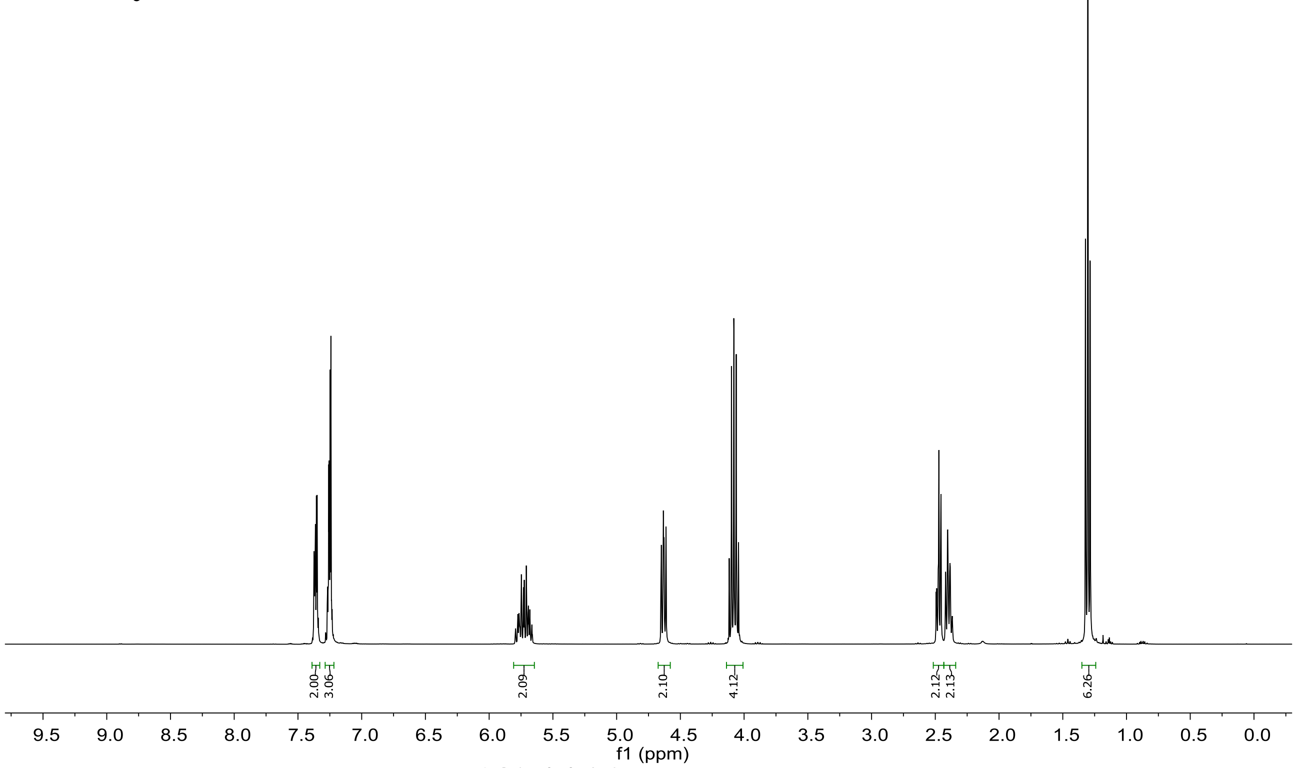
2.00
3.06
2.09
2.10
4.12
2.12
2.13
6.26
9.5 9.0 8.5 8.0 7.5 7.0 6.5 6.0 5.5 5.0 4.5 4.0 3.5 3.0 2.5 2.0 1.5 1.0 0.5 0.0
f1 (ppm)

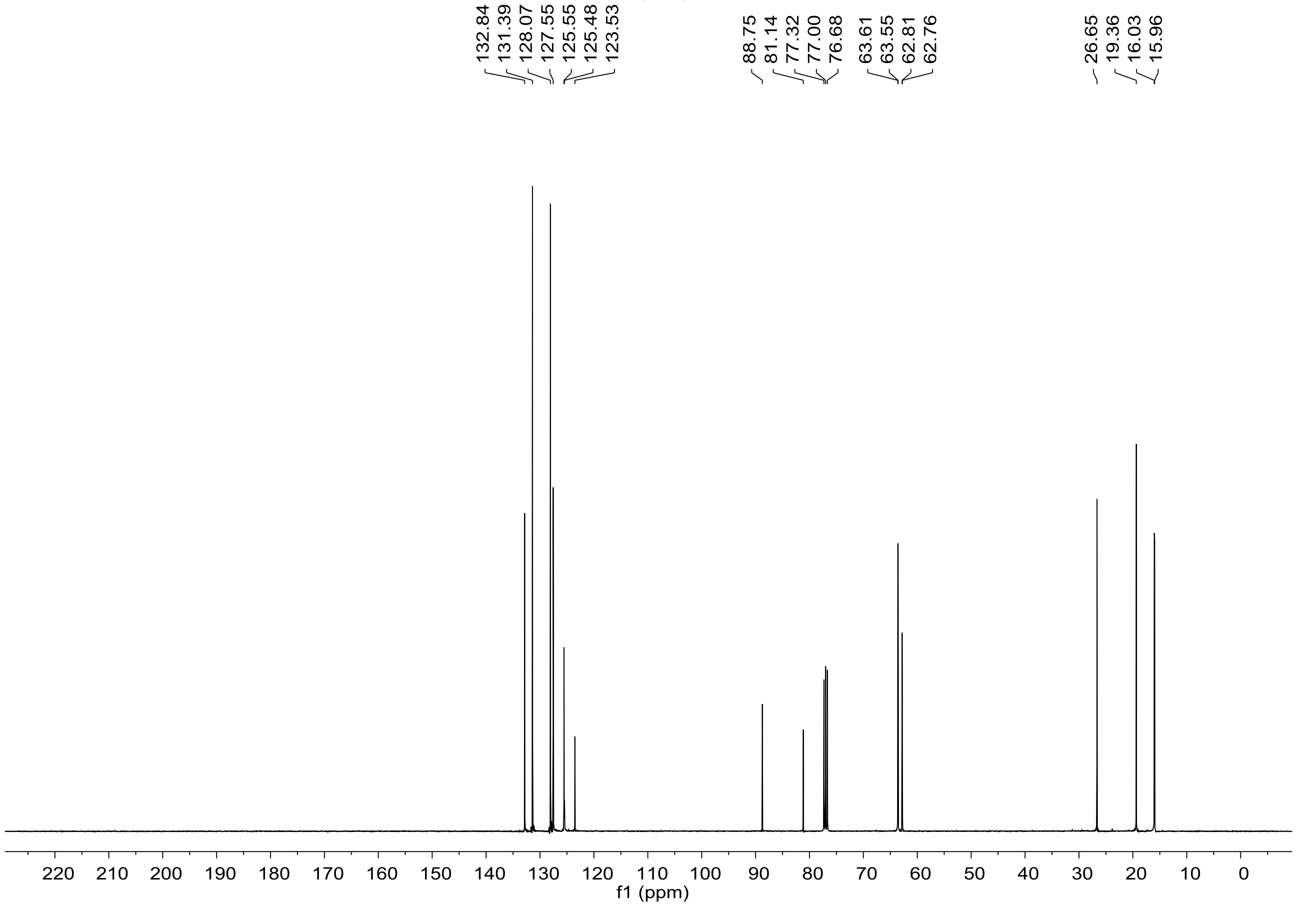
132.84
131.39
128.07
127.55
125.55
125.48
123.53
88.75
81.14
77.32
77.00
76.68
63.61
63.55
62.81
62.76
26.65
19.36
16.03
15.96
220 210 200 190 180 170 160 150 140 130 120 110 100 90 80 70 60 50 40 30 20 10 0
f1 (ppm)

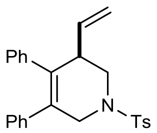

2a

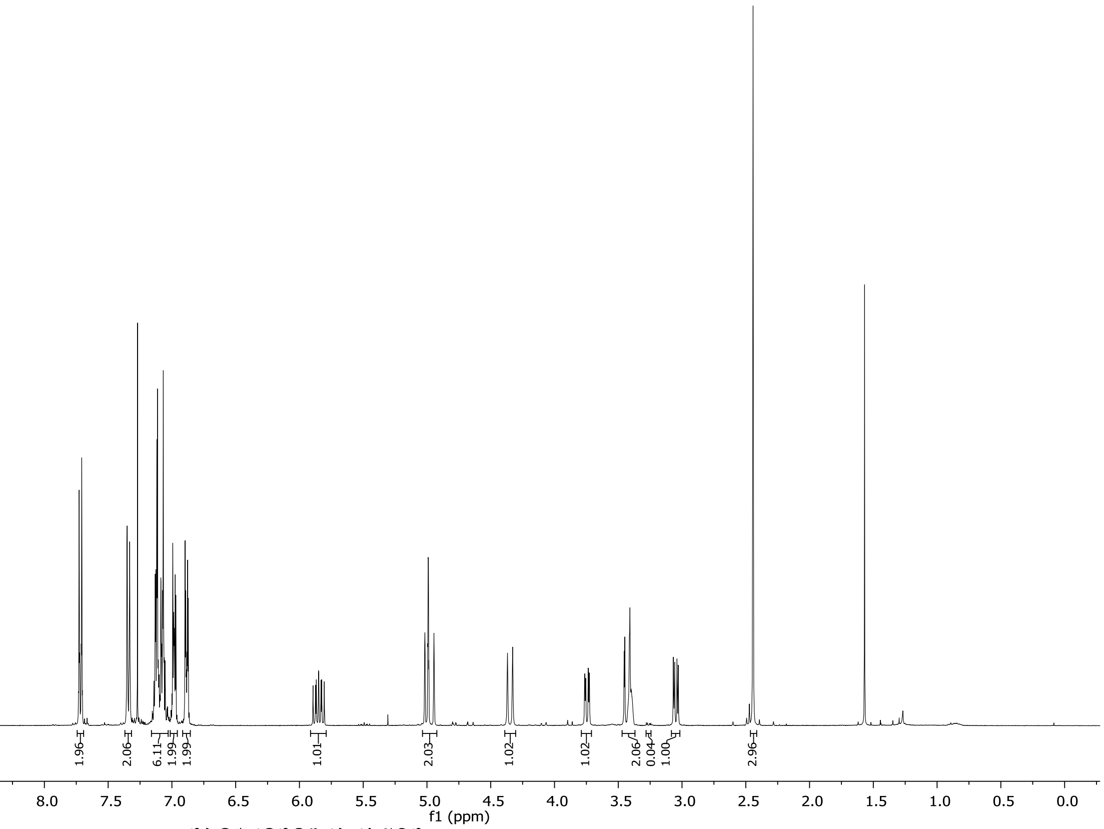
1.96
2.06
6.11
1.99
1.99
1.01
2.03
1.02
1.02
2.06
0.04
1.00
2.96
9.5 9.0 8.5 8.0 7.5 7.0 6.5 6.0 5.5 5.0 4.5 4.0 3.5 3.0 2.5 2.0 1.5 1.0 0.5 0.0
f1 (ppm)

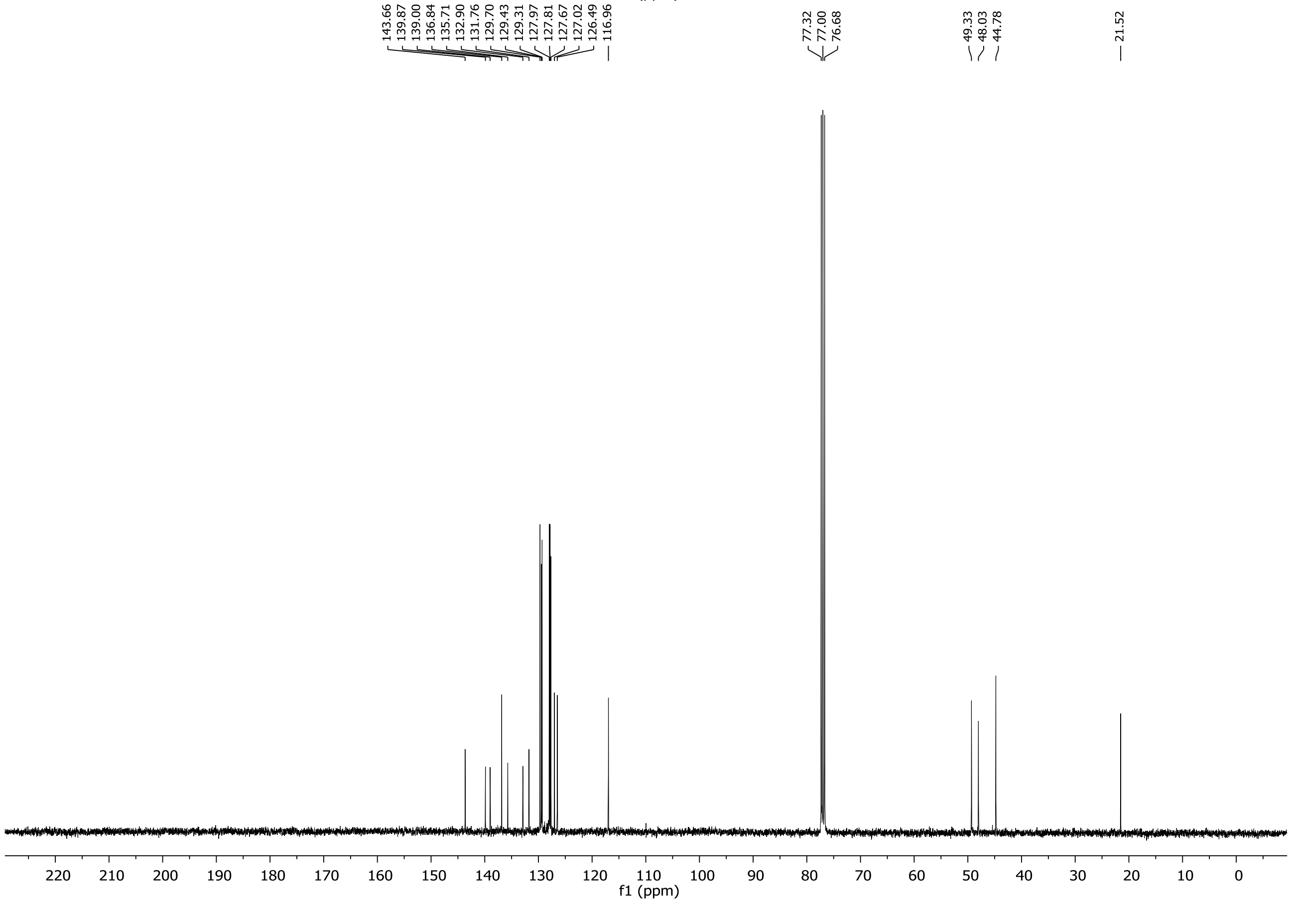
143.66
139.87
139.00
136.84
135.71
132.90
131.76
129.70
129.43
129.31
127.97
127.81
127.67
127.02
126.49
116.96
77.32
77.00
76.68
49.33
48.03
44.78
21.52
220 210 200 190 180 170 160 150 140 130 120 110 100 90 80 70 60 50 40 30 20 10 0
f1 (ppm)

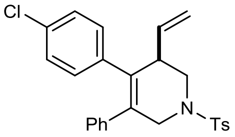
Cl
N
Ph
Ts

2b

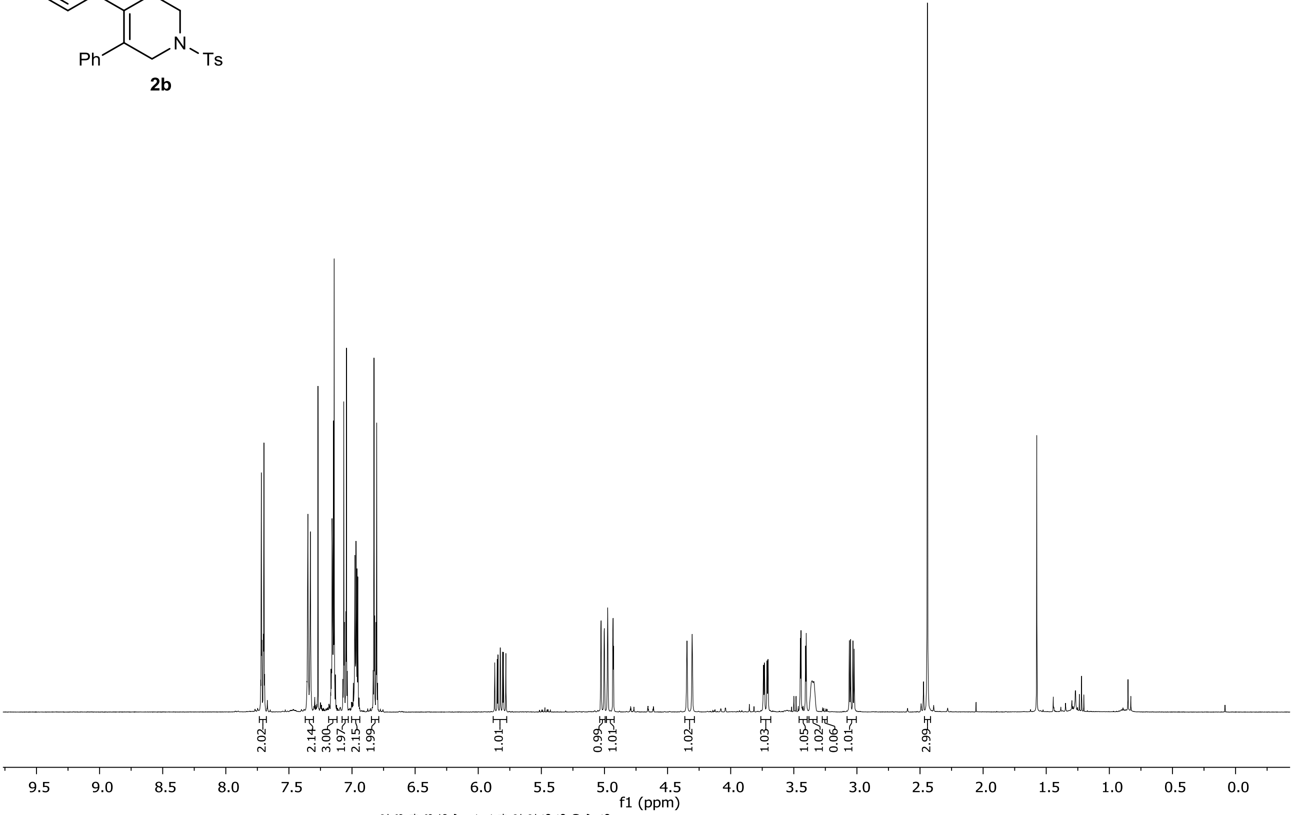
2.02
2.14
3.00
1.97
2.15
1.99
1.01
0.99
1.01
1.02
1.03
1.05
1.02
0.06
1.01
2.99
9.5 9.0 8.5 8.0 7.5 7.0 6.5 6.0 5.5 5.0 4.5 4.0 3.5 3.0 2.5 2.0 1.5 1.0 0.5 0.0
f1 (ppm)

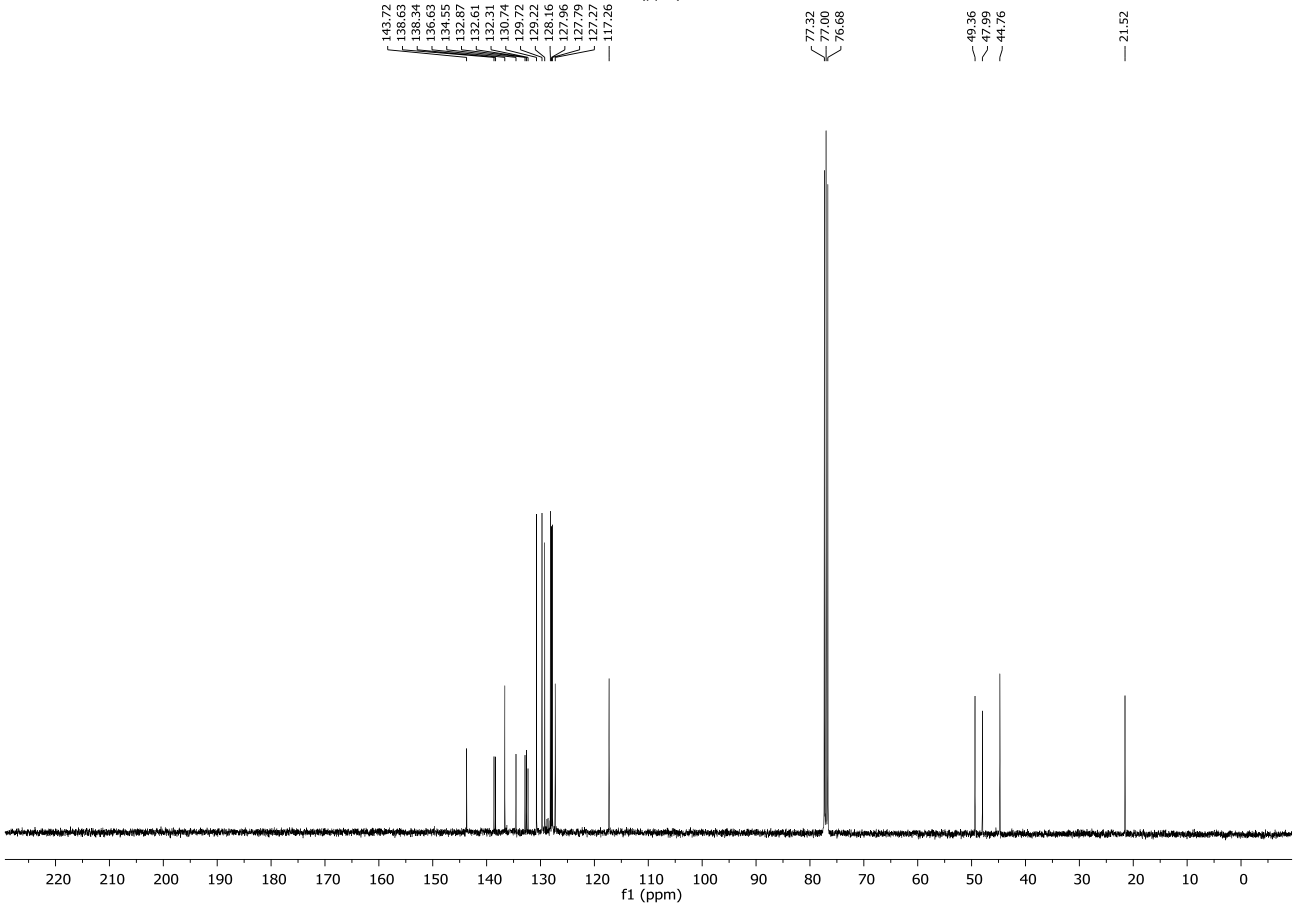
143.72
138.63
138.34
136.63
134.55
132.87
132.61
132.31
130.74
129.72
129.22
128.16
127.96
127.79
127.27
117.26
77.32
77.00
76.68
49.36
47.99
44.76
21.52
220 210 200 190 180 170 160 150 140 130 120 110 100 90 80 70 60 50 40 30 20 10 0
f1 (ppm)

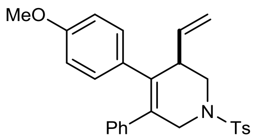
MeO
Ph
N
Ts

2c

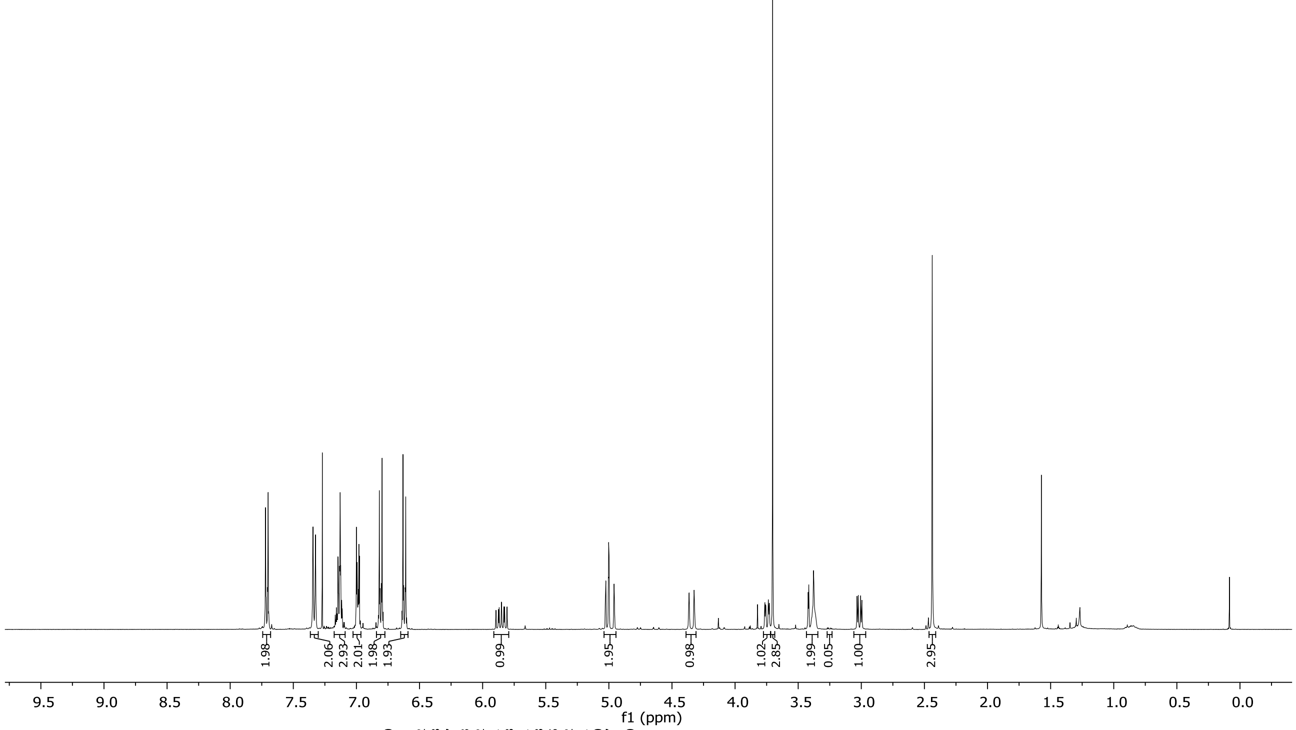
1.98
2.06
2.93
2.01
1.98
1.93
0.99
1.95
0.98
1.02
2.85
1.99
0.05
1.00
2.95
9.5 9.0 8.5 8.0 7.5 7.0 6.5 6.0 5.5 5.0 4.5 4.0 3.5 3.0 2.5 2.0 1.5 1.0 0.5 0.0
f1 (ppm)

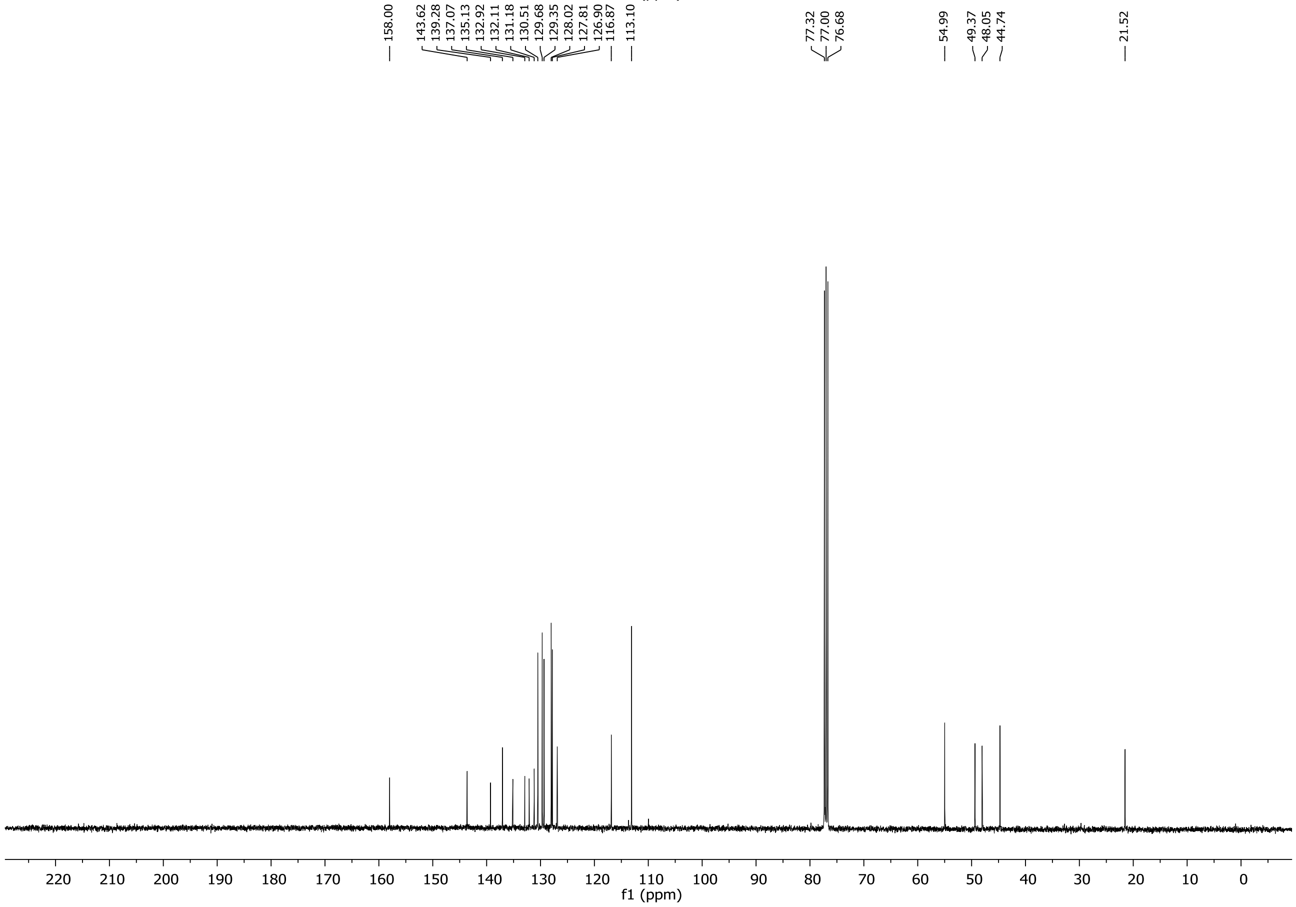
158.00
143.62
139.28
137.07
135.13
132.92
132.11
131.18
130.51
129.68
129.35
128.02
127.81
126.90
116.87
113.10
77.32
77.00
76.68
54.99
49.37
48.05
44.74
21.52
220 210 200 190 180 170 160 150 140 130 120 110 100 90 80 70 60 50 40 30 20 10 0
f1 (ppm)

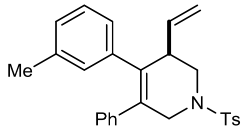
Me
Ph
N
Ts

2d

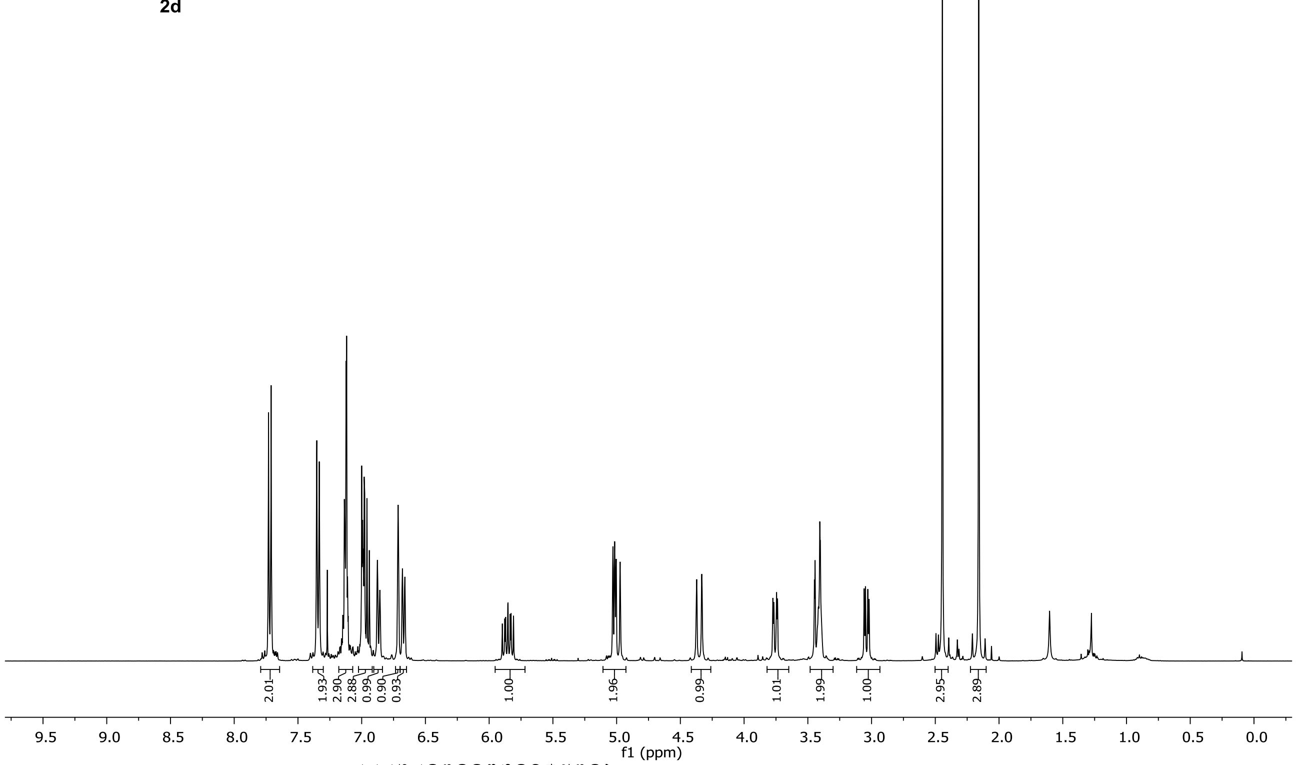
2.01
1.93
2.90
2.88
0.99
0.90
0.93
1.00
1.96
0.99
1.01
1.99
1.00
2.95
2.89
f1 (ppm)

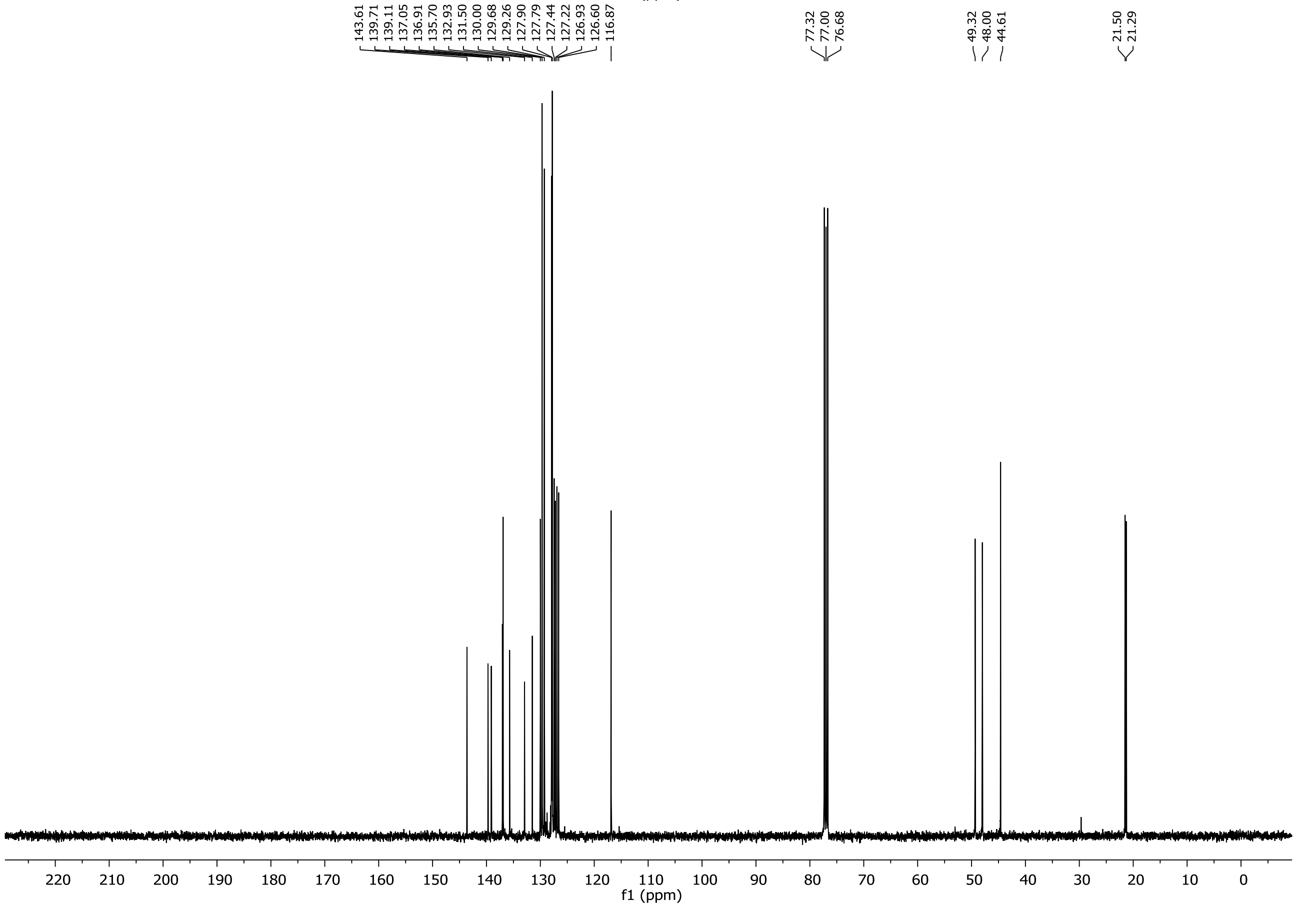
143.61
139.71
139.11
137.05
136.91
135.70
132.93
131.50
130.00
129.68
129.26
127.90
127.79
127.44
127.22
126.93
126.60
116.87
77.32
77.00
76.68
49.32
48.00
44.61
21.50
21.29
f1 (ppm)

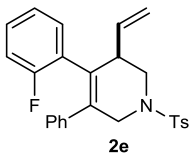
F
Ph
N
Ts

2e

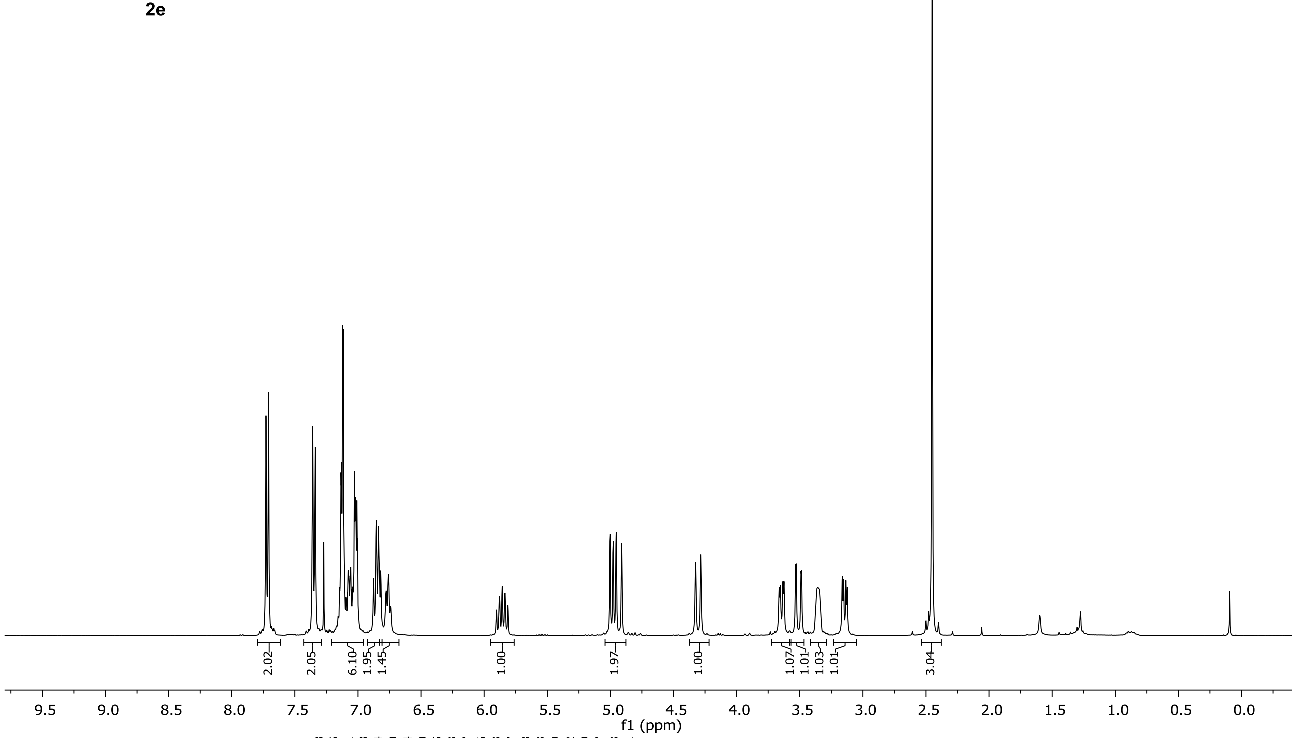
2.02
2.05
6.10
1.95
1.45
1.00
1.97
1.00
1.07
1.01
1.03
1.01
3.04
9.5 9.0 8.5 8.0 7.5 7.0 6.5 6.0 5.5 5.0 4.5 4.0 3.5 3.0 2.5 2.0 1.5 1.0 0.5 0.0
f1 (ppm)

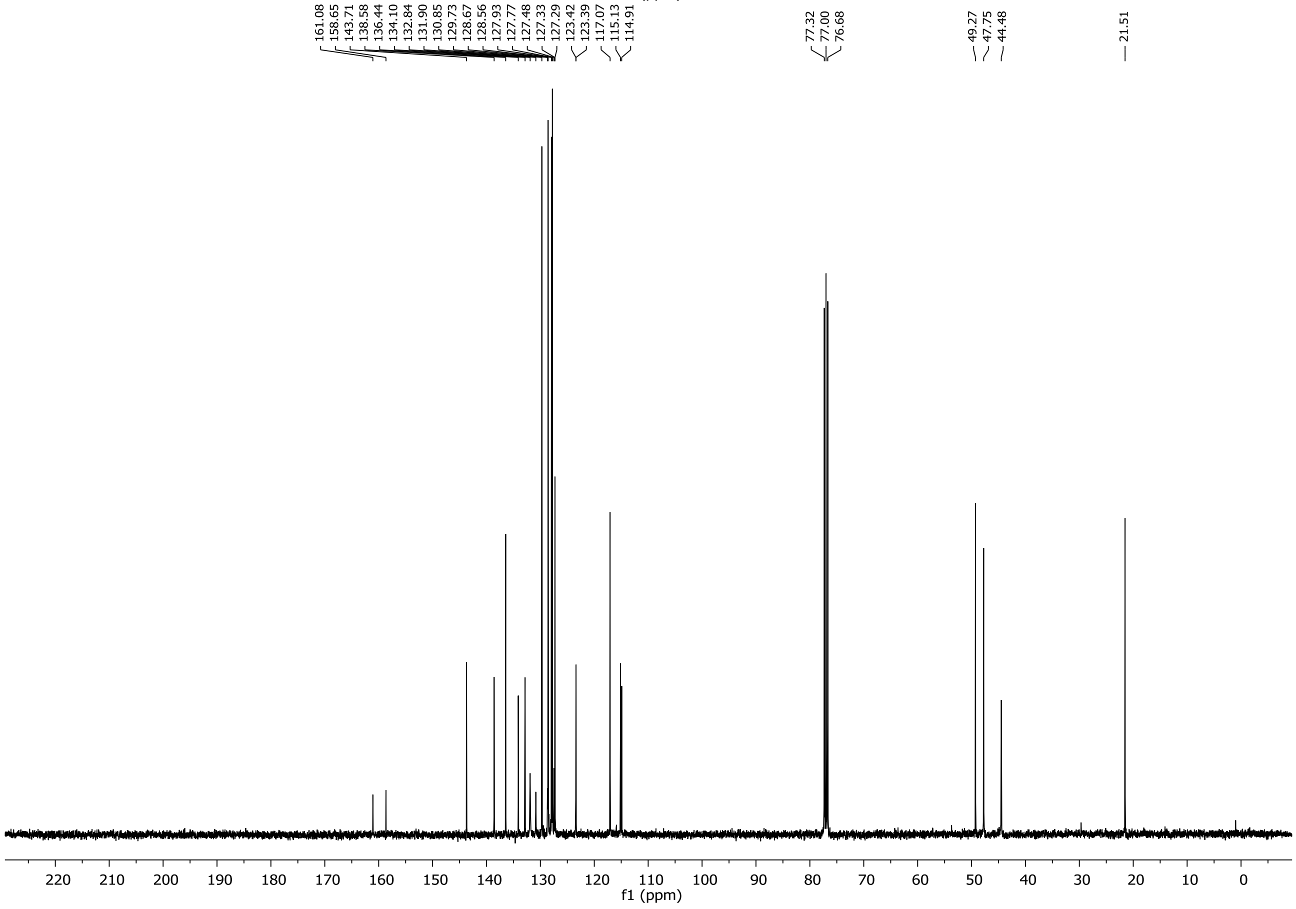
161.08
158.65
143.71
138.58
136.44
134.10
132.84
131.90
130.85
129.73
128.67
128.56
127.93
127.77
127.48
127.33
127.29
123.42
123.39
117.07
115.13
114.91
77.32
77.00
76.68
49.27
47.75
44.48
21.51
220 210 200 190 180 170 160 150 140 130 120 110 100 90 80 70 60 50 40 30 20 10 0
f1 (ppm)

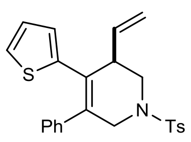
S
Ph
N
Ts

2f

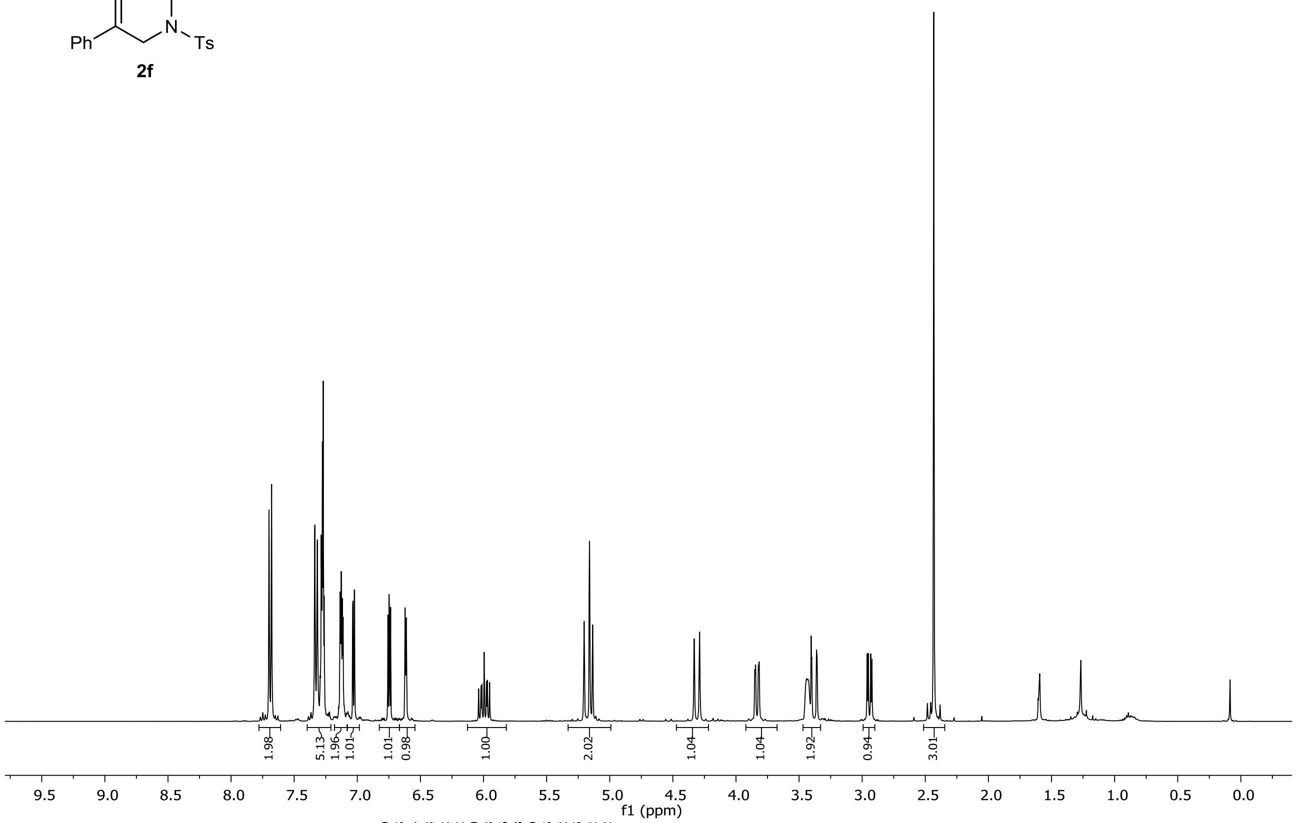
1.98
5.13
1.96
1.01
1.01
0.98
1.00
2.02
1.04
1.04
1.92
0.94
3.01
9.5 9.0 8.5 8.0 7.5 7.0 6.5 6.0 5.5 5.0 4.5 4.0 3.5 3.0 2.5 2.0 1.5 1.0 0.5 0.0
f1 (ppm)

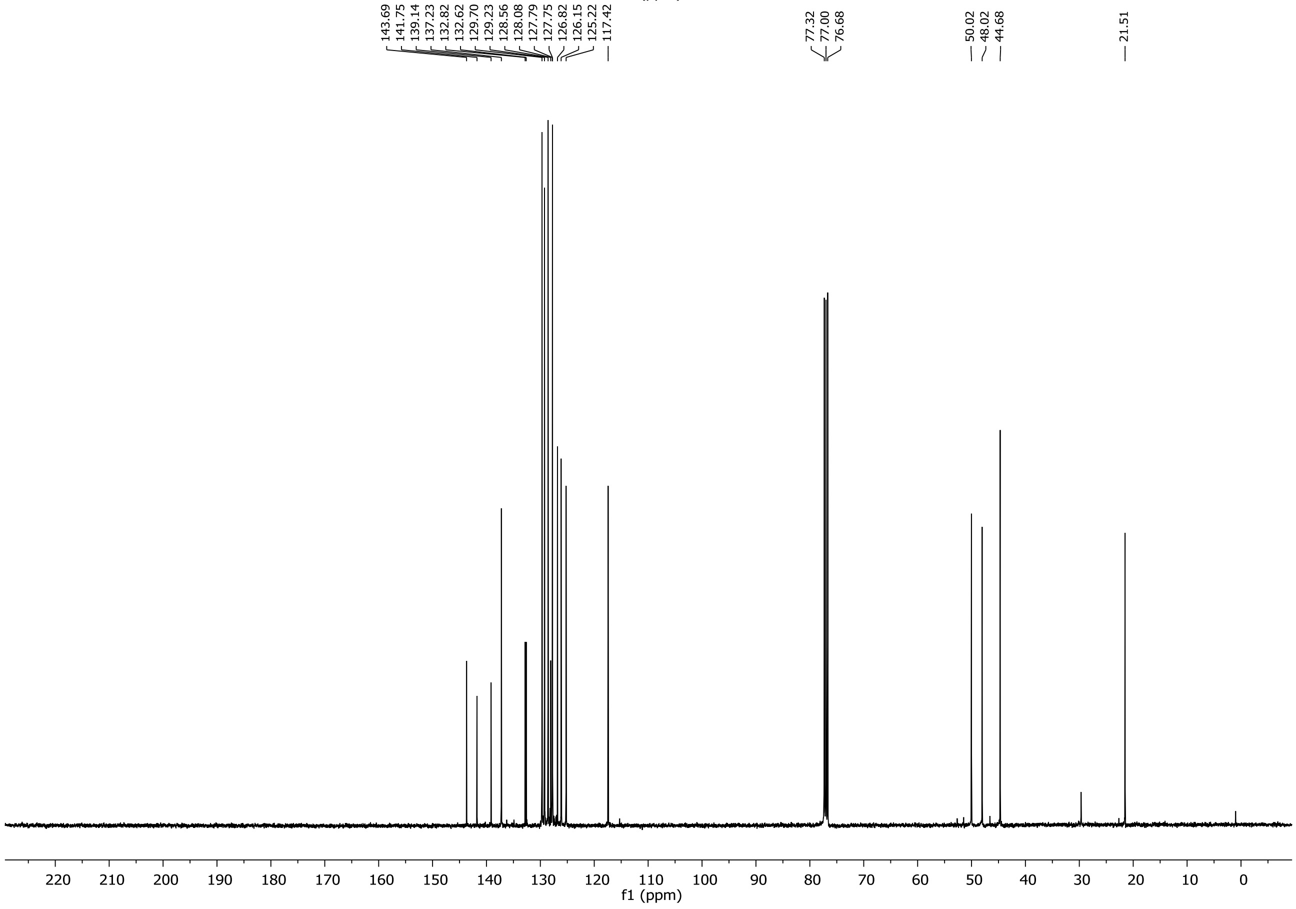
143.69
141.75
139.14
137.23
132.82
132.62
129.70
129.23
128.56
128.08
127.79
127.75
126.82
126.15
125.22
117.42
77.32
77.00
76.68
50.02
48.02
44.68
21.51
220 210 200 190 180 170 160 150 140 130 120 110 100 90 80 70 60 50 40 30 20 10 0
f1 (ppm)

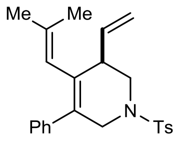
Me
Me
Ph
N
Ts

2g

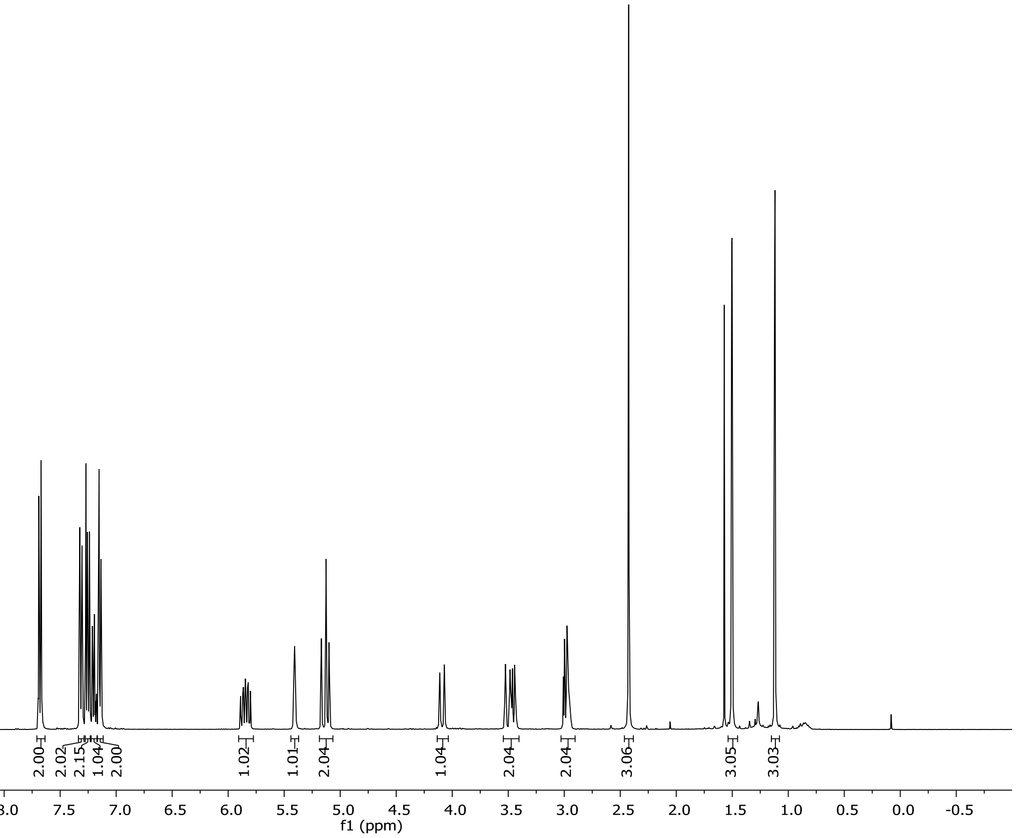
2.00
2.02
2.15
1.04
2.00
1.02
1.01
2.04
1.04
2.04
2.04
3.06
3.05
3.03
f1 (ppm)

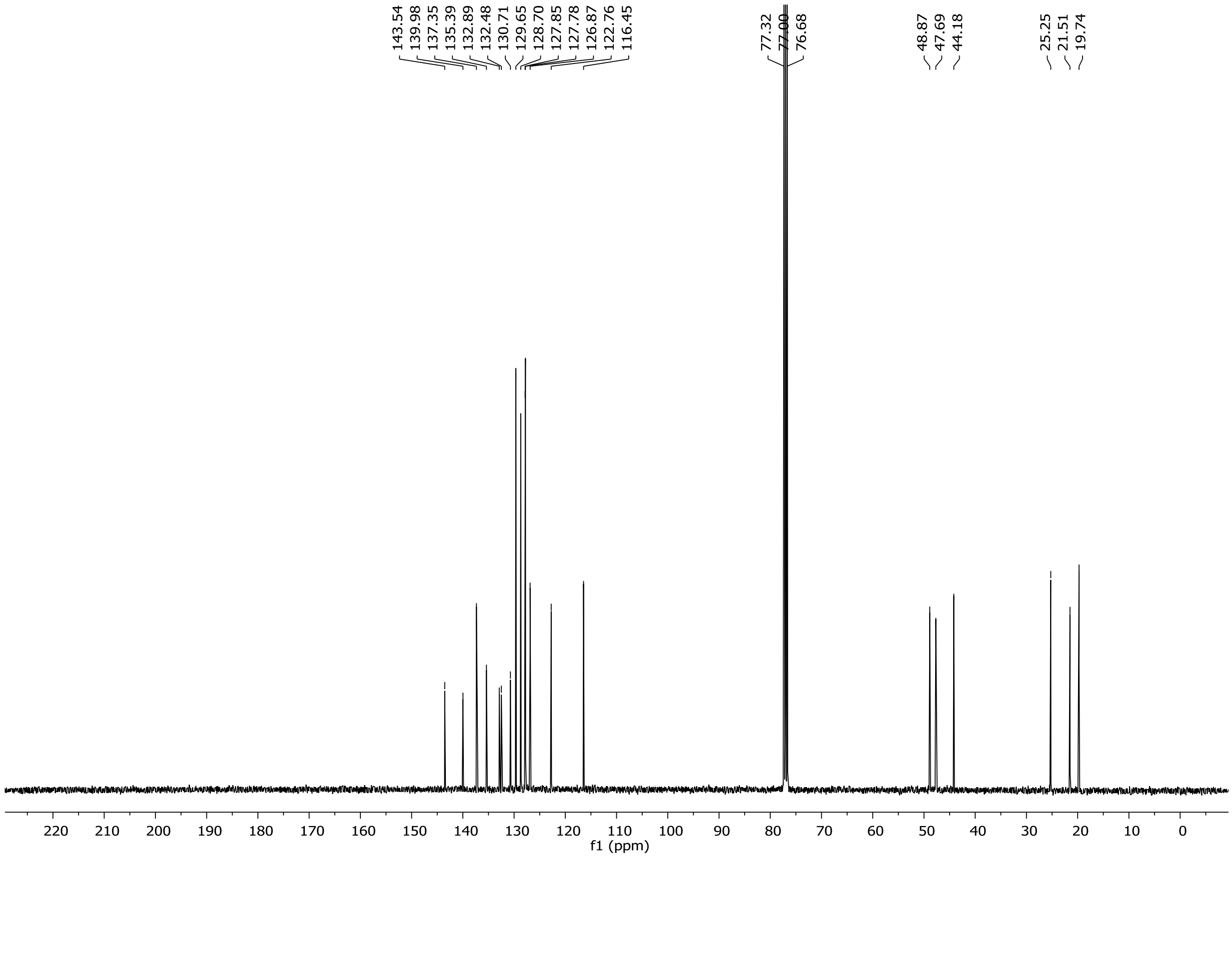
143.54
139.98
137.35
135.39
132.89
132.48
130.71
129.65
128.70
127.85
127.78
126.87
122.76
116.45
77.32
77.00
76.68
48.87
47.69
44.18
25.25
21.51
19.74
f1 (ppm)

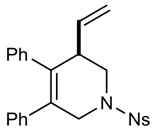
Ph
Ph
N
Ns

2h

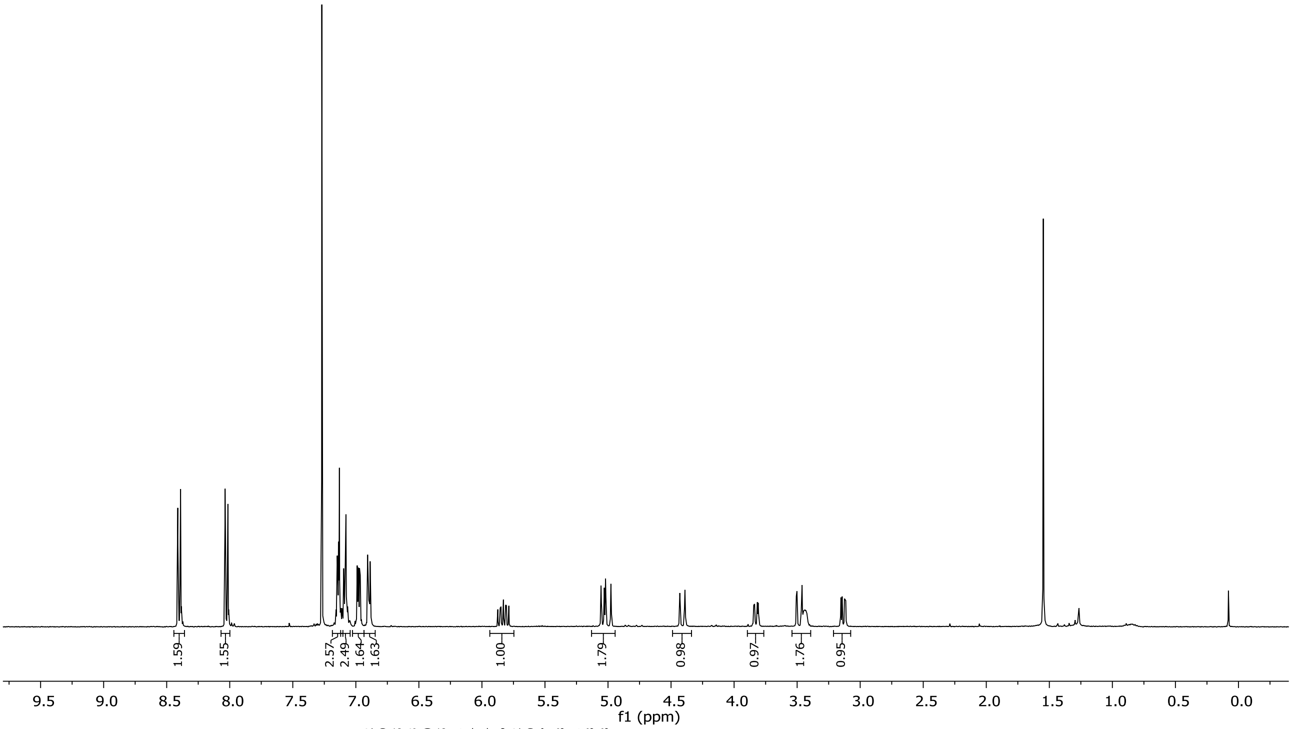
1.59
1.55
2.57
2.49
1.64
1.63
1.00
1.79
0.98
0.97
1.76
0.95
f1 (ppm)

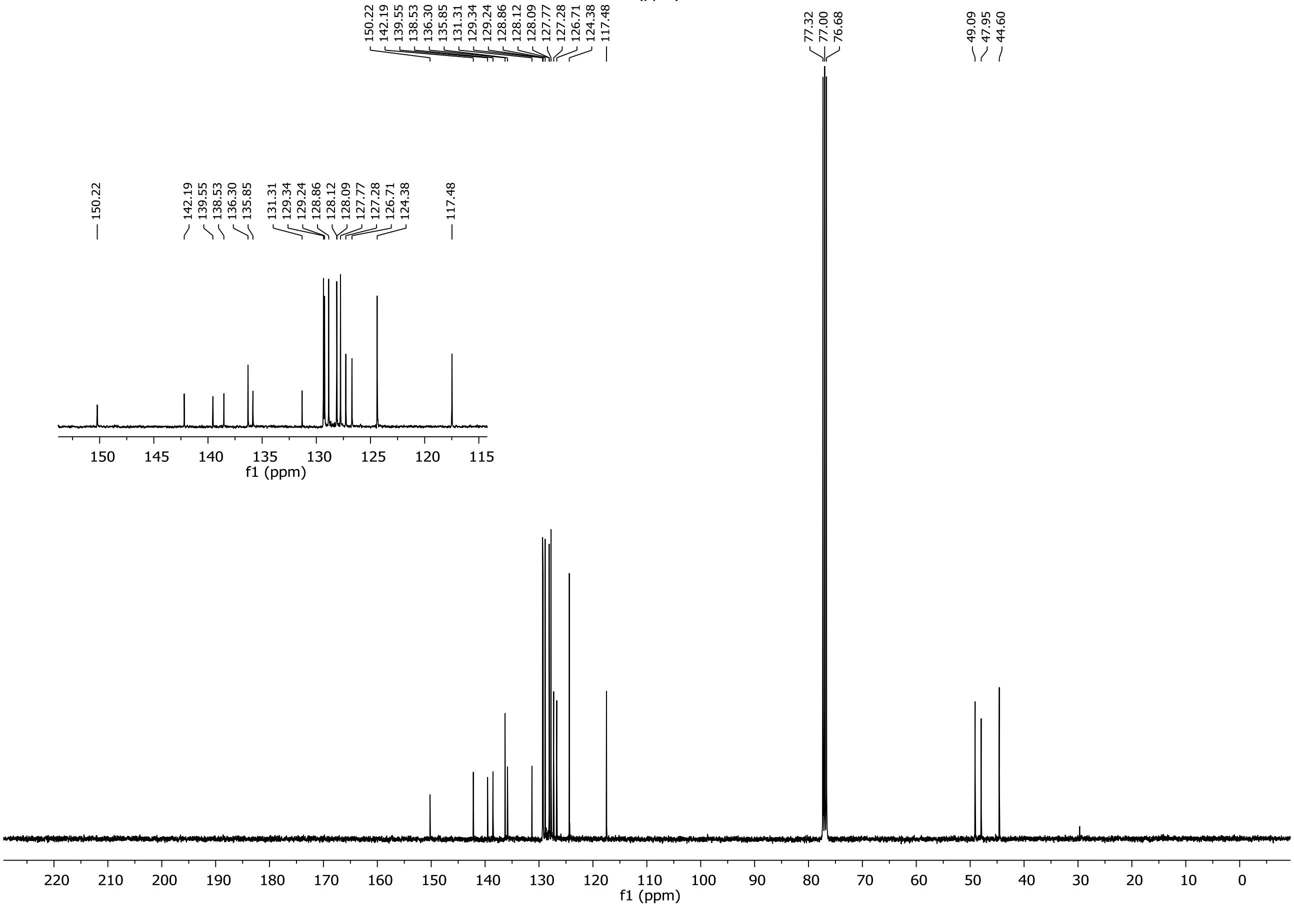
150.22
142.19
139.55
138.53
136.30
135.85
131.31
129.34
129.24
128.86
128.12
128.09
127.77
127.28
126.71
124.38
117.48
77.32
77.00
76.68
49.09
47.95
44.60
f1 (ppm)

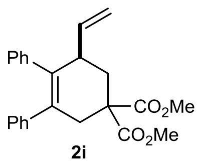
Ph
Ph
CO_2Me
CO_2Me

2i

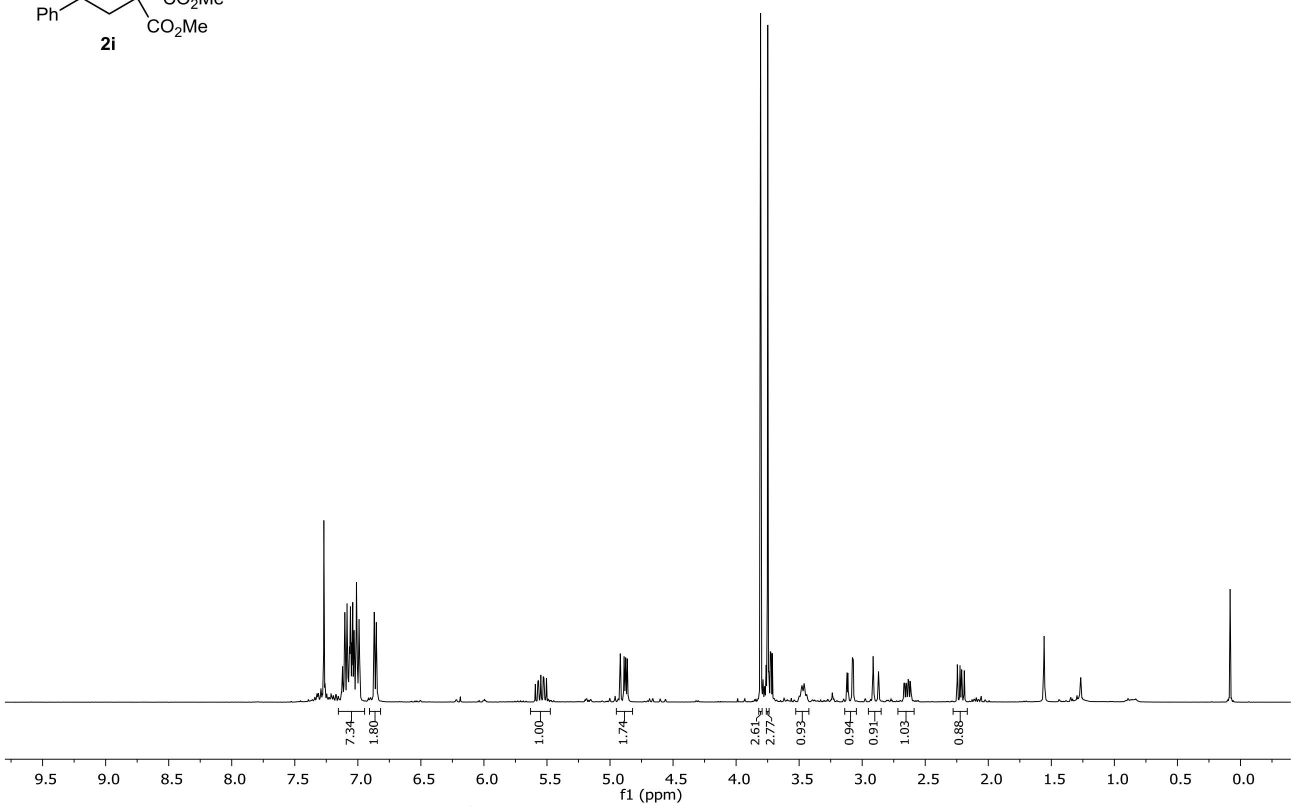
7.34
1.80
1.00
1.74
2.61
2.77
0.93
0.94
0.91
1.03
0.88
f1 (ppm)

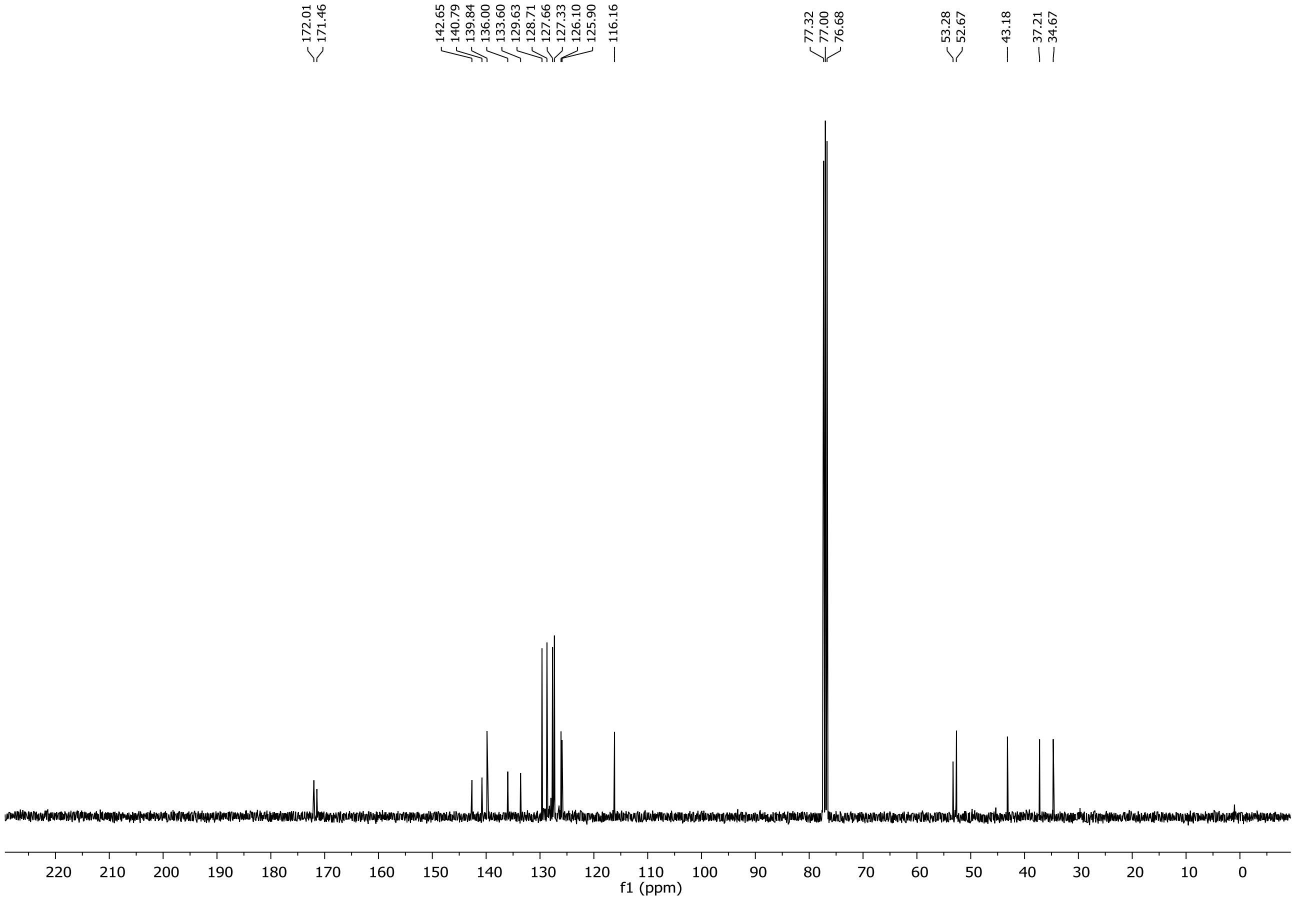
172.01
171.46
142.65
140.79
139.84
136.00
133.60
129.63
128.71
127.66
127.33
126.10
125.90
116.16
77.32
77.00
76.68
53.28
52.67
43.18
37.21
34.67
f1 (ppm)

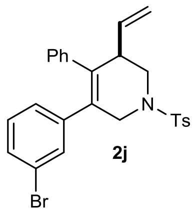
Ph
N
Ts
Br
2j

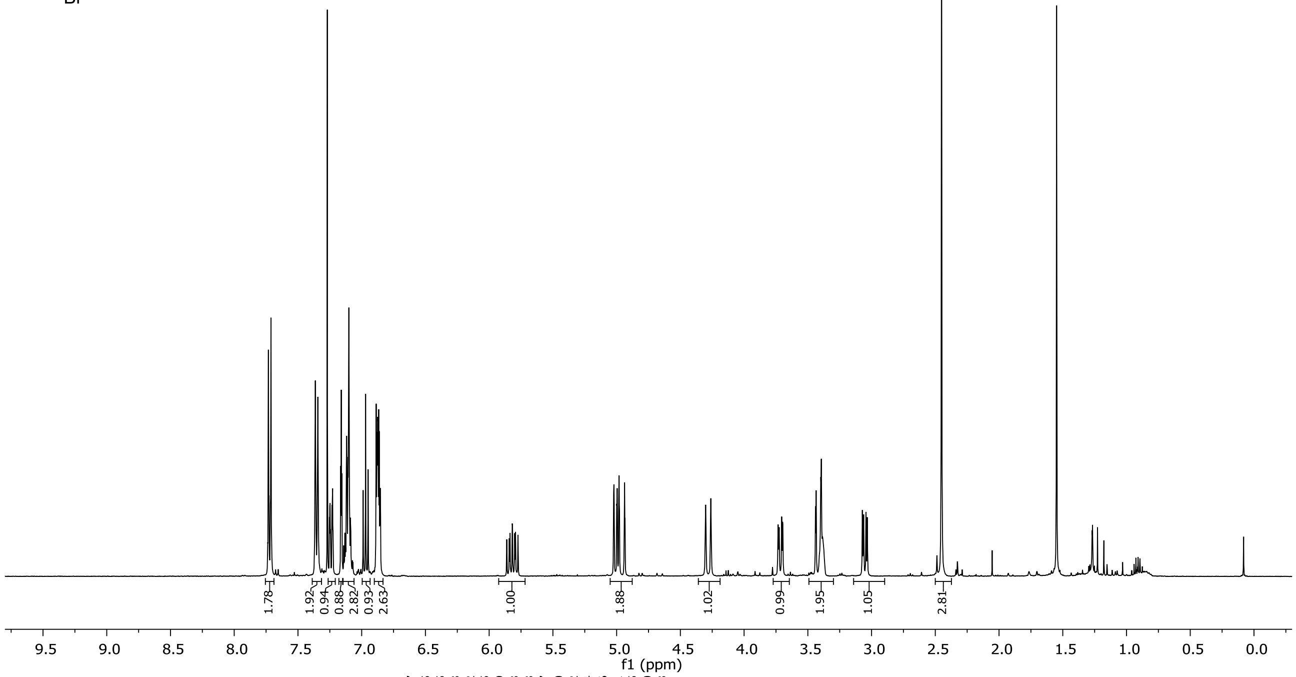
1.78
1.92
0.94
0.88
2.82
0.93
2.63
1.00
1.88
1.02
0.99
1.95
1.05
2.81
9.5 9.0 8.5 8.0 7.5 7.0 6.5 6.0 5.5 5.0 4.5 4.0 3.5 3.0 2.5 2.0 1.5 1.0 0.5 0.0
f1 (ppm)

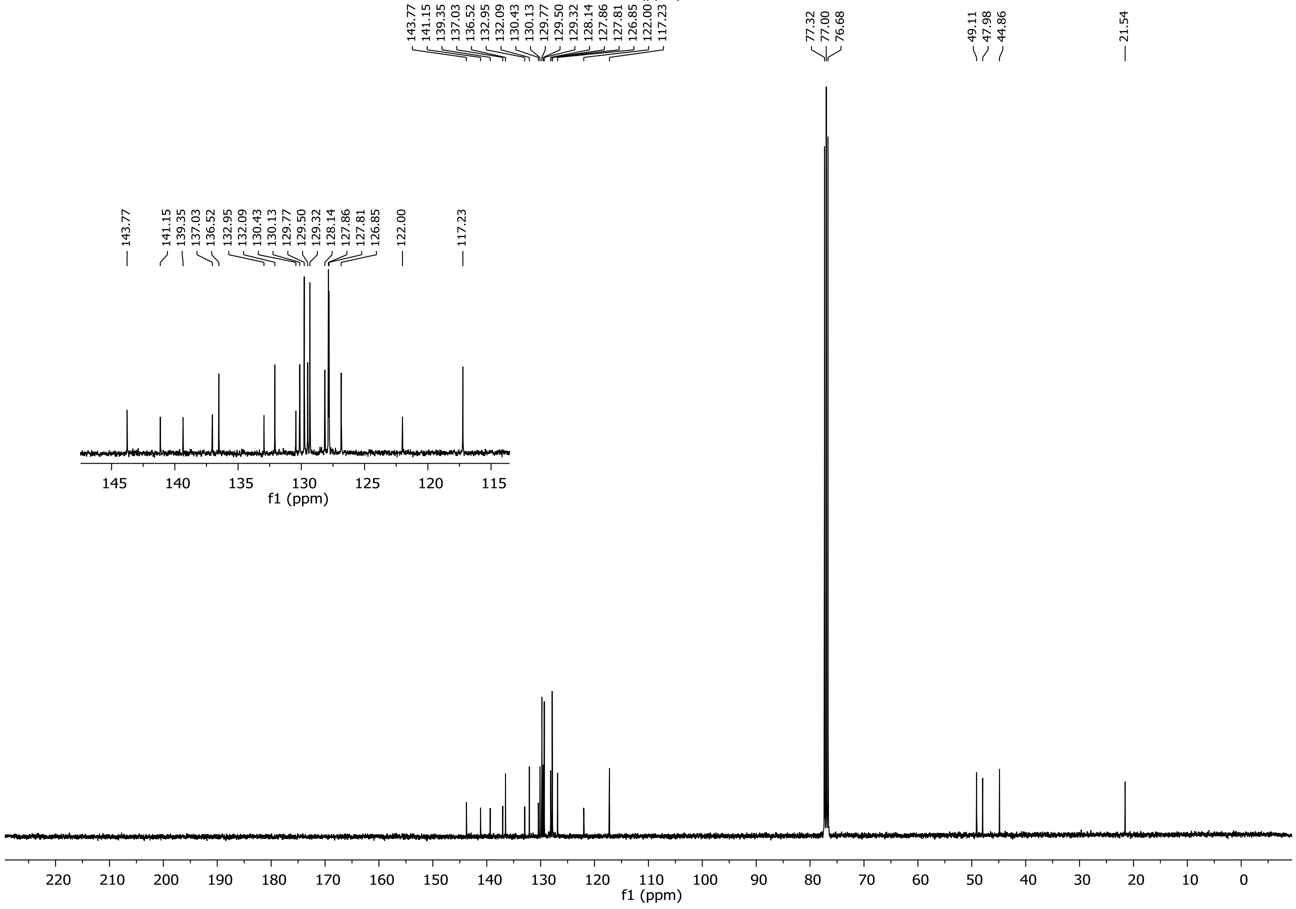
143.77
141.15
139.35
137.03
136.52
132.95
132.09
130.43
130.13
129.77
129.50
129.32
128.14
127.86
127.81
126.85
122.00
117.23
77.32
77.00
76.68
49.11
47.98
44.86
21.54
145 140 135 130 125 120 115
f1 (ppm)
220 210 200 190 180 170 160 150 140 130 120 110 100 90 80 70 60 50 40 30 20 10 0
f1 (ppm)

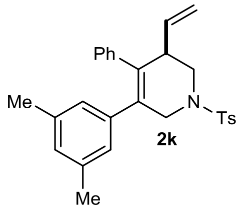
Ph
Me
N
Ts
2k
Me

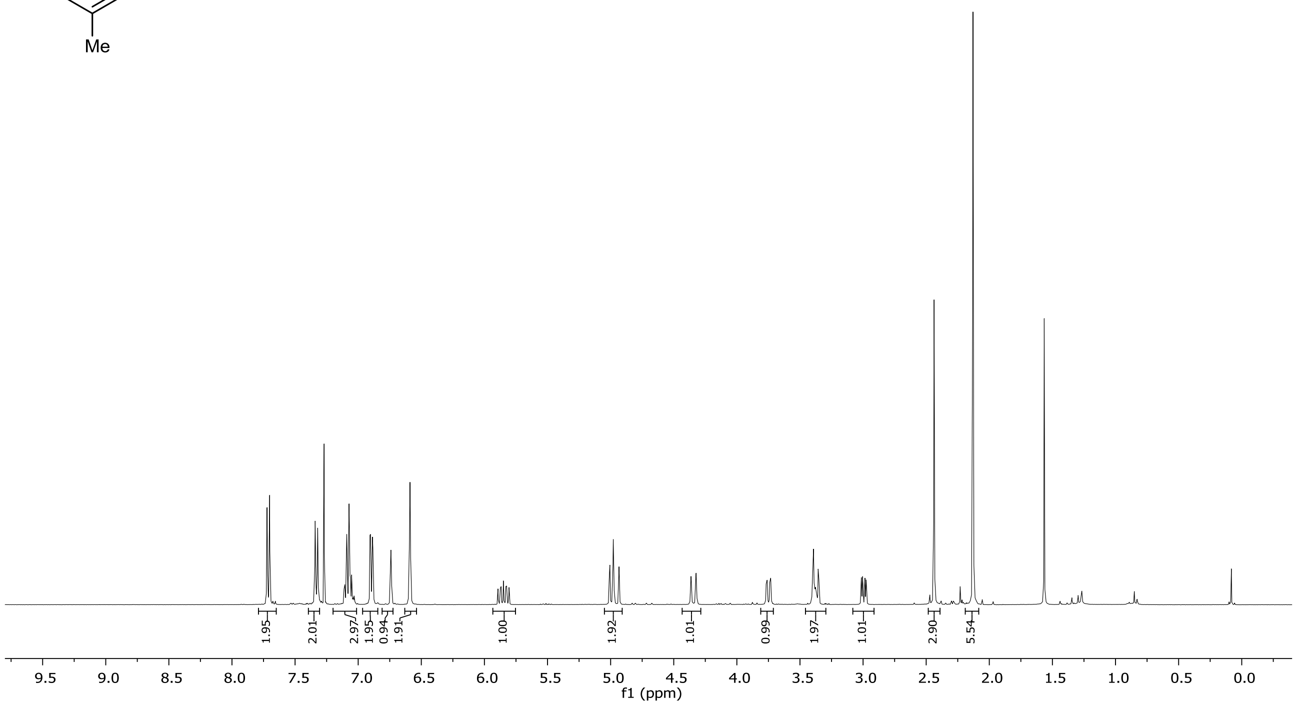
1.95
2.01
2.97
1.95
0.94
1.91
1.00
1.92
1.01
0.99
1.97
1.01
2.90
5.54
9.5 9.0 8.5 8.0 7.5 7.0 6.5 6.0 5.5 5.0 4.5 4.0 3.5 3.0 2.5 2.0 1.5 1.0 0.5 0.0
f1 (ppm)

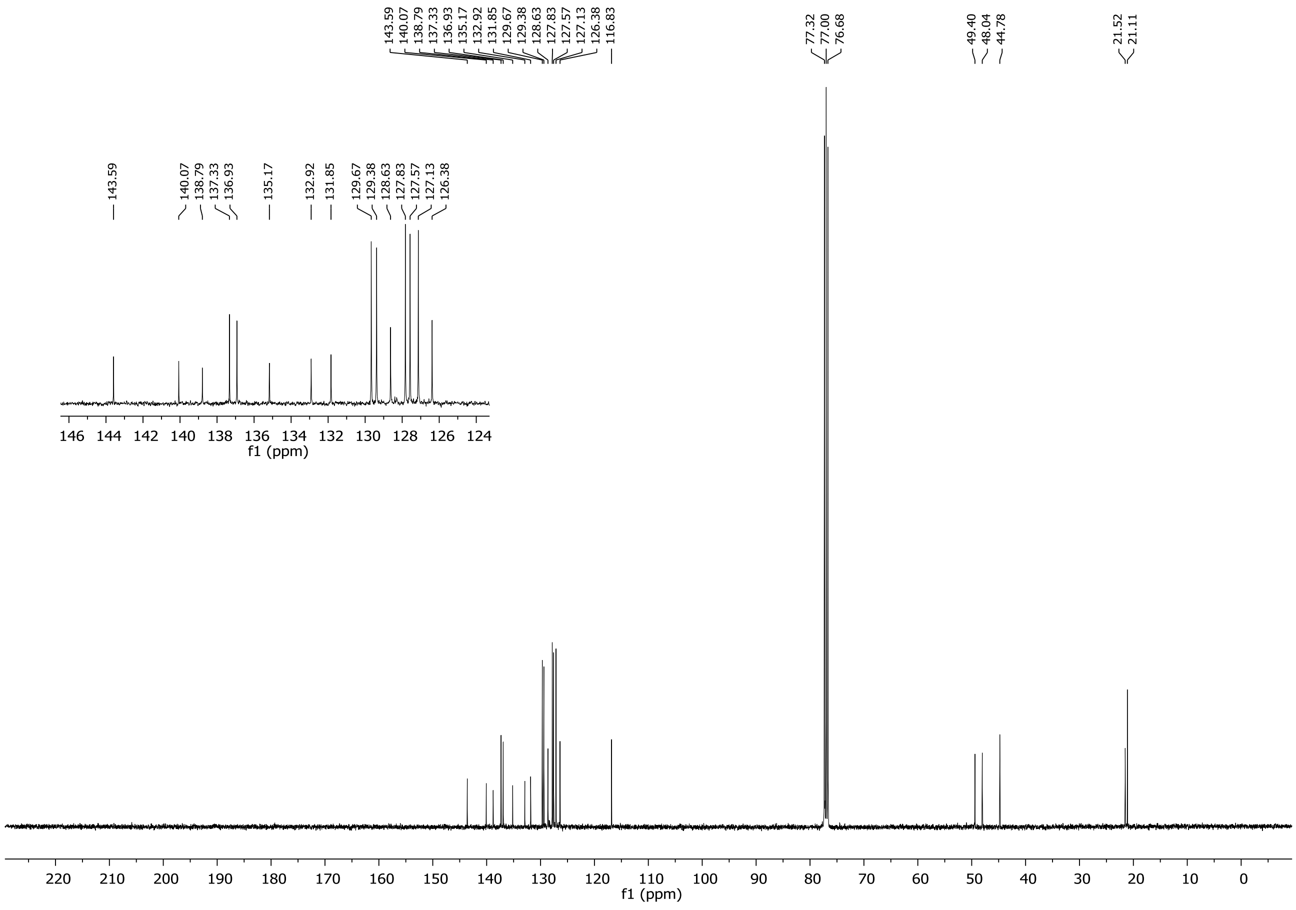
143.59
140.07
138.79
137.33
136.93
135.17
132.92
131.85
129.67
129.38
128.63
127.83
127.57
127.13
126.38
116.83
77.32
77.00
76.68
49.40
48.04
44.78
21.52
21.11
146 144 142 140 138 136 134 132 130 128 126 124
f1 (ppm)
220 210 200 190 180 170 160 150 140 130 120 110 100 90 80 70 60 50 40 30 20 10 0
f1 (ppm)

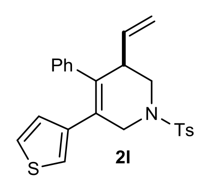
Ph
N
Ts
S

2l

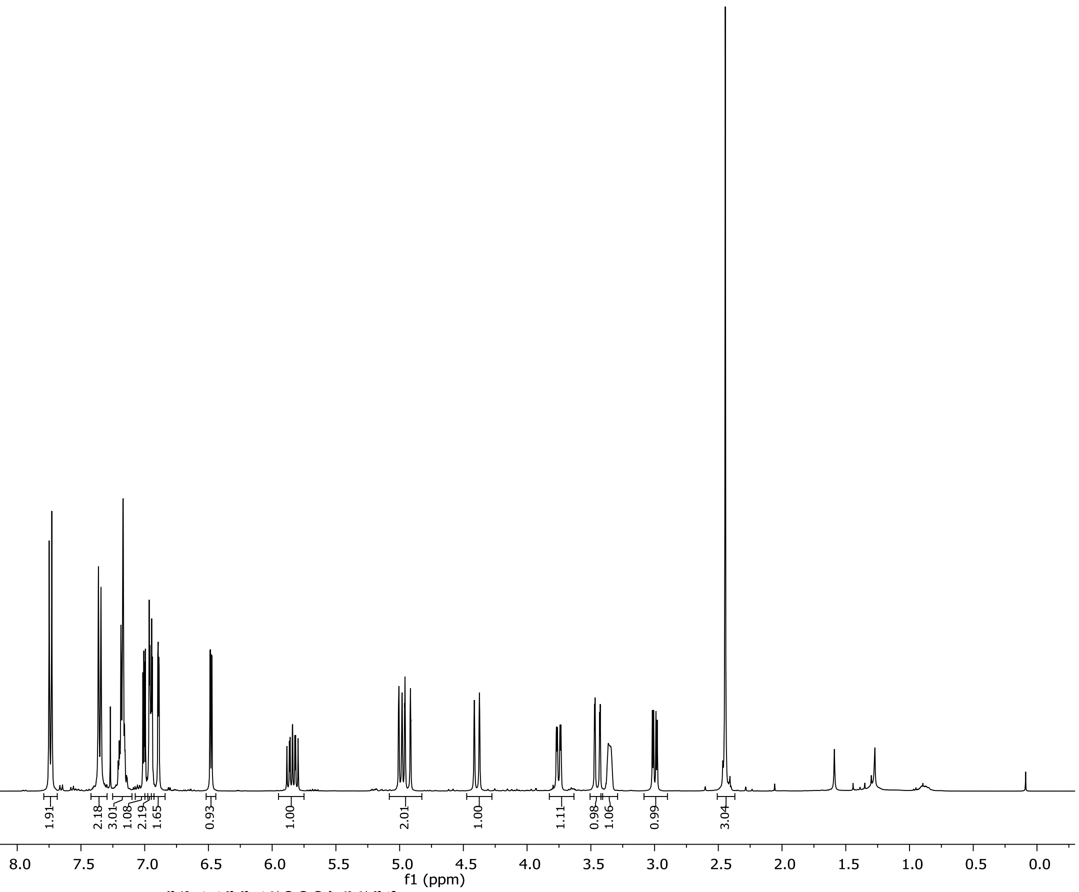
1.91
2.18
3.01
1.08
2.19
1.65
0.93
1.00
2.01
1.00
1.11
0.98
1.06
0.99
3.04
9.5 9.0 8.5 8.0 7.5 7.0 6.5 6.0 5.5 5.0 4.5 4.0 3.5 3.0 2.5 2.0 1.5 1.0 0.5 0.0
f1 (ppm)

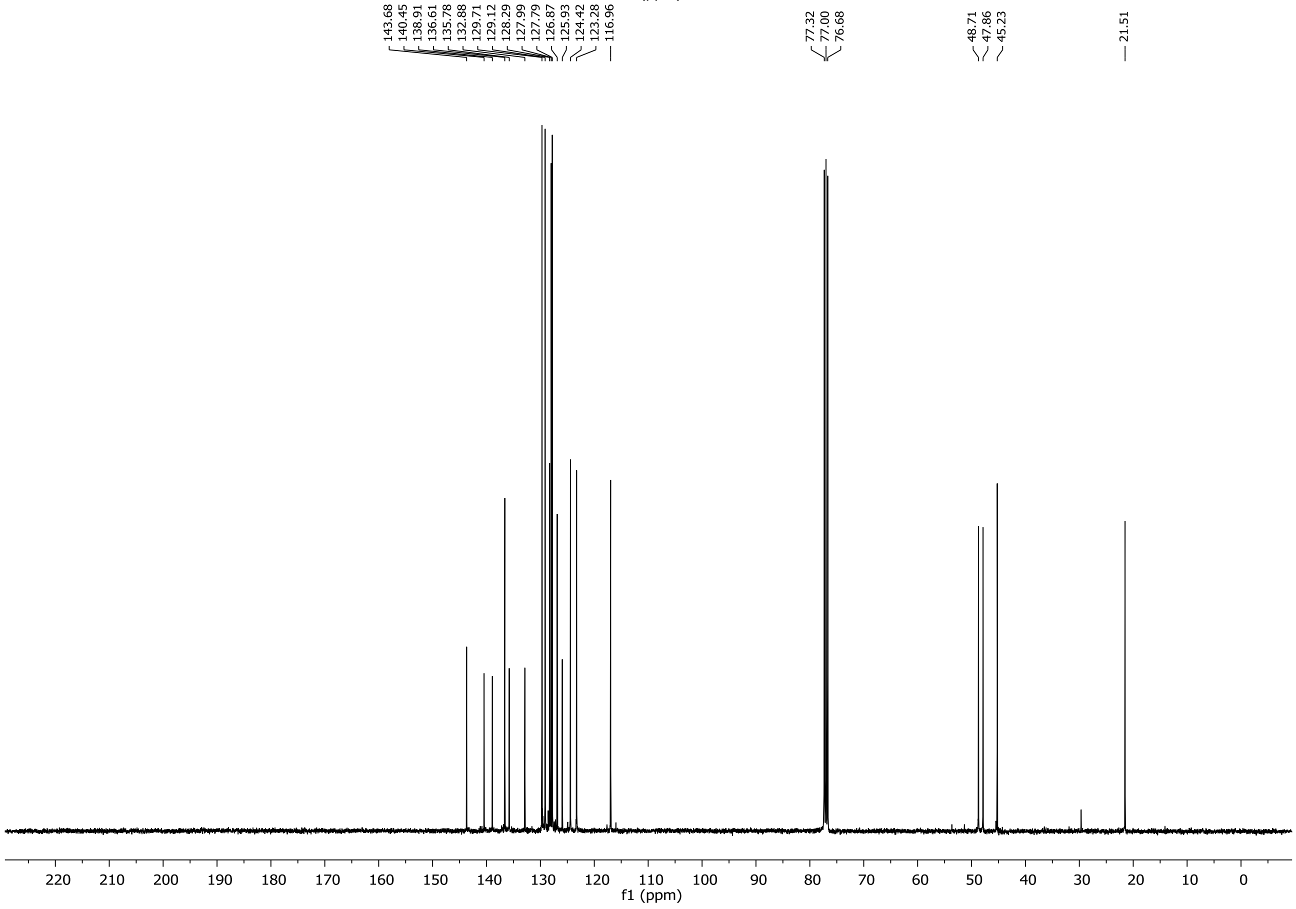
143.68
140.45
138.91
136.61
135.78
132.88
129.71
129.12
128.29
127.99
127.79
126.87
125.93
124.42
123.28
116.96
77.32
77.00
76.68
48.71
47.86
45.23
21.51
220 210 200 190 180 170 160 150 140 130 120 110 100 90 80 70 60 50 40 30 20 10 0
f1 (ppm)

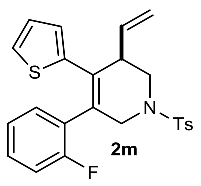
S
N
Ts
F

2m

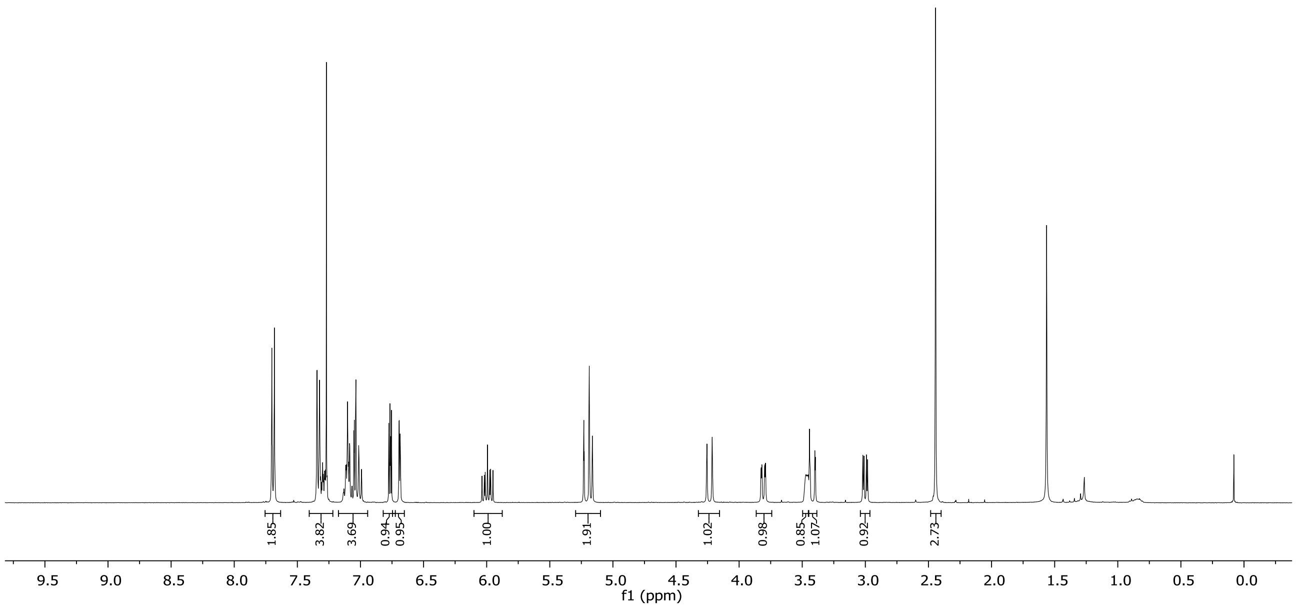
1.85
3.82
3.69
0.94
0.95
1.00
1.91
1.02
0.98
0.85
1.07
0.92
2.73
9.5 9.0 8.5 8.0 7.5 7.0 6.5 6.0 5.5 5.0 4.5 4.0 3.5 3.0 2.5 2.0 1.5 1.0 0.5 0.0
f1 (ppm)

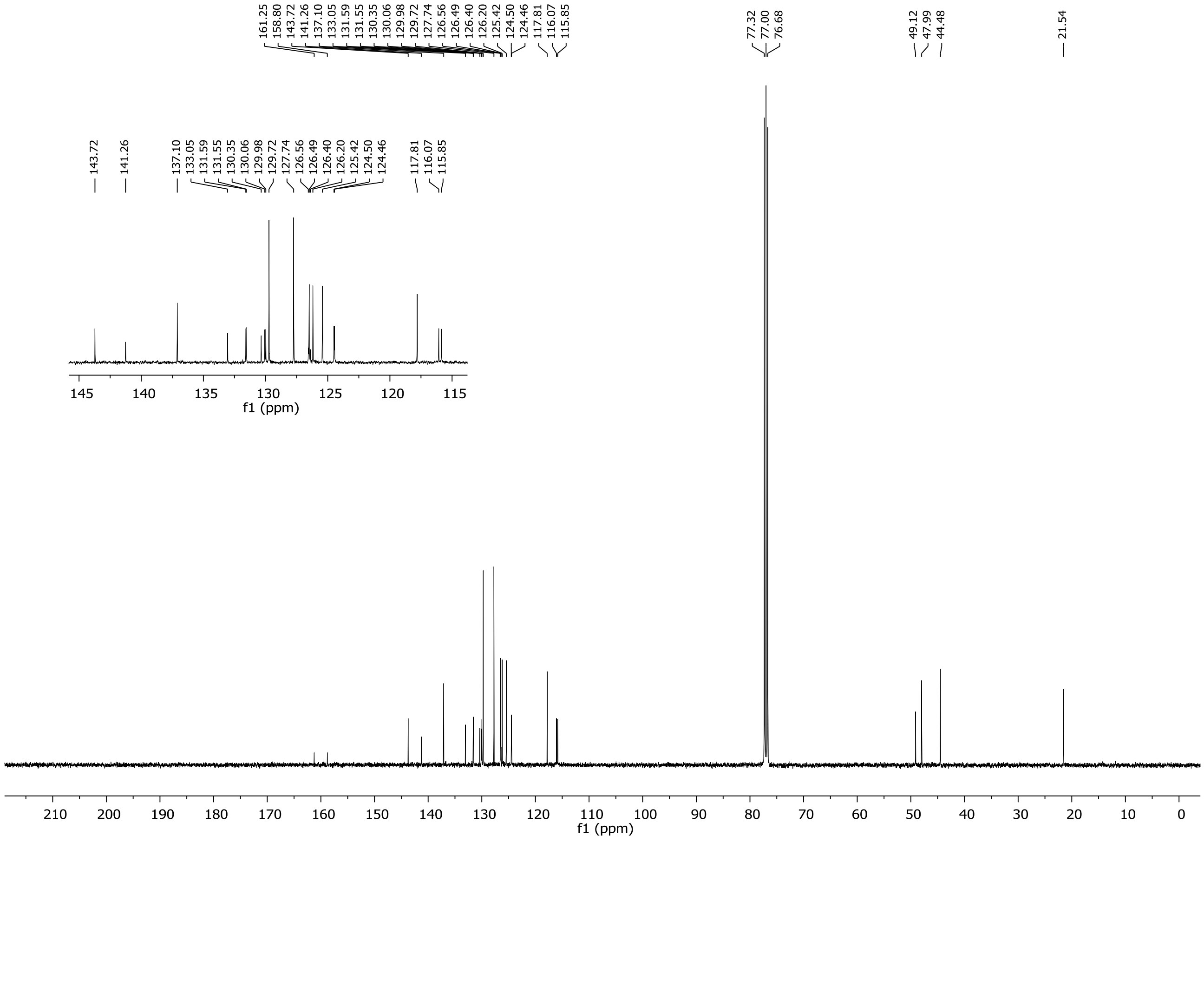
161.25
158.80
143.72
141.26
137.10
133.05
131.59
131.55
130.35
130.06
129.98
129.72
127.74
126.56
126.49
126.40
126.20
125.42
124.50
124.46
117.81
116.07
115.85
77.32
77.00
76.68
49.12
47.99
44.48
21.54
145 140 135 130 125 120 115
f1 (ppm)
210 200 190 180 170 160 150 140 130 120 110 100 90 80 70 60 50 40 30 20 10 0
f1 (ppm)

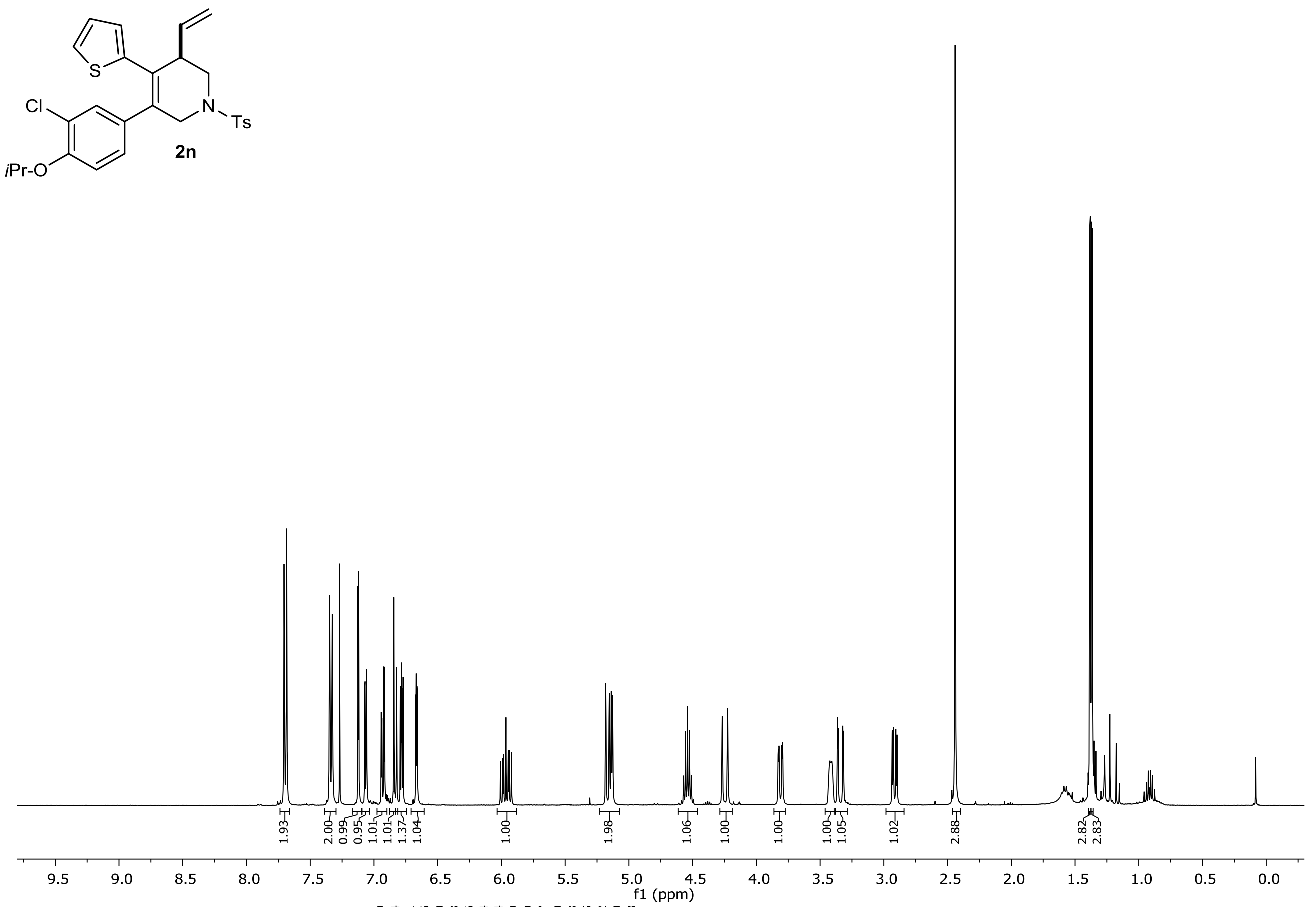
Cl
iPr-O
S
N
Ts
2n
1.93
2.00
0.99
0.95
1.01
1.01
1.37
1.04
1.00
1.98
1.06
1.00
1.00
1.00
1.05
1.02
2.88
2.82
2.83
f1 (ppm)

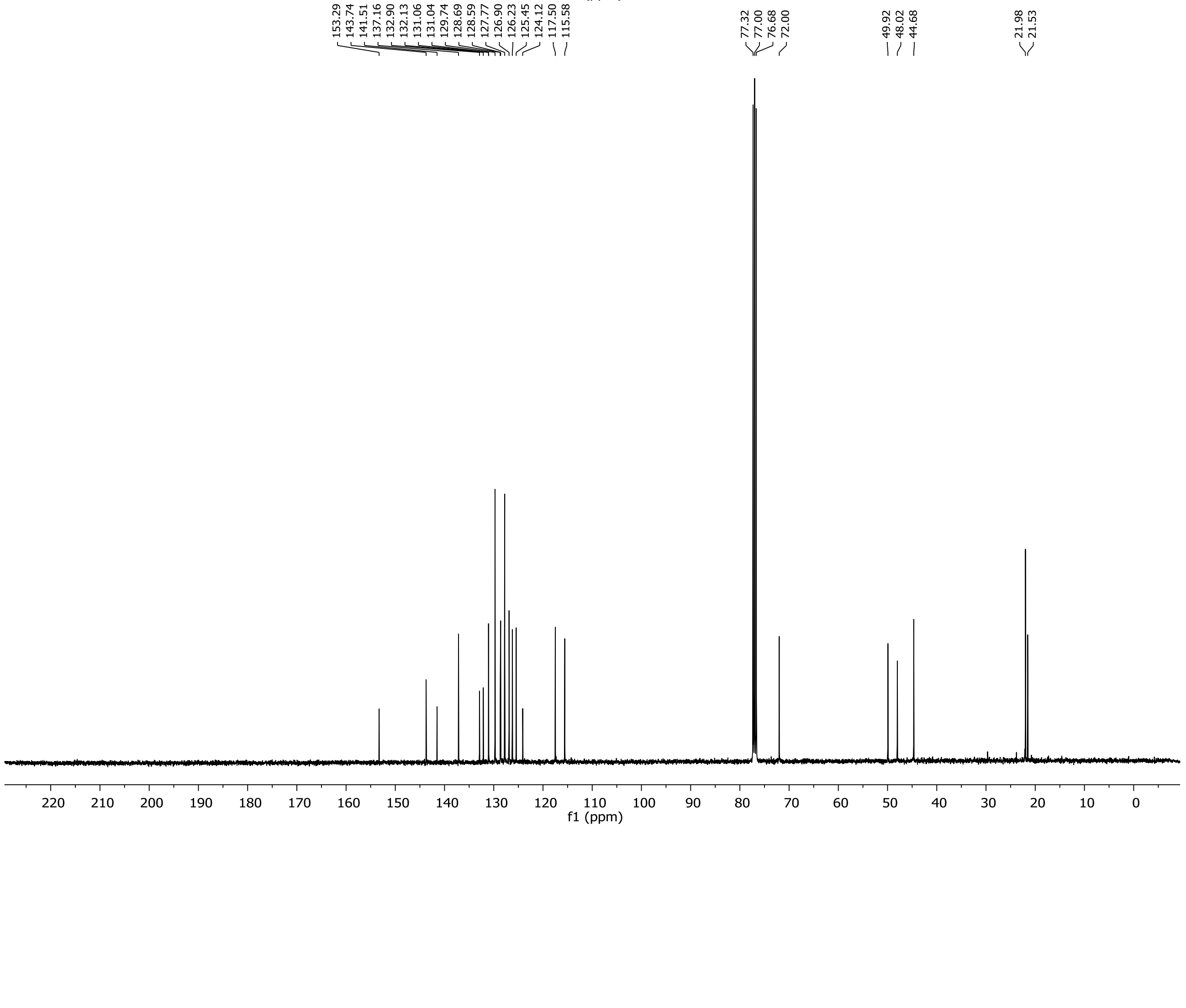
153.29
143.74
141.51
137.16
132.90
132.13
131.06
131.04
129.74
128.69
128.59
127.77
126.90
126.23
125.45
124.12
117.50
115.58
77.32
77.00
76.68
72.00
49.92
48.02
44.68
21.98
21.53
f1 (ppm)

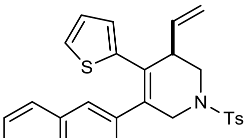
S
N
Ts

2o

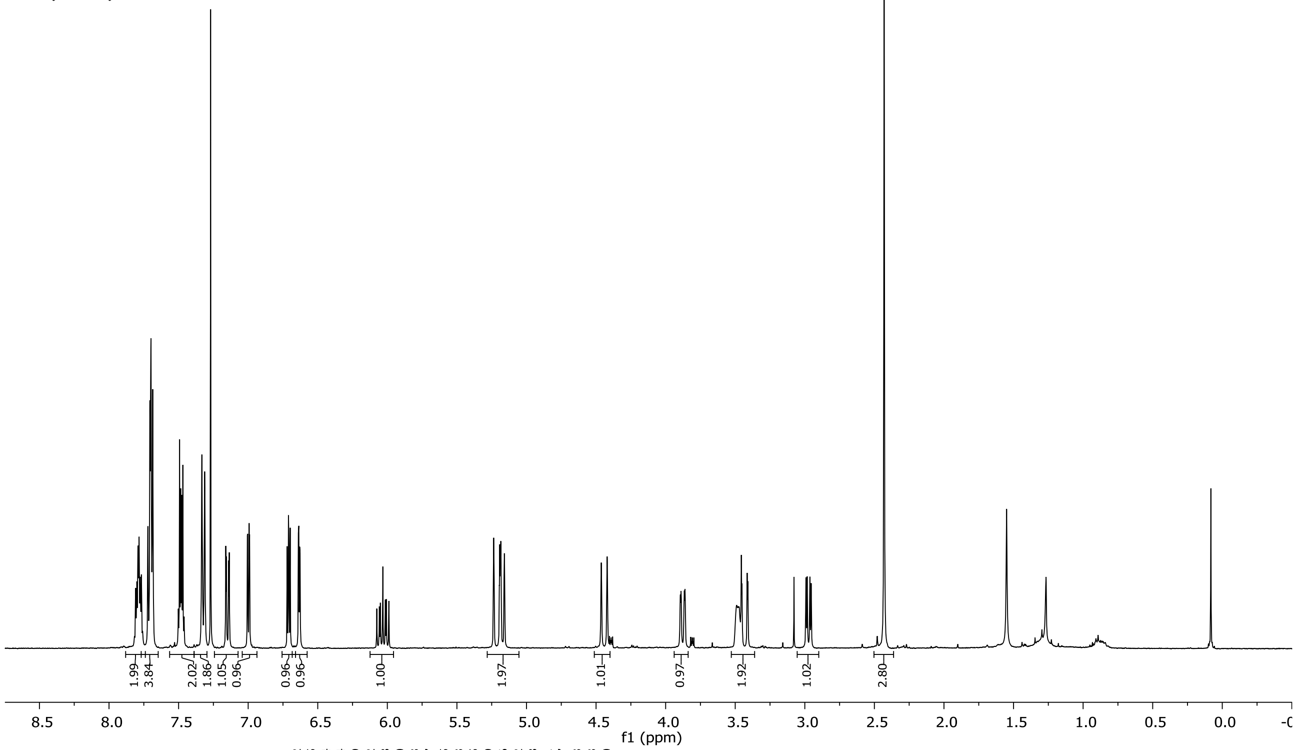
1.99
3.84
2.02
1.86
1.05
0.96
0.96
0.96
1.00
1.97
1.01
0.97
1.92
1.02
2.80
8.5 8.0 7.5 7.0 6.5 6.0 5.5 5.0 4.5 4.0 3.5 3.0 2.5 2.0 1.5 1.0 0.5 0.0
f1 (ppm)

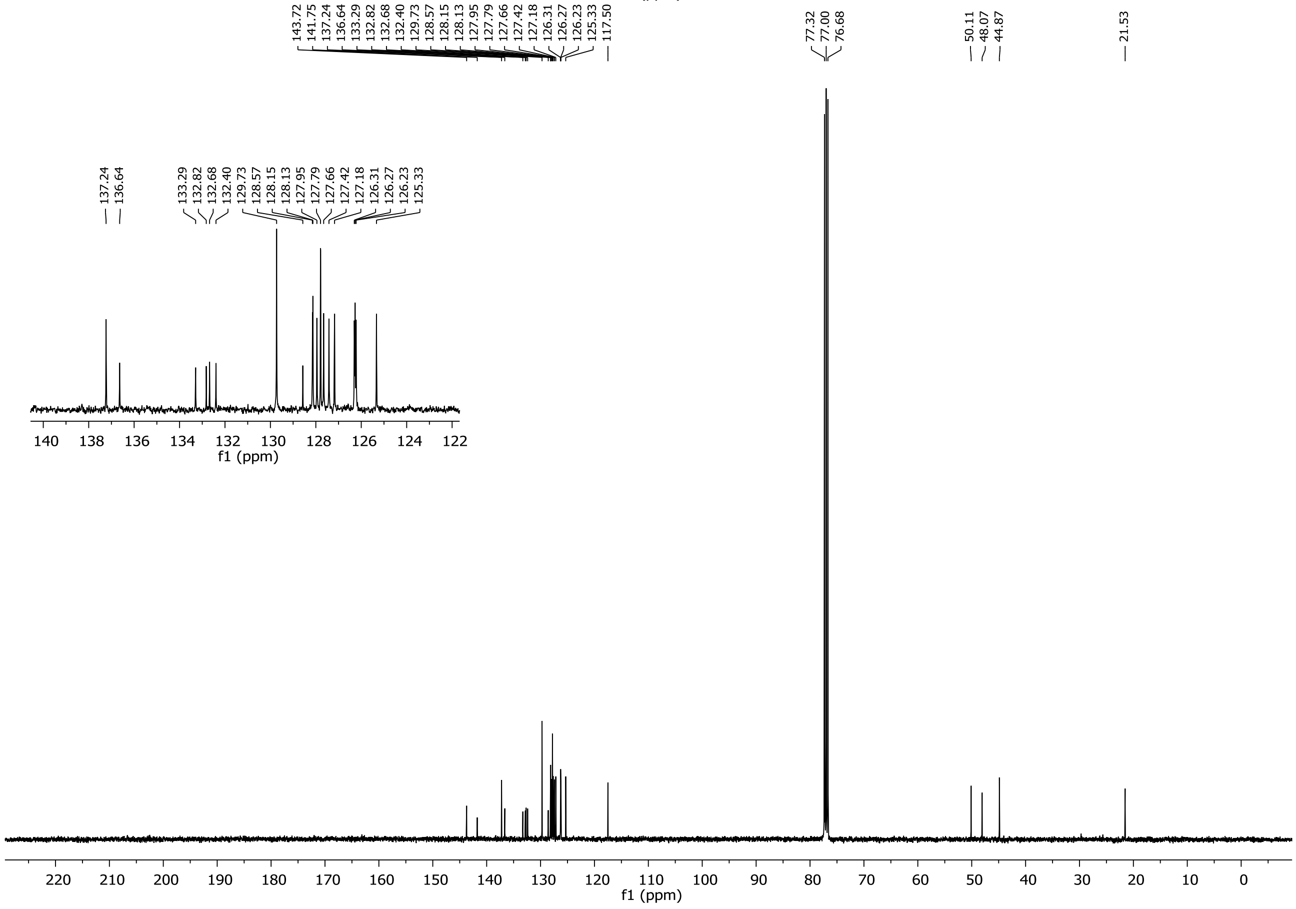
143.72
141.75
137.24
136.64
133.29
132.82
132.68
132.40
129.73
128.57
128.15
128.13
127.95
127.79
127.66
127.42
127.18
126.31
126.27
126.23
125.33
117.50
77.32
77.00
76.68
50.11
48.07
44.87
21.53
137.24
136.64
133.29
132.82
132.68
132.40
129.73
128.57
128.15
128.13
127.95
127.79
127.66
127.42
127.18
126.31
126.27
126.23
125.33
140 138 136 134 132 130 128 126 124 122
f1 (ppm)
220 210 200 190 180 170 160 150 140 130 120 110 100 90 80 70 60 50 40 30 20 10 0
f1 (ppm)

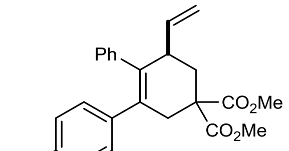
Ph
CO_2Me
CO_2Me
Me

2p

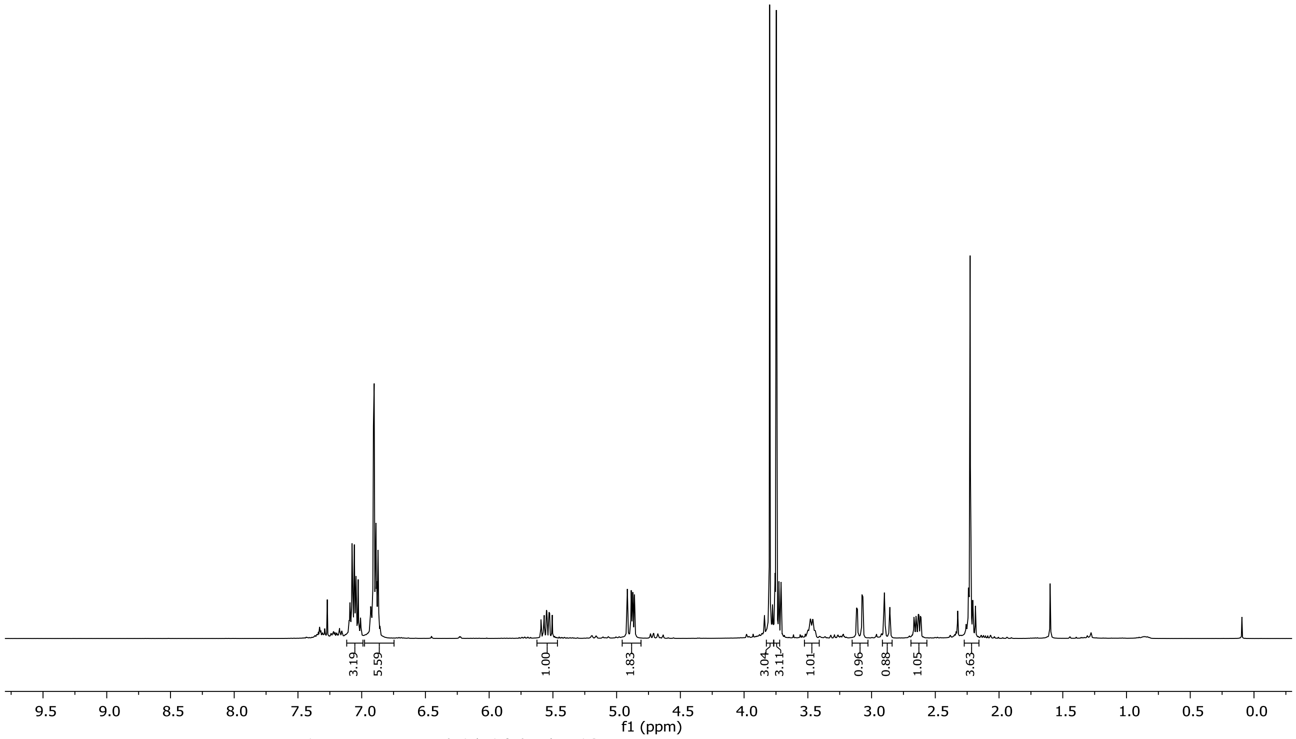
3.19
5.59
1.00
1.83
3.04
3.11
1.01
0.96
0.88
1.05
3.63
9.5 9.0 8.5 8.0 7.5 7.0 6.5 6.0 5.5 5.0 4.5 4.0 3.5 3.0 2.5 2.0 1.5 1.0 0.5 0.0
f1 (ppm)

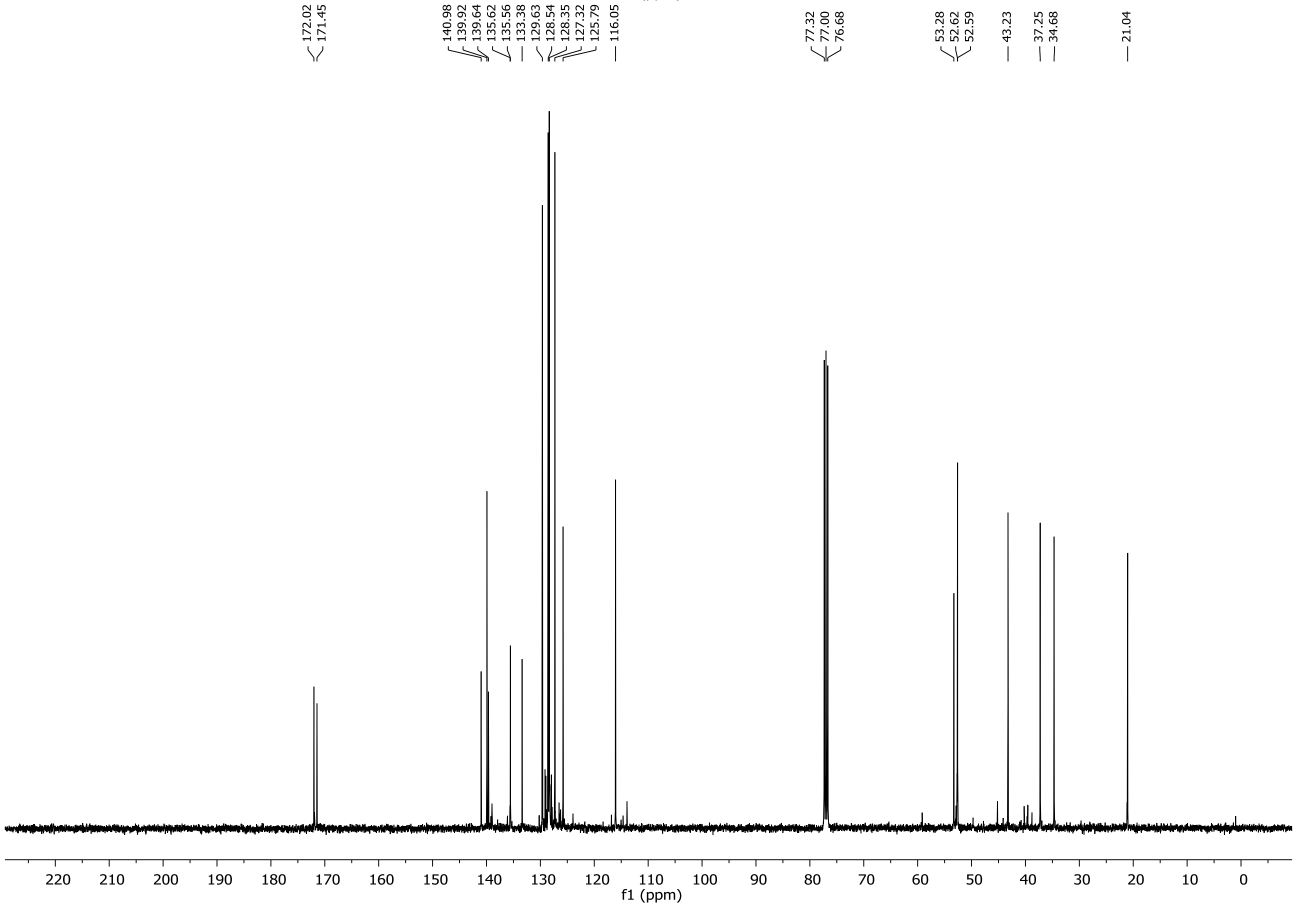
172.02
171.45
140.98
139.92
139.64
135.62
135.56
133.38
129.63
128.54
128.35
127.32
125.79
116.05
77.32
77.00
76.68
53.28
52.62
52.59
43.23
37.25
34.68
21.04
220 210 200 190 180 170 160 150 140 130 120 110 100 90 80 70 60 50 40 30 20 10 0
f1 (ppm)

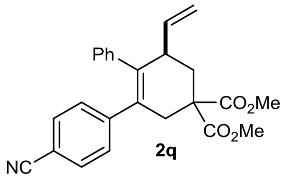
Ph
CO2Me
CO2Me
NC

2q

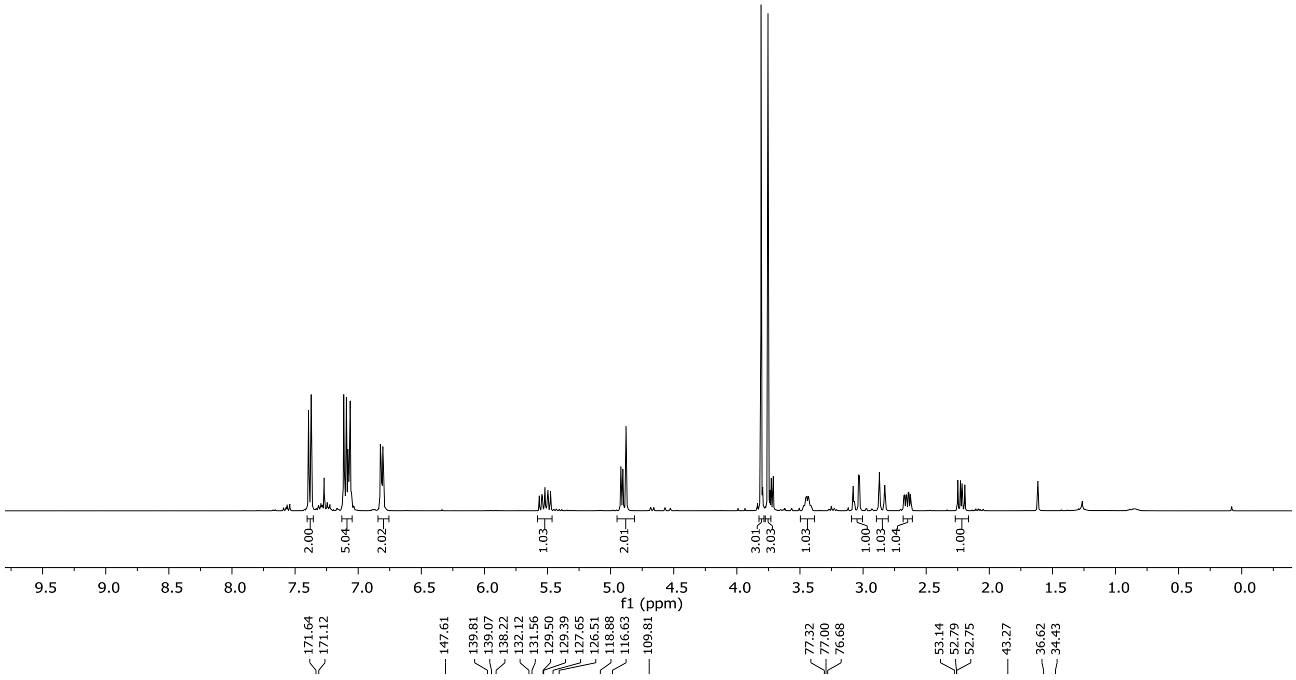
2.00
5.04
2.02
1.03
2.01
3.01
3.03
1.03
1.00
1.03
1.04
1.00
f1 (ppm)

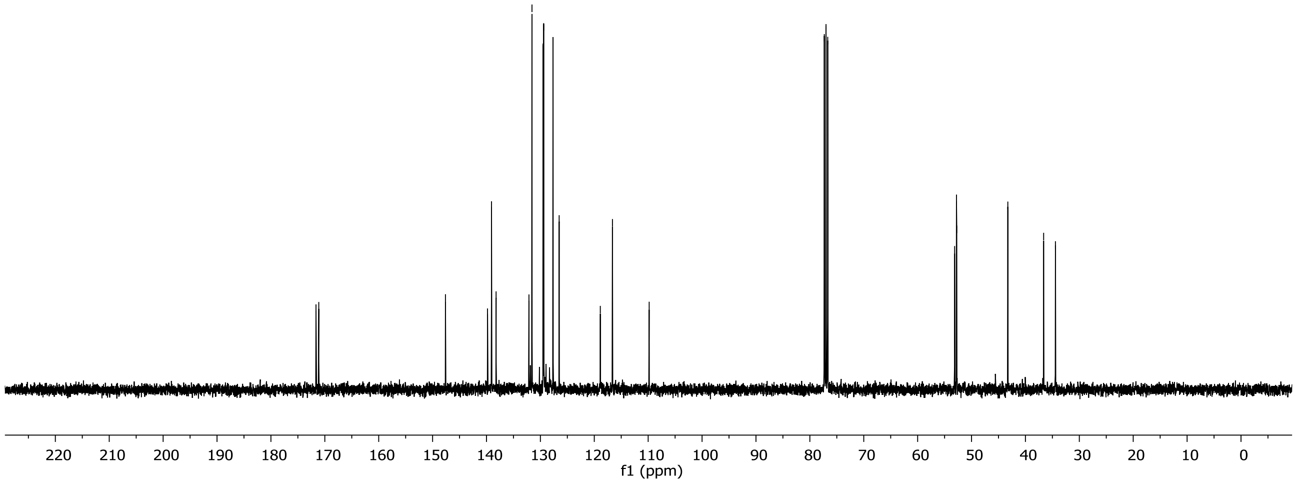
171.64
171.12
147.61
139.81
139.07
138.22
132.12
131.56
129.50
129.39
127.65
126.51
118.88
116.63
109.81
77.32
77.00
76.68
53.14
52.79
52.75
43.27
36.62
34.43
f1 (ppm)

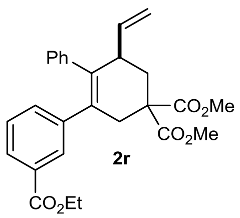
Ph
CO2Me
CO2Me
CO2Et

2r

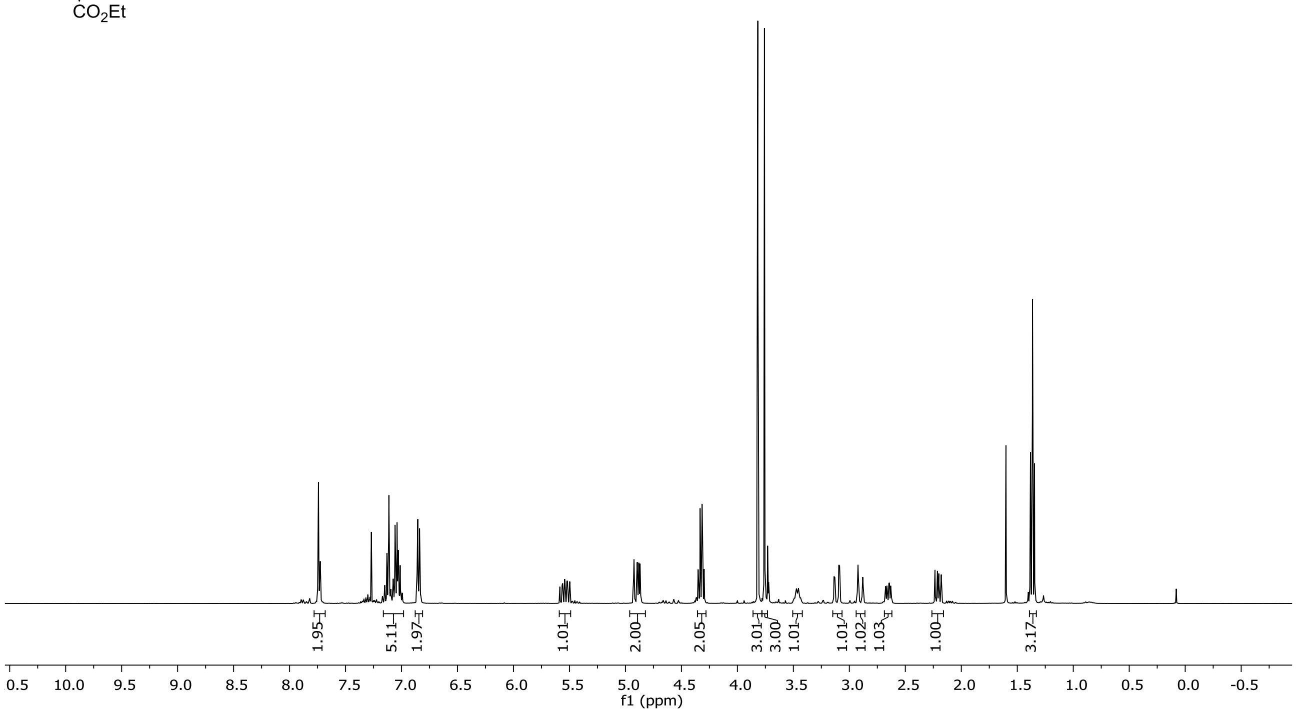
1.95
5.11
1.97
1.01
2.00
2.05
3.01
3.00
1.01
1.01
1.02
1.03
1.00
3.17
f1 (ppm)

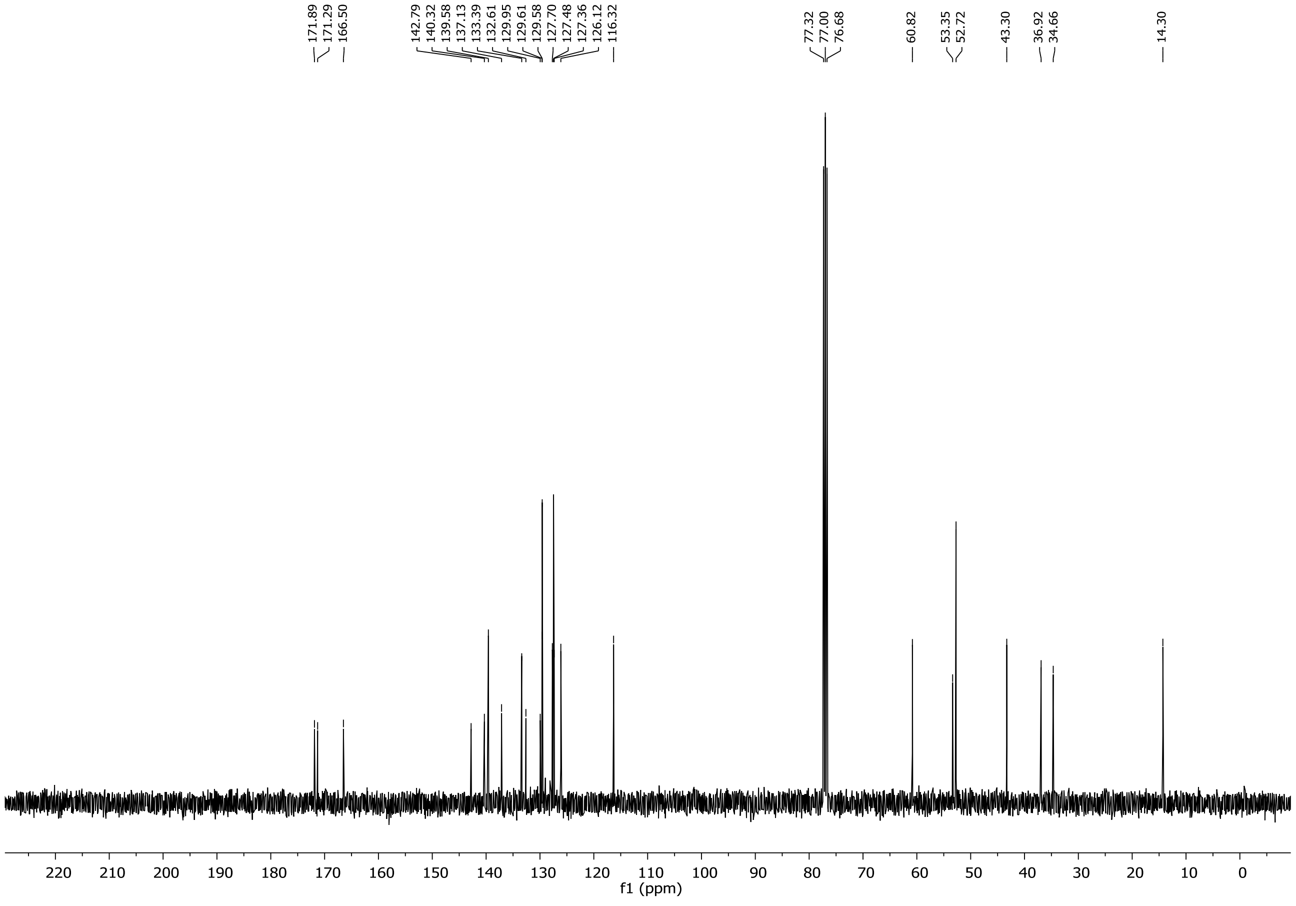
171.89
171.29
166.50
142.79
140.32
139.58
137.13
133.39
132.61
129.95
129.61
129.58
127.70
127.48
127.36
126.12
116.32
77.32
77.00
76.68
60.82
53.35
52.72
43.30
36.92
34.66
14.30
f1 (ppm)

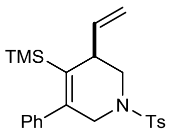
TMS
Ph
N
Ts

2s

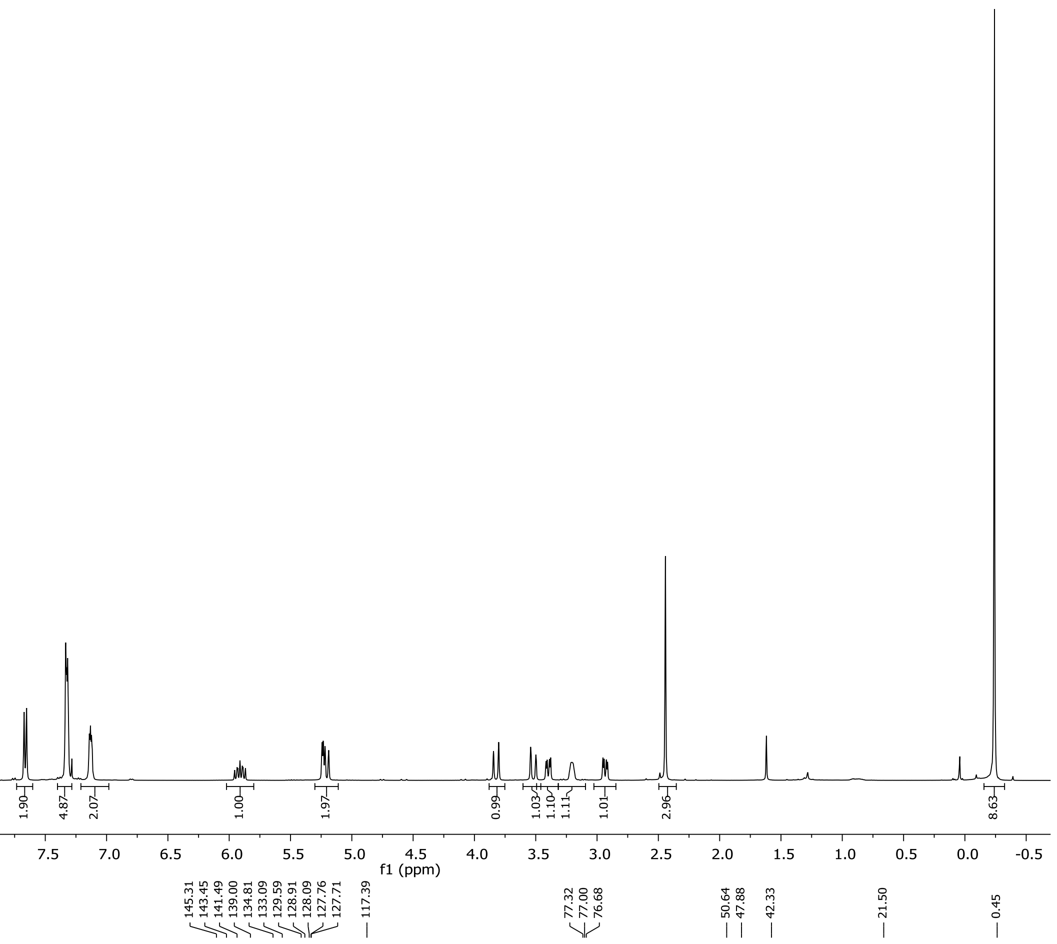
1.90
4.87
2.07
1.00
1.97
0.99
1.03
1.10
1.11
1.01
2.96
8.63
9.5 9.0 8.5 8.0 7.5 7.0 6.5 6.0 5.5 5.0 4.5 4.0 3.5 3.0 2.5 2.0 1.5 1.0 0.5 0.0 -0.5
f1 (ppm)

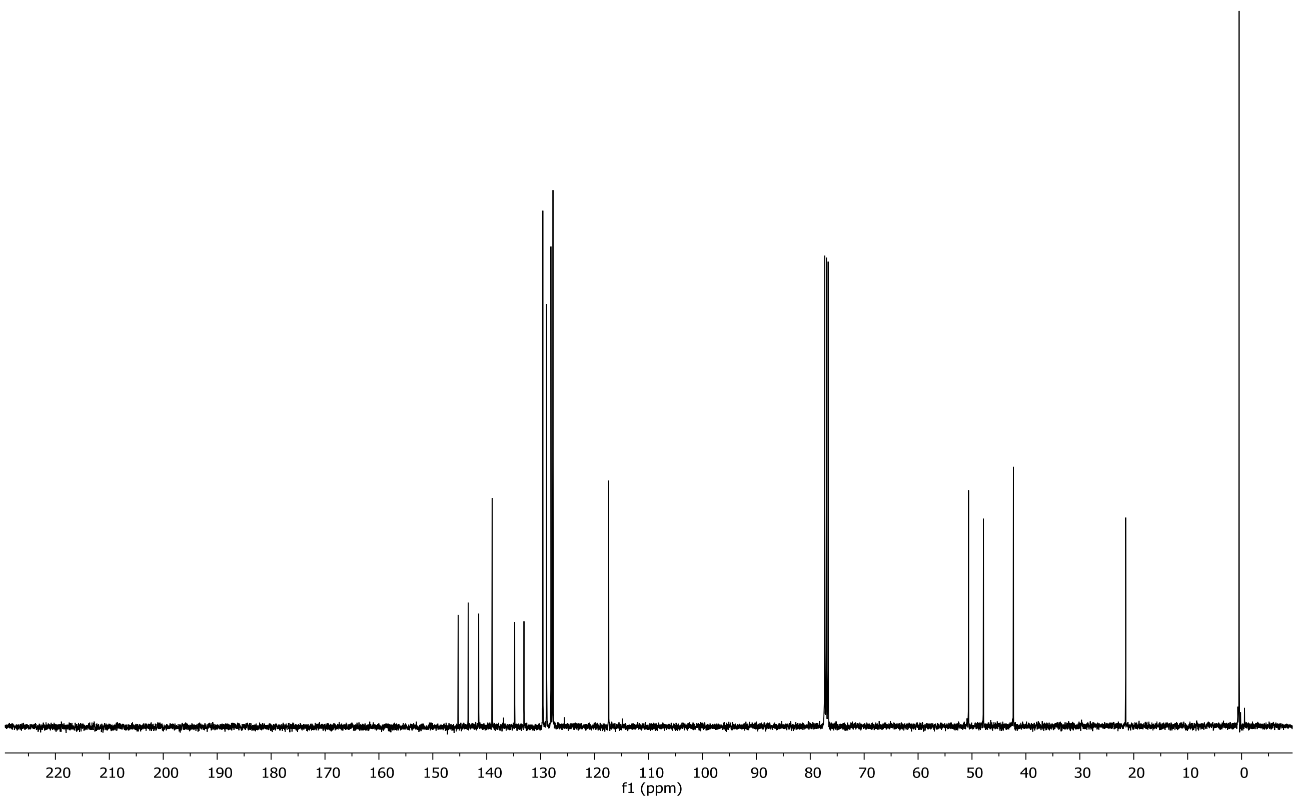
145.31
143.45
141.49
139.00
134.81
133.09
129.59
128.91
128.09
127.76
127.71
117.39
77.32
77.00
76.68
50.64
47.88
42.33
21.50
0.45
220 210 200 190 180 170 160 150 140 130 120 110 100 90 80 70 60 50 40 30 20 10 0
f1 (ppm)

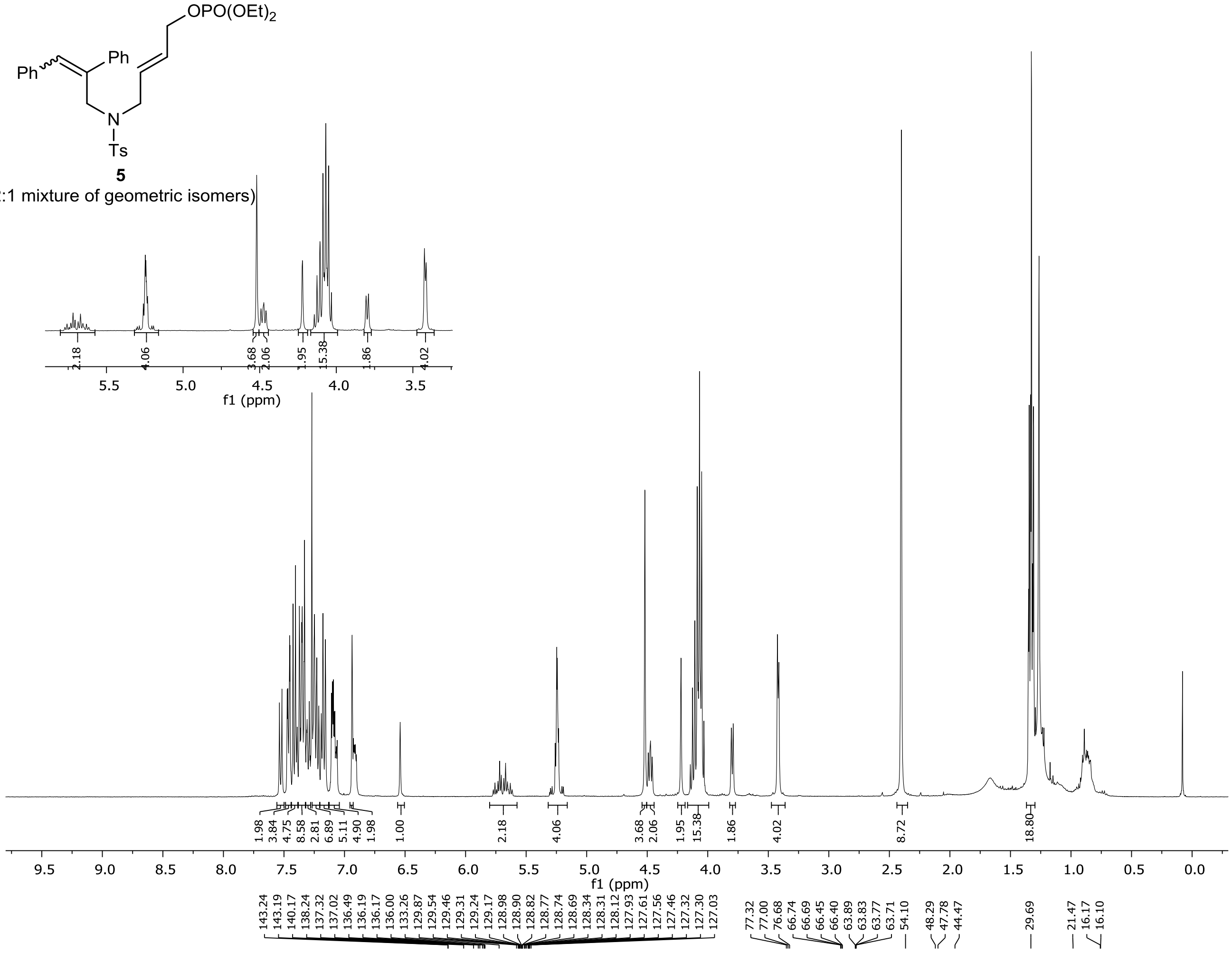

5
(2:1 mixture of geometric isomers)

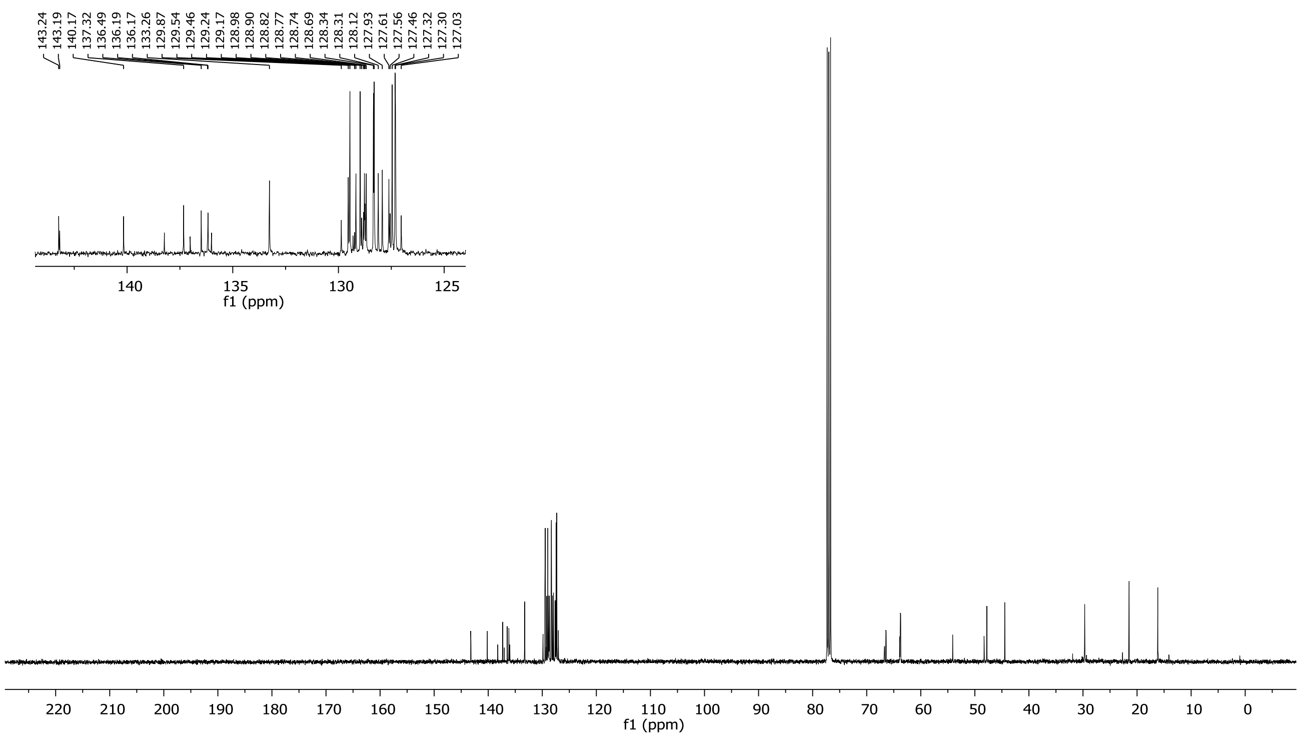

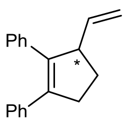
Ph
Ph
*

7

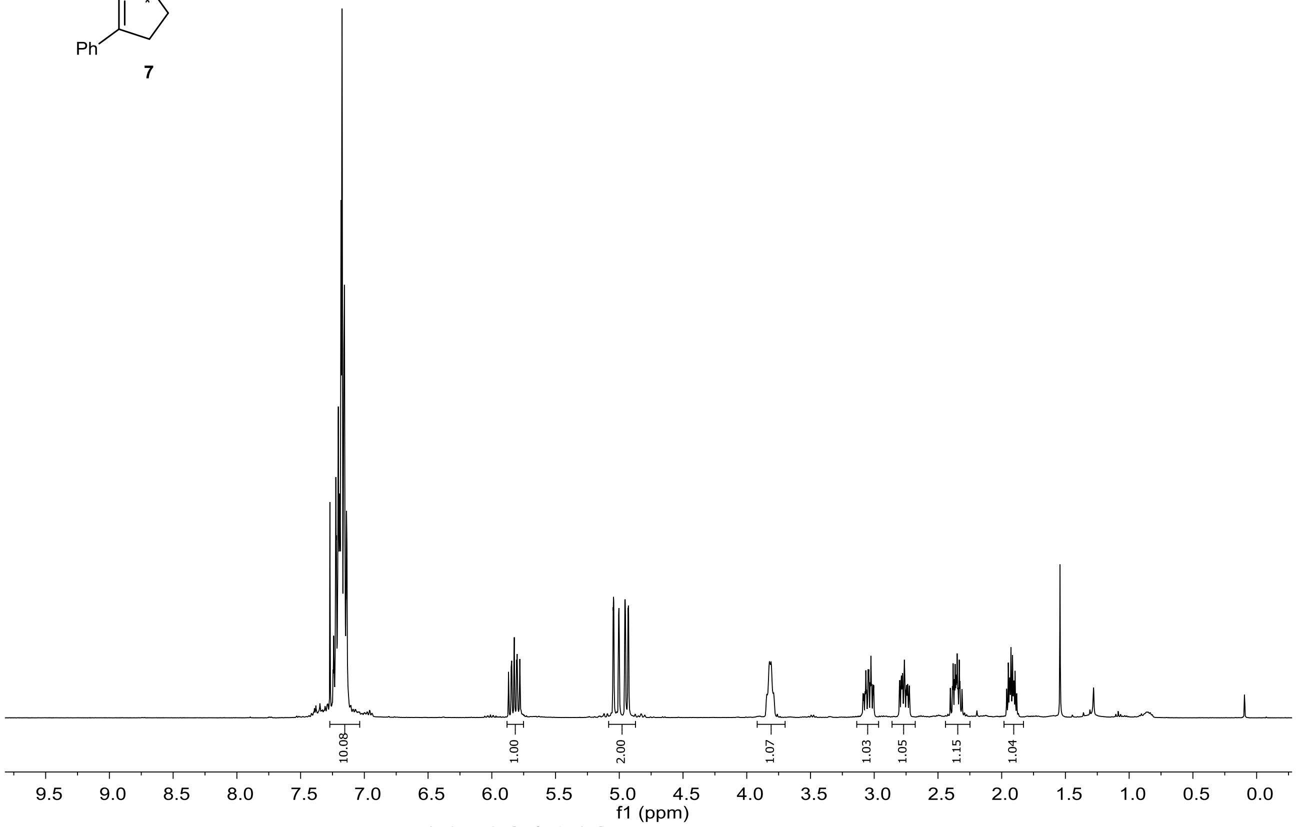
10.08
1.00
2.00
1.07
1.03
1.05
1.15
1.04
9.5 9.0 8.5 8.0 7.5 7.0 6.5 6.0 5.5 5.0 4.5 4.0 3.5 3.0 2.5 2.0 1.5 1.0 0.5 0.0
f1 (ppm)

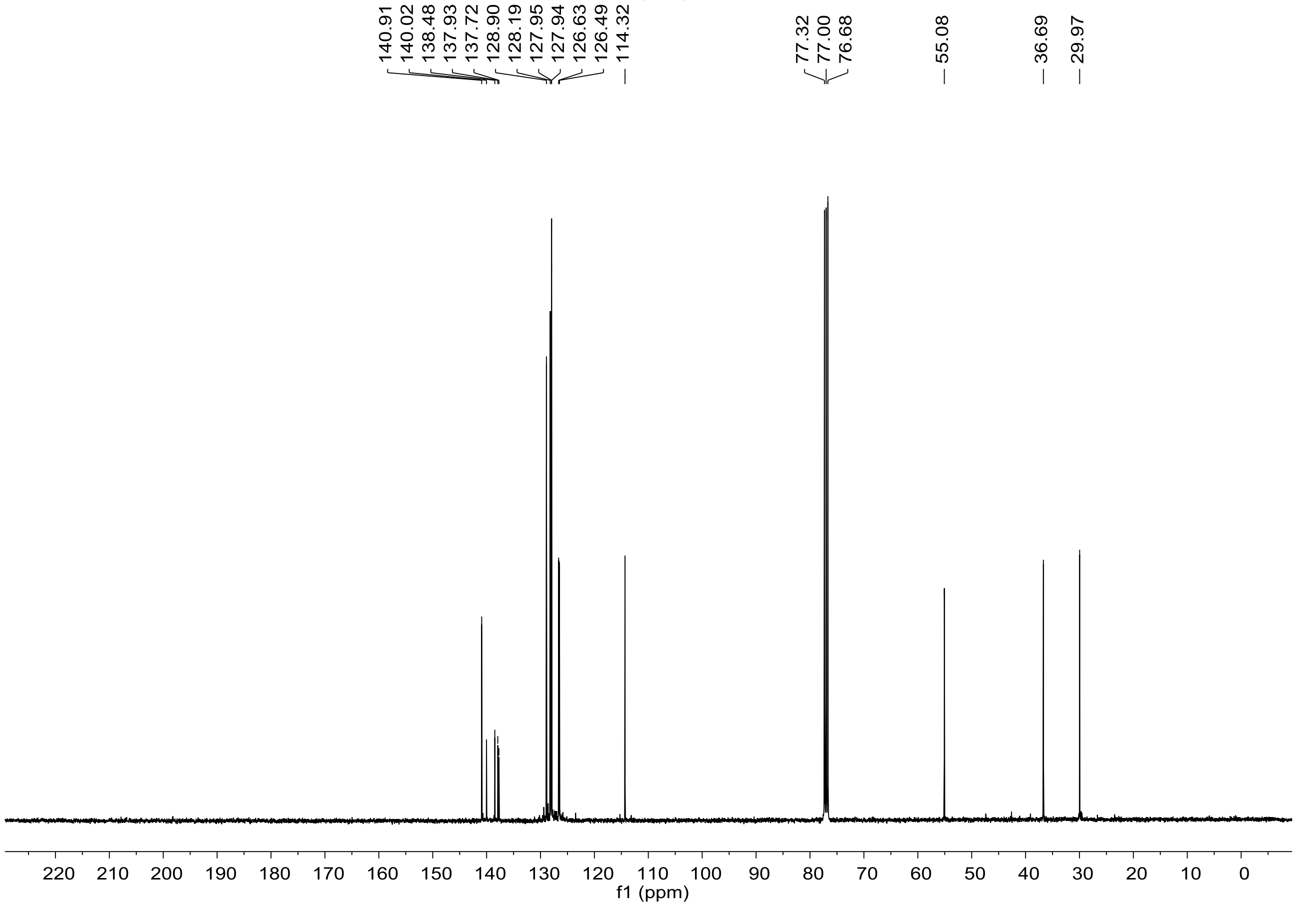
140.91
140.02
138.48
137.93
137.72
128.90
128.19
127.95
127.94
126.63
126.49
114.32
77.32
77.00
76.68
55.08
36.69
29.97
220 210 200 190 180 170 160 150 140 130 120 110 100 90 80 70 60 50 40 30 20 10 0
f1 (ppm)

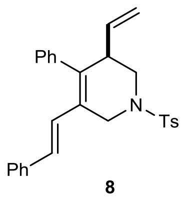
Ph
N
Ts
Ph

8

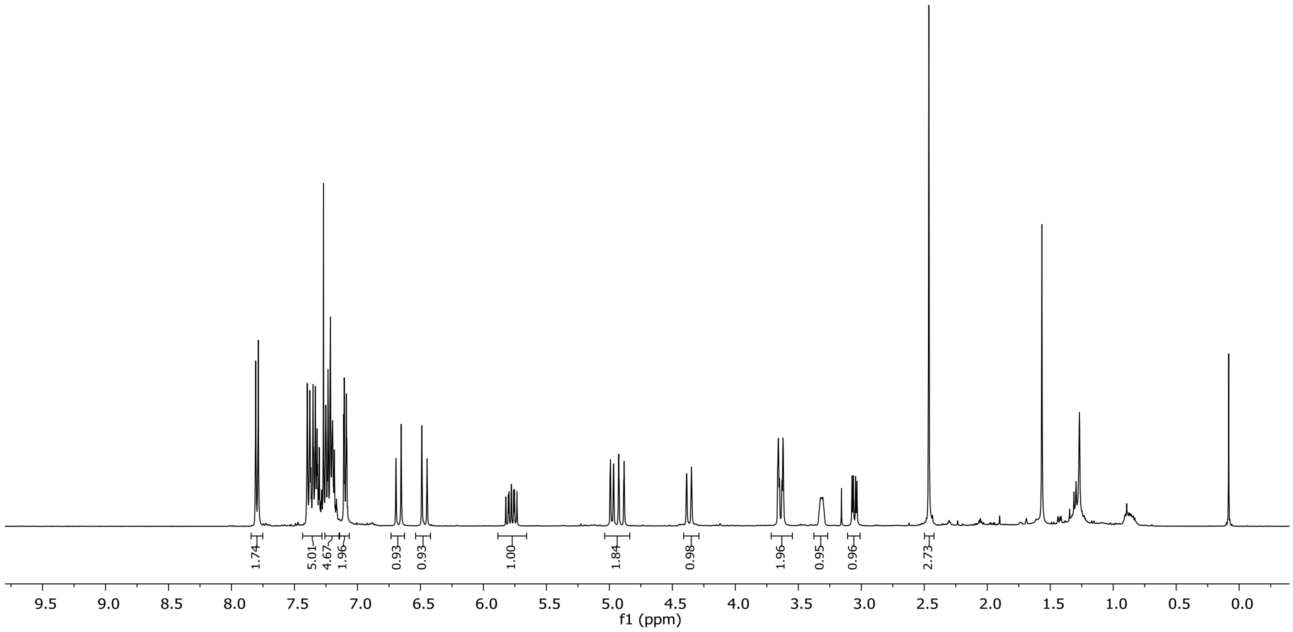
1.74
5.01
4.67
1.96
0.93
0.93
1.00
1.84
0.98
1.96
0.95
0.96
2.73
9.5 9.0 8.5 8.0 7.5 7.0 6.5 6.0 5.5 5.0 4.5 4.0 3.5 3.0 2.5 2.0 1.5 1.0 0.5 0.0
f1 (ppm)

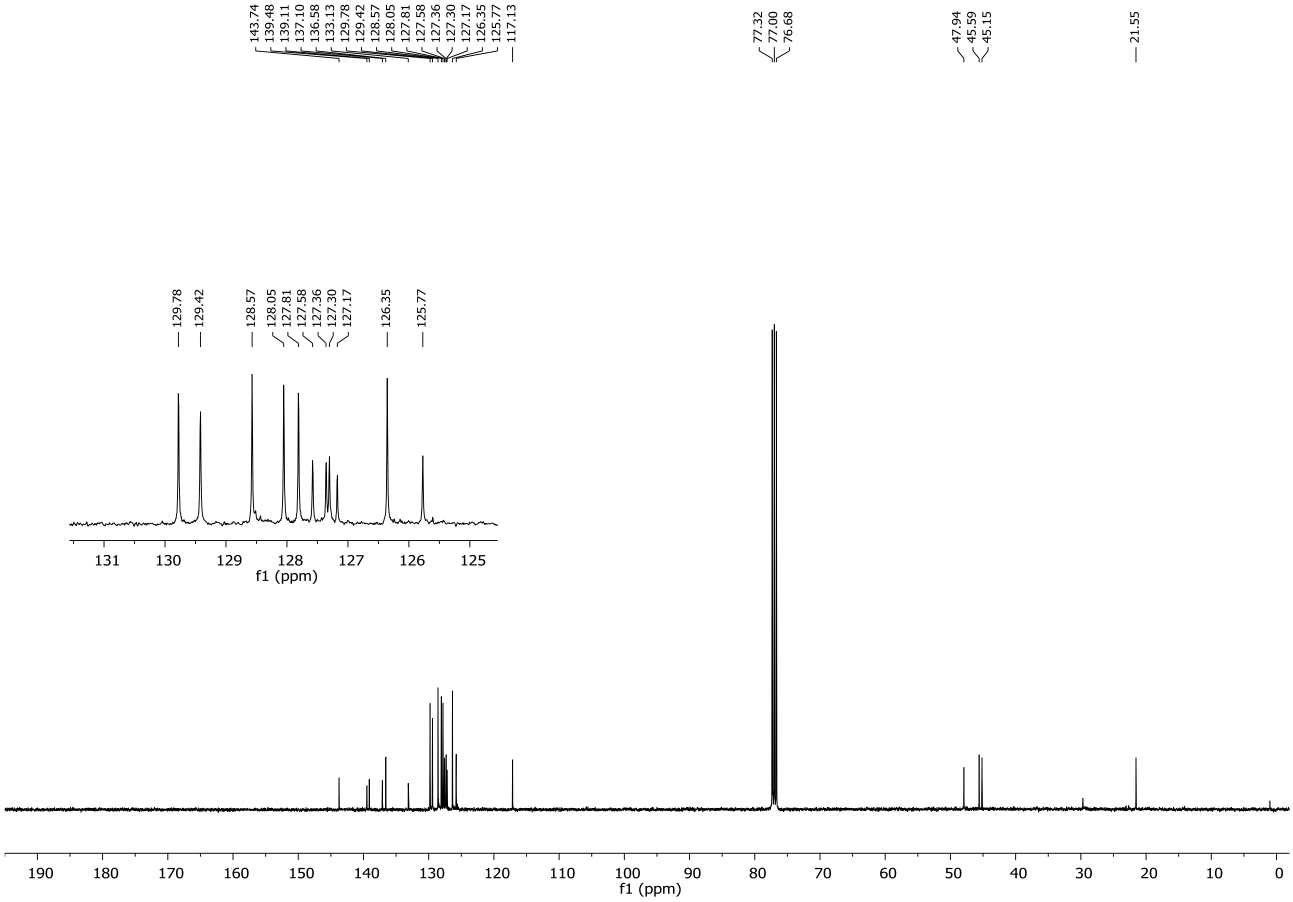
143.74
139.48
139.11
137.10
136.58
133.13
129.78
129.42
128.57
128.05
127.81
127.58
127.36
127.30
127.17
126.35
125.77
117.13
77.32
77.00
76.68
47.94
45.59
45.15
21.55
129.78
129.42
128.57
128.05
127.81
127.58
127.36
127.30
127.17
126.35
125.77
131 130 129 128 127 126 125
f1 (ppm)
190 180 170 160 150 140 130 120 110 100 90 80 70 60 50 40 30 20 10 0
f1 (ppm)

HPLC Traces

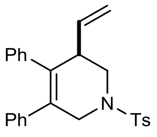

2a

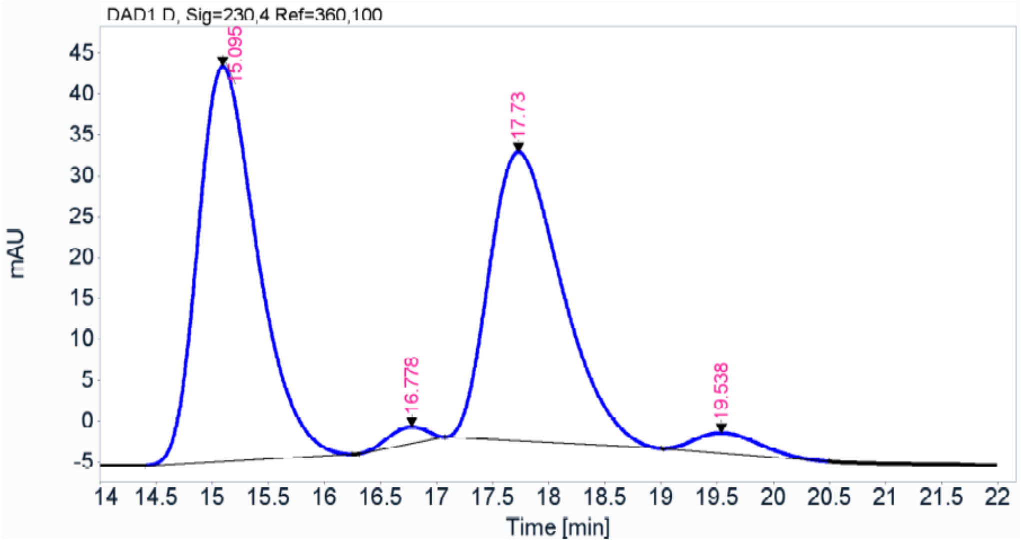

Signal:	DAD1 D, Sig=230,4 Ref=360,100				
RT [min]	Type	Width [min]	Area	Height	Area%
15.095	BB	0.5645	1773.271	48.2840	50.54
16.778	BB	0.3995	48.275	2.0148	1.38
17.730	BB	0.6949	1589.645	35.3305	45.30
19.538	BB	0.6154	97.647	2.4477	2.78

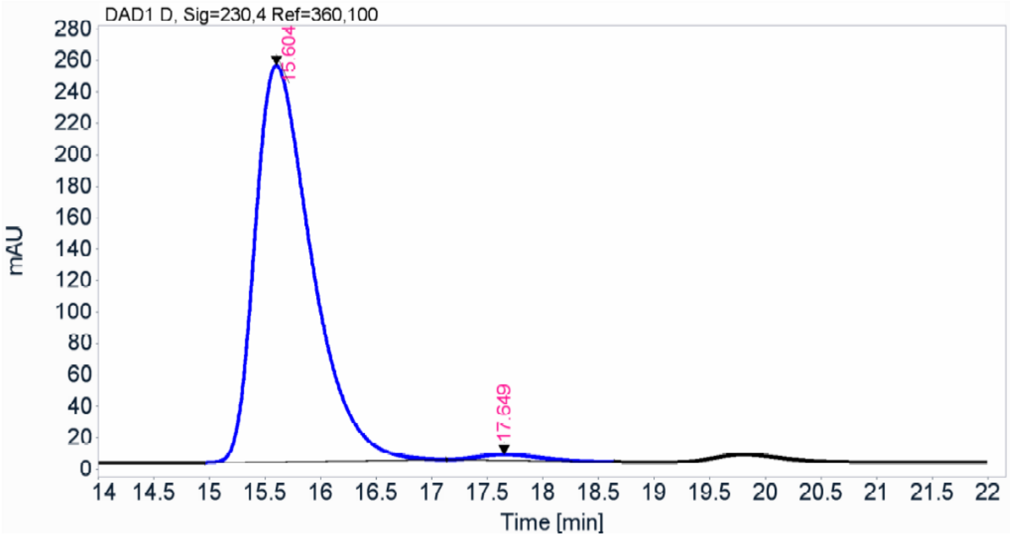

Signal:	DAD1 D, Sig=230,4 Ref=360,100				
RT [min]	Type	Width [min]	Area	Height	Area%
15.604	BB	0.5387	8871.138	252.0253	98.46
17.649	BB	0.5720	138.338	3.6515	1.54

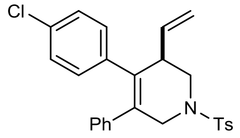

2b

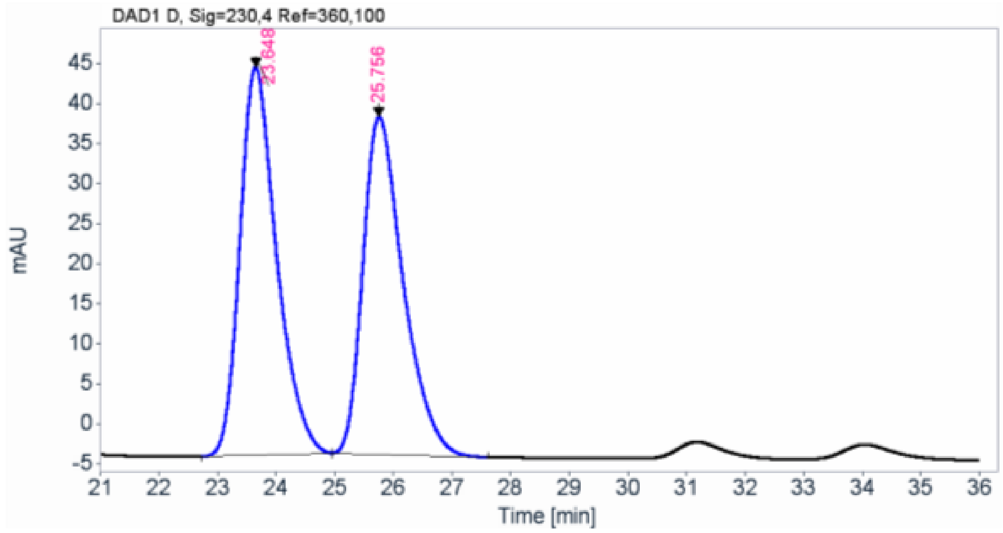

Signal:	DAD1 D, Sig=230,4 Ref=360,100				
RT [min]	Type	Width [min]	Area	Height	Area%
23.648	BB	0.6502	2103.302	48.4795	51.12
25.756	BB	0.7177	2011.269	42.2265	48.88

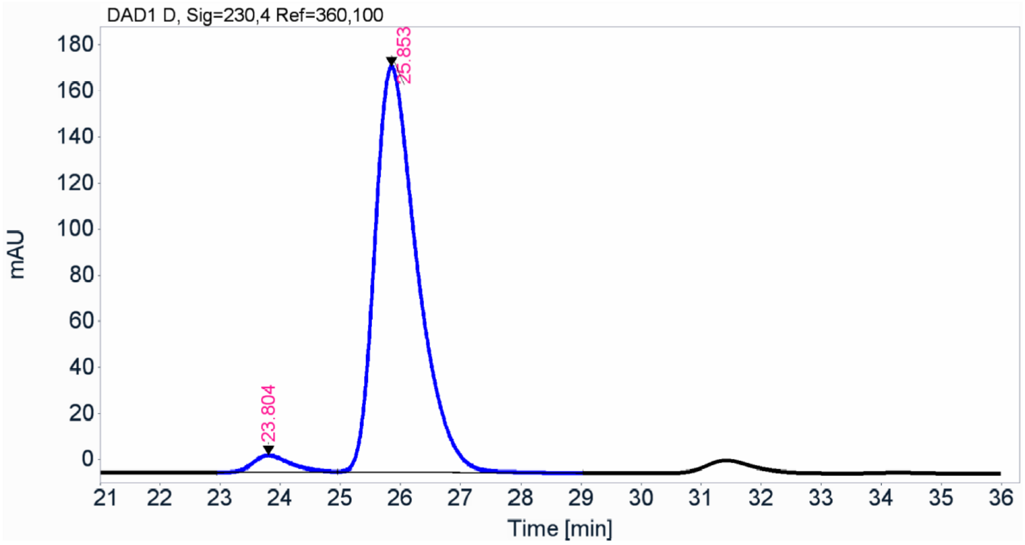

Signal:	DAD1 D, Sig=230,4 Ref=360,100				
RT [min]	Type	Width [min]	Area	Height	Area%
23.804	BB	0.6382	303.683	7.1975	3.42
25.853	BB	0.7353	8570.601	175.6333	96.58

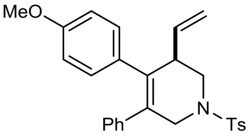

2c

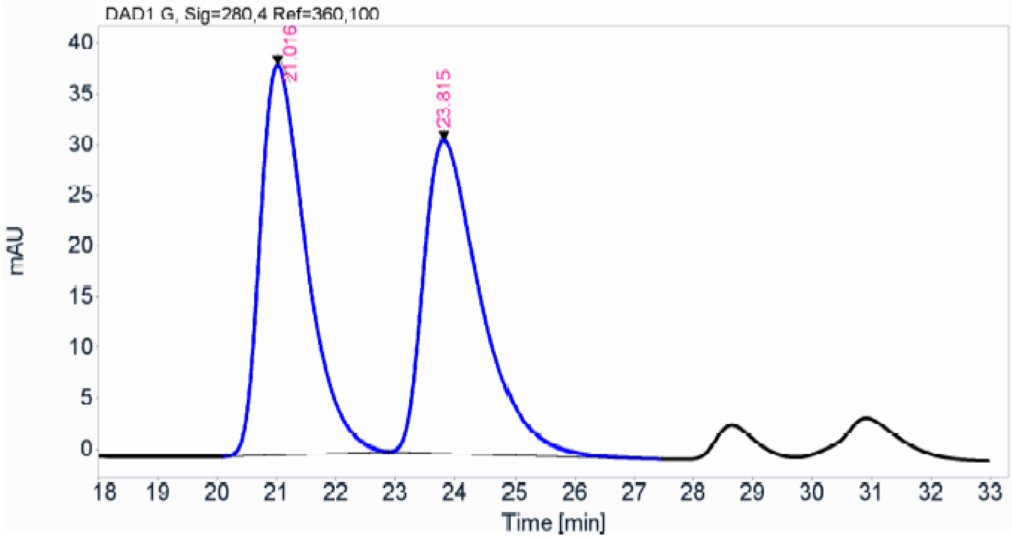

Signal:	DAD1 G, Sig=280,4 Ref=360,100				
RT [min]	Type	Width [min]	Area	Height	Area%
21.016	BB	0.8184	2055.044	38.3974	50.15
23.815	BB	0.9967	2042.585	30.8403	49.85

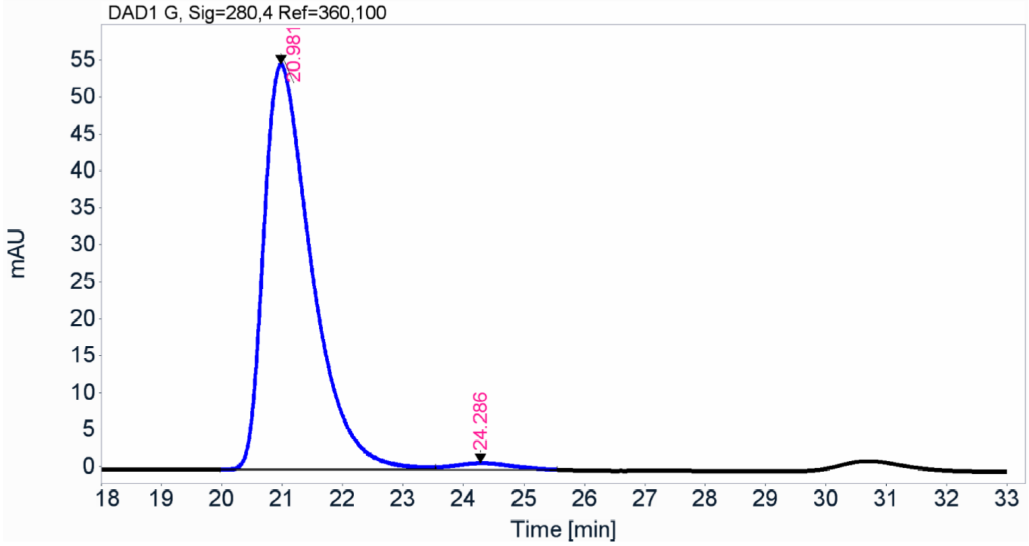

Signal:	DAD1 G, Sig=280,4 Ref=360,100				
RT [min]	Type	Width [min]	Area	Height	Area%
20.981	MF	0.9156	3007.832	54.7505	98.13
24.286	FM	1.1343	57.474	0.8445	1.87

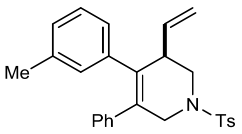

2d

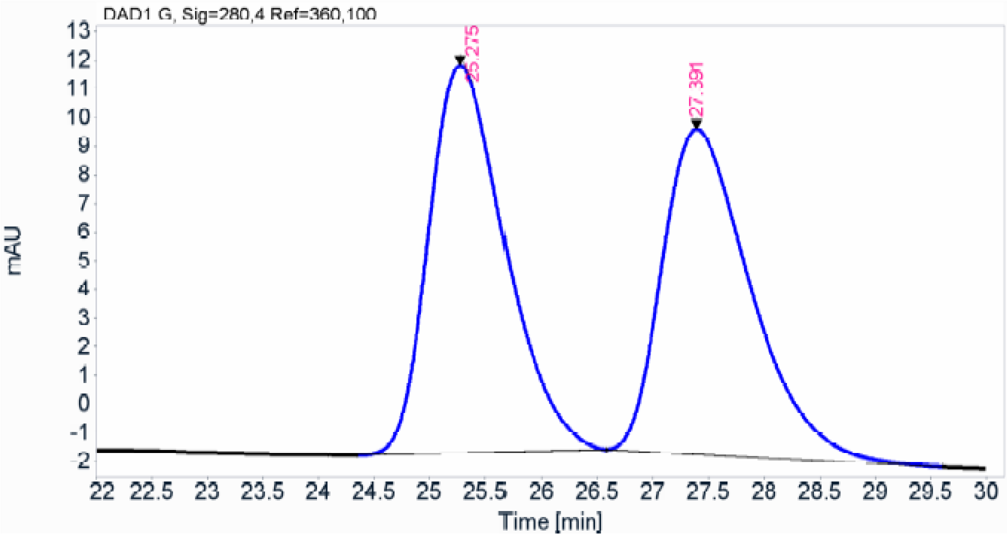

Signal: DAD1 G, Sig=280,4 Ref=360,100

RT [min]	Type	Width [min]	Area	Height	Area%
25.275	BB	0.7091	638.762	13.5231	50.51
27.391	BBA	0.8282	625.930	11.3367	49.49

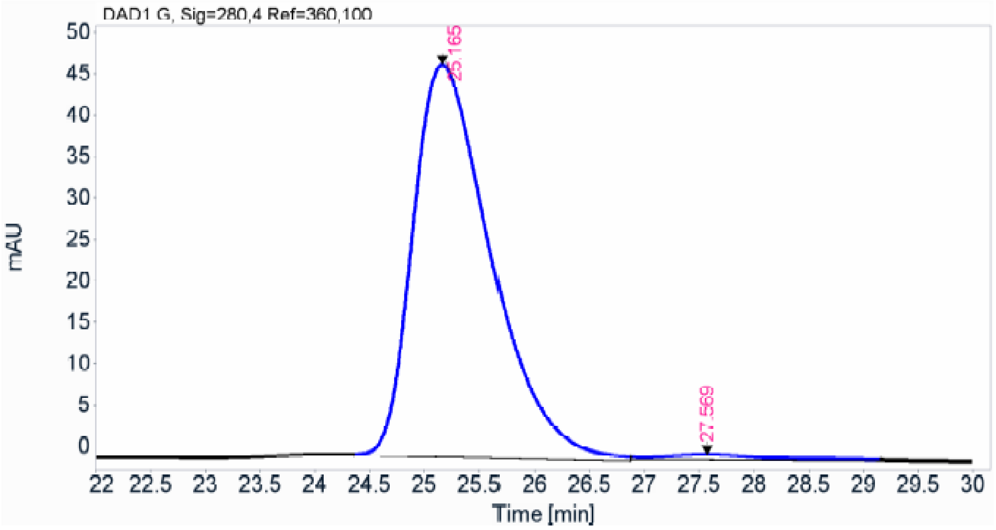

Signal: DAD1 G, Sig=280,4 Ref=360,100

RT [min]	Type	Width [min]	Area	Height	Area%
25.165	MM T	0.8250	2345.032	47.3730	97.96
27.569	MM T	1.0735	48.939	0.6597	2.04

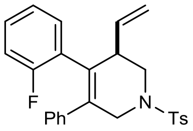

2e

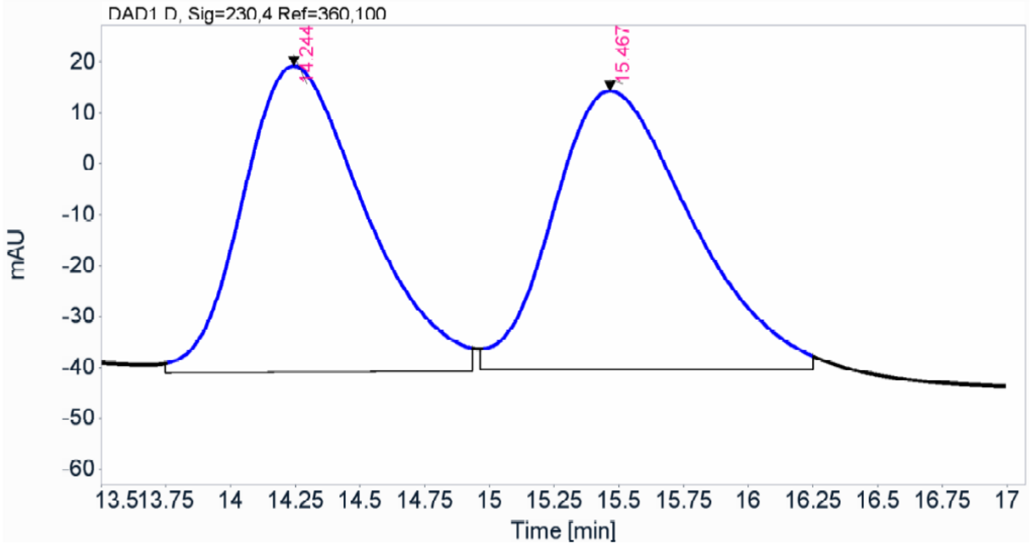

Signal:	DAD1 D, Sig=230,4 Ref=360,100				
RT [min]	Type	Width [min]	Area	Height	Area%
14.244	MM	0.5471	1976.302	60.2088	49.36
15.467	MM	0.6174	2027.482	54.7283	50.64

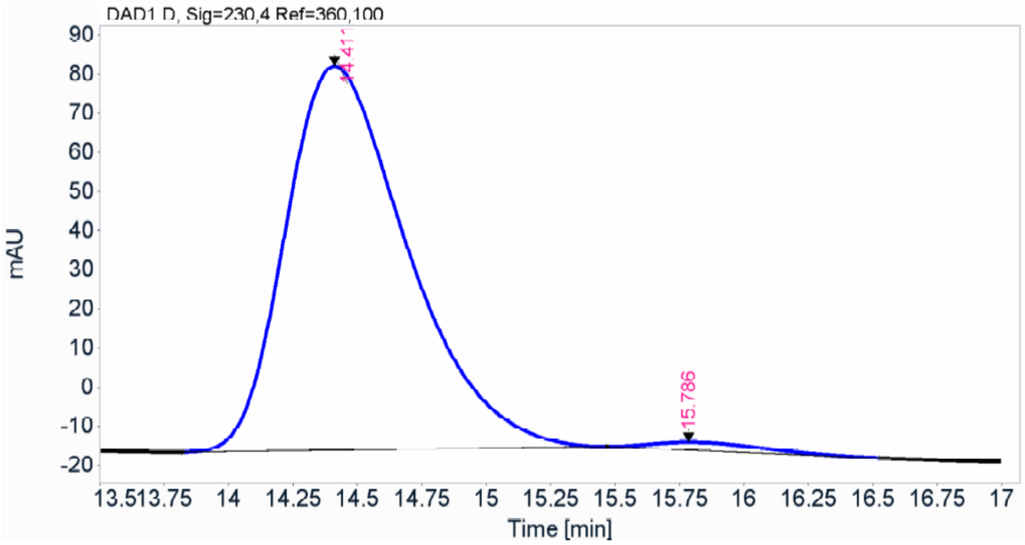

Signal:	DAD1 D, Sig=230,4 Ref=360,100				
RT [min]	Type	Width [min]	Area	Height	Area%
14.411	BB	0.5055	3225.453	98.1154	98.11
15.786	BBA	0.4687	62.013	2.0169	1.89

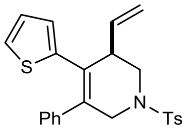

2f

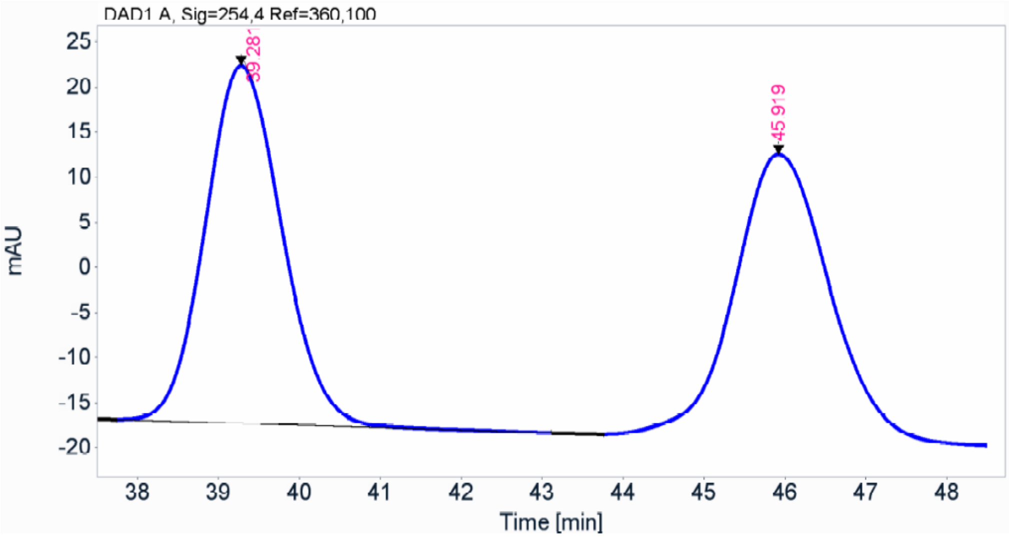

Signal: DAD1 A, Sig=254,4 Ref=360,100

RT [min]	Type	Width [min]	Area	Height	Area%
39.281	BB	1.0138	2606.877	39.7147	50.17
45.919	BB	1.2477	2588.778	31.6680	49.83

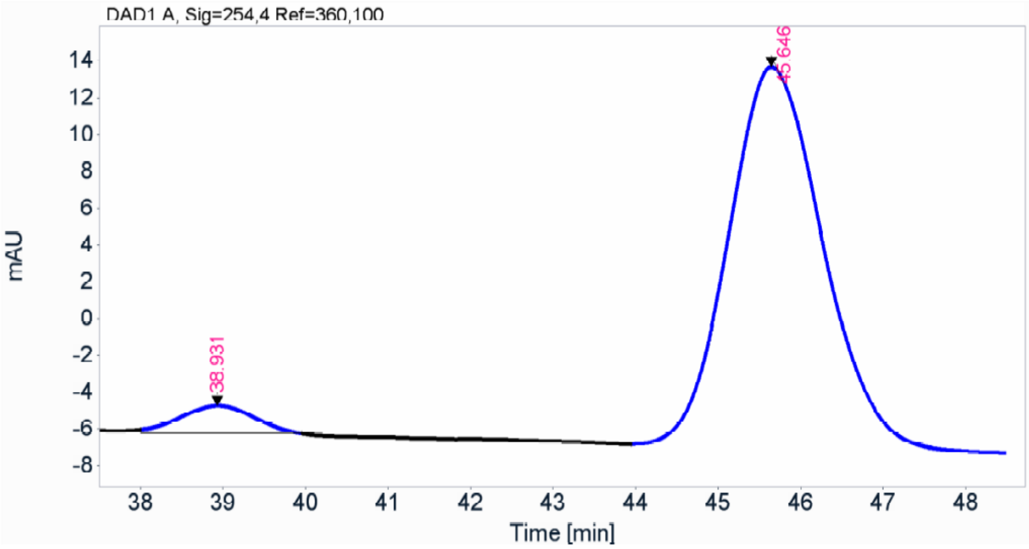

Signal: DAD1 A, Sig=254,4 Ref=360,100

RT [min]	Type	Width [min]	Area	Height	Area%
38.931	MM	1.0121	89.221	1.4693	5.17
45.646	BB	1.1997	1636.846	20.6733	94.83

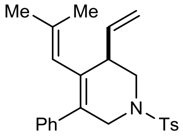

2g

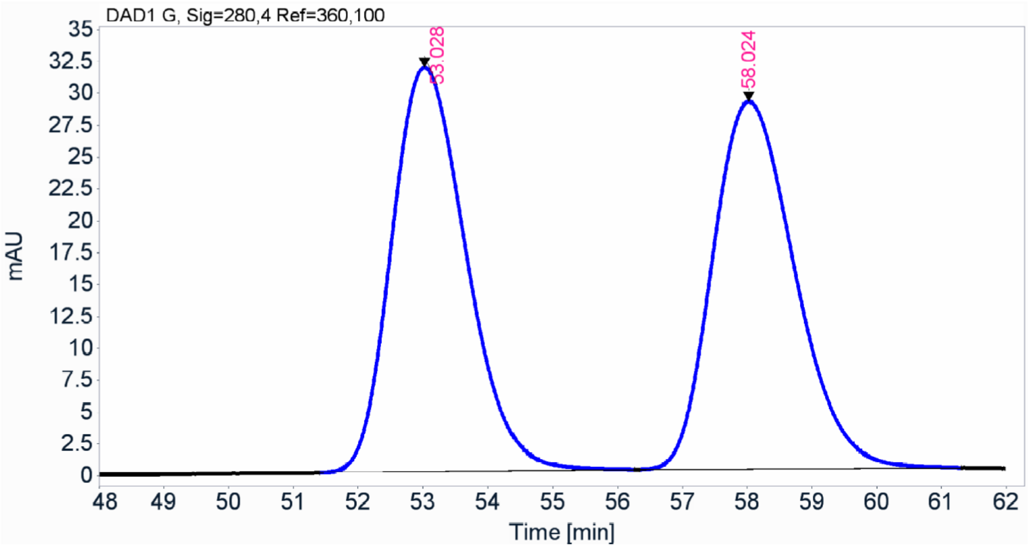

Signal:	DAD1 G, Sig=280,4 Ref=360,100				
**RT [min]**	**Type**	**Width [min]**	**Area**	**Height**	**Area%**
53.028	BB	1.2420	2528.980	31.7240	49.99
58.024	BB	1.3358	2530.060	28.8312	50.01

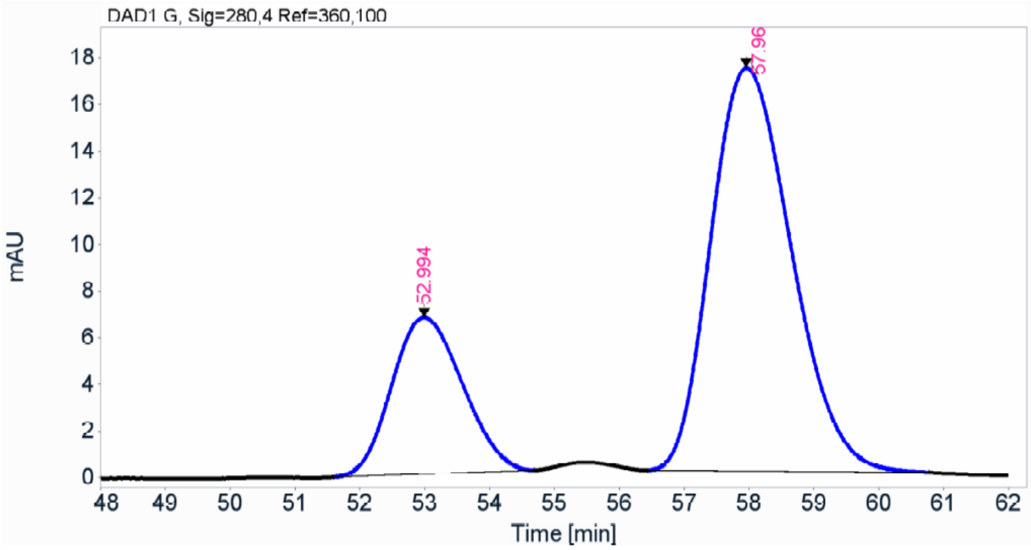

Signal:	DAD1 G, Sig=280,4 Ref=360,100				
**RT [min]**	**Type**	**Width [min]**	**Area**	**Height**	**Area%**
52.994	BB	1.0358	508.281	6.7094	25.40
57.960	BB	1.3108	1492.440	17.2939	74.60

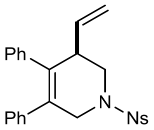

2h

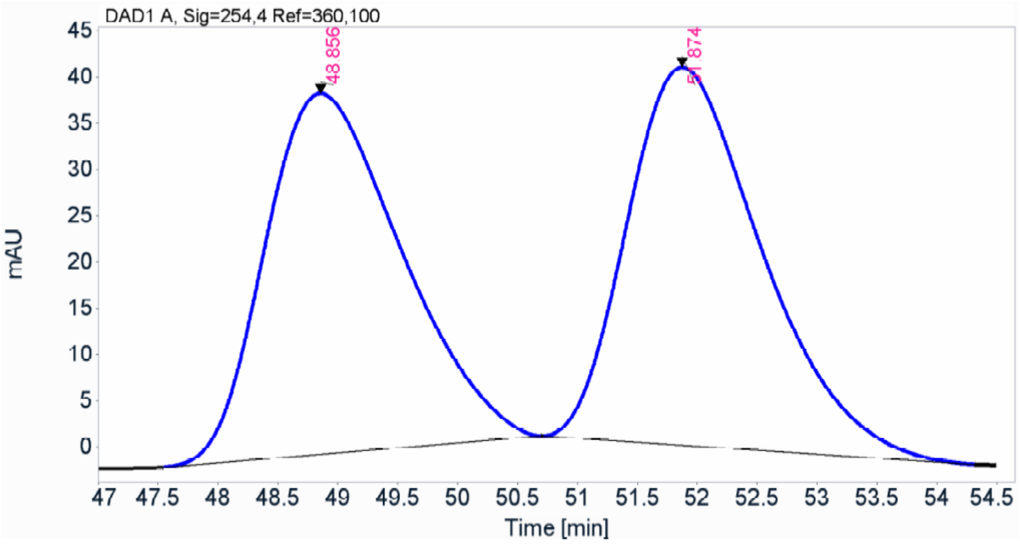

Signal:	DAD1 A, Sig=254,4 Ref=360,100				
RT [min]	Type	Width [min]	Area	Height	Area%
48.856	BB	1.1994	3075.840	39.0327	49.09
51.874	BB	1.1879	3190.441	40.9096	50.91

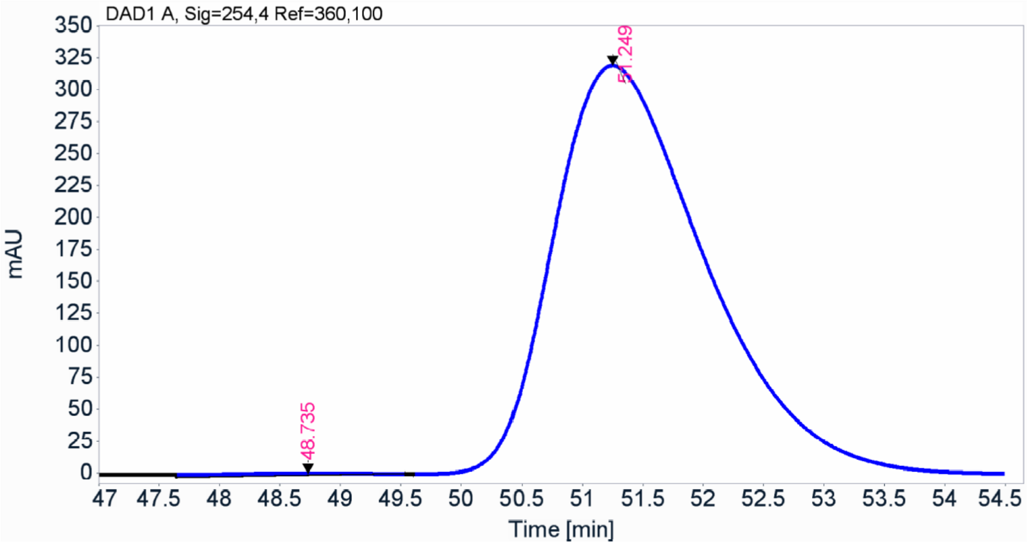

Signal:	DAD1 A, Sig=254,4 Ref=360,100				
RT [min]	Type	Width [min]	Area	Height	Area%
48.735	MM	1.1396	138.475	1.4328	0.49
51.249	BB	1.3414	27846.869	319.3287	99.51

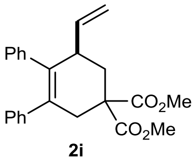

2i

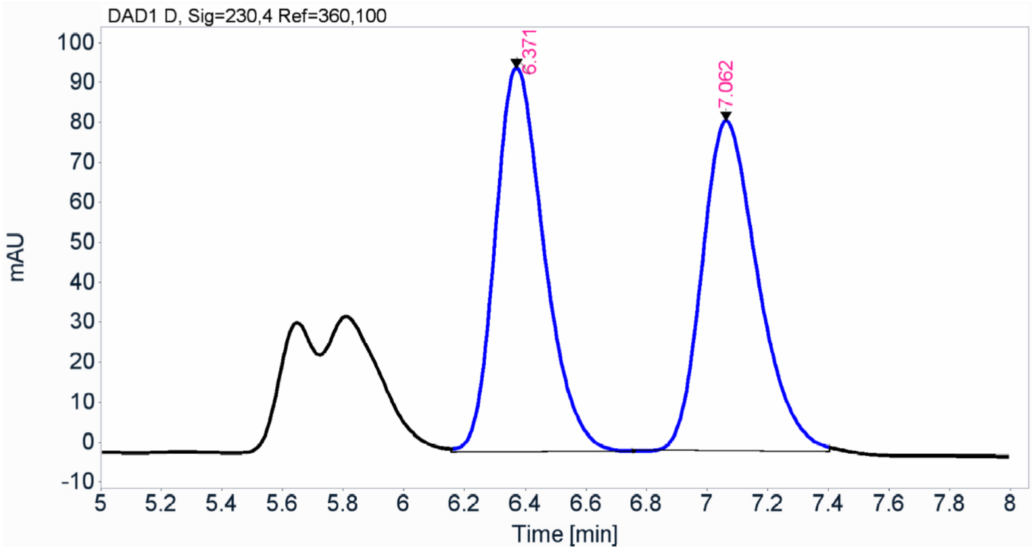

Signal:	DAD1 D, Sig=230,4 Ref=360,100				
RT [min]	Type	Width [min]	Area	Height	Area%
6.371	VB	0.1714	1064.633	95.9647	50.20
7.062	MM	0.2133	1056.120	82.5256	49.80

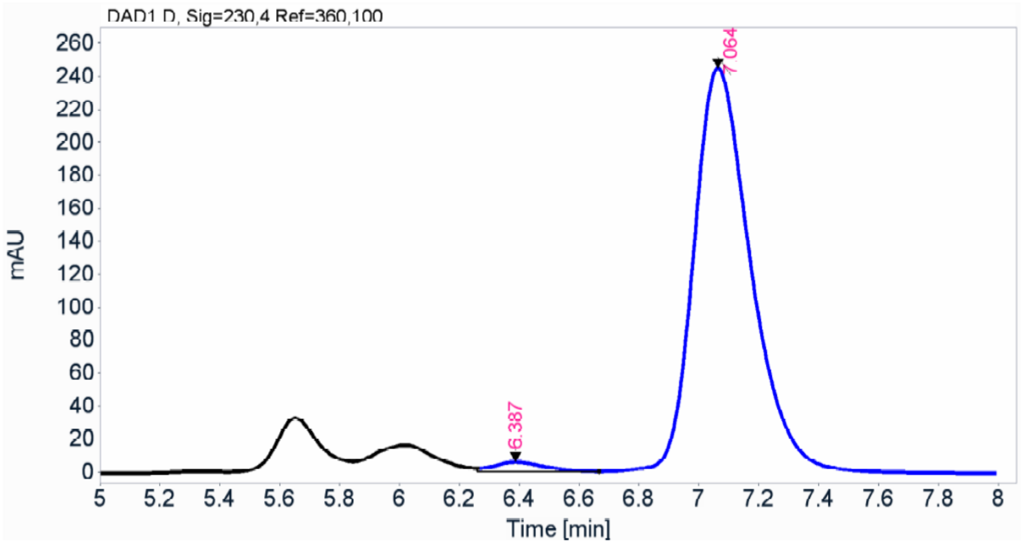

Signal:	DAD1 D, Sig=230,4 Ref=360,100				
RT [min]	Type	Width [min]	Area	Height	Area%
6.387	VB	0.1747	67.885	5.7935	2.06
7.064	BB	0.2017	3233.022	245.3988	97.94

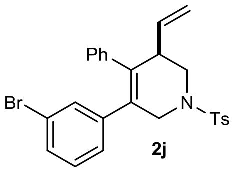

2j

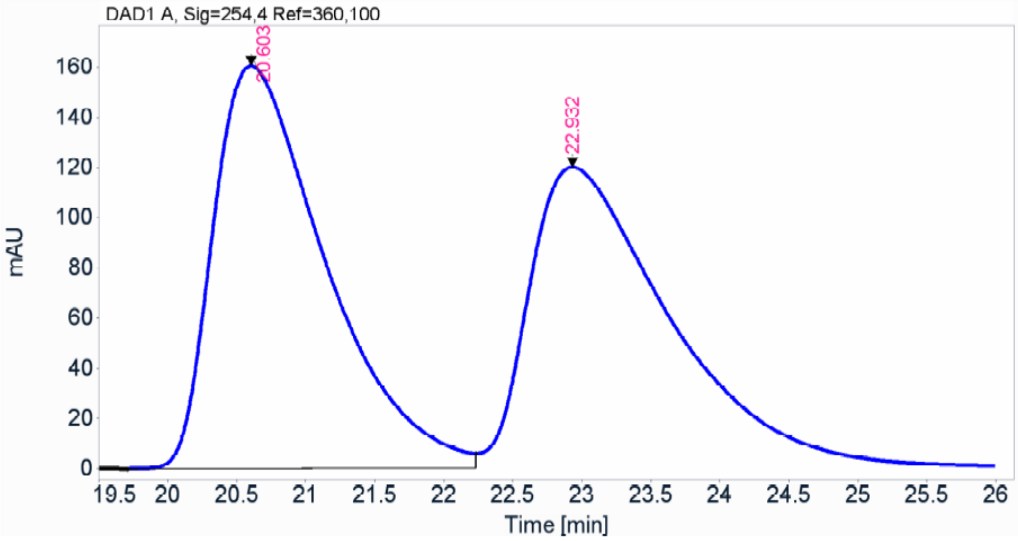

Signal: DAD1 A, Sig=254,4 Ref=360,100

RT [min]	Type	Width [min]	Area	Height	Area%
20.603	BV	0.8609	9066.855	160.5438	51.66
22.932	VB	1.0437	8484.602	119.8890	48.34

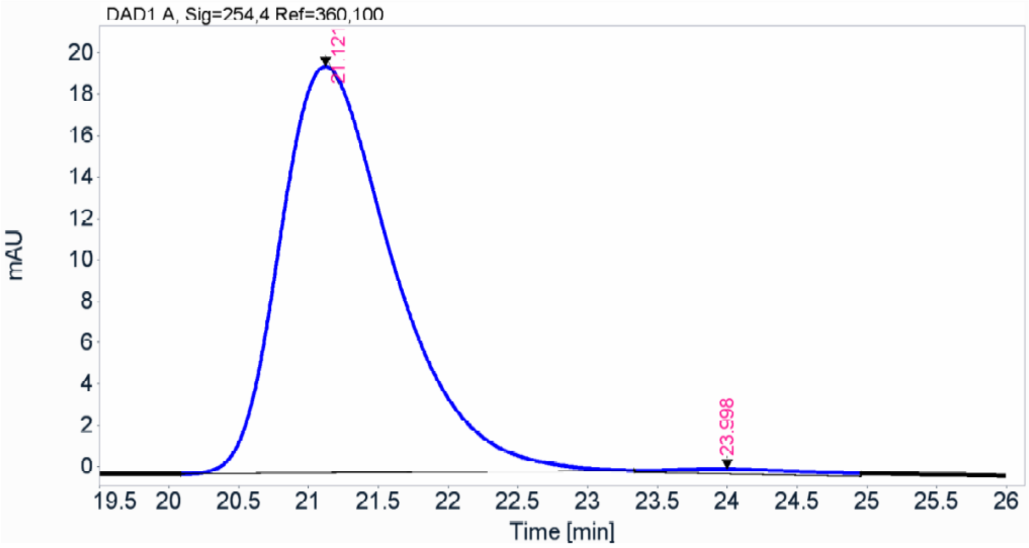

Signal: DAD1 A, Sig=254,4 Ref=360,100

RT [min]	Type	Width [min]	Area	Height	Area%
21.121	BB	0.8617	1110.336	19.6354	98.44
23.998	MM	1.2105	17.632	0.2428	1.56

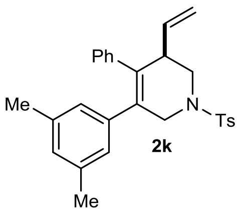

2k

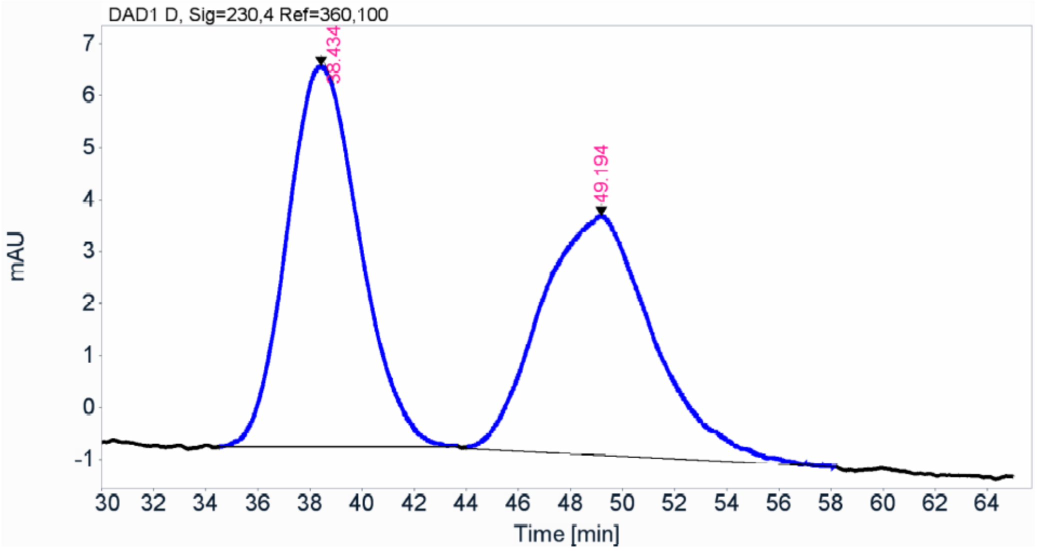

Signal:	DAD1 D, Sig=230,4 Ref=360,100				
**RT [min]**	**Type**	**Width [min]**	**Area**	**Height**	**Area%**
38.434	MM T	3.2365	1421.410	7.3196	50.78
49.194	MM T	4.9900	1377.488	4.6008	49.22

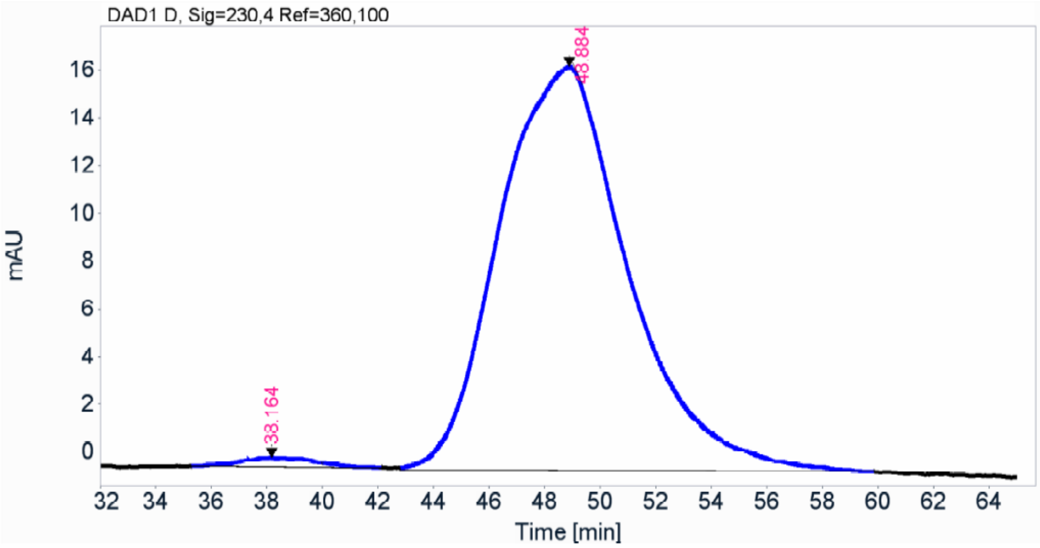

Signal:	DAD1 D, Sig=230,4 Ref=360,100				
**RT [min]**	**Type**	**Width [min]**	**Area**	**Height**	**Area%**
38.164	MM T	3.1245	71.723	0.3826	1.30
48.884	MM T	5.3570	5428.820	16.8900	98.70

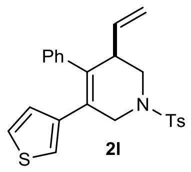

2l

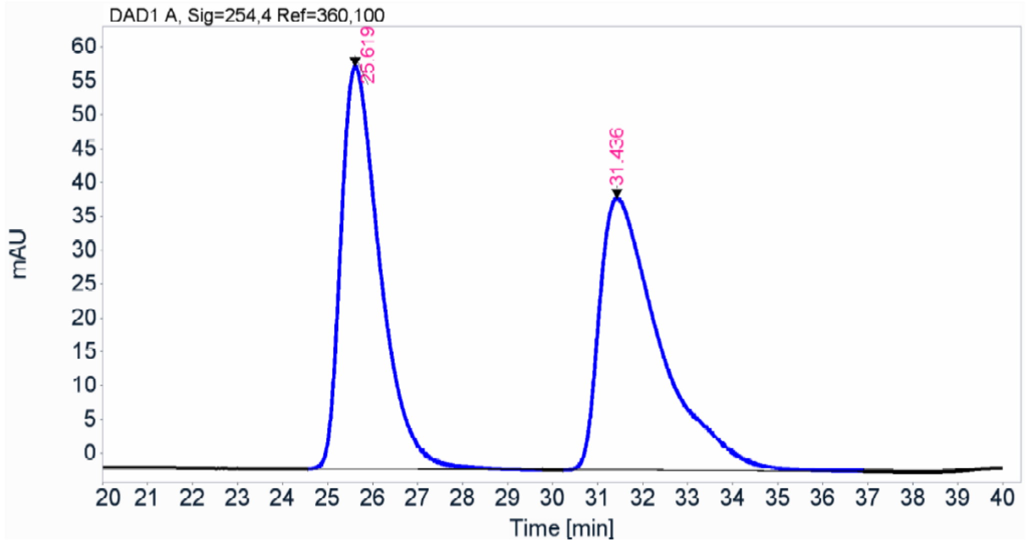

**Signal:**	DAD1 A, Sig=254,4 Ref=360,100				
**RT [min]**	**Type**	**Width [min]**	**Area**	**Height**	**Area%**
25.619	BB	0.9150	3586.155	59.3462	49.03
31.436	BB	1.3262	3728.228	40.1189	50.97

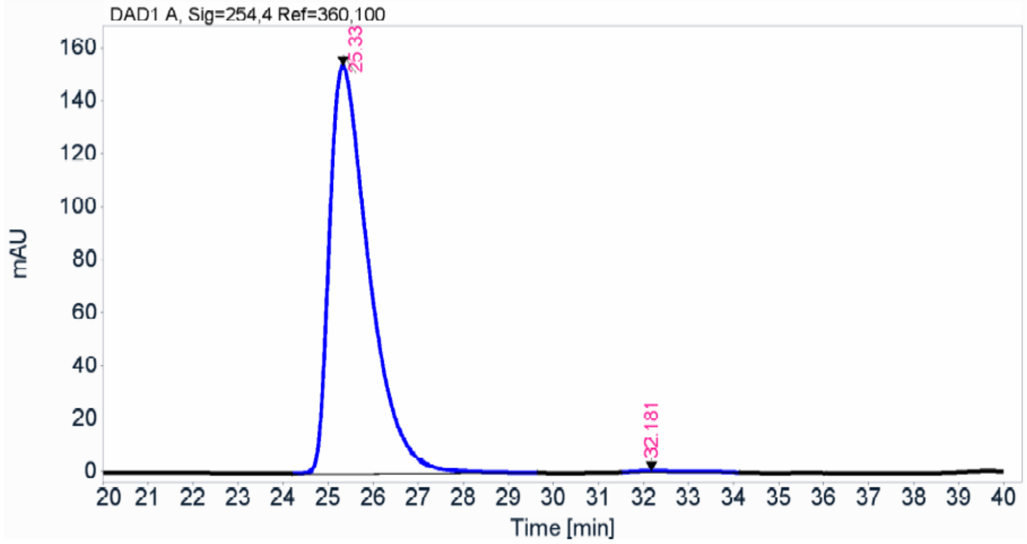

**Signal:**	DAD1 A, Sig=254,4 Ref=360,100				
**RT [min]**	**Type**	**Width [min]**	**Area**	**Height**	**Area%**
25.330	MM T	1.0209	9438.657	154.0860	99.50
32.181	MM T	1.5743	47.537	0.5033	0.50

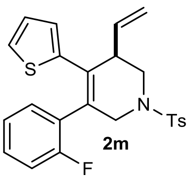

2m

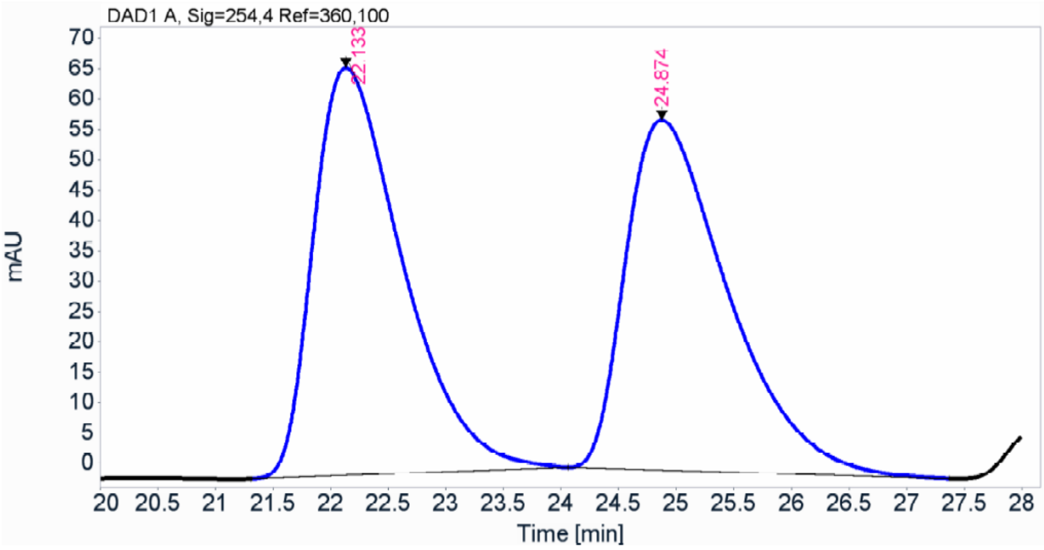

Signal:	DAD1 A, Sig=254,4 Ref=360,100				
RT [min]	Type	Width [min]	Area	Height	Area%
22.133	BB	0.8209	3608.438	67.1572	50.39
24.874	BB	0.9364	3552.758	57.6990	49.61

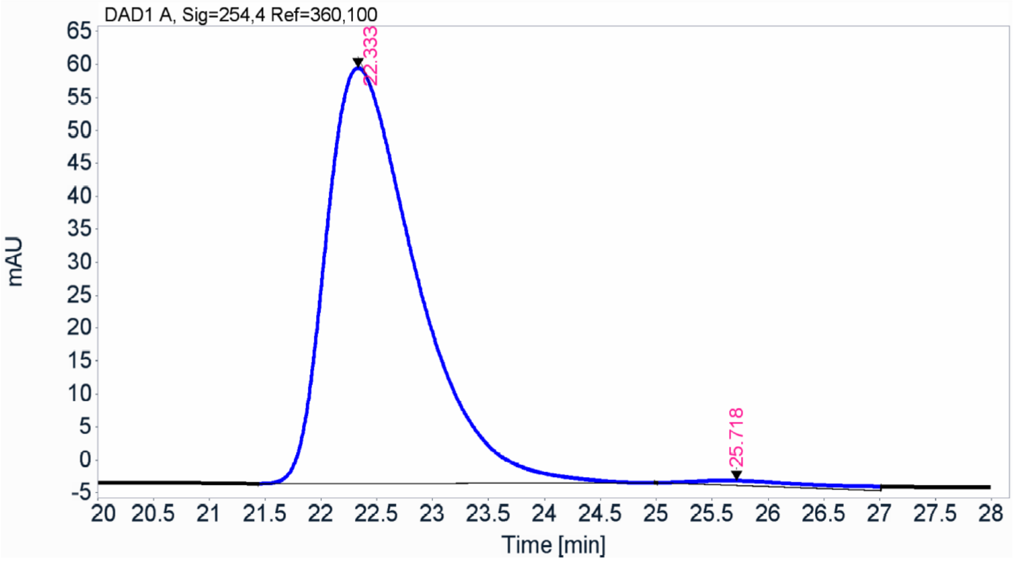

Signal:	DAD1 A, Sig=254,4 Ref=360,100				
RT [min]	Type	Width [min]	Area	Height	Area%
22.333	BB	0.8503	3536.500	63.0549	98.18
25.718	MM	1.3930	65.446	0.7830	1.82

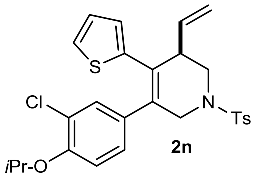

2n

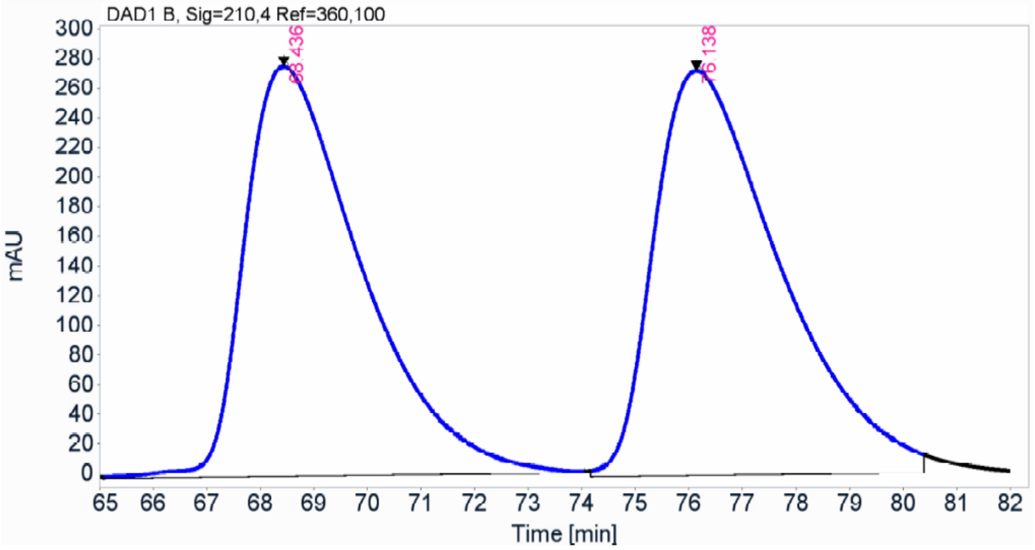

Signal:	DAD1 B, Sig=210,4 Ref=360,100				
RT [min]	Type	Width [min]	Area	Height	Area%
68.436	MM	2.5224	41837.422	276.4390	48.54
76.138	MM	2.7092	44355.672	272.8735	51.46

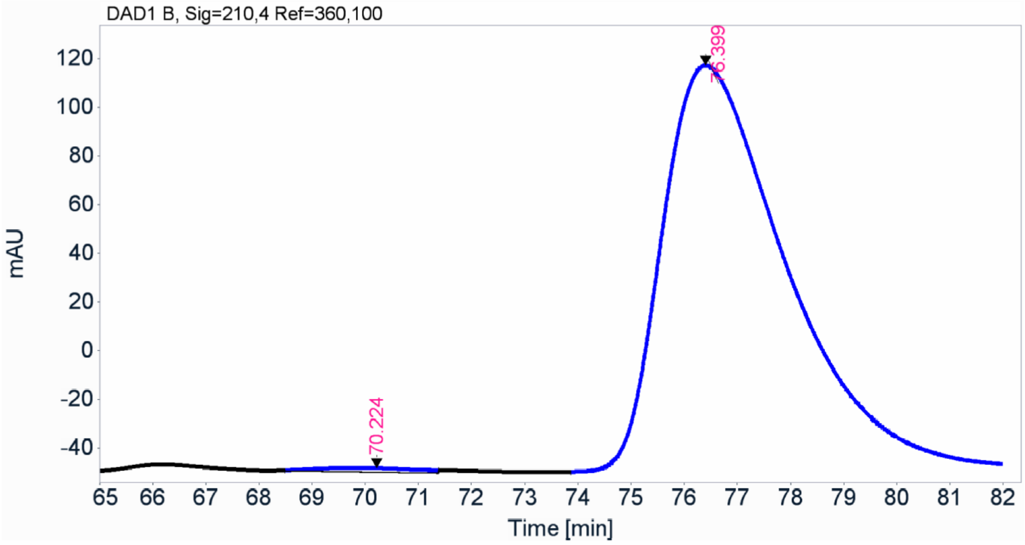

Signal:	DAD1 B, Sig=210,4 Ref=360,100				
RT [min]	Type	Width [min]	Area	Height	Area%
70.224	MM	2.1920	219.380	1.6680	0.80
76.399	BB	2.3722	27121.809	166.6931	99.20

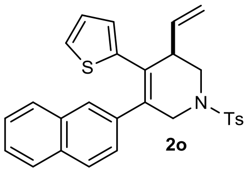

2o

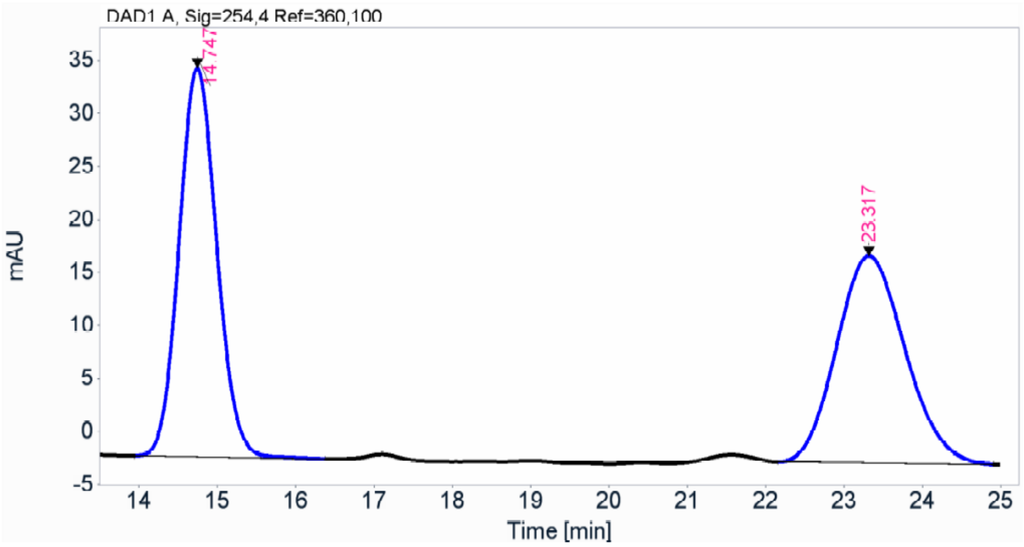

Signal:	DAD1 A, Sig=254,4 Ref=360,100				
RT [min]	Type	Width [min]	Area	Height	Area%
14.747	BB	0.5194	1223.264	36.6491	50.75
23.317	BB	0.9345	1187.036	19.5453	49.25

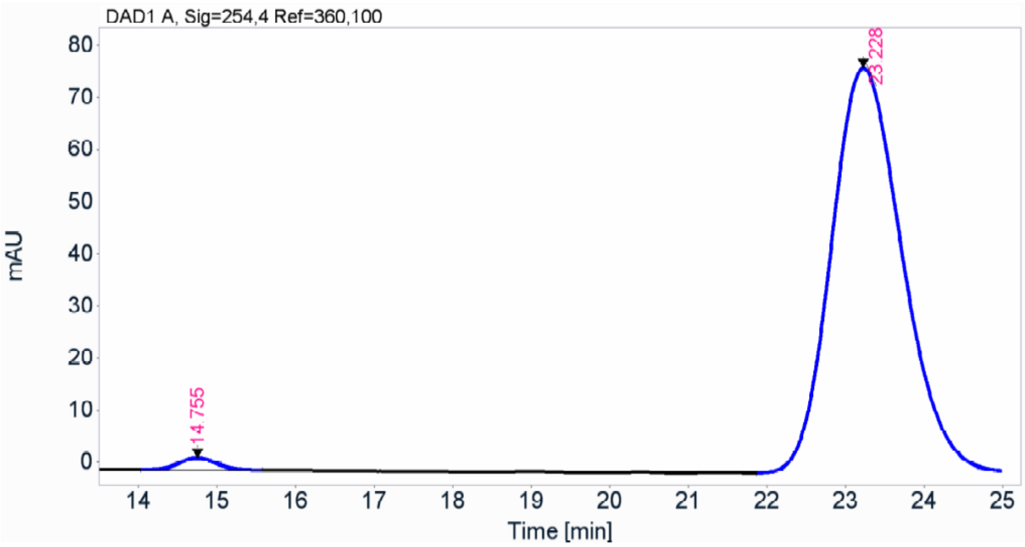

Signal:	DAD1 A, Sig=254,4 Ref=360,100				
RT [min]	Type	Width [min]	Area	Height	Area%
14.755	BB	0.5075	76.973	2.3177	1.57
23.228	BB	0.9728	4830.931	77.5437	98.43

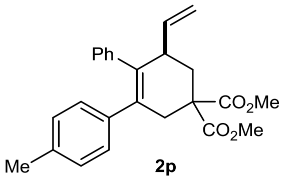

2p

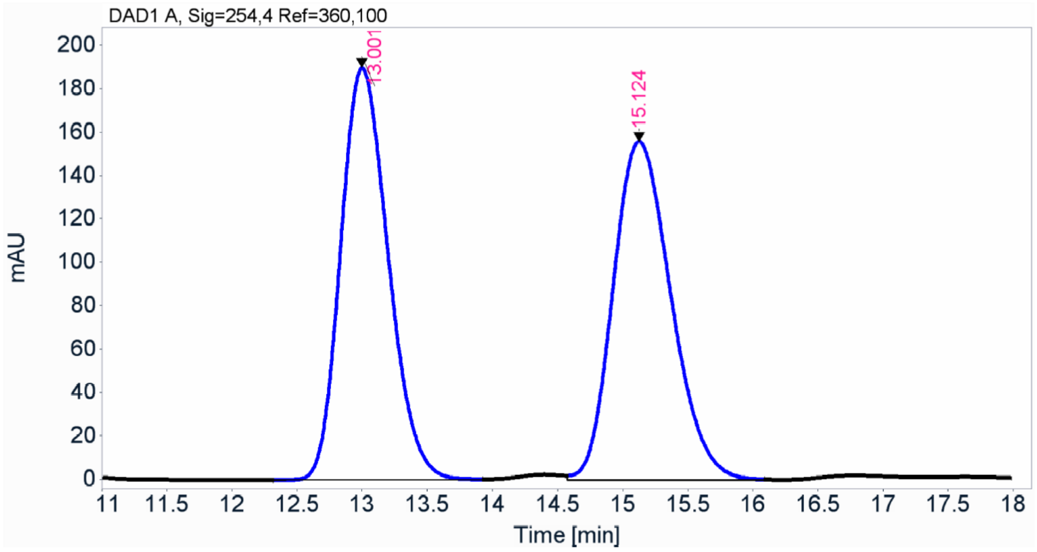

Signal:	DAD1 A, Sig=254,4 Ref=360,100				
RT [min]	Type	Width [min]	Area	Height	Area%
13.001	BB	0.3955	4803.970	189.6460	49.91
15.124	MM T	0.5149	4821.157	156.0419	50.09

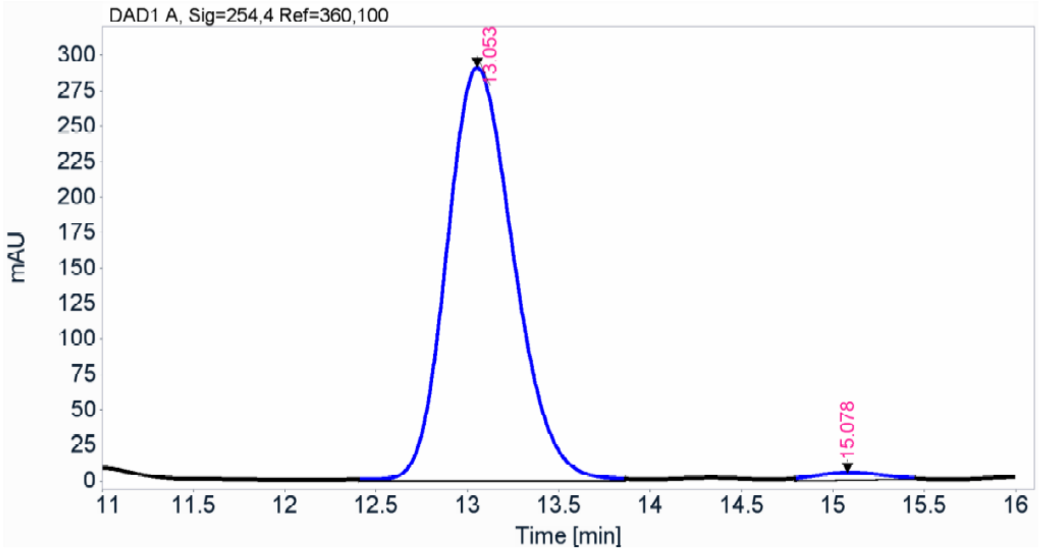

Signal:	DAD1 A, Sig=254,4 Ref=360,100				
RT [min]	Type	Width [min]	Area	Height	Area%
13.053	MM T	0.4242	7417.531	291.4574	98.13
15.078	MM T	0.4369	141.092	5.3825	1.87

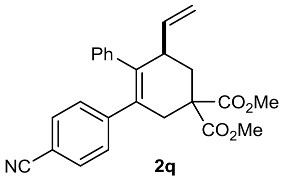

2q

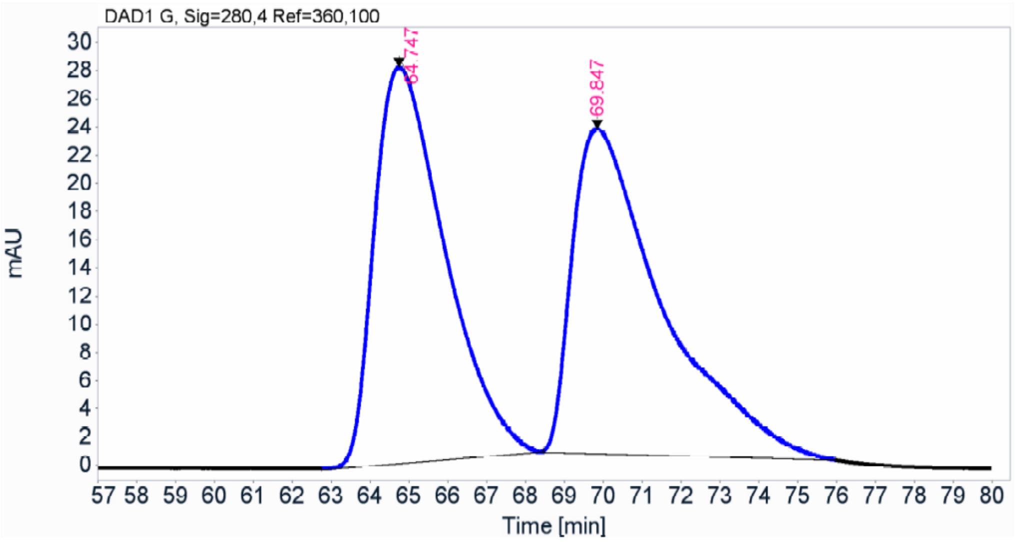

Signal: DAD1 G, Sig=280,4 Ref=360,100

RT [min]	Type	Width [min]	Area	Height	Area%
64.747	MM T	2.1637	3548.377	28.1221	48.92
69.847	MM T	2.6807	3705.299	23.0371	51.08

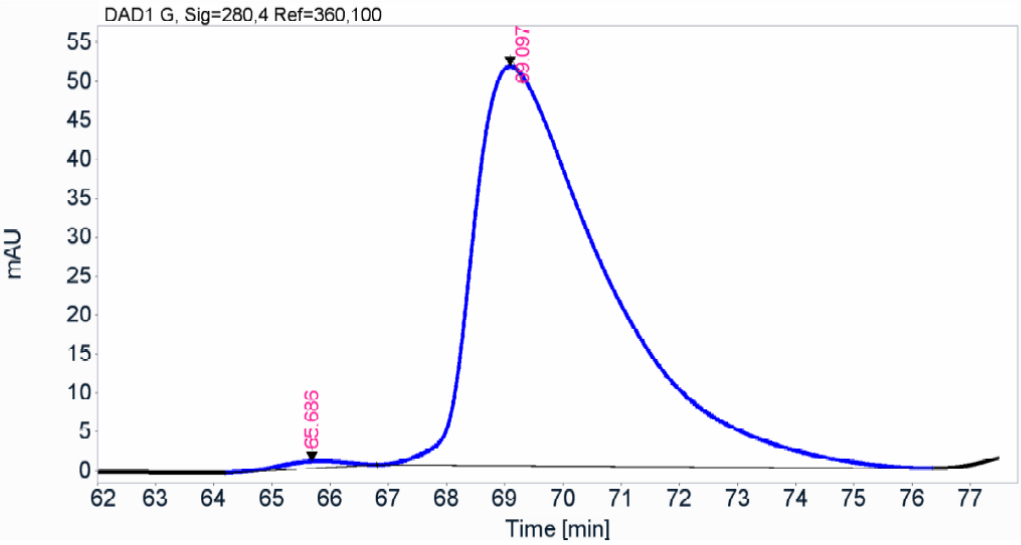

Signal: DAD1 G, Sig=280,4 Ref=360,100

RT [min]	Type	Width [min]	Area	Height	Area%
65.686	MM T	1.3110	68.709	0.8735	0.85
69.097	BB	2.1598	8046.288	51.3013	99.15

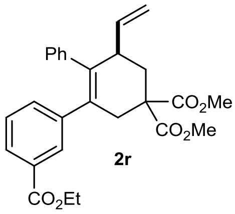

2r

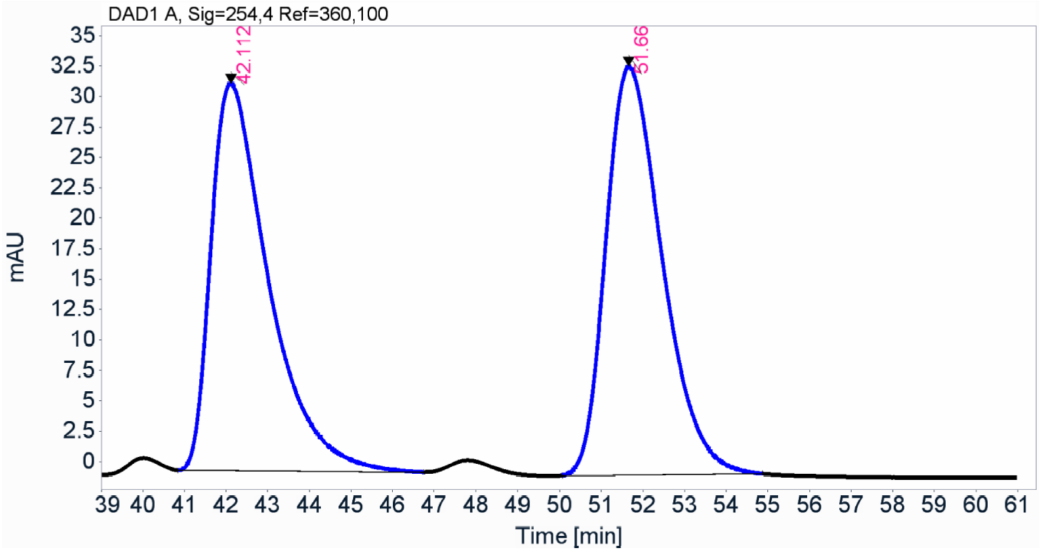

Signal: DAD1 A, Sig=254,4 Ref=360,100

RT [min]	Type	Width [min]	Area	Height	Area%
42.112	BB	1.4014	3058.093	31.8149	49.21
51.660	MM T	1.5686	3156.541	33.5394	50.79

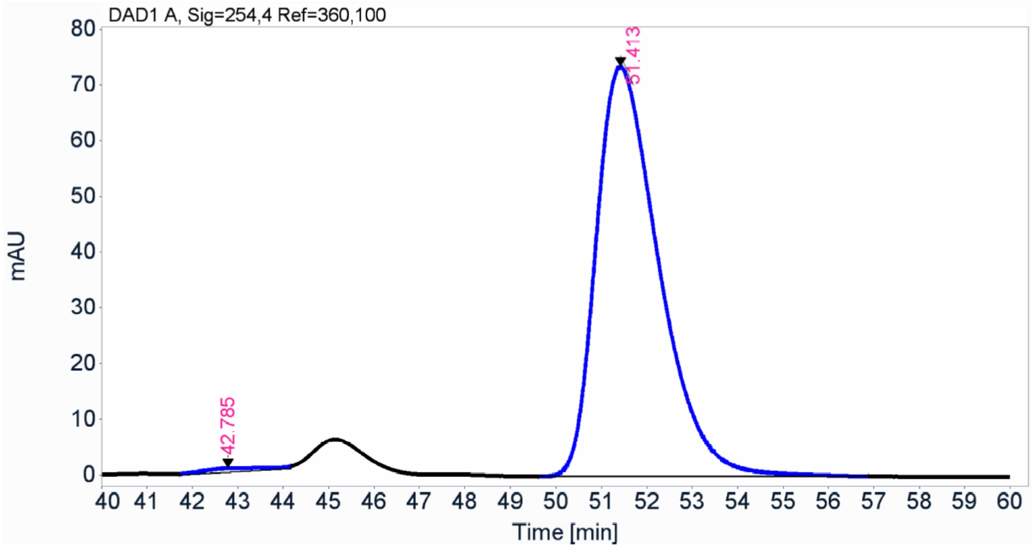

Signal: DAD1 A, Sig=254,4 Ref=360,100

RT [min]	Type	Width [min]	Area	Height	Area%
42.785	MM T	1.4803	72.658	0.7346	1.02
51.413	BB	1.4412	7050.078	73.3622	98.98

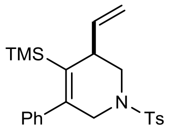

2s

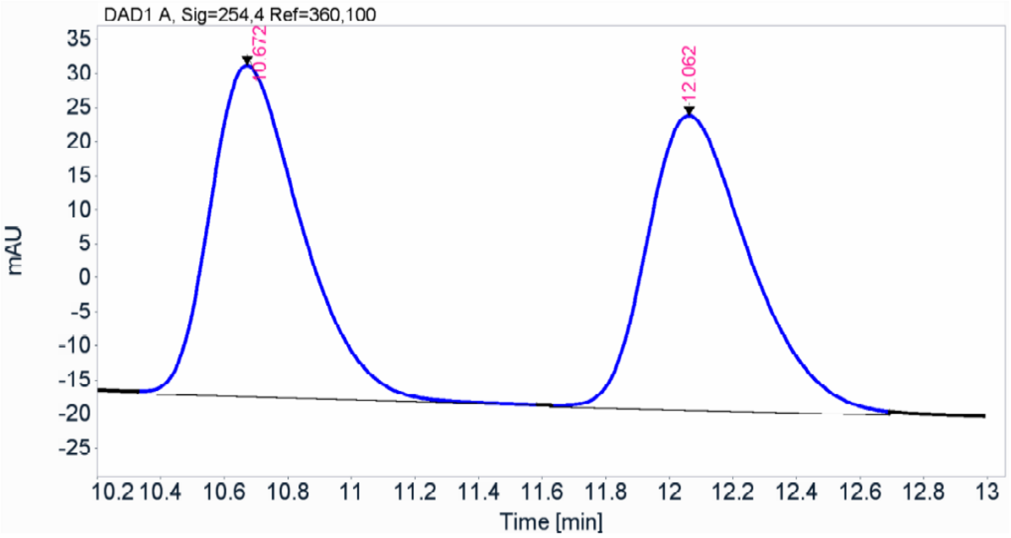

Signal:	DAD1 A, Sig=254,4 Ref=360,100				
**RT [min]**	**Type**	**Width [min]**	**Area**	**Height**	**Area%**
10.672	VB	0.3042	967.419	48.6107	49.86
12.062	MM	0.3750	972.659	43.2333	50.14

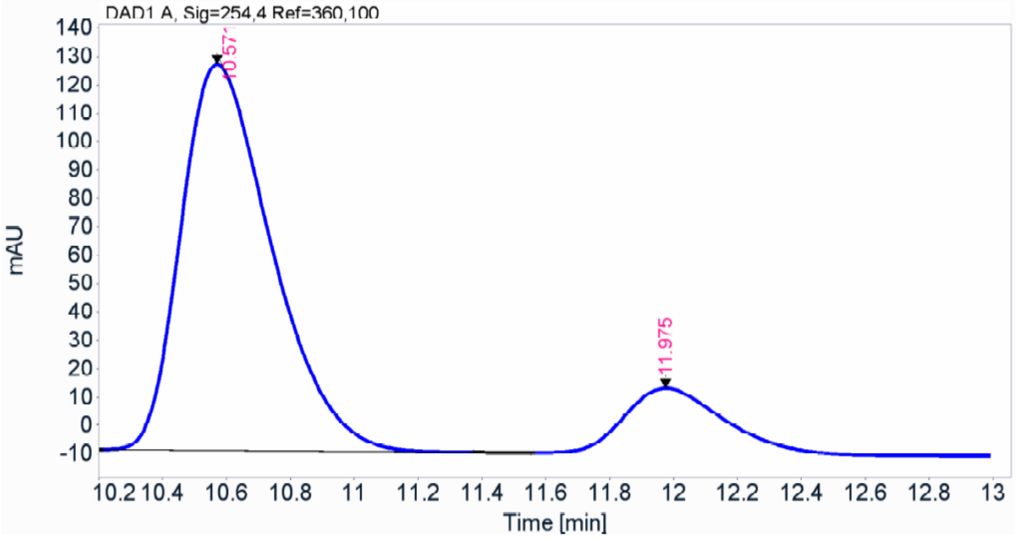

Signal:	DAD1 A, Sig=254,4 Ref=360,100				
**RT [min]**	**Type**	**Width [min]**	**Area**	**Height**	**Area%**
10.571	BB	0.3036	2676.275	135.9793	84.25
11.975	BB	0.3303	500.215	23.1284	15.75

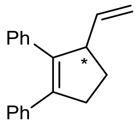

7

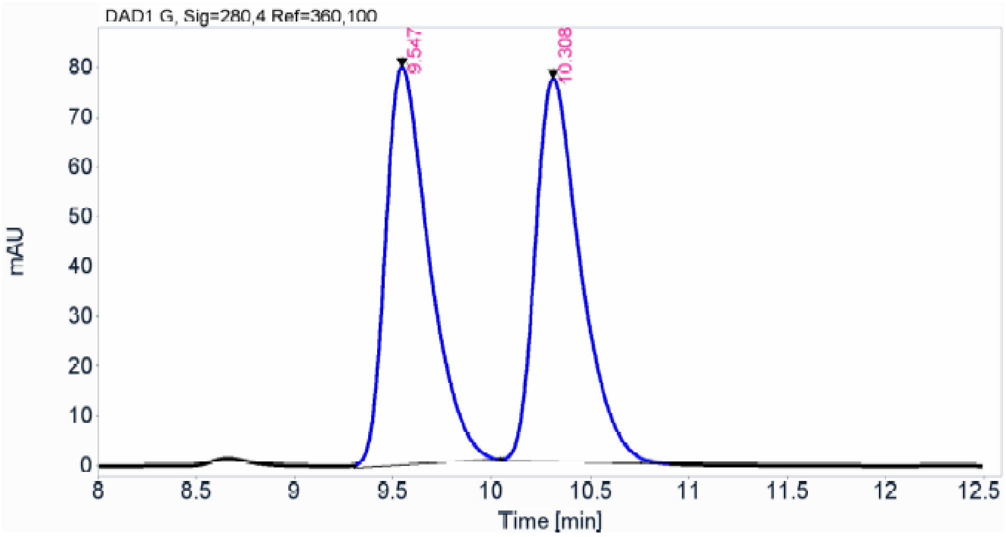

Signal:	DAD1 G, Sig=280,4 Ref=360,100				
RT [min]	Type	Width [min]	Area	Height	Area%
9.547	MM T	0.2576	1236.946	80.0158	50.15
10.308	MM T	0.2661	1229.396	77.0028	49.85

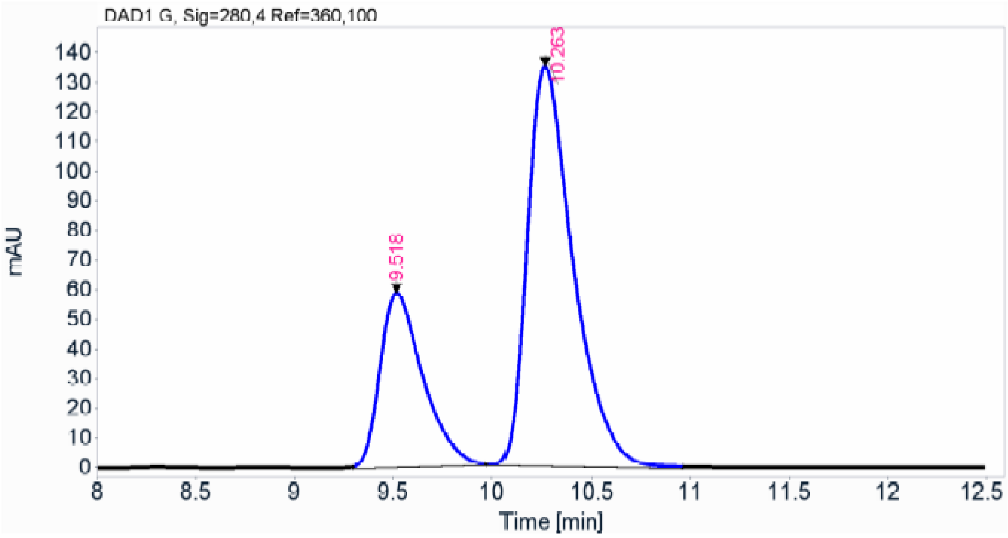

Signal:	DAD1 G, Sig=280,4 Ref=360,100				
RT [min]	Type	Width [min]	Area	Height	Area%
9.518	MM T	0.2526	892.558	58.9009	29.07
10.263	MM T	0.3000	2177.604	134.8732	70.93

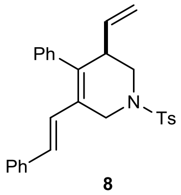

8

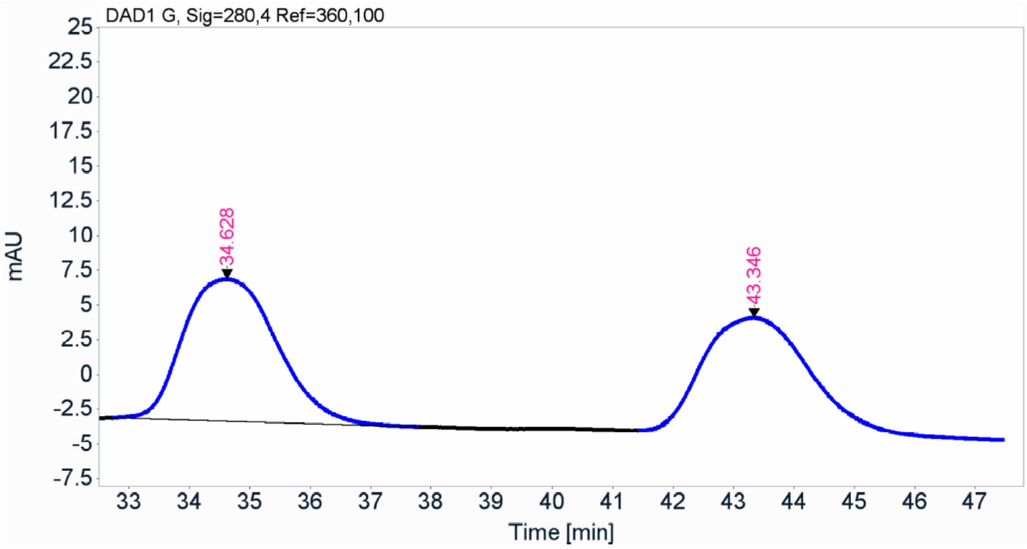

Signal: DAD1 G, Sig=280,4 Ref=360,100

RT [min]	Type	Width [min]	Area	Height	Area%
34.628	BB	1.2604	1096.038	10.2589	51.74
43.346	BB	1.4498	1022.286	8.3065	48.26

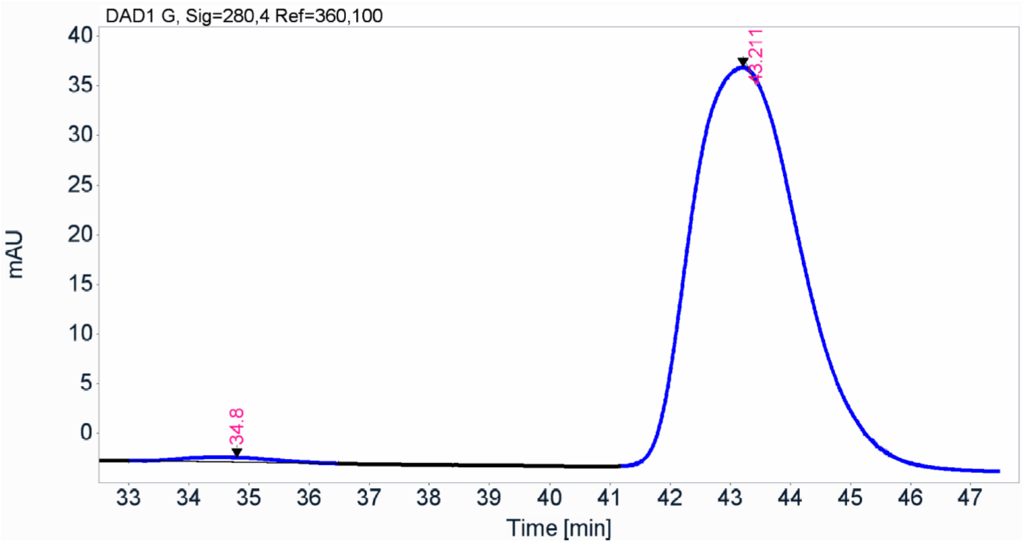

Signal: DAD1 G, Sig=280,4 Ref=360,100

RT [min]	Type	Width [min]	Area	Height	Area%
34.800	MM	1.8166	53.896	0.4945	1.05
43.211	BB	1.9796	5067.077	40.3348	98.95

References

(1) Schrems, M. G.; Pfaltz, A. *Chem. Commun.* **2009**, 6210.

(2) Mitchell, M.; Qaio, L.; Wong, C.-H. *Adv. Synth. Catal.* **2001**, *343*, 596.

(3) Ito, Y.; Yoshimatsu, M. *Chem. Lett.* **2014**, *43*, 1758.

(4) Kavanagh, Y.; O'Brien, M.; Evans, P. *Tetrahedron* **2009**, *65*, 8259.

(5) Schiller, R.; Pour, M.; Fáková, H.; Kuneš, J.; Císařová, I. *J. Org. Chem.* **2004**, *69*, 6761.

(6) Shu, X. Z.; Huang, S.; Shu, D.; Guzei, I. A.; Tang, W. *Angew. Chem. Int. Ed.* **2011**, *50*, 8153.

(7) Park, Y.; Kim, S. Y.; Park, J. H.; Cho, J.; Kang, Y. K.; Chung, Y. K. *Chem. Commun.* **2011**, 5190.

(8) Yeh, M. C. P.; Lin, M. N.; Chang, W. J.; Liou, J. L.; Shih, Y. F. *J. Org. Chem.* **2010**, *75*, 6031.

(9) Yu, Z.; Liu, L.; Zhang, J. *Chem. Eur. J.* **2016**, *22*, 8488.

(10) Gansäuer, A.; Shi, L.; Otte, M. *J. Am. Chem. Soc.* **2010**, *132*, 11858.
